# Systematic revision of the genus *Peronia* Fleming, 1822 (Gastropoda, Euthyneura, Pulmonata, Onchidiidae)

**DOI:** 10.3897/zookeys.972.52853

**Published:** 2020-10-01

**Authors:** Benoît Dayrat, Tricia C. Goulding, Deepak Apte, Sadar Aslam, Adam Bourke, Joseph Comendador, Munawar Khalil, Xuân Quảng Ngô, Siong Kiat Tan, Shau Hwai Tan

**Affiliations:** 1 Department of Biology, Pennsylvania State University, University Park, PA 16802, USA; 2 Bombay Natural History Society, Hornbill House, Opp. Lion Gate, Shaheed Bhagat Singh Road, Mumbai 400 001, Maharashtra, India; 3 Centre of Excellence in Marine Biology, University of Karachi, Karachi 75270, Pakistan; 4 College of Engineering, Information Technology and the Environment, Charles Darwin University, Ellengowan Dr, Casuarina, NT 0810, Australia; 5 National Museum of the Philippines, Taft Ave, Ermita, Manila, 1000, Metro Manila, Philippines; 6 Department of Marine Science, Universitas Malikussaleh, Reuleut Main Campus, Kecamatan Muara Batu, North Aceh, Aceh, 24355, Indonesia; 7 Institute of Tropical Biology, Vietnam Academy of Science and Technology, 85 Tran Quoc Toan Street, District 3, Ho Chi Minh City, Vietnam; 8 Graduate University of Science and Technology, Vietnam Academy of Science and Technology, 18 Hoang Quoc Viet, Cau Giay, Hanoi, Vietnam; 9 Lee Kong Chian Natural History Museum, 2 Conservatory Dr, National University of Singapore, 117377, Singapore; 10 Centre for Marine and Coastal Studies, Universiti Sains Malaysia, 11800, Minden Penang, Malaysia; 11 Marine Science Laboratory, School of Biological Sciences, Universiti Sains Malaysia, 11800, Minden Penang, Malaysia

**Keywords:** Biodiversity, Coral Triangle, Indo-West Pacific, integrative taxonomy, mangrove, South-East Asia

## Abstract

The genus *Peronia* Fleming, 1822 includes all the onchidiid slugs with dorsal gills. Its taxonomy is revised for the first time based on a large collection of fresh material from the entire Indo-West Pacific, from South Africa to Hawaii. Nine species are supported by mitochondrial (COI and 16S) and nuclear (ITS2 and 28S) sequences as well as comparative anatomy. All types available were examined and the nomenclatural status of each existing name in the genus is addressed. Of 31 *Peronia* species-group names available, 27 are regarded as invalid (twenty-one synonyms, sixteen of which are new, five *nomina dubia*, and one homonym), and four as valid: *Peronia peronii* (Cuvier, 1804), *Peronia verruculata* (Cuvier, 1830), *Peronia platei* (Hoffmann, 1928), and *Peronia madagascariensis* (Labbé, 1934a). Five new species names are created: *P. griffithsi* Dayrat & Goulding, **sp. nov.**, *P. okinawensis* Dayrat & Goulding, **sp. nov.**, *P. setoensis* Dayrat & Goulding, **sp. nov.**, *P. sydneyensis* Dayrat & Goulding, **sp. nov.**, and *P. willani* Dayrat & Goulding, **sp. nov.***Peronia* species are cryptic externally but can be distinguished using internal characters, with the exception of *P. platei* and *P. setoensis*. The anatomy of most species is described in detail here for the first time. All the secondary literature is commented on and historical specimens from museum collections were also examined to better establish species distributions. The genus *Peronia* includes two species that are widespread across the Indo-West Pacific (*P. verruculata* and *P. peronii*) as well as endemic species: *P. okinawensis* and *P. setoensis* are endemic to Japan, and *P. willani* is endemic to Northern Territory, Australia. Many new geographical records are provided, as well as a key to the species using morphological traits.

## Introduction

Onchidiid slugs live in the intertidal, worldwide, except at the poles. Their larvae are released in sea water and, in that sense, onchidiids are truly marine. As adult slugs, however, they breathe air through a lung and die if they are immersed in water for too long. The slugs of the genus *Peronia* Fleming, 1822a are found across the entire tropical and subtropical Indo-West Pacific, from South Africa to Hawaii. They primarily inhabit rocky shores and coral rubble, can occasionally be found on muddy sand, but are typically not found inside mangrove forests.

The genus *Peronia* includes all onchidiid slugs with a dorsal notum bearing ramified appendages, or dorsal gills, which are most easily seen when animals are relaxed. Dorsal gills tend to be retracted when live animals are crawling at low tide, and they can be hard to see on specimens preserved without relaxation. In fact, Cuvier did not mention dorsal gills in the original description of *Onchidium peronii* Cuvier, 1804, the first *Peronia* species ever recognized. Dorsal gills were first illustrated by Savigny (1817: pl. 2, fig. 3.5) on a plate of gastropods from the Red Sea in the famous *Description de l’Egypte*, and first described by Audouin (1826: 19) in the explanation of Savigny’s plate. Dorsal gills are either present or absent on the dorsal notum of onchidiid slugs, and all slugs with dorsal gills belong to the genus *Peronia* (Dayrat et al. 2017: 1861).

For the past sixty years or so, authors have accepted only two valid *Peronia* species names for two species broadly distributed across the Indo-West Pacific (e.g., Solem 1959: 38–39; Marcus and Marcus 1970: 213–214; Britton 1984: 183): *P. peronii* (Cuvier, 1804) and *P. verruculata* (Cuvier, 1830). However, the differences between *P. peronii* and *P. verruculata* have remained unclear, to say the least, and both names have been used arbitrarily. More importantly, 31 species-group names are available for onchidiids with dorsal gills and their exact application has never been addressed. Indeed, the taxonomy of the genus *Peronia* is so challenging that people have avoided it for decades, and Labbé (1934a) is the last author who created species names for onchidiids with dorsal gills, except for the recent *Peronia persiae*Maniei et al., 2020a, regarded in the present work as a synonym of *P. verruculata*. The taxonomy of the genus *Peronia* is comprehensively revised here for the first time. The goals of the present revision are to determine how many *Peronia* species there are, where they are distributed, how they are related, how they can be identified, how many of the available species names are valid, and to create new names if needed.

All the available types of all onchidiid species were re-examined in the context of our revision of the whole family (Dayrat 2009; Dayrat et al. 2016, 2017, 2018, 2019a, b, c, d; Dayrat and Goulding 2017; Goulding et al. 2018a, b, c), which served as a basis to establish a complete list of all the species names available in the genus *Peronia*. For the sake of clarity, important features (especially intestinal loops) of the types of *Peronia* nominal species are illustrated here. In many cases, lectotypes are designated in order to clarify the application of species names.

Fresh material was collected across the entire Indo-West Pacific, from South Africa to Japan, Hawaii, and eastern Australia. Special attention was paid to collecting fresh material from type localities. Specimens from which DNA could be extracted were also obtained from museum collections (the first author visited many collections around the world). Old museum specimens from which DNA could not be extracted were also examined, especially in cases of interesting geographical records or when specimens were included in important onchidiid studies (Semper 1880–1885; Plate 1893; Hoffmann 1928; Labbé 1934a).

Because they are notoriously cryptic, *Peronia* species were first delineated using DNA sequences. Then, the anatomy of the specimens was examined in order to determine diagnostic characters for each species as well as individual variation. As in our previous revisions (Dayrat et al. 2016, 2017, 2018, 2019a, b, c, d; Dayrat and Goulding 2017; Goulding et al. 2018a, b, c), both mitochondrial and nuclear DNA sequences were used for species delineation and relationships.

Nine *Peronia* species are recognized here, five of which are new to science: *P. griffithsi* Dayrat & Goulding, sp. nov., *P. madagascariensis* (Labbé, 1934a), *P. okinawensis* Dayrat & Goulding, sp. nov., *P. peronii* (Cuvier, 1804), *P. platei* (Hoffmann, 1928), *P. setoensis* Dayrat & Goulding, sp. nov., *P. sydneyensis* Dayrat & Goulding, sp. nov., *P. verruculata* (Cuvier, 1830), and *P. willani* Dayrat & Goulding, sp. nov. Both *P. madagascariensis* and *P. platei* were only known from the original descriptions and are described anatomically in detail for the first time. Amazingly, the best anatomical description of *P. peronii* so far is Cuvier’s (1804) original description, but many traits are described and illustrated here for the first time. Finally, the anatomy of all mitochondrial units of *P. verruculata* is described in detail for the first time from numerous localities, although some anatomical information was scattered in the literature for three of them (units #1, #3, and #4).

These nine species cannot be distinguished externally, except for the very large individuals of *P. peronii* (longer than 100 mm). However, details of the internal anatomy can help separate species, except for *P. platei* and *P. setoensis* which are both cryptic externally and internally. Geographic distribution varies greatly among *Peronia* species. Three species are broadly distributed across the Indo-West Pacific, from the western Indian Ocean to the West Pacific: *P. griffithsi*, *P. peronii*, and *P. verruculata*. The six other species are characterized by much narrower geographic ranges. Three species are even endemic: *Peronia okinawensis* and *P. setoensis* are endemic to Japan, and *P. willani* is endemic to the Northern Territory, Australia.

Of the 31 *Peronia* species names available, four are valid and 27 are invalid: 21 synonyms (16 of which are new), five *nomina dubia*, and one junior secondary homonym. The large number of available names in *Peronia* is explained by a combination of three main factors. First, *Peronia* slugs have often been collected, because they are common across the Indo-West Pacific and because they mostly live in the rocky intertidal, which is more easily accessible than mangrove forests where most other onchidiids are found. Second, earlier zoologists created new species names without examining the types of existing nominal species and without proper knowledge of individual variation, which resulted in many names being added unnecessarily. Third, *Peronia* is a genus for which molecular data were critically needed, because species are externally cryptic; also, species could hardly be delineated just based on their internal anatomy because they differ only with respect to minute anatomical details. The fact that five new species names are needed in *Peronia* even though there already are 31 available names shows that a comprehensive revision was desperately needed.

## Materials and methods

### Nomenclature

Establishing a complete list of available names for a taxon often requires an enormous amount of time but it is the keystone of any taxonomic revision, because otherwise it would be impossible to address the nomenclatural status of available names and to determine how many new species names are needed.

All available type specimens were re-examined beyond the taxon of interest (*Peronia*) because species names often are incorrectly classified when they are first created. For instance, *Onchidium durum* Labbé, 1934a was originally created for slugs with a smooth notum, but the types of *O. durum* clearly bear dorsal gills. Ignoring *O. durum* because it was created for slugs with a smooth notum would have led to an incomplete list of available *Peronia* species names. Several species names had to be transferred to *Peronia*, because they refer to slugs with dorsal gills, regardless of whether species were originally described with dorsal gills or not. When type specimens are not located, one needs to go through original species descriptions very carefully, and still beyond the taxon of interest. Reciprocally, not all species names ever classified in *Peronia* belong to *Peronia*: for instance, several specific names originally combined with *Peronia* refer to *Onchidella* species. Finally, many species names of doubtful application need to be commented upon.

In total, 51 species-group names had to be considered for the revision of *Peronia*. Of these, only 31 are available *Peronia* species names (Table 1). Indeed, ten of those 51 names are not classified in *Peronia*: eight names refer to *Onchidella* species, one to a *Wallaconchis* species, and one to a *Marmaronchis* species. And, ten other names are *nomina dubia* as they refer to species which may belong to any onchidiid genus or which may not even belong to an onchidiid genus.

**Table 1.** Alphabetic list of the 51 existing species-group names of which the nomenclatural status is addressed in the present work. Details can be found in the text: comments on the four valid *Peronia* species names, their synonyms, and the junior homonym are in the species remarks; comments on the fifteen *nomina dubia* and the ten names that must be classified in other genera are in the general discussion.

Species-group names	Type locality	Nomenclatural status
*Peronia acinosa* Gould, 1852	Fiji	*Nomen dubium* (onchidiid or not)
*Peronia alderi* JE Gray, 1850	Unknown	*Nomen dubium* (*Peronia*)
*Peronia anomala* Labbé, 1934a	Red Sea	New junior subjective synonym (*P. verruculata*, Red Sea)
*Onchidium astridae* Labbé, 1934b	West Papua	New junior subjective synonym (*P. verruculata*, unit #1)
*Onchidium ater* Lesson, 1831a	West Papua	* Wallaconchis *
*Onchidium branchiferum* Plate, 1893	Philippines	New junior subjective synonym (*P. verruculata*, unit #1)
*Scaphis carbonaria* Labbé, 1934a	New Caledonia	New junior subjective synonym (*P. verruculata*, unit #1)
*Onchidium celticum* Cuvier in Audouin and Milne-Edwards 1832	France	* Onchidella *
*Onchidium cinereum* Quoy & Gaimard, 1833	Tonga	*Nomen dubium* (*Wallaconchis*)
*Peronia corpulenta* Gould, 1852	Fiji	*Nomen dubium* (onchidiid or not)
*Onchidium durum* Labbé, 1934a	Red Sea	New junior subjective synonym (*P. verruculata*, Red Sea)
*Onchidium elberti* Simroth, 1920	Sulawesi	Junior subjective synonym (*P. verruculata*, unit #1)
*Onchidium ferrugineum* Lesson, 1831a	West Papua	Junior subjective synonym (*P. verruculata*, unit #1)
*Paraperonia fidjiensis* Labbé, 1934a	Fiji	New junior subjective synonym (*P. peronii*)
*Onchis fruticosa* Stimpson, 1855	Japan	*Nomen dubium* (*Peronia*)
*Peronia gaimardi* Labbé, 1934a	Vanikoro	New junior subjective synonym (*P. verruculata*, unit #1)
*Paraperonia gondwanae* Labbé, 1934a	Western India	New junior subjective synonym (*P. verruculata*, unit #4)
*Paraperonia gondwanae hombroni* Labbé, 1934a	Torres Strait	*Nomen dubium* (*Peronia*)
*Onchidium granulosum* Lesson, 1831b	New Ireland	*Nomen dubium* (onchidiid, *Peronia* or not)
*Scaphis gravieri* Labbé, 1934a	Mayotte	New junior subjective synonym (*P. verruculata*, unit #5)
*Onchidella griseofusca* Tapparone Canefri, 1874	Singapore	*Nomen dubium* (onchidiid, *Peronia* or not)
*Onchidium incisum* Quoy & Gaimard, 1832	Ascension Island	* Onchidella *
*Quoya indica* Labbé, 1934a	Indian Ocean	*Nomen dubium* (*Peronia*)
*Peronia indolens* Couthouy in Gould 1852	Brazil	* Onchidella *
*Peronia irrorata* Gould, 1852	New Zealand	* Onchidella *
*Paraperonia jousseaumei* Labbé, 1934a	Red Sea	New junior subjective synonym (*P. madagascariensis*)
*Peronia laevis* Blainville, 1826	West Papua	*Marmaronchis*: junior objective synonym of *Marmaronchis vaigiensis* (Quoy & Gaimard, 1825)
*Scaphis lata* Labbé, 1934a	Vietnam	New junior subjective synonym (*P. verruculata*, unit #1)
*Paraperonia madagascariensis* Labbé, 1934a	Madagascar	Valid (*Peronia madagascariensis*)
*Peronia mauritiana* Blainville, 1824	Mauritius	Junior objective synonym (*P. peronii*)
*Peronia marginata* Couthouy in Gould 1852	Tierra del Fuego	* Onchidella *
*Onchidium melanopneumon* Bergh, 1884	Fiji	Junior subjective synonym (*P. peronii*)
*Onchidium multiradiatum* Semper, 1882	Unknown	*Nomen dubium* (onchidiid, *Peronia* or not)
*Onchidium nebulosum* Semper, 1880	Palau	*Nomen dubium* (*Peronia*)
*Onchidium nigricans* Quoy & Gaimard, 1832	New Zealand	* Onchidella *
*Onchidium oniscoides* Blainville, 1816	Unknown	*Nomen dubium* (onchidiid, not *Peronia*)
*Peronia parthenopeia* Delle Chiaje, 1841	Sicily	* Onchidella *
*Onchidium patelloide* Quoy & Gaimard, 1832	New Zealand	* Onchidella *
*Onchidium peronii* Cuvier, 1804	Mauritius	Valid (*Peronia peronii*)
*Peronia persiae* Maniei et al., 2020a	Iran	New junior subjective synonym (*P. verruculata*, unit #4)
*Onchidium planatum* Quoy & Gaimard, 1825	Guam	*Nomen dubium* (onchidiid or not)
*Onchidium platei* Hoffmann, 1928	Tahiti	Valid (*Peronia platei*)
*Onchidium punctatum* Quoy & Gaimard, 1832	West Papua	New junior subjective synonym (*P. peronii*)
*Peronia savignii* Récluz, 1869	Red Sea	New junior objective synonym (*P. verruculata*, Red Sea)
*Onchidium savignyi* Semper, 1880	Philippines	Junior secondary homonym (*P. savignii*)
*Peronia semituberculata* Blainville, 1826	Guam	*Nomen dubium*, junior objective synonym of *Onchidium planatum* Quoy & Gaimard, 1825 (*nomen dubium*)
*Onchidium straelenii* Labbé, 1934b	Aru Islands	*Nomen dubium* (onchidiid, not *Peronia*)
*Onchidium tonganum* Quoy & Gaimard, 1832	Tonga	Junior subjective synonym (*P. peronii*)
*Scaphis tonkinensis* Labbé, 1934a	Vietnam	New junior subjective synonym (*P. verruculata*, unit #1)
*Onchidium verruculatum* Cuvier, 1830	Red Sea	Valid (*Peronia verruculata*)
*Scaphis viridis* Labbé, 1934a	Torres Strait	New junior subjective synonym (*P. verruculata*, unit #1)

### Incorrect subsequent spellings

Many subsequent incorrect spellings are encountered in the onchidiid literature. The subsequent incorrect spelling of a name is not available and, to our knowledge, no subsequent incorrect spelling is in prevailing usage (ICZN 1999: Article 33.3). Subsequent incorrect spellings of specific names are corrected throughout the present monograph. When a spelling mistake is quite big, it is pointed out, such as, for instance, when JE Gray (1850: 117) erroneously used *Peronia tongensis* instead of *Peronia tongana*, and when Mörch (1872b: 325) erroneously used *Peronia vermiculata* instead of *Peronia verruculata*. In addition, many *Peronia* species were originally classified in Buchannan’s (1800)*Onchidium*, for which some authors (e.g., Plate 1893; Hoffmann 1928; Labbé, 1934a) used the unjustified emendation *Oncidium*, which is systematically corrected as *Onchidium*.

### Museum collection abbreviations

**AM** Australian Museum, Sydney, New South Wales, Australia

**ANSP** Academy of Natural Sciences, Drexel University, Philadelphia, Pennsylvania, USA

**BNHS** Bombay Natural History Society, Mumbai, India

**BPBM** Bernice Pauahi Bishop Museum, Honolulu, Hawaii, USA

**CASIZ** California Academy of Sciences, San Francisco, California, USA

**ITBZC** Institute of Tropical Biology, Zoology Collection, Vietnam Academy of Science and Technology, Ho Chi Minh City, Vietnam

**MNHN** Muséum national d’Histoire naturelle, Paris, France

**MTQ** Museum of Tropical Queensland, Townsville, Queensland, Australia

**NHMD** Zoological Museum, Natural History Museum of Denmark, University of Copenhagen, Denmark

**NHMUK** Natural History Museum, London, United Kingdom

**NMSA** KwaZulu-Natal Museum, Pietermaritzburg, KwaZulu-Natal, South Africa

**NSMT** National Museum of Nature and Science, Tokyo, Japan

**NTM** Museum and Art Gallery Northern Territory, Darwin, Northern Territory, Australia

**PNM** National Museum of the Philippines, Manila, Philippines

**RBINS** Royal Belgian Institute of Natural Sciences, Brussels, Belgium

**SMF** Senckenberg Forschungsinstitut und Naturmuseum, Frankfurt am Main, Germany

**SMNH** Swedish Museum of Natural History, Stockholm, Sweden

**UF** University of Florida, Gainesville, USA

**UMIZ** Universitas Malikussaleh, North Aceh, Sumatra, Indonesia

**USMMC** Universiti Sains Malaysia, Mollusk Collection, Penang, Malaysia

**WAM** Western Australian Museum, Perth, Western Australia, Australia

**ZMB** Museum für Naturkunde, Berlin, Germany

**ZMH** Zoologisches Museum, Hamburg, Germany

**ZSM** Zoologische Staatssammlung München, Munich, Germany

**ZRC** Zoological Reference Collection, Lee Kong Chian Natural History Museum, National University of Singapore

### Collecting

Our molecular analyses and anatomical species descriptions are based on a data set of 179 individuals specifically gathered for the present study. Of those 179 individuals, 112 were collected by the Dayrat lab, of which 91 were deposited in countries or states of origin (Australia, Hawaii, India, Indonesia, Japan, Malaysia, Philippines, Singapore, Vietnam) and 21 (from Madagascar and Mauritius) were deposited at the MNHN. Of those 179 individuals, 36 were collected during MNHN expeditions organized by Philippe Bouchet (Madagascar, Mozambique, New Caledonia, Papua New Guinea, and Vanuatu) and are all preserved at the MNHN; the specimens from New Caledonia were collected by Adam Bourke. Of those 179 individuals, 12 were collected by several collaborators: Sadar Aslam collected three specimens from Pakistan deposited at the MNHN; Clay Carlson collected two specimens from Guam deposited at the CAS and the MNHN; Owen Griffiths collected one specimen from Mauritius deposited at the MNHN; Shau Hwai (Aileen) Tan collected two specimens from Malaysia deposited at the USM; and Tomoyuki Nakano collected four specimens from Japan deposited at the NSMT. And, finally, 19 were found in museum collections: four (AM), four (NHMUK), two (NMSA), and nine (UF).

Collecting expeditions of the Dayrat lab were led by Benoît Dayrat in the Andaman Islands (India), West Bengal (India), Peninsular Malaysia, the Philippines, Singapore, New South Wales (Australia), and Northern territory (Australia), by Tricia Goulding in Queensland (Australia), Mauritius, Madagascar, Vietnam, and western India, by Munawar Khalil in Indonesia, and by Rebecca Cumming in Japan. Sites were accessed by car or by boat. Although each site was explored for an average of two hours, the exact time spent at each site also depended on the time of the low tide, the weather conditions, etc. Photographs were taken to document the kind of habitat being visited as well as the diverse microhabitats where specimens were collected. Specimens were individually numbered and photographed in their respective habitat. At each site, as much diversity as possible was sampled: even specimens that looked similar were individually numbered so that the presence of cryptic diversity could be tested. Importantly, a piece of tissue was cut for all specimens individually numbered for DNA extraction and the remainder of each specimen was relaxed (using magnesium chloride) and fixed (using 10% formalin or 70% ethanol) for comparative anatomy.

### Specimens

**Specimens included in molecular analyses.** DNA extraction numbers unique to each individual are indicated in phylogenetic trees as well as in lists of material examined and in figure captions (numbers are between brackets). Our molecular data set includes 190 *Peronia* individuals, only eleven of which correspond to COI sequences obtained from GenBank or BOLD (Table 2). Anatomical descriptions are based on those 179 *Peronia* individuals for which sequences were generated for the present study as well as the available type material for existing names (see below).

All DNA sequences for the eleven outgroups are from our previous studies (Dayrat et al. 2016, 2017, 2018, 2019a, b, c, d; Dayrat and Goulding 2017; Goulding et al. 2018a, b, c), with the exception of the nuclear sequences for *Laspionchis boucheti*, which are new (Table 2). Most *Peronia* mitochondrial and nuclear sequences in our molecular data set are new (Table 2). New mitochondrial COI and 16S sequences are provided for 169 individuals, and COI and 16S sequences for ten specimens are from a previous study (Dayrat et al. 2011; see below). In addition, all COI sequences in GenBank and BOLD closely related to *Peronia* sequences in our dataset were examined, and COI sequences for eleven individuals were selected to be included in phylogenetic analyses (Table 2; see below). All nuclear 28S and ITS2 sequences are new except for two individuals which have been used as outgroups in several of our previous studies: [696-2] from Okinawa and [706] from Hawaii (Table 2).

**Table 2.** DNA extraction numbers and GenBank accession numbers for all the specimens included in the present study. The letter H next to an extraction number indicates the holotype. Sequences marked with an asterisk (*) are from our former publications (Dayrat et al. 2011, 2016, 2017, 2018, 2019a, b, c, d; Dayrat and Goulding 2017; Goulding et al. 2018a, b, c). In addition, 11 COI sequences also marked with an asterisk (*) were obtained from GenBank (GB) and BOLD: four sequences from China (Sun et al. 2014), two from Singapore (Chang et al. 2018), two from Japan (Takagi et al. 2019), one from the Persian Gulf (unpublished), one from Gujarat, western India (unpublished), and one from Iran (Maniei et al. 2020a). Abbreviations: Australian Museum, Sydney (AM); Bombay Natural History Society, India (BNHS); Bernice Pauahi Bishop Museum, Honolulu, Hawaii, USA (BPBM); California Academy of Sciences, San Francisco, California, USA (CASIZ); Institute of Tropical Biology, Zoology Collection, Vietnam Academy of Science and Technology (ITBZC); Muséum national d’Histoire naturelle, Paris, France (MNHN); Museum of Tropical Queensland, Townsville, Queensland, Australia (MTQ); Natural History Museum, London, United Kingdom (NHMUK); KwaZulu-Natal Museum, Pietermaritzburg, KwaZulu-Natal, South Africa (NMSA); National Museum of Nature and Science, Tokyo, Japan (NSMT); Museum and Art Gallery Northern Territory, Darwin, Northern Territory, Australia (NTM); National Museum of the Philippines, Manila (PNM); University of Florida, Gainesville, Florida, USA (UF); Universitas Malikussaleh, North Aceh, Sumatra, Indonesia (UMIZ); Universiti Sains Malaysia Mollusc Collection, Penang, Malaysia (USMMC); Zoological Reference Collection, Lee Kong Chian Natural History Museum, National University of Singapore (ZRC).

Species	Individual (DNA #)	Voucher	Locality	GenBank COI	GenBank 16S	GenBank ITS2	GenBank 28S
*Alionchis jailoloensis*	5137 H	UMIZ 00117	Indonesia, Halmahera	MG953528*	MG953538*	MG953548*	MK122918*
*Laspionchis boucheti*	1688 H	NTM P.57614	Australia, Northern Territory	MH619249*	MH619310*	MT652862	MT652995
*Marmaronchis vaigiensis*	1183	ZRC.MOL.3007	Singapore	MK122812*	MK122854*	MK122877*	MK122910*
*Melayonchis eloisae*	1011 H	ZRC.MOL.6499	Singapore	KX240026*	KX240050*	MK122904*	MK125515*
*Onchidella celtica*	5013	MNHN-IM-2019-1604	France	MG958715*	MG958717*	MK122906*	MK122921*
*Onchidina australis*	1523	AM C.468918.002	Australia, New South Wales	KX179548*	KX179561*	MG958719*	MG958887*
*Onchidium typhae*	965	USMMC 00005	Peninsular Malaysia	KX179509*	KX179525*	MG958720*	MG958885*
*Paromoionchis tumidus*	1732	UMIZ 00121	Indonesia, Sumatra	MH054951*	MH055104*	MH055196*	MH055268*
*Peronina tenera*	960	USMMC 00039	Peninsular Malaysia	MG958740*	MG958796*	MG958840*	MG958874*
*Platevindex luteus*	1001	ZRC.MOL.10179	Singapore	MG958714*	MG958716*	MG958718*	MG958888*
*Wallaconchis sinanui*	2740	UMIZ 00059	Indonesia, Ambon	MG970713*	MG970881*	MG971093*	MG971161*
***P. verruculata* (unit #1)**	1538	AM C.448363	Australia, Queensland (19°S)	MT653148	MT652693	MT652863	MT652996
	2571	MTQ	Australia, Queensland (16°S)	MT653149	MT652694	MT652864	
	2620	MTQ	Australia, Queensland (20°S)	MT653150	MT652695		
	2622	MTQ	Australia, Queensland (20°S)	MT653151	MT652696		
	2682	MTQ	Australia, Queensland (21°S)	MT653152	MT652697	MT652865	
	GB		China, Guangdong (21°N)	JN543152*			
	GB		China, Fujian (26°N)	JN543153*			
	GB		China, Guangxi (21°N)	JN543154*			
	GB		China, Hainan (18°N)	JN543165*			
	2724	UMIZ 00162	Indonesia, Ambon (03°S)	MT653153	MT652698	MT652866	MT652997
***P. verruculata* (unit #1)**	2729	UMIZ 00162	Indonesia, Ambon (03°S)	MT653154	MT652699		
	2856	UMIZ 00163	Indonesia, Ambon (03°S)	MT653155	MT652700	MT652867	
	3080	UMIZ 00164	Indonesia, Bali (08°S)	MT653156	MT652701	MT652868	
	3115	UMIZ 00165	Indonesia, Bali (08°S)	MT653157	MT652702	MT652869	
	5068	UMIZ 00166	Indonesia, Halmahera (00°S)	MT653158	MT652703		
	5120	UMIZ 00167	Indonesia, Halmahera (01°S)	MT653159	MT652704	MT652870	
	5124	UMIZ 00167	Indonesia, Halmahera (01°S)	MT653160	MT652705		
	5130	UMIZ 00167	Indonesia, Halmahera (01°S)	MT653161	MT652706		
	2987	UMIZ 00168	Indonesia, Lombok (08°S)	MT653162	MT652707	MT652871	
	2868	UMIZ 00169	Indonesia, Seram (02°S)	MT653163	MT652708		
	2870	UMIZ 00169	Indonesia, Seram (02°S)	MT653164	MT652709	MT652872	MT652998
	3441	UMIZ 00169	Indonesia, Seram (02°S)	MT653165	MT652710		
	731	NHMUK 20050628	Indonesia, Sulawesi	HQ660046*	HQ659914*		
	2127	UMIZ 00170	Indonesia, Sulawesi (01°N)	MT653166	MT652711		
	2150	UMIZ 00171	Indonesia, Sulawesi (01°N)	MT653167	MT652712		
	2162	UMIZ 00171	Indonesia, Sulawesi (01°N)	MT653168	MT652713	MT652873	
	1747	UMIZ 00172	Indonesia, Sumatra (05°S)	MT653169	MT652714	MT652874	
	1759	UMIZ 00173	Indonesia, Sumatra (05°S)	MT653170	MT652715	MT652875	MT652999
	5904	UMIZ 00174	Indonesia, Timor (10°S)	MT653171	MT652716	MT652876	
	5925	UMIZ 00175	Indonesia, Timor (10°S)	MT653172	MT652717	MT652877	
	5927	UMIZ 00175	Indonesia, Timor (10°S)	MT653173	MT652718		
	3751	NSMT-Mo 78988	Japan, Wakayama (33°N)	MT653174	MT652719	MT652878	
	3752	NSMT-Mo 78988	Japan, Wakayama (33°N)	MT653175	MT652720	MT652879	MT653000
	GB		Japan, Kagoshima (31°N)	LC390389*			
	6202	MNHN-IM-2019-1591	New Caledonia (22°S)	MT653176	MT652721	MT652880	MT653001
	6212	MNHN-IM-2019-1592	New Caledonia (22°S)	MT653177	MT652722	MT652881	MT653002
	6214	MNHN-IM-2019-1593	New Caledonia (21°S)	MT653178	MT652723	MT652882	
	698	UF 253871	Palau (07°N)	MT653179	MT652724	MT652883	MT653003
	5467	MNHN-IM-2013-12008	PNG, Madang (05°S)	MT653180	MT652725		
	5468	MNHN-IM-2013-12009	PNG, Madang (05°S)	MT653181	MT652726	MT652884	MT653004
	5469	MNHN-IM-2013-12010	PNG, Madang (05°S)	MT653182	MT652727		
	6085	MNHN-IM-2013-50974	PNG, New Ireland (02°S)	MT653183	MT652728	MT652885	MT653005
	6087	MNHN-IM-2013-53523	PNG, New Ireland (02°S)	MT653184	MT652729		
***P. verruculata* (unit #1)**	6088	MNHN-IM-2013-53525	PNG, New Ireland (02°S)	MT653185	MT652730		
	3379	PNM 041274	Philippines, Bohol (09°N)	MT653186	MT652731		
	3380	PNM 041274	Philippines, Bohol (09°N)	MT653187	MT652732	MT652886	
	3433	PNM 041276	Philippines, Bohol (09°N)	MT653188	MT652733		
	3437	PNM 041276	Philippines, Bohol (09°N)	MT653189	MT652734		
	712	UF 368518	Philippines, Cebu (09°N)	HQ660050*	HQ65991*		
	3160	PNM 041277	Philippines, Luzon (13°N)	MT653190	MT652735	MT652887	
	3161	PNM 041277	Philippines, Luzon (13°N)	MT653191	MT652736		
	704	UF 368517	Philippines, Negros (09°N)	MT653192	MT652737		
	991	ZRC.MOL.10497	Singapore (01°N)	MT653193	MT652738	MT652888	MT653006
	GB		Singapore	MH002570*			
	5480	MNHN-IM-2013-62392	Vanuatu (17°S)	MT653194	MT652739	MT652889	
	5481	MNHN-IM-2013-62393	Vanuatu (17°S)	MT653195	MT652740	MT652890	MT653007
	5620	ITBZC IM 00021	Vietnam (12°N)	MT653196	MT652741	MT652891	
	5621	ITBZC IM 00021	Vietnam (12°N)	MT653197	MT652742		
	5639	ITBZC IM 00023	Vietnam (08°N)	MT653198	MT652743	MT652892	
	5670	ITBZC IM 00022	Vietnam (08°N)	MT653199	MT652744	MT652893	
***P. verruculata* (unit #2)**	1072	BNHS 1072	India, Andaman (11°N)	MT653200	MT652745		
	1077	BNHS 119	India, Andaman (11°N)	MT653201	MT652746		
	1079	BNHS 120	India, Andaman (11°N)	MT653202	MT652747		
	1080	BNHS 121	India, Andaman (11°N)	MT653203	MT652748		
	1081	BNHS 122	India, Andaman (11°N)	MT653204	MT652749		
	1084	BNHS 117	India, Andaman (11°N)	MT653205	MT652750		
	1741	UMIZ 00179	Indonesia, Sumatra (05°S)	MT653206	MT652751	MT652894	
	1742	UMIZ 00179	Indonesia, Sumatra (05°S)	MT653207	MT652752	MT652895	
	1746	UMIZ 00178	Indonesia, Sumatra (05°S)	MT653208	MT652753	MT652896	MT653008
	1795	UMIZ 00180	Indonesia, Sumatra (05°S)	MT653209	MT652754	MT652897	
	1796	UMIZ 00180	Indonesia, Sumatra (05°S)	MT653210	MT652755	MT652898	
	1797	UMIZ 00180	Indonesia, Sumatra (05°S)	MT653211	MT652756	MT652899	MT653009
***P. verruculata* (unit #3)**	974	USMMC 00064	Peninsular Malaysia (06°N)	MT653212	MT652757	MT652900	MT653010
	975	USMMC 00064	Peninsular Malaysia (06°N)	MT653213	MT652758	MT652901	
	976	USMMC 00051	Peninsular Malaysia (06°N)	MT653214	MT652759	MT652902	
	977	USMMC 00064	Peninsular Malaysia (06°N)	MT653215	MT652760	MT652903	
	2546	USMMC 00065	Peninsular Malaysia (05°N)	MT653216	MT652761	MT652904	MT653011
	2547	USMMC 00065	Peninsular Malaysia (05°N)	MT653217	MT652762	MT652905	MT653012
	989	ZRC.MOL.16070	Singapore (01°N)	MT653218	MT652763	MT652906	MT653013
***P. verruculata* (unit #3)**	990	ZRC.MOL.10496	Singapore (01°N)	MT653219	MT652764	MT652907	MT653014
	GB		Singapore	MH002601*			
***P. verruculata* (unit #4)**	1141	BNHS 22	India, western coast (19°N)	MT653220	MT652765		
	1143	BNHS 24	India, western coast (19°N)	MT653221	MT652766		
	1144	BNHS 23	India, western coast (19°N)	MT653222	MT652767		
	6164	MNHN-IM-2019-1384	Pakistan (24°N)	MT653223	MT652768	MT652908	MT653015
	6165	MNHN-IM-2019-1385	Pakistan (24°N)	MT653224	MT652769	MT652909	MT653016
	6166	MNHN-IM-2019-1386	Pakistan (24°N)	MT653225	MT652770	MT652910	MT653017
	GB		Iran (26°N)	MK993404*	MK993392*		
***P. verruculata* (unit #5)**	3140	MNHN-IM-2019-1610	Madagascar (12°S)	MT653226	MT652771	MT652911	MT653018
	3142	MNHN-IM-2019-1610	Madagascar (12°S)	MT653227	MT652772	MT652912	
	3143	MNHN-IM-2019-1611	Madagascar (12°S)	MT653228	MT652773	MT652913	
	3144	MNHN-IM-2019-1611	Madagascar (12°S)	MT653229	MT652774	MT652914	
	3146	MNHN-IM-2019-1611	Madagascar (12°S)	MT653230	MT652775	MT652915	MT653019
	3149	MNHN-IM-2019-1611	Madagascar (12°S)	MT653231	MT652776	MT652916	
	3231	MNHN-IM-2019-1610	Madagascar (12°S)	MT653232	MT652777	MT652917	
	3597	MNHN-IM-2019-1610	Madagascar (12°S)	MT653233	MT652778	MT652918	
	3598	MNHN-IM-2019-1610	Madagascar (12°S)	MT653234	MT652779	MT652919	
	3600	MNHN-IM-2019-1611	Madagascar (12°S)	MT653235	MT652780	MT652920	MT653020
	730	NHMUK 20080190	Mozambique (12°S)	HQ660045*	HQ659913*		
	733	NHMUK 20060257	Mozambique (11°S)	HQ660047*	HQ659915*		
	5507	MNHN-IM-2013-62395	Mozambique (26°S)	MT653236	MT652781	MT652920	MT653021
	5510	MNHN-IM-2013-62398	Mozambique (26°S)	MT653237	MT652782	MT652920	MT653022
***P. griffithsi***	2934	UMIZ 00177	Indonesia, Kei (05°S)	MT653238	MT652783	MT652923	MT653023
	2936	UMIZ 00176	Indonesia, Kei (05°S)	MT653239	MT652784	MT652924	MT653024
	3566	UMIZ 00177	Indonesia, Kei (05°S)	MT653240	MT652785	MT652925	
	3153	MNHN-IM-2019-1608	Mauritius (20°S)	MT653241	MT652786	MT652926	MT653025
	3154	MNHN-IM-2019-1608	Mauritius (20°S)	MT653242	MT652787	MT652927	MT653026
	3155	MNHN-IM-2019-1608	Mauritius (20°S)	MT653243	MT652788		
	3156	MNHN-IM-2019-1608	Mauritius (20°S)	MT653244	MT652789	MT652928	MT653027
	3157 H	MNHN-IM-2000-35265	Mauritius (20°S)	MT653245	MT652790	MT652929	MT653028
	3606	MNHN-IM-2019-1608	Mauritius (20°S)	MT653246	MT652791	MT652930	
	3607	MNHN-IM-2019-1608	Mauritius (20°S)	MT653247	MT652792		
	3608	MNHN-IM-2019-1608	Mauritius (20°S)	MT653248	MT652793	MT652931	
	6095	MNHN-IM-2013-53535	PNG, New Ireland (02°S)	MT653249	MT652794	MT652932	MT653029
***P. madagascariensis***	BOLD		India, Gujarat, Dwarka (22°N)	LGEN099-14*			
	GB		Iran, Persian Gulf	LC027608*			
	5500	MNHN-IM-2009-16391	Madagascar (25°S)	MT653250	MT652795	MT652933	
	5501	MNHN-IM-2009-16392	Madagascar (25°S)	MT653251	MT652796	MT652934	MT653030
	5502	MNHN-IM-2009-16393	Madagascar (25°S)	MT653252	MT652797	MT652935	
	5503	MNHN-IM-2009-16396	Madagascar (25°S)	MT653253	MT652798	MT652936	
	5504	MNHN-IM-2009-16412	Madagascar (25°S)	MT653254	MT652799		
	5506	MNHN-IM-2009-16418	Madagascar (25°S)	MT653255	MT652800	MT652937	MT653031
	735	NHMUK 20060414	Mozambique (12°S)	HQ660042*	HQ659910*	MT652938	MT653032
	703	UF 332088	Oman (23°N)	MT653256	HQ659912*	MT652939	MT653033
	5841	NMSA W7547	South Africa (29°S)	MT653257	MT652801	MT652940	MT653034
	5842	NMSA W7547	South Africa (29°S)	MT653258	MT652802	MT652941	MT653035
***P. okinawensis***	696-2	UF 352288	Japan, Okinawa (26°N)	HQ660043*	HQ659911*	MG958871*	MG958883*
	696-3	UF 352288	Japan, Okinawa (26°N)	MT653259	MT652803	MT652942	MT653036
	696-4 H	UF 352288	Japan, Okinawa (26°N)	MT653260	MT652804	MT652943	MT653037
***P. peronii***	443	CASIZ 180486	Guam (13°N)	HQ660041*	HQ659909*	MT652944	MT653038
	5840	MNHN-IM-2019-1609	Guam (13°N)	MT653261	MT652805	MT652945	MT653039
	GB		Japan, Okinawa	LC390402*			
	1553	MNHN-IM-2019-1607	Mauritius (20°S)	MT653262	MT652806	MT652946	MT653040
	3605	MNHN-IM-2019-1606	Mauritius (20°S)	MT653263	MT652807	MT652947	MT653041
	5872	MNHN-IM-2019-1605	Mauritius (20°S)	MT653264	MT652808	MT652948	
	5874	MNHN-IM-2019-1605	Mauritius (20°S)	MT653265	MT652809	MT652949	
	5471	MNHN-IM-2013-12500	PNG, Madang (05°S)	MT653266	MT652810	MT652950	MT653042
	5472	MNHN-IM-2013-14052	PNG, Madang (05°S)	MT653267	MT652811	MT652951	MT653043
	5474	MNHN-IM-2013-14054	PNG, Madang (05°S)	MT653268	MT652812	MT652952	
	5476	MNHN-IM-2013-16260	PNG, Madang (05°S)	MT653269	MT652813	MT652953	MT653044
	5477	MNHN-IM-2013-15872	PNG, Madang (05°S)	MT653270	MT652814	MT652954	MT653045
	6086	MNHN-IM-2013-53482	PNG, New Ireland (02°S)	MT653271	MT652815	MT652955	MT653046
***P. platei***	5405	MNHN-IM-2013-13762	PNG, Madang (05°S)	MT653272	MT652816		
	5410	MNHN-IM-2013-15765	PNG, Madang (05°S)	MT653273	MT652817	MT652956	
	5412	MNHN-IM-2013-13351	PNG, Madang (05°S)	MT653274	MT652818	MT652957	MT653047
	5464	MNHN-IM-2013-15871	PNG, Madang (05°S)	MT653275	MT652819	MT652958	MT653048
	706	UF 303653	USA, Hawaii (21°N)	HQ660038*	HQ659906*	MG958722*	MG958884*
	5380	UF 303653	USA, Hawaii (21°N)	MT653276	MT652820	MT652959	MT653049
	6160	BPBM 284527	USA, Hawaii (21°N)	MT653277	MT652821	MT652960	MT653050
	6161	BPBM 284528	USA, Hawaii (21°N)	MT653278	MT652822	MT652961	MT653051
***P. setoensis***	3753	NSMT-Mo 78987	Japan, Wakayama (33°N)	MT653279	MT652823	MT652962	MT653052
	3754	NSMT-Mo 78987	Japan, Wakayama (33°N)	MT653280	MT652824	MT652963	MT653053
	5382	NSMT-Mo 78986	Japan, Wakayama (33°N)	MT653281	MT652825	MT652964	MT653054
	5383 H	NSMT-Mo 78985	Japan, Wakayama (33°N)	MT653282	MT652826	MT652965	MT653055
	5384	NSMT-Mo 78986	Japan, Wakayama (33°N)	MT653283	MT652827	MT652966	MT653056
	5385	NSMT-Mo 78986	Japan, Wakayama (33°N)	MT653284	MT652828	MT652967	
***P. sydneyensis***	1513	AM C.468912.004	New South Wales (33°S)	MT653285	MT652829	MT652968	MT653057
	1516 H	AM C.468916.001	New South Wales (33°S)	MT653286	MT652830	MT652969	MT653058
	1517	AM C.468915.001	New South Wales (33°S)	MT653287	MT652831	MT652970	MT653059
	734	AM C.459511	Queensland (22°S)	HQ660048*	HQ659916*		
	1539	AM C.459510	Queensland (22°S)	MT653288	MT652832		
	1540	AM C.459511	Queensland (22°S)	MT653289	MT652833	MT652971	MT653060
	2646	MTQ	Queensland (20°S)	MT653290	MT652834	MT652972	
	2653	MTQ	Queensland (20°S)	MT653291	MT652835	MT652973	
	2656	MTQ	Queensland (20°S)	MT653292	MT652836	MT652974	
	2661	MTQ	Queensland (20°S)	MT653293	MT652837		
	2662	MTQ	Queensland (20°S)	MT653294	MT652838	MT652975	MT653061
	2664	MTQ	Queensland (20°S)	MT653295	MT652839	MT652976	
***P. sydneyensis***	2667	MTQ	Queensland (20°S)	MT653296	MT652840		
	2680	MTQ	Queensland (21°S)	MT653297	MT652841		
	6189	MNHN-IM-2019-1594	New Caledonia (22°S)	MT653298	MT652842	MT652977	
	6195	MNHN-IM-2019-1595	New Caledonia (22°S)	MT653299	MT652843	MT652978	MT653062
	6209	MNHN-IM-2019-1596	New Caledonia (22°S)	MT653300	MT652844	MT652979	
	6213	MNHN-IM-2019-1597	New Caledonia (21°S)	MT653301	MT652845	MT652980	MT653063
	6220	MNHN-IM-2019-1598	New Caledonia (21°S)	MT653302	MT652846	MT652981	MT653064
	6222	MNHN-IM-2019-1599	New Caledonia (21°S)	MT653303	MT652847	MT652982	
***P. willani***	1620	NTM P.57626	Northern Territory (12°S)	MT653304	MT652848	MT652983	MT653065
	1623	NTM P.57627	Northern Territory (12°S)	MT653305	MT652849	MT652984	MT653066
	1624	NTM P.57627	Northern Territory (12°S)	MT653306	MT652850	MT652985	
	1625	NTM P.57627	Northern Territory (12°S)	MT653307	MT652851	MT652986	
	1626	NTM P.57627	Northern Territory (12°S)	MT653308	MT652852	MT652987	MT653067
	1628 H	NTM P.57625	Northern Territory (12°S)	MT653309	MT652853	MT652988	MT653068
	1629	NTM P.57627	Northern Territory (12°S)	MT653310	MT652854		
	1653	NTM P.57626	Northern Territory (12°S)	MT653311	MT652855	MT652989	
	1654	NTM P.57626	Northern Territory (12°S)	MT653312	MT652856	MT652990	
	1655	NTM P.57626	Northern Territory (12°S)	MT653313	MT652857	MT652991	MT653069
	1667	NTM P.57627	Northern Territory (12°S)	MT653314	MT652858	MT652992	MT653070
	1668	NTM P.57627	Northern Territory (12°S)	MT653315	MT652859	MT652993	
	1669	NTM P.57627	Northern Territory (12°S)	MT653316	MT652860		
	1670	NTM P.57627	Northern Territory (12°S)	MT653317	MT652861		

**COI sequences publicly available.** Eleven COI sequences obtained from GenBank (10) and BOLD (1) were added to our own data set (179 COI sequences) for a total of 190 sequences. Four COI sequences are from China (Sun et al. 2014), two from Singapore (Chang et al. 2018), two from Japan (Takagi et al. 2019), one from the Persian Gulf (unpublished), one from Gujarat, western India (unpublished), and one from Iran (Maniei et al. 2020a). All those sequences were merely referred to as *Peronia* sp., except for the specimens from China (referred to as *Peronia verruculata*), the specimen from western India (referred to as *Onchidium verruculatum*), and the specimen from Iran (recently described as *P. persiae*, a name regarded here as a synonym of *P. verruculata*, unit #4). Correct identifications are provided here for all those sequences (Table 2). Note that in the case of duplicate sequences available in GenBank, only one representative was selected. So, for instance, Chang et al. (2018) published many *Peronia*COI sequences that cluster in two mitochondrial units which they refer to as “Singapore clade” and “*Peronia* sp. 2 clade.” One sequence for their “Singapore clade” and one sequence for their “*Peronia* sp. 2 clade” are included here, which is enough to demonstrate that their two units correspond to our two mitochondrial units #1 and #3 of *Peronia verruculata*. Also, all COI sequences by Maniei et al. (2020a) for *P. persiae* cluster together within the unit #4 of *P. verruculata*, so only one of those individuals is included in our analyses: one individual is enough to demonstrate that *P. persiae* is a junior synonym of *P. verruculata*. A 16S sequence is available for the individual from Iran (Maniei et al. 2020a); no 16S sequences are available for any of the other COI sequences from GenBank and BOLD, so gaps were inserted in the mitochondrial concatenated alignment.

**Vouchers used in Dayrat et al. (2011).** Ten of our *Peronia* specimens were tentatively identified by Dayrat et al. (2011) at a time when nothing was known about the onchidiid species diversity in general and most especially in the genus *Peronia*. Most of those ten specimens were merely referred to with numbers (e.g., *Peronia* sp. 1). In order to avoid any confusion, those specimens are all included here so that correct species names are provided (Table 2). The specimen [443] (CASIZ 180486) identified as *Peronia peronii* from Guam really belongs to *P. peronii*. The specimen [696-2] (UF 352288) identified as Peronia cf. verruculata from Okinawa belongs to the new species *P. okinawensis*. The specimen [706] (UF 303653) identified as *Peronia* sp. 1 from Hawaii belongs to *P. platei*. The specimen [734] (AM C.459511) identified as *Peronia* sp. 3 from Queensland, Australia, belongs to the new species *P. sydneyensis*. Two specimens belong to *P. madagascariensis*: [735] (NHMUK 20060414) identified as Peronia cf. peronii from Mozambique, and [703] (UF 332088) identified as *Peronia* sp. 2 from Oman. Four specimens belong to *P. verruculata*: [712] (UF 368518) identified as *Scaphis* sp. from Cebu, Philippines, [730] (NHMUK 20080190) identified as *Peronia* sp. 4 from Mozambique, [733] (NHMUK 20060257) identified as *Peronia* sp. 5 from Mozambique, and [731] (NHMUK 20050628) identified as *Peronia* sp. 6 from Sulawesi, Indonesia.

**Types of existing species-group names.** All type specimens available for all onchidiid species-group names have been examined in context of the revision of the entire family. Comments on many onchidiid types can be found in our previous revisions (Dayrat et al. 2016, 2017, 2018, 2019a, b, c, d; Dayrat and Goulding 2017; Goulding et al. 2018a, b, c). In total, 118 type specimens (holotypes, lectotypes, paralectotypes, syntypes, etc.) are commented on here for the first time. Fifteen of those 118 type specimens are commented on in the general discussion because they are types of *nomina dubia* which may or may not refer to *Peronia* slugs. All the other (103) types are commented on in species descriptions because they are the types of 25 species-group names which must be classified in *Peronia* and which are not *nomina dubia* (Table 1).There are only two *Peronia* species names for which types could not be located: *Scaphis lata* Labbé, 1934a, and *Paraperonia jousseaumei* Labbé, 1934a. Finally, 14 lectotypes are designated here in order to clarify the application of 14 species names, usually because syntypes belong to different species or come from very distant localities.

Many type specimens were not labeled as types and were found within the general collections. In most cases, it was easy to determine that specimens were types because the information on the labels would match perfectly to that of the original descriptions. However, finding Labbé’s types was challenging, with the exception of the holotype, by monotypy, of *Onchidium astridae* Labbé, 1934b, preserved in Bruxelles (RBINS I.G.9223/MT.3822): it was not marked as a holotype, but the name *Onchidium astridae* is on the label, and the locality and collector information is matching.

The types of all the other *Peronia* species (and one subspecies) described by Labbé are preserved at the MNHN (the monograph in which those new taxa were described was almost exclusively based on material from the MNHN). The major issue with this material is that Labbé did not write any of his new species names on any of the labels. To be fair and fully accurate, there are actually three jars for which a specific name was written in pencil and in tiny letters on labels: one jar contains the type material of *Onchidium durum* (MNHN-IM-2000-33698), and two other jars contain part of the type material of *Paraperonia gondwanae* (MNHN-IM-2000-33683, MNHN-IM-2000-33688). Eleven years ago, Dayrat (2009) considered that identifying the types of Labbé’s onchidiid species names in the MNHN collection would be too risky (because specimens could be erroneously interpreted as types). However, after Virginie Héros (who is in charge of the Mollusk type collection at the MNHN) correctly remarked that it should still be possible to find some of Labbé’s types, an excel file was generated including all the old onchidiid material preserved at the MNHN and all the material cited in Labbé’s (1934a) monograph. By comparing various information (localities, names of the collectors, collecting dates, specimen sizes), it then became clear that many specimens could be identified as types with great confidence, even though they were not labeled as types and Labbé’s species names were not indicated on the labels.

For instance, originally, no jar clearly labeled as the type material of *Scaphis carbonaria* was found at the MNHN. However, of the old jars found at the MNHN with specimens from New Caledonia, only one matches perfectly the information provided in Labbé’s original description of *S. carbonaria*: an individual collected in 1880 by Réveillère (with an identification as *Peronia*). Other jars with one or more specimens from New Caledonia were collected by Fisher in 1878 and by François in 1894. Therefore, it is extremely likely that the specimen collected by Réveillère in 1880 is the holotype, by monotypy, of *Scaphis carbonaria* (MNHN-IM-2000-33708). In many cases, however, identifying the types happened to be much more challenging because there were several jars with the same locality, the same collector, and the same collecting date. In order to avoid any future confusion, Labbé’s types are commented on in great detail in species descriptions. There are only two of Labbé’s species for which no type material could be confidently traced back at the MNHN: *Scaphis lata* Labbé, 1934a, and *Paraperonia jousseaumei* Labbé, 1934a.

Finally, the type material of *Peronia persiae*, recently described by Maniei et al. (2020a), was not borrowed for examination. Regardless, there is no doubt that *P. persiae* is a junior synonym of both *P. verruculata* (Cuvier, 1830) and *P. gondwanae* (Labbé, 1934a) (Table 1), because all the COI and 16S sequences published for *P. persiae* cluster within the mitochondrial unit #4 of *P. verruculata*.

**Additional material examined (historical museum collections).** In addition to the 189 specimens included in the molecular analyses (not including the eleven outgroups) and the 118 type specimens of existing nominal species, 297 old specimens were obtained from museum collections from which no DNA could be extracted. Those specimens correspond to a total of 60 jars. One jar contains 161 specimens. All other jars contain fewer than 15 specimens. These old museum specimens are not included in the anatomical species descriptions, except for the description of *Peronia verruculata* from the Red Sea. Instead, these additional specimens are commented on in the species remarks. The additional specimens were especially useful to provide geographic records from places which could not be visited, such as the Chagos Archipelago, Nicobar Islands, Persian Gulf, and Socotra. Identifying *Peronia* species using only anatomical traits is challenging but possible (see below). Finally, some of the historical specimens from museum collections were studied by previous authors, and their re-examination allowed us to confirm or reject many identifications from the literature.

### Anatomical preparations and descriptions

Size (length/width) is indicated in millimeters (mm) for each specimen. Both the external morphology and the internal anatomy were studied. All anatomical observations were made under a dissecting microscope and drawn with a camera lucida. Radulae and male reproductive organs were prepared for scanning electron microscopy (Zeiss SIGMA Field Emission Scanning Electron Microscopy). Radulae were cleaned in 10% NaOH for a week, rinsed in distilled water, briefly cleaned in an ultrasonic water bath (less than a minute), sputter-coated with gold-palladium and examined by SEM. Soft parts (penis, accessory penial gland, etc.) were dehydrated in ethanol and critical point dried before coating.

Anatomical species descriptions are based on those 179 *Peronia* individuals for which sequences were generated for the present study as well as on the available type material for species with existing names (see below). To avoid unnecessary repetition, the description of anatomical features that are virtually identical between *Peronia* species (e.g., nervous system, heart, and stomach) is not repeated for each species. However, all the characters that are useful for species comparison (e.g., intestinal loops and male apparatus) are described for every species. Special attention has been given to illustrating the holotype and the type locality of each new species.

Species are being described following a phylogenetic order. The detailed description of *Peronia verruculata* is based on the mitochondrial unit #1, by far the most widespread (from Peninsular Malaysia to the West Pacific) and most abundant (55 specimens in our study), but variations in the other units are precisely reported and figure captions indicate the unit to which each illustrated individual belongs.

### Types of intestinal loops

**Figure 1.**
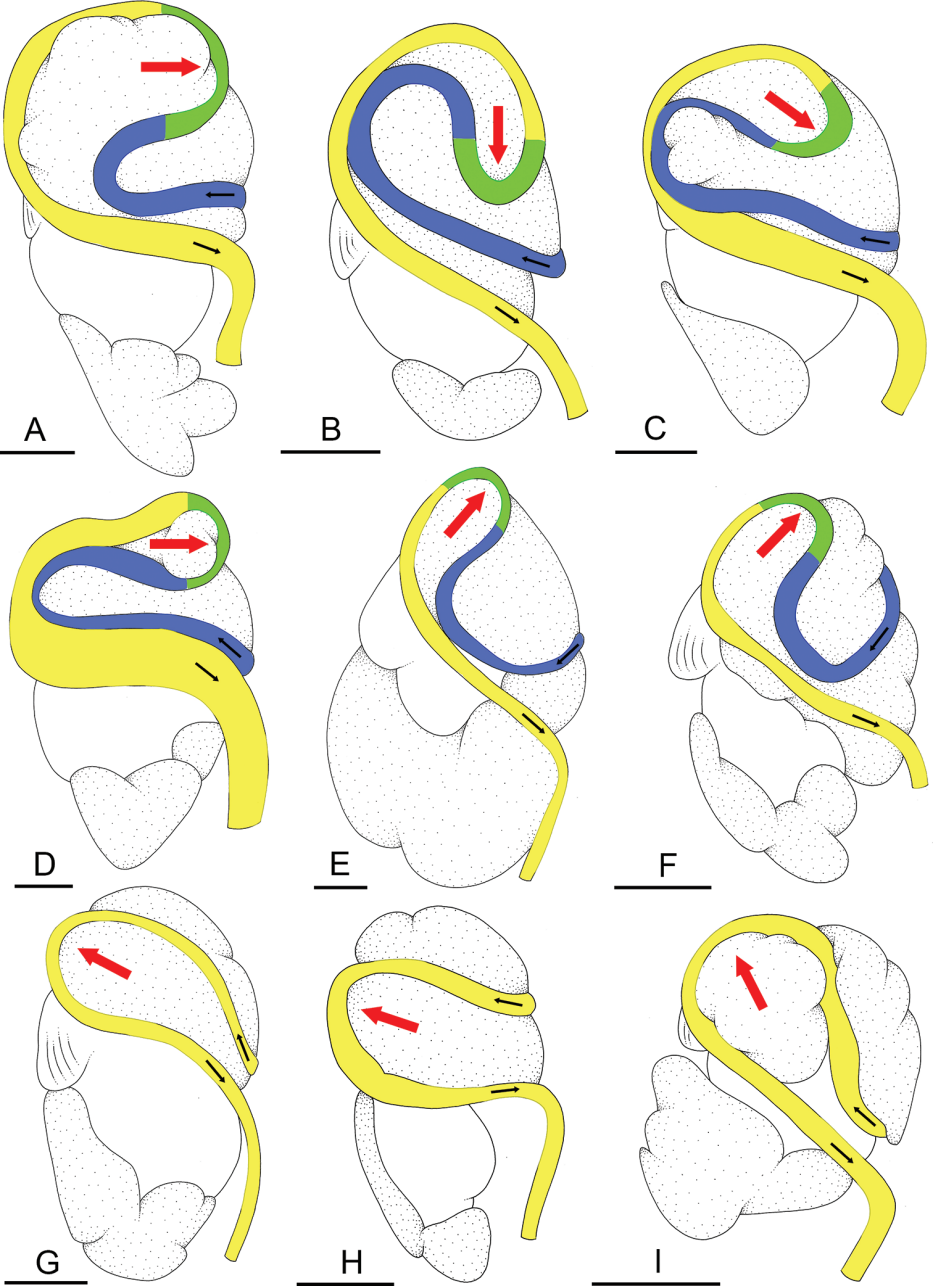
Intestinal types found in *Peronia* species. A clockwise intestinal loop is colored in blue, a counterclockwise intestinal loop is colored in yellow, and a transitional loop between them is colored in green. The big red arrow indicates the orientation of the transitional loop (**A–F**) or counterclockwise loop (**G–I**), and the small black arrows indicates the direction of the intestinal transport **A** type I, with a transitional loop oriented at 3 o’clock, *P. sydneyensis*, Australia, New South Wales, [1517] (AM C.468915.001) **B** type I, with a transitional loop oriented at 6 o’clock, *P. verruculata*, lectotype of *P. anomala*, Red Sea (MNHN-IM-2000-33678) **C** type I, with a transitional loop oriented between 4 and 5 o’clock, *P. verruculata*, lectotype, Red Sea (MNHN-IM-2000-22941) **D** type I, with a transitional loop oriented at 3 o’clock, *P. peronii*, paralectotype of *Onchidium peronii*, Timor (MNHN-IM-2000- 22938) **E** type I, with a transitional loop oriented at 1 o’clock, *P. peronii*, lectotype of *Paraperonia fidjiensis*, Fiji (MNHN-IM-2000-33692) **F** type I, with a transitional loop oriented between 1 and 2 o’clock, *P. okinawensis*, holotype, Japan, Okinawa, [696-4 H] (UF 352288) **G** type V, with a counterclockwise loop oriented between 10 and 11 o’clock, *P. madagascariensis*, Madagascar, [5501] (MNHN-IM-2009-16392) **H** type V, with a counterclockwise loop oriented at 10 o’clock, *P. platei*, lectotype, French Polynesia (SMNH-Type-7537) **I** type V, with a counterclockwise loop oriented at 11 o’clock, *P. griffithsi*, holotype, Mauritius, [3157 H] (MNHN-IM-2000-35265). Scale bars: 2 mm (**A, B**), 5 mm (**C–G**), 3 mm (**H, I**).

In onchidiids, types of intestinal loops are defined based on the pattern of the intestine on the dorsal aspect of the digestive gland (with the digestive gland still in place). Plate (1893) first distinguished four types of intestinal loops (types I to IV) and Labbé (1934a) later added a type V. Only the types I and V are found in *Peronia*. Hoffmann (1928: 51, pl. 3, fig. 11) noted before Labbé that intestinal loops of type V differ from other types and he referred to them as type Ia. Labbé’s terminology (type V) is preferred because past authors have adopted it and because a type V is very different from a type I. The different types of intestinal loops and their individual variation are best revealed by coloring sections of the intestine differently (Dayrat et al. 2019b, c, d): a clockwise intestinal loop is colored in blue, a counterclockwise intestinal loop is colored in yellow, and a transitional loop between them is colored in green (Fig. 1).

The intestine first appears dorsally on the right side. In intestinal loops of type I, the intestine starts by forming a clockwise (blue) loop which does not make a complete circle. As a result, the transitional (green) loop is oriented to the right (Fig. 1A–F). In two species with intestinal loops of type I (*P. okinawensis* and *P. peronii*), the transitional loop is oriented between 12 and 3 o’clock (Fig. 1D–F). In the three other species with intestinal loops of type I (*P. sydneyensis*, *P. verruculata*, and *P. willani*), the transitional loop is oriented between 3 and 6 o’clock (Fig. 1A–C). In intestinal loops of type V, the intestine starts by forming immediately a counterclockwise (yellow) loop. In intestinal loops of type V, the counterclockwise loop is oriented between 10 and 11 o’clock (Fig. 1G–I). Four *Peronia* species are characterized by intestinal loops of type V: *P. griffithsi*, *P. madagascariensis*, *P. platei*, and *P. setoensis*.

### DNA extraction and PCR amplification

DNA was extracted using a phenol-chloroform extraction protocol with cetyltrimethyl-ammonium bromide (CTAB). The mitochondrial cytochrome *c* oxidase I region (COI) and 16S region were amplified using the following universal primers (all 5’-3’): LCO1490 GGT CAA CAA ATC ATA AAG ATA TTG G, and HCO2198 TAA ACT TCA GGG TGA CCA AAR AAY CA (Folmer et al. 1994), 16Sar-L CGC CTG TTT ATC AAA AAC AT (Palumbi 1996), and the modified Palumbi primer 16S 972R CCG GTC TGA ACT CAG ATC ATG T (Dayrat et al. 2011). The nuclear ITS2 and 28S regions were amplified with the following primers: LSU-1 CTA GCT GCG AGA ATT AAT GTG A, and LSU-3 ACT TTC CCT CAC GGT ACT TG (Wade and Mordan 2000), 28SC1 ACC CGC TGA ATT TAA GCA T (Hassouna et al. 1984), and 28SD3 GAC GAT CGA TTT GCA CGT CA (Vonnemann et al. 2005). The 25 μl PCRs for COI and 16S contained 15.8 μl of water, 2.5 μl of 10× PCR Buffer, 1.5 μl of 25 mM MgCl_2_, 0.5 μl of each 10 μM primer, 2 μl of dNTP Mixture, 0.2 μl (1 unit) of TaKaRa Taq (Code No. R001A), 1 μl of 20 ng/μl template DNA, and 1 μl of 100× BSA (Bovine Serum Albumin). The PCRs for ITS2 used the reagents in the same amounts as COI and 16S, except that water was reduced to 14.8 μl and the amount of 100× BSA was increased to 2 μl. The PCRs for 28S included 14.8 μl of water, 2.5 μl of 10× PCR Buffer, 0.5 μl of each 10 μM primer, 1 μl of dNTP Mixture, 5 μl of Q solution (which includes MgCl_2_) and 0.5 μl of 20 ng/μl template DNA. The thermoprofile used for COI and 16S was: 5 minutes at 94 °C; 30 cycles of 40 seconds at 94 °C, 1 minute at 46 °C, and 1 minute at 72 °C; and a final extension of 10 minutes at 72 °C. The ITS2 thermoprofile was: 1 minute at 96 °C; 35 cycles of 30 seconds at 94 °C, 30 seconds at 50 °C, and 1 minute at 72 °C; and a final extension of 10 minutes at 72 °C. The 28S thermoprofile was: 4 minutes at 94 °C; 38 cycles of 50 seconds at 94 °C, 1 minute at 52 °C, and 2 minutes 30 seconds at 72 °C; and a final extension of 10 minutes at 72 °C. The PCR products were cleaned with ExoSAP-IT (Affymetrix, Santa Clara, CA, USA) prior to sequencing. Untrimmed sequenced fragments represented approximately 680 bp for COI, 530 bp for 16S, 740 bp for ITS2, and 1030 bp for 28S.

### Phylogenetic analyses

Chromatograms were consulted to resolve rare ambiguous base calls. DNA sequences were aligned using Clustal W in MEGA 7 (Kumar et al. 2016). Eleven onchidiid species outside *Peronia* were selected as outgroups from our previous studies (Dayrat et al. 2011, 2016, 2017, 2018, 2019a, b, c, d; Dayrat and Goulding 2017; Goulding et al. 2018a, b, c): *Alionchis jailoloensis* Goulding & Dayrat in Goulding et al. 2018a, *Laspionchis boucheti* Dayrat & Goulding in Dayrat et al. 2019b; *Marmaronchis vaigiensis* (Quoy & Gaimard, 1825), *Melayonchis eloisae* Dayrat in Dayrat et al. 2017, *Onchidella celtica* (Cuvier in Audouin and Milne-Edwards 1832), *Onchidina australis* (Semper, 1880), *Onchidium typhae* Buchannan, 1800, *Paromoionchis tumidus* (Semper, 1880), *Peronina tenera* (Stoliczka, 1869), *Platevindex luteus* (Semper, 1880), and *Wallaconchis sinanui* Goulding & Dayrat in Goulding et al. 2018b. All new DNA sequences were deposited in GenBank and vouchers deposited in museum collections (Table 2). The ends of each alignment were trimmed. Alignments of mitochondrial (COI and 16S) sequences and nuclear (ITS2 and 28S) sequences were concatenated separately in order to test whether these two data sets support the same relationships. The concatenated mitochondrial alignment included 1014 nucleotide positions: 614 (COI) and 400 (16S). The concatenated ITS2 and 28S alignment included 1544 nucleotide positions: 535 (ITS2) and 1009 (28S). The haplotype ITS2 alignment (in which identical sequences were grouped into a single haplotype sequence) included 740 nucleotide positions.

Three independent sets of phylogenetic analyses were performed: 1) Maximum Likelihood and Bayesian analyses with concatenated mitochondrial COI and 16S sequences; 2) Maximum Parsimony analyses with concatenated nuclear ITS2 and 28S sequences; 3) Maximum Parsimony analyses with ITS2 haplotype sequences. Maximum Parsimony analyses were conducted in PAUP v 4.0 (Swofford 2002) with gaps coded as a fifth character state, and 100 bootstrap replicates conducted using a full heuristic search. Prior to Maximum Likelihood and Bayesian phylogenetic analyses, the best-fitting evolutionary model was selected for each locus separately using the Model Selection option from Topali v2.5 (Milne et al. 2004): a GTR + G model was independently selected for COI and 16S. Maximum Likelihood analyses were performed using PhyML (Guindon and Gascuel 2003) as implemented in Topali. Node support was evaluated using bootstrapping with 100 replicates. Bayesian analyses were performed using MrBayes v3.1.2 (Ronquist and Huelsenbeck 2003) as implemented in Topali, with five simultaneous runs of 1.5×10^6^ generations each, sample frequency of 100, and burn in of 25% (and posterior probabilities were also calculated). Topali did not detect any issue with respect to convergence. All analyses were run several times and yielded the same result.

In addition, genetic distances between COI sequences were calculated in MEGA 7 as uncorrected p-distances. COI sequences were also translated into amino acid sequences in MEGA using the invertebrate mitochondrial genetic code to check for the presence of stop codons (no stop codon was found).

## Results

### Molecular phylogenetic analyses

The monophyly of *Peronia* is strongly supported in all analyses except in the mitochondrial ML analyses (bootstrap of 58), which confirms that all onchidiid slugs with dorsal gills belong to the same clade (Figs 2–4).

**Figure 2.**
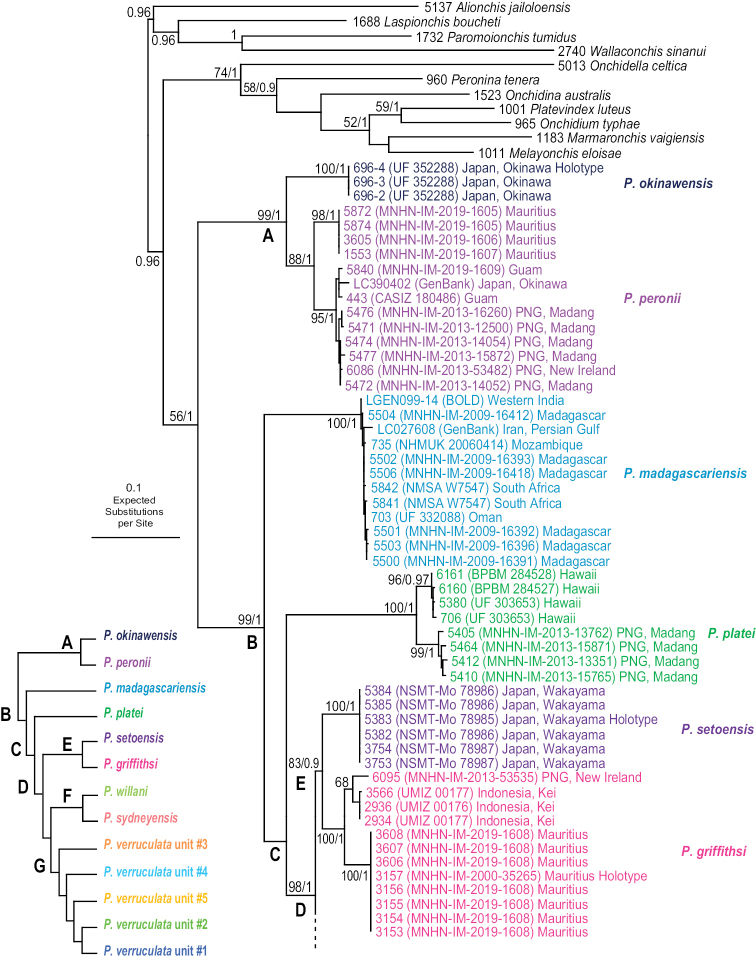
Phylogenetic relationships between *Peronia* species based on concatenated mitochondrial COI and 16S DNA sequences. Numbers by the branches are the bootstrap values (maximum likelihood analysis) and the posterior probabilities (Bayesian analysis). Only the values > 50% (ML) and > 0.9 (Bayesian) are indicated. Numbers for each individual correspond to unique identifiers for DNA extraction. Information on specimens can be found in the lists of material examined and in Table 2. The color used for each species or mitochondrial unit is the same as the color used in Figs 3–6.

**Figure 2.**
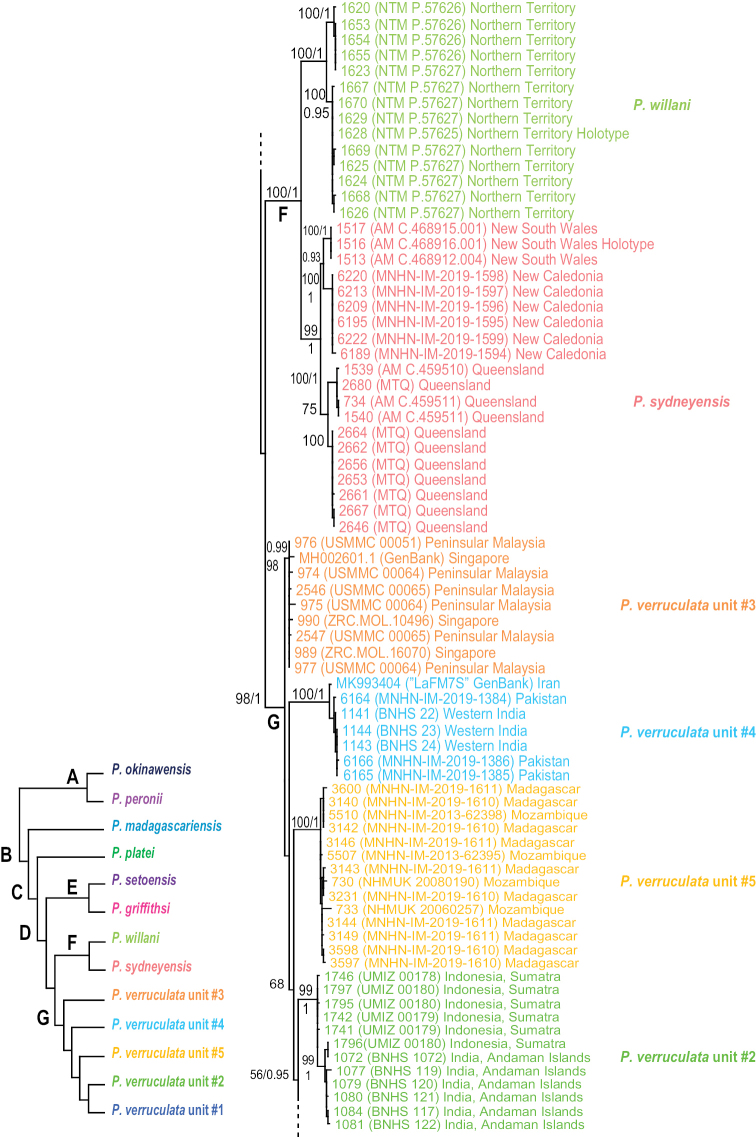
Continued.

**Figure 2.**
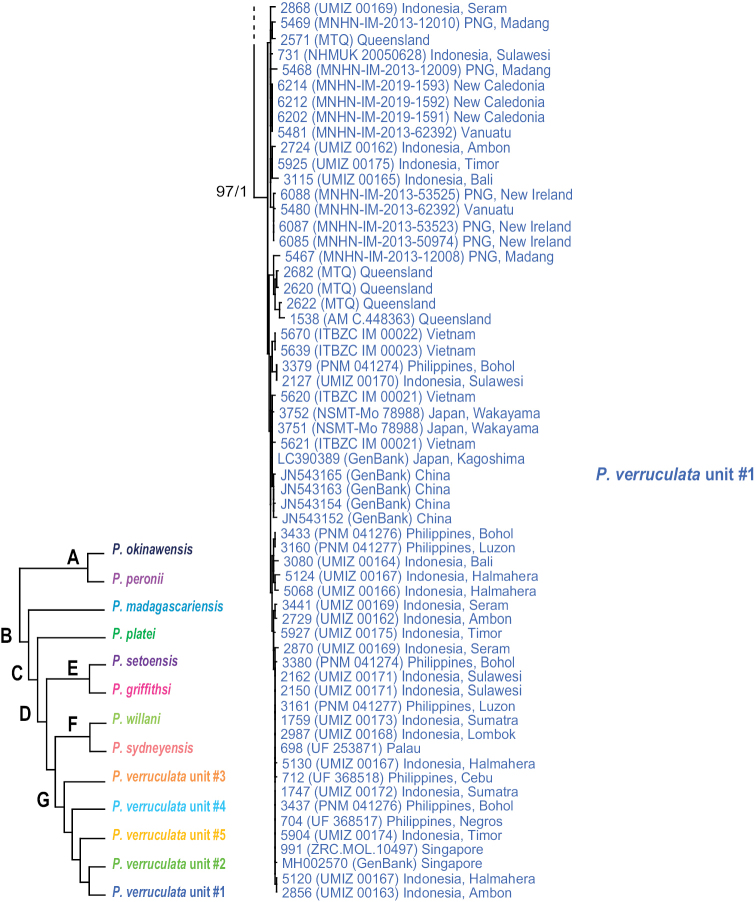
Continued.

**Figure 3.**
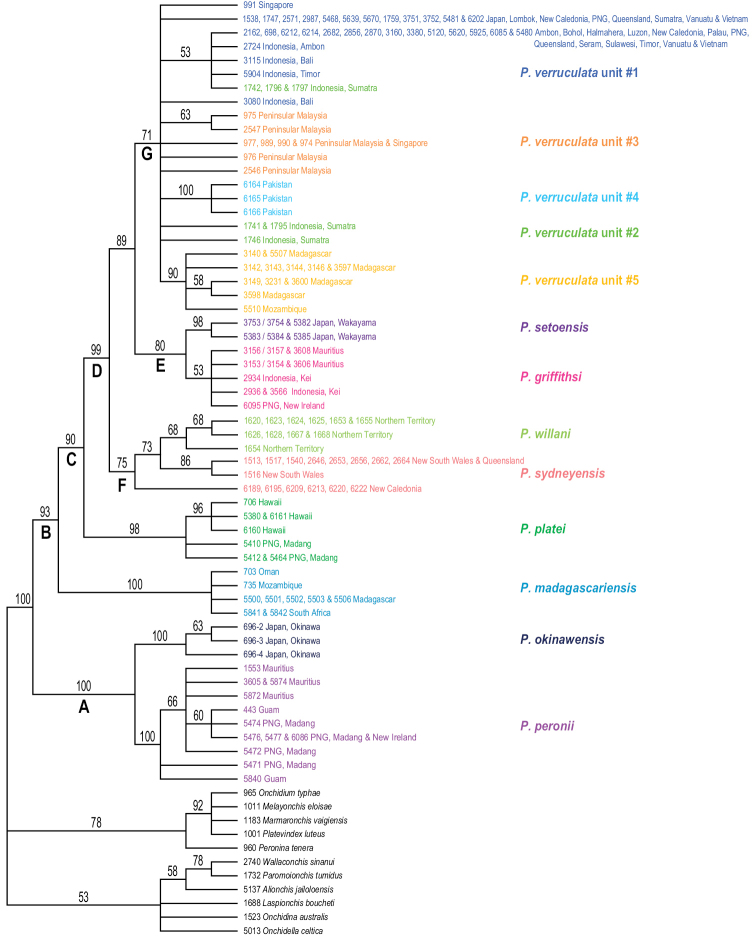
Maximum parsimony consensus tree within *Peronia* based on ITS2 sequences (identical sequences are represented as a single haplotype sequence). Numbers by the branches are the bootstrap values. Only the values > 50% are indicated. Numbers for each individual correspond to unique identifiers for DNA extraction. Information on specimens can be found in the lists of material examined and in Table 2. The color used for each species or mitochondrial unit is the same as the color used in Figs 2, 4–6.

**Figure 4.**
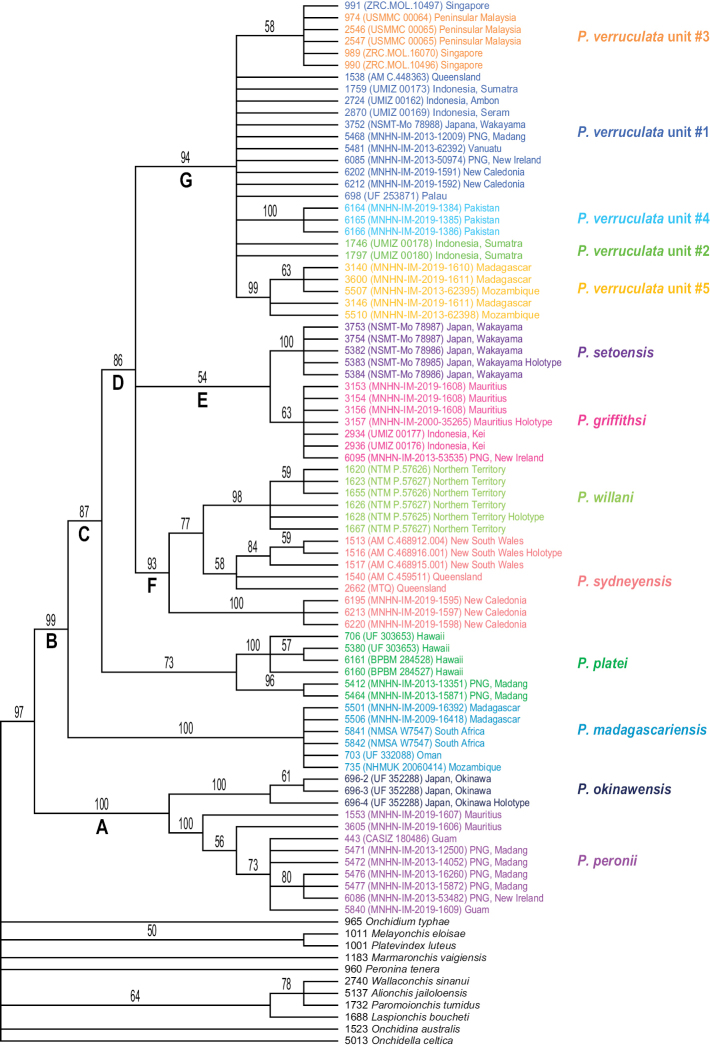
Maximum parsimony consensus tree within *Peronia* based on concatenated nuclear ITS2 and 28S sequences. Numbers by the branches are the bootstrap values. Only the values > 50% are indicated. Numbers for each individual correspond to unique identifiers for DNA extraction. Information on specimens can be found in the lists of material examined and in Table 2. The color used for each species or mitochondrial unit is the same as the color used in Figs 2, 3, 5, 6.

Seven nodes of higher relationships among *Peronia* species are well supported. Supports are indicated here in parentheses in the following order: ML bootstrap in mitochondrial analysis, Bayesian posterior probability in mitochondrial analysis, bootstrap in ITS2 analysis, bootstrap in ITS2 and 28S analysis (bootstrap values below 50% and posterior probabilities below 0.90 are replaced by a dash). Most basally, *Peronia* is always split in clades A and B. Clade A is strongly supported (99, 1.0, 100, 100) and includes *P. peronii* and *P. okinawensis*. Clade B is also strongly supported (99, 1, 93, 99) and includes clade C and *P. madagascariensis* as its most basal species. Clade C, which is consistently recovered but moderately supported (-, -, 90, 87), includes clade D and *P. platei* as its most basal species. Clade D (98, 1, 99, 86) includes the three clades E, F, and G, of which the relationships are unresolved (Fig. 4) or incongruent (Figs 2, 3). Clade E (83, 0.9, 80, 54) includes *P. griffithsi* and *P. setoensis*. Clade F (100, 1.0, 75, 93) includes *P. sydneyensis* and *P. willani*. Clade G (97, 1, 71, 94) includes the five least-inclusive mitochondrial units of *P. verruculata*. The relationships of those five units are not resolved (all support values are very low).

The monophyly of each species recognized here is strongly supported in all analyses, except for the special case of *P. sydneyensis* (see below, species delineation). Within four species, some least-inclusive units are supported by the mitochondrial markers but not by comparative anatomy and nuclear markers (Figs 2–4): two units within *P. peronii* (one unit from Mauritius and the other from the West Pacific); two units within *P. platei* (one unit from Hawaii and the other from Papua New Guinea); two units within *P. griffithsi* (one unit from Mauritius and the other from Kei Islands and Papua New Guinea); and three units of *P. verruculata* from South-East Asia and the West Pacific (units #1, #2, and #3). Two least-inclusive mitochondrial units within *P. verruculata* from the western Indian Ocean (units #4 and #5) are also monophyletic in nuclear analyses (Figs 2–4) but are anatomically cryptic (see below). Note that populations of *P. verruculata* from the Red Sea are not represented in molecular analyses (see below, species delineation).

In mitochondrial analyses (Fig. 2), *P. sydneyensis* and *P. willani* form together the strongly supported clade E and the monophyly of each species is also strongly supported. In nuclear analyses (Figs 3, 4), they also form a strongly supported clade but *P. sydneyensis* is paraphyletic with respect to *P. willani*. Both species are close geographically (*P. sydneyensis* is distributed in New South Wales, Queensland and New Caledonia, and *P. willani* is distributed in the Northern Territory) and may be the result of a recent divergence. The paraphyly in nuclear analyses most likely is the result of incomplete lineage sorting (see below, species delineation).

### Pairwise genetic divergences

**Figure 5.**
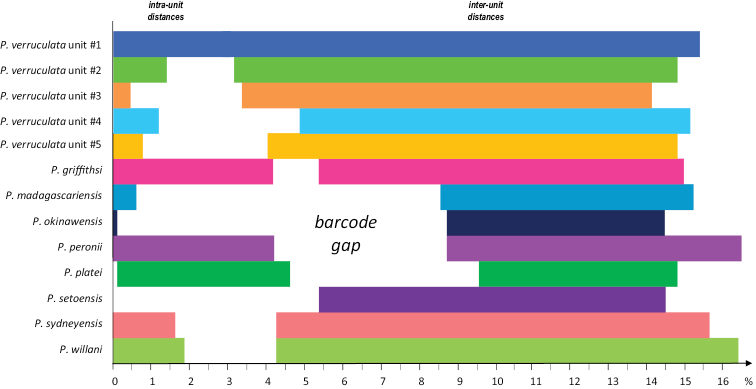
Diagram to help visualize the data on pairwise genetic distances between COI sequences within and between species and mitochondrial units (*P. verruculata*) in *Peronia* (see Table 3). Ranges of minimum to maximum distances are indicated (in percentages). For instance, within *P. willani*, individual sequences are between 0 and 1.9% divergent; individual sequences between *P. willani* and the other species or units are minimally 4.3% and maximally 16.8% divergent. The colors are the same as those used in Figs 2–4, 6.

Pairwise genetic distances were calculated for a total of 13 units (Fig. 5, Table 3): the five mitochondrial units within *P. verruculata* as well as the eight other species. A barcode gap is found in all cases, apart from the mitochondrial unit #1 of *P. verruculata*.

**Table 3.** Pairwise genetic distances between mitochondrial COI sequences in *Peronia*. Ranges of minimum to maximum distances are indicated (in percentage). For instance, the intra-specific divergences within *P. madagascariensis* are between 0 and 0.6%, while the inter-specific divergences between *P. griffithsi* and *P. madagascariensis* are between 9.3 and 11.3%.

Units		1	2	3	4	5	6	7	8	9	10	11	12	13
**1**	*P. verruculata* (unit #1)	0.0–3.6												
**2**	*P. verruculata* (unit #2)	3.2–6.6	0.0–1.4											
**3**	*P. verruculata* (unit #3)	3.4–5.4	4.7–6.1	0.0–0.4										
**4**	*P. verruculata* (unit #4)	4.9–8.2	6.6–7.3	4.9–6.0	0.0–1.2									
**5**	*P. verruculata* (unit #5)	4.0–6.1	4.7–6.4	4.5–5.4	6.5–8–2	0.0–0.8								
**6**	*P. griffithsi*	7.5–10.4	8.3–10.0	7.5–8.7	8.0–9.7	7.0–9.0	0.0–4.2							
**7**	*P. madagascariensis*	9.3–11.3	9.3–10.6	9.5–10.3	9.2–11.0	8.5–10.0	9.3–11.3	0.0–0.6						
**8**	*P. okinawensis*	12.0–14.0	12.8–13.6	12.3–13.1	13.6–14.2	11.5–12.4	13.0–14.5	12.0–12.8	0.0–0.2					
**9**	*P. peronii*	11.3–15.4	11.5–14.7	12.1–14.2	12.6–15.2	12.0–14.8	11.5–15.0	12.4–15.3	8.6–9.4	0.0–4.3				
**10**	*P. platei*	9.5–12.5	10.5–12.9	11.1–13.5	11.1–12.9	12.1–14.1	10.9–13.3	14.2–14.8	12.8–13.6	11.3–13.5	0.2–4.7			
**11**	*P. setoensis*	7.1–8.8	8.3–8.6	7.0–7.5	6.5–7.0	8.7–9.5	5.4–7.0	11.0–11.3	13.9–14.2	11.3–14.5	10.9–12.0	0.0–0.0		
**12**	*P. sydneyensis*	6.8–9.2	7.6–9.3	6.6–8.0	7.0–8.0	7.0–8.3	5.6–6.8	10.0–11.6	12.6–13.8	14.0–15.7	9.8–11.7	7.1–7.8	0.0–1.6	
**13**	*P. willani*	7.3–9.2	8.3–9.1	6.1–8.0	7.5–8.9	7.8–9.3	5.9–7.5	10.3–11.4	13.8–14.6	13.8–16.8	11.4–12.2	6.5–6.6	4.3–5.4	0.0–1.9

### Comparative anatomy

All *Peronia* slugs are characterized by dorsal gills which are not found in other onchidiids. They are also all characterized by a unique combination of internal traits: they are the only onchidiid slugs with intestinal loops of type I or V, an accessory penial gland, and no rectal gland. The fact that any slug with this combination of traits belongs to a *Peronia* species is helpful to identify specimens with dorsal gills retracted inside the notum.

There are no external differences between *Peronia* species. In the field, it is not possible to reliably identify any of them, especially because sympatric species are often found together at the exact same sites. Individuals of very large size (longer than 100 mm) are only found in *P. peronii*, but smaller individuals are impossible to distinguish externally from other species. Also, tall papillae over the entire notum seem to be mostly found in *P. peronii* and *P. madagascariensis*, but that may be due to the fact that slugs of both species are the largest, and it remains difficult to define exactly what a tall papilla is because papilla size is highly variable.

**Figure 6.**
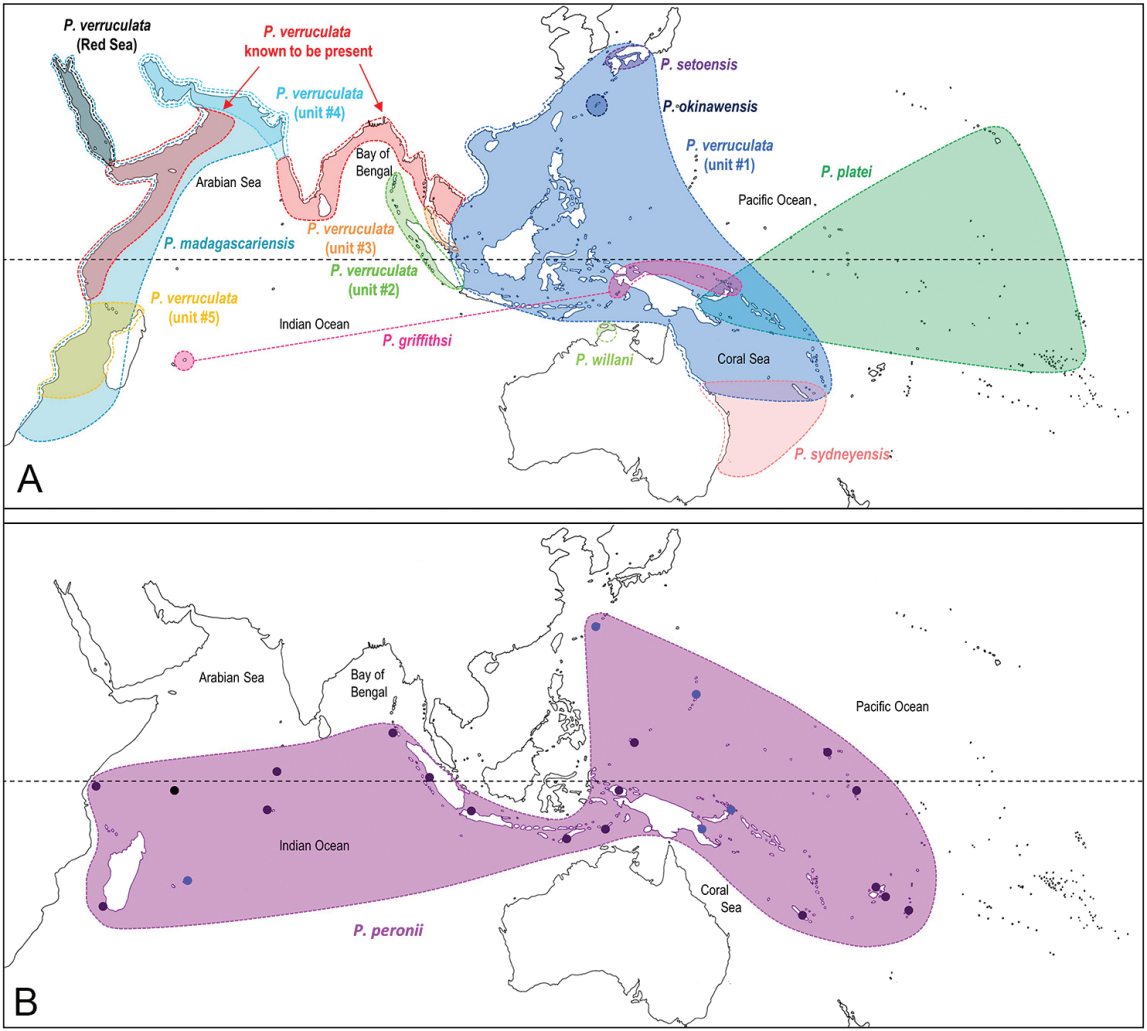
Geographical distribution of the *Peronia* species **A** distribution of all *Peronia* species except for *P. peronii***B** distribution of *P. peronii*. The colors are the same as those used in Figs 2–5. Colored areas correspond to hypothetical geographical ranges based on confirmed records only. Distinct colors are used for each unit of *P. verruculata*. The distribution of *P. verruculata* in the Indo-West Pacific is actually continuous. However, because it is unclear which units are present in regions from where we have no fresh material of *P. verruculata* (red areas), no unit of *P. verruculata* is shown there. For *P. peronii*, black dots correspond to material identified based on anatomical characters and blue dots correspond to material with DNA sequences. Details on species distribution can be found in each species description.

Internal differences help identify some species reliably, but not all (Table 4). Internal differences are almost exclusively based on combinations of traits because no *Peronia* species is characterized by any unique, distinctive feature, except for *P. peronii* (characterized by a spine of the accessory penial gland longer than 3 mm) and *P. sydneyensis* (characterized by strong protuberances on the spine of the accessory penial gland), and it remains difficult to identify *Peronia* species anatomically. For instance, where they overlap geographically (Queensland and New Caledonia), *P. verruculata* and *P. sydneyensis* can only be distinguished based on the length of the spine of the accessory penial gland, the presence of strong protuberances near the tip of the spine of the accessory penial gland, and the length of the penial hooks, which are all traits that are hardly accessible to a non-expert. However, only two *Peronia* species are cryptic externally and internally: *P. setoensis* and *P. platei*, which are not sister taxa (Figs 2–4) and do not overlap geographically, at least based upon current data (Fig. 6). Finally, the mitochondrial units of *P. verruculata* cannot be reliably distinguished anatomically.

Types of intestinal loops are useful for the identification of *Peronia* species (Fig. 1): species are characterized by intestinal loops of type V (*P. griffithsi*, *P. madagascariensis*, *P. platei*, and *P. setoensis*), type I with a transitional loop oriented between 12 and 3 o’clock (*P. okinawensis* and *P. peronii*), or type I with a transitional loop oriented between 3 and 6 o’clock (*P. sydneyensis*, *P. verruculata*, *P. willani*). Exceptions exist but are remarkably rare: only one individual in *P. sydneyensis* was found with a transitional loop slightly outside the range of that species (at 2 o’clock). Types of intestinal loops, however, can only be used in combination with other traits for the purpose of species identification.

The insertion of the retractor muscle of the penis is not very useful in identification because it mostly matches the distribution of the respective intestinal loop types (Table 4). An insertion near the heart is only found in the two species with intestinal loops of type I and transitional loops oriented between 12 and 3 o’clock (*P. peronii* and *P. okinawensis*). Within each species, all individuals share the same insertion of the retractor muscle (either near the heart or at the posterior end of the visceral cavity). However, in *P. griffithsi*, which is widely distributed from the West Pacific to Mauritius, individuals are characterized by both insertions. In *P. peronii*, the retractor muscle can exceptionally be vestigial (with no clear insertion).

The length of the muscular sac of the accessory penial gland varies depending on the size of animals, but it is useful to help identify some species. Indeed, only two species (*P. peronii* and *P. willani*) are characterized by a muscular sac which is longer than 20 mm (Table 4). The length of the spine of the accessory penial gland is helpful to distinguish closely related species which, otherwise, are very similar anatomically: *P. peronii* (at least 3 mm) and *P. okinawensis* (less than 2.3 mm); *P. setoensis* (more than 0.9 mm) and *P. griffithsi* (less than 0.62 mm); and *P. sydneyensis* (less than 1 mm) and *P. willani* (more than 1.5 mm). The diameter of the spine at its base can be used in exactly the same way. The length of penial hooks also differs between species: the longest hooks are found in *P. madagascariensis* (up to 100 μm), the shortest in *P. setoensis* and *P. griffithsi* (less than 25 μm).

**Table 4.** Summary of traits that can help identify *Peronia* species, based exclusively on specimens examined for the present revision. Species are arranged in a phylogenetic order (Figs 2–4). Traits are described in detail in species descriptions.

Species		Max. animal length (mm)	Papillae with dorsal eyes	Intestinal type (transitional loop orientation)	Retractor muscle insertion (near heart or end of visceral cavity)	Muscular sac length (mm)	Accessory penial gland spine length (mm)	Accessory penial gland spine diameter base (μm)	Accessory penial gland spine diameter tip (μm)	Penis hooks length (μm)	Distribution
*P. peronii*		140	15–20	I (12–3 o’clock)	heart (exc. vestigial)	>20	3.0–5.0	400–500	160–200	<50	Indo-West Pacific (Zanzibar to Okinawa and Tonga)
*P. okinawensis*		27	10–15	I (12–3 o’clock)	heart	<15	1.8–2.3	240–300	115–150	<35	Japan (Okinawa)
*P. madagascariensis*		80	12–18	V	end of VC	<15	2–2.4	200–230	70–80	<100	Western Indian Ocean
*P. platei*		30	7–10	V	end of VC	<5	0.7–1.0	65–100	20–30	<60	Western Pacific (PNG to Hawaii & French Polynesia)
*P. setoensis*		20	8–12	V	end of VC	<5	0.9–1.2	80–85	15–25	<25	Japan (Wakayama)
*P. griffithsi*		25	6–10	V	end of VC (exc. heart)	<5	0.50–0.62	60–65	15–20	<25	Indo-West Pacific (Mauritius to Kei Islands & New Ireland)
*P. sydneyensis*		50	8–16	I (3–6 o’clock)	end of VC	<10	0.6–1.0	90–100	20–50	<30	Queensland, New South Wales & New Caledonia
*P. willani*		65	10–25	I (3–6 o’clock)	end of VC	<25	1.5–1.9	240–250	80–100	<37	Northern Territory
*P. verruculata*	#1	60 (exc. 73)	10–22	I (3–6 o’clock)	end of VC	<15	1.4–2.0	100–270	35–50	<50	Singapore to eastern Australia, New Caledonia & Japan
	#2	55	14–22			<10	1.4–1.7	140–160	30–35		Sumatra & Andaman
	#3	40	10–18				1.8–2.2	200–270	40–80	<60	Malaysia & Singapore
	#4	60					(1.3) 2.2–2.8	200	50		Western India, Pakistan & Persian Gulf
	#5	50	10–20				1.8–2.0	150–180	45–50	<55	Mozambique & Madagascar
	#6	40	10–18			<15	2.0–2.4	140–200	55–60		Red Sea

### Species delineation

The delineation of *Peronia* species is straightforward. They are all supported by independent data sets: they are reciprocally monophyletic with both mitochondrial and nuclear markers, and their monophyly is strongly supported; they are all separated by a large barcode gap; and they each are characterized by a unique combination of anatomical traits (with the exception of *P. setoensis* and *P. platei*, which are cryptic). Only two species need special attention: *P. sydneyensis* and *P. verruculata*.

The paraphyly of *P. sydneyensis* with respect to *P. willani* in nuclear analyses most likely is the result of incomplete lineage sorting, because lineage sorting progresses more rapidly for mitochondrial alleles than for nuclear alleles (Funk and Omland 2003). Also, *P. sydneyensis* and *P. willani* species are clearly distinct anatomically: in particular, *P. sydneyensis* is characterized by unique, strong protuberances near the tip of the spine of the accessory penial gland. Therefore, *P. sydneyensis* and *P. willani* are regarded as two recent but well-delineated species.

Despite some genetic structure, *Peronia verruculata* is regarded as a single species for various reasons. In mitochondrial analyses, *P. verruculata* is split in five least-inclusive mitochondrial units of which the relationships are basically unresolved due to low support (Fig. 2). In nuclear analyses, the mitochondrial units #1, #2, and #3 are not monophyletic (Figs 3, 4). Therefore, they should not be recognized as distinct taxa. In nuclear analyses, units #4 and #5 are monophyletic (Figs 3, 4). However, the mitochondrial units #1, #2, and #3 do not form a monophyletic group with respect to units #4 and #5. Recognizing mitochondrial units #4 and #5 each as a separate taxon would mean that mitochondrial units #1, #2, and #3 would also have to be recognized as separate taxa, which is unwarranted for the reasons given above.

All mitochondrial units of *P. verruculata* are cryptic anatomically (their anatomical traits display overlapping variation) while *P. verruculata* is clearly distinct from other *Peronia* species (Table 4). Finally, it would seem premature to recognize units #4 and #5 as independent lineages because our geographical sampling of *P. verruculata* is not continuous (Fig. 6). Future samples from southern India (including Sri Lanka) or the Arabian Sea (the coast of Yemen, Oman, Somalia) might show that the individuals of the western mitochondrial units #4 and #5 can still interbreed, exactly like units #1, #2, and #3. We therefore refrain from naming those five mitochondrial units within *P. verruculata*. They are merely regarded as mitochondrial units that indicate some genetic structure, but the current data do not suggest that they should be recognized as distinct taxa. Note that taxon names are already available for the mitochondrial units #1, #4, and #5 of *P. verruculata* (Table 1).

Finally, note that *Peronia verruculata* was described from the Red Sea, from which no fresh material could be obtained. However, the specimens examined from the Red Sea are anatomically indistinguishable from the specimens of the five mitochondrial units of *P. verruculata* (Table 4). Therefore, at this stage, there is no reason to think that the populations from the Red Sea belong to a distinct species and that the name *P. verruculata* cannot apply to the whole species from the Red Sea and South Africa all the way to the West Pacific (Japan, New Caledonia, and Queensland). At any rate, there are plenty of available names that can be used in the future if it were to be demonstrated that the populations from the Red Sea belong to a distinct species (see remarks on *P. verruculata*).

### Species distribution

Geographic distribution is discussed in detail with each species description. The map of species distributions only illustrates the records that are regarded as correct (Fig. 6). Most of those correct records correspond to the specimens included in our molecular analyses. However, they also include types as well as historical museum specimens and records from the literature which could be positively identified using anatomical traits (e.g., intestinal loops, length of the spine of the accessory penial gland).

The secondary literature was read with great attention, especially in cases where it could provide geographical records not included in our material. Every record found in the literature is commented on (in the species remarks). Records from the literature are certainly not taken for granted because the secondary literature is plagued with two major issues. First, past authors did not always take the time to examine type specimens. For instance, Labbé (1934a) did not examine the types of *Onchidium verruculatum* and *Onchidium peronii* which are preserved at the Paris Museum (he did not list them in the material examined for these species), even though his study was almost exclusively based on material from that institution. Second, because there was no proper knowledge about intraspecific character variation, nobody knew which character could help distinguish species or not. For instance, Hoffmann’s (1928: 73) record of *Peronia verruculata* from Hawaii was never questioned, but *Peronia* slugs from Hawaii are all characterized by intestinal loops of type V, which means that they cannot belong to *P. verruculata* (which is characterized by intestinal loops of type I).

### Systematics and anatomical descriptions

#### Family Onchidiidae Rafinesque, 1815

##### Genus Peronia

Taxon classificationAnimaliaSystellommatophoraOnchidiidae

Fleming, 1822a

Onchis Férussac, 1822: xxxi. Nomen oblitum.
Peronia Fleming, 1822a: 574; Fleming 1822b: 463. Nomen protectum.
Peronia Blainville, 1824: 280 [junior homonym of Peronia Fleming, not a reference of Peronia Fleming].
Eudrastus Gistel, 1848: x.
Paraperonia Labbé, 1934a: 196.
Scaphis Labbé, 1934a: 203.
Lessonia Labbé, 1934a: 213 [junior homonym of Lessonia Swainson, 1832, replaced by Lessonina Starobogatov, 1976].
Quoya Labbé, 1934a: 216.
Lessonina Starobogatov, 1976: 211.
Quoyella Starobogatov, 1976: 211 [unnecessary replacement name for Quoya Labbé, 1934a].

###### Type species.

*Onchis*: *Onchidium peronii* Cuvier, 1804, by monotypy.

*Peronia* Fleming: *Onchidium peronii* Cuvier, 1804, by monotypy.

*Peronia* Blainville: *Peronia mauritiana* Blainville, 1824, by original designation.

*Eudrastus*: *Onchidium tonganum* Quoy & Gaimard, 1832, by subsequent designation (Baker 1938: 86).

*Paraperonia*: *Paraperonia gondwanae* Labbé, 1934a, by subsequent designation (Starobogatov 1976: 211).

*Scaphis*: *Onchidium astridae* Labbé, 1934b, by subsequent designation (Starobogatov 1976: 211).

*Quoya*: *Quoya indica* Labbé, 1934a, by monotypy.

*Lessonina*: *Onchidium ferrugineum* Lesson, 1831a, by monotypy.

*Quoyella*: *Quoya indica* Labbé, 1934a, by monotypy.

###### Etymology.

*Onchis*: After the Greek *ὁ ὂγκος*, *oncos*, which means mass, or tumor.

*Peronia*: After François Péron [1775–1810], zoologist of the Baudin expedition between 1800 and 1803, during which he collected the two slugs (from Mauritius and Timor) which Cuvier described as *Onchidium peronii* in 1804.

*Eudrastus*: Likely, although for unclear reasons, from the Greek *εὖ*, *eu*, for true, and *δραστέoς*, *drasteos*, a verbal adjective which means to be done.

*Paraperonia*: From the Greek *παρα*, *para*, meaning beside, and *Peronia*.

*Scaphis*: After the Greek *ἡ σκᾰφίs*, which means small boat (Labbé, 1934a: 202).

*Quoya*: After the French naturalist Jean René Constant Quoy [1790–1869], a member of two circumnavigations from 1817 to 1820 with captain Freycinet and from 1826 to 1829 with captain Dumont d’Urville. Quoy and Joseph Paul Gaimard [1793–1858] described several species of onchidiids based on their collections in the southern seas. *Quoyella* has the same etymology.

*Lessonina*: After the French naturalist René Primevère Lesson [1794–1849], a member of a circumnavigation from 1822 to 1825 with captain Duperrey. Lesson described several species of onchidiids based on his collections in the southern seas, such as the type species of *Lessonina*, *Onchidium ferrugineum*, which he collected in West Papua, Indonesia. Labbé’s invalid name *Lessonia* was also dedicated to Lesson.

###### Gender.

*Onchis*: Masculine. Férussac did not specify the gender of *Onchis* which he did not combine with any specific name, and even the binomen *Onchis peronii*, which Férussac did not use *per se*, would not help in that respect. Because *Onchis* is derived from the masculine Greek noun *ὁ ὂγκος*, it is considered to be of masculine gender.

*Peronia*: Feminine. No gender was specified by Fleming, and the combination *Peronia peronii* does not help to determine it. Because no gender was originally specified or indicated and because *Peronia* ends in -*a*, it is treated as a name of feminine gender (ICZN 1999: Article 30.2.4). Indeed, *Peronia mauritiana*, an early combination used by Blainville (1824: 281), shows that *Peronia* has always been treated as a name of feminine gender.

*Eudrastus*: Masculine. No gender was originally specified or indicated. *Eudrastus* ends in a word derived from a word of variable gender (a verbal adjective) and should be treated as masculine (ICZN 1999: Article 30.1.4.2).

*Paraperonia*: Feminine. Gender of *Peronia*.

*Scaphis*: Feminine. The gender was not specified by Labbé, but his original combinations *S. atra*, *S. carbonaria*, *S. lata*, and *S. punctata* indicate that he treated *Scaphis* as a name of feminine gender, which is correct since *Scaphis* is derived from the feminine Greek noun *ἡ σκᾰφίs*.

*Quoya*: Feminine. The gender was not specified by Labbé, but his original combination *Q. indica* indicates that he treated *Quoya* as a name of feminine gender, which is assumed to be the gender of *Quoyella* as well.

*Lessonina*: Feminine. The gender was not specified by Starobogatov, and no gender was specified for *Lessonia* by Labbé. Labbé’s original combination *Lessonia ferruginea* indicates that he treated *Lessonia* as a name of feminine gender, which is assumed to be the gender of *Lessonina* as well.

###### Diagnosis.

Body not flattened. Dorsal gills present. Dorsal eyes present. No retractable, central papilla present. Eyes at tip of short ocular tentacles. Male opening below right ocular tentacle and to its left. Foot wide. Pneumostome median, on ventral hyponotum. Intestinal loops of types I or V. Rectal gland absent. Accessory penial gland present, with muscular sac. Penis with hooks.

###### Remarks.

Phylogenetic analyses show that all species of slugs with dorsal gills belong to the same clade (Figs 2–4). Seven generic names apply to that clade (excluding spelling mistakes, unjustified emendations, replaced names, and *Peronia* Blainville, 1824, a junior homonym of *Peronia* Fleming, 1822a). Note that the species name of a type species can be valid (such as *Peronia peronii*), synonymous (such as *Onchidium tonganum*, junior synonym of *P. peronii*), or even a *nomen dubium* (such as *Quoya indica*). Remarks on the nomenclatural history of the genus *Peronia* follow a chronological order.

Cuvier (1804) described the first *Peronia* species as *Onchidium peronii* but did not mention the presence of dorsal gills. Nor did he illustrate them. He only described a mantle covered by small warts subdivided in even smaller warts. Dorsal gills are actually present on the dorsum of the type specimen of *Onchidium peronii* from Timor, but they are retracted, as most often seen in preserved specimens. Cuvier (1804: 41) also confessed that he would have believed *O. peronii* to be terrestrial, due to its pulmonary cavity “similar to that of reptiles”, but that he regarded it as marine because Péron was certain to have collected it in seawater. But, Cuvier (1804: 41) adds: “I think at least that it comes to the surface to open its [pulmonary] hole, and naturally take air to breathe, as do our *bulines* [*Bulinus*] and our *planorbes* [*Planorbis*] which, although aquatic, breathe only air.” Actually, *Peronia* slugs hide in crevices at high tide and only come out at low tide.

Cuvier (1804: 38) decided to classify his new species in Buchannan’s (1800) genus *Onchidium* because of the “extreme external resemblance” between *O. peronii* and Buchannan’s *O. typhae*, despite the fact that, according to Buchannan, sexes are separate in *O. typhae*, while Cuvier’s *O. peronii* is hermaphroditic. In his description of *O. typhae*, Buchannan (1800) wrote that slugs live in Bengal on leaves of *Typha* reeds and are “very nearly allied” to *Limax*, suggesting that they are terrestrial, although he did not mention the presence of a pulmonary cavity and did not clearly state whether the slugs were terrestrial or not. At any rate, authors considered that Buchannan’s (1800)*O. typhae* was not a marine species and Blainville (1817: 440) argued that Buchannan’s *O. typhae* was “generically” different from Cuvier’s *O. peronii*, and that *Onchidium* should be restricted to *O. typhae*. However, Blainville (1817) did not propose any new generic name for Cuvier’s (1804)*Onchidium peronii*.

In his *Histoire naturelle générale et particulière des mollusques terrestres et fluviatiles*, Férussac (1819: 80–82) agreed with Blainville (1817) that Cuvier’s (1804)*Onchidium peronii* was distinct from Buchannan’s (1800)*O. typhae*, but, like Blainville, he refrained from creating a new generic name for *O. peronii*. A year later, Fleming (1820: 616) stated that he thought Cuvier’s *O. peronii* should probably be classified in a different genus from Buchannan’s *Onchidium*: “This species [*Onchidium typhae* Buchannan], however, if the description be accurate, differs essentially from the one described by Cuvier [*Onchidium peronii*], and would lead us to infer that a new genus would be necessary for the reception of the species of the last-mentioned naturalist.”

The two generic names *Onchis* and *Peronia* were independently created in 1822 for *O. peronii*, respectively by Férussac (1822) and Fleming (1822a, b) who both follow Blainville’s (1817) argument according to which a marine and hermaphroditic species (*O. peronii*) cannot be classified in the same genus as a terrestrial species with separate sexes (*O. typhae*). Interestingly, neither Férussac (1822) nor Fleming (1822a, b) mention dorsal gills (which, again, Cuvier did not mention in the original description of *O. peronii*). Dorsal gills were first illustrated by Savigny (1817: pl. 2, fig. 3.5) in the *Description de l’Egypte* for slugs from the Red Sea; for a collation, see Baring (1838) and Sherborn (1897). However, gills remained completely unnoticed because the explanation of Savigny’s plate was published nearly ten years later by Audouin (1826: 18–20).

The exact date of publication of *Onchis* is 13 April 1822 (when the pages xxv–xlvii were published); a collation for Férussac’s (1821–1822)*Tableaux* can be found in Coan and Kabat (2019). Férussac (1822: xxxi) clearly distinguishes two genera of onchidiids: “Genre I. Onchide, *Onchis*; Onchidium, Cuvier, Ocken. (Marin.)” and “Genre II. Onchidie, *Onchidium*, Buchannan, Ocken.” Note that Oken (1815: 307), to which Férussac (1822) refers, merely listed *Onchidium typhae* and *O. peronii*. According to Férussac, *Onchis* clearly refers to Cuvier’s *Onchidium peronii*, supposedly marine and living underwater, and *Onchidium* is restricted to Buchannan’s *Onchidium typhae*, thought not to live underwater. In the *Tableau systématique de la famille de limaces* (part of the 16^th^ livraison published on 13 July 1822), Férussac (1822: 8) also considered two genera: the “premier genre,” i.e., the first genus ever described, Buchannan’s *Onchidium*, and another genus, unnamed, with *Onchidium peronii* as type.

*Onchis* is not etymologically rigorous. The latinization of *ὂγκος* is *oncos* or *oncus*, as in the English word oncology. The Greek letter *κ* is “c” in Latin, while *χ* becomes “ch.” That Férussac used *onchis* instead of *oncos* is not surprising, as naturalists often took liberties with the latinization of Greek words. A famous example being the word taxonomy, created as *taxonomie* by De Candolle (1813: 19) from the Greek words *taxis* (arrangement, order) and *nomos* (law, rule): *taxis* should have stayed as *taxi*- to form *taxinomie*, taxinomy, exactly like in the English word taxidermy (from *taxis* and *dermis*, skin). However, the Code does not require taxon names to be etymologically correct. Therefore, the intentional spelling change of *Onchis* to *Oncus* by Agassiz (1846: 259; 1848: 748) is an unjustified emendation because *Onchis* is not the result of “inadvertent error, such as a lapsus calami or a copyist’s or printer’s error” (ICZN 1999: Article 32.5.1) and therefore *Onchis* must not be corrected. The emendation of *Onchidium* into *Oncidium* by Agassiz (1846: 259; 1848: 748) also is unjustified for the same reason.

The generic name *Peronia* first appeared in two different venues, both published by Fleming (1822). One venue is Fleming’s (1822a: 574) article “Mollusca” in the fifth volume (second part) of the *Supplement to the fourth*, *fifth*, *and sixth editions of the Encyclopædia Britannica* published in May 1822 (as clearly indicated in a memorandum at the end of the sixth volume of the *Supplement*), even though the *Supplement* was only completed in 1824 (date on the title page). The other venue is Fleming’s (1822b: 463) *Philosophy of Zoology* which, according to Feuer and Smith (1972: 55), was not published earlier than May 1822 but no later than June 1822. The mention of *Peronia* in the *Supplement* is considered here to be the earliest one because it was published in May 1822.

*Peronia* Fleming, 1822a is an objective junior synonym of *Onchis* Férussac, 1822, because Férussac’s *Onchis* was published prior to Fleming’s *Peronia* and both generic names share the same type species (*Onchidium peronii*). However, to the best of our knowledge, *Onchis* has only been used twice in a binomen, and both times before 1899: by Stimpson (1855) for *Onchis fruticosa*, a species name that has remained unnoticed until now, and by Mörch (1863) for Onchis (Peronella) armadilla Mörch, 1863, i.e., *Onchidella armadilla* (Mörch, 1863). Reversal of precedence applies here (ICZN 1999: Article 23.9). *Onchis*, the senior synonym, “has not been used as a valid name after 1899” (ICZN 1999: Article 23.9.1.1) and *Peronia*, the junior synonym, “has been used for a particular taxon, as its presumed valid name, in at least 25 works, published by at least 10 authors in the immediately preceding 50 years and encompassing a span of not less than 10 years.” (ICZN 1999: Article 23.9.1.2) A chronological list of 25 works meeting the criteria of ICZN Article 23.9.1.2 is provided here, all of which mentioning *Peronia*, *Peronia verruculata*, or *Peronia peronii* as valid names: Marcus and Marcus (1970), Starobogatov (1976), Britton (1984), Biskupiak and Ireland (1985), Faulkner (1987), Pietra (1990), Arimoto et al. (1993), Davies-Coleman and Garson (1998), Pietra (2002), Nakaoka et al. (2006), Carbone et al. (2009), Morrisey et al. (2010), Dayrat et al. (2011), White et al. (2011), Gaitán-Espitia et al. (2013), Mandal and Harkantra (2013), Sun et al. (2014), Bitaab et al. (2015), Harasewych et al. (2015), Liu et al. (2015), Wardiatno et al. (2015), Sun et al. (2016), Santhosh Kumar et al. (2016), Solanki et al. (2017), Xu et al. (2018). *Onchis* Férussac, 1822, objective senior synonym, is regarded as a *nomen oblitum*, and *Peronia* Fleming, 1822a, objective junior synonym, is regarded as a *nomen protectum* (ICZN 1999: Article 23.9.1.2).

Fleming (1822a: 571, 574) classified *Onchidium* (with only the type species *O. typhae*) in a group of slugs that “reside constantly on the land,” and transferred *O. peronii* to *Peronia*, a genus for marine slugs that have “their residence constantly in water” and look like *Onchidium*. However, Fleming (1822a: 574) expressed doubts that *Peronia* slugs are air-breathing, as Cuvier (1804) claimed in the original description of *O. peronii*:

“This genus, which we have named in honor of M. Peron, was referred by Cuvier to the Onchidium of Buchanan (…) and the species termed *O. Peronii*. It was found creeping upon marine rocks, under water, at the Mauritius, by M. Peron. M. Cuvier conjectures that it breathes free air, and has accordingly inserted it among the *Pulmones aquatique* [*Pulmonés aquatiques*, i.e., aquatic pulmonates]. Some doubts, however, may reasonably be entertained about the truth of this supposition. It would certainly be an unexpected occurrence to find a marine gasteropodous mollusca obliged to come to the surface at intervals to respire. It will probably be found that it is truly branchiferous.”

It was Audouin (1826) who demonstrated later that both Cuvier and Fleming were correct because *Peronia peronii* can breathe through both its pulmonary cavity and dorsal gills.

Blainville (1824: 280) created the generic name *Peronia* without being aware that Fleming (1822a, b) had already created exactly the same name two years before. Indeed, that Blainville (1824: 258) wrote “our genus *Péronie*” clearly suggests that he thought he was the author of *Peronia*. Also, most past authors attributed the authorship of *Peronia* to Blainville instead of Fleming (e.g., Stoliczka 1869: 100; Plate 1893: 102; Labbé 1934a: 189). *Peronia* Blainville, 1824 is a junior homonym of *Peronia* Fleming, 1822a and thus cannot be used as a valid name (ICZN 1999: Article 52.2). However, *Peronia* Blainville is also a junior objective synonym of *Peronia* Fleming, because they “both denote nominal taxa with name-bearing types whose own names are themselves objectively synonymous.” (ICZN 1999: “objective synonym” in the glossary) Indeed, *O. peronii*, the type species of *Peronia* Fleming, and *P. mauritiana*, the type species of *Peronia* Blainville are objective synonyms because they share the same lectotype, i.e., the specimen from Mauritius which Cuvier (1804: pl. 6) illustrated (see below, the comments on the type material of *O. peronii* and *P. mauritiana*).

When he created the generic name *Peronia*, Blainville (1824: 280, 281) cited only one species name, *Peronia mauritiana*, a junior objective synonym of *Onchidium peronii*. Blainville (1824: 281) also claimed that he knew four or five other species of marine onchidiids from the southern hemisphere, without naming them, but Blainville (1826: 523) listed them two years later (Table 1): *Peronia laevis*, a junior objective synonym of *Marmaronchis vaigiensis*; *Peronia semituberculata*, a junior objective synonym of *Onchidium planatum*, itself a *nomen dubium* which may or may not refer to an onchidiid species; *Peronia oniscoides*, which all authors ignored except for Labbé (1934a: 243) and which clearly does not refer to a *Peronia* species (see general discussion). In addition, Blainville (1826: 523) also pointed out that *Onchidium celticum*, a name which Cuvier used for small marine slugs from the coast of Brittany, France, could also refer to a *Peronia*; *Onchidium celticum* remained a *nomen nudum* until 1832, when it was described by Audouin and Milne-Edwards (1832: 118).

Like Cuvier (1804), Férussac (1822), and Fleming (1822a, b), Blainville (1824) did not mention the existence of dorsal gills. Dorsal gills were first described by Audouin (1826: 18–20) in the explanation of a plate by Savigny (1817: pl. 2) from the *Description de l’Egypte*. Savigny’s (1817: pl. 2, figs 3.1–3.8) plate displays eight drawings for two onchidiid slugs from the Red Sea, with one of them clearly representing a dorsal gill (Savigny 1817: pl. 2, fig. 3.5). According to Audouin (1826: 19), it was Cuvier himself who identified those two slugs as *Onchidium peronii*, although Cuvier (1830) later changed his mind and created the new name *Onchidium verruculatum* for them. More importantly, Audouin (1826: 19) described in great detail the “small vascular branches” at the posterior end of the dorsum, or “tubercles” that work as “true gills.” And Audouin (1826: 19) even made this clever statement:

“The Onchidie thus would have at the same time a pulmonary apparatus and a branchial apparatus; and that structure is in perfect agreement with what we know of the habits of that mollusk: Péron says that it is aquatic; on the contrary M. Cuvier, without the authority of this observer, would have believed it to be terrestrial. (...) We think that the Onchidie, at least the species illustrated here, enjoys the capacity to breathe under water thanks to the help of those ramified tubercles which cover the posterior end of its body, without the necessity of coming up to the surface; which is relatively difficult for an animal that slowly crawls at the bottom underwater. As for the pulmonary opening, it indicates that the onchidie breathes air as well; and we must suppose that several times in its life it finds itself in the condition to do so.”

Audouin supposedly assumed that those slugs were truly aquatic.

Because *Peronia* was originally used as a genus for all marine onchidiids by both Fleming (1822a, b) and Blainville (1824, 1826), several *Peronia* species names already existed by 1830: *Peronia mauritiana*, *P. peronii*, *P. oniscoides*, *P. semituberculata*, and *P. laevis* (see above). Of those names, only the two objective synonyms *P. mauritiana* and *P. peronii* refer to true *Peronia* slugs, i.e., slugs with dorsal gills (Table 1). Cuvier (1830: 46) did not see the need for a genus assignment for marine onchidiid species and still only recognized *Onchidium*, but other naturalists started transferring species names from *Onchidium* to *Peronia*. Lesson (1833: pl. 19) transferred his own *Onchidium ferrugineum* Lesson, 1831a to *Peronia*, and clearly specified that he agreed with Blainville that marine onchidiids should be classified in a distinct genus. Dorsal gills are very clearly described by Lesson (1831a: 128–130; 1831b: 300–302; 1832: 36–37, fig. 32; 1833: pl. 19) in *O. ferrugineum*, but they were not the reason why he transferred it from *Onchidium* to *Peronia*. Shortly after that, Oken (1834a) also transferred six *Onchidium* species names by Quoy and Gaimard (1832–1833) to *Peronia* (*P. cinerea*, *P. incisa*, *P. nigricans*, *P. patelloides*, *P. punctata*, and *P. tongana*), with no justification but most likely because he also adopted the idea that marine onchidiids should not be classified in *Onchidium*.

The name *Eudrastus* was created by Gistel (1848: x), as a replacement name for “Peronia (Quoy, Isis 1834. 287.).” Gistel refers here to a report (Oken 1834a: 283–310) on Quoy and Gaimard’s (1832–1833) contribution to the *Voyage de découvertes de l’Astrolabe* published in *Isis*, the encyclopedic journal edited by Lorenz Oken from 1817 to 1848. This report was most likely written by Oken himself, as was often the case (Kertesz 1986), which would explain that the six onchidiid specific names mentioned (*tongana*, *incisa*, *patelloides*, *nigricans*, *punctata*, *cinerea*) are combined with *Peronia* instead of *Onchidium*, the generic name originally used by Quoy and Gaimard (1832–1833). Regardless of who authored that *Isis* report, Gistel (1848) did create the new generic name *Eudrastus* for those six species. Baker (1938: 86) subsequently designated *Onchidium tonganum* Quoy & Gaimard, 1832 (*Peronia tongana* in *Isis*), as the type species of *Eudrastus*. *Onchidium tonganum* is regarded here as a junior subjective synonym of *Peronia peronii*, so *Eudrastus* is a junior subjective synonym of *Peronia*. Britton (1984: 182–183) suggested that *Eudrastus* should be regarded as a junior synonym of *Peronia* because it seemed to be based on “unimportant characters.”

John Edwards Gray (1847: 179) attributed the authorship of *Peronia* to Blainville (with an erroneous date of 1825) but, most importantly, gave its modern definition to *Peronia* by restricting it to six species of slugs with “radiating processes” on the back (JE Gray 1850: 117): *P. alderi*, *P. ferruginea*, *P. mauritiana*, *P. peronii*, *P. punctata*, and *P. tongana*. All those names refer to true *Peronia* slugs with dorsal gills. JE Gray (1850: 117) restricted *Onchidium* to Buchannan’s *O. typhae* and included all the other marine species without dorsal gills in a new genus *Onchidella*.

JE Gray’s (1850) clarity only lasted for a few years. Indeed, Adams and Adams (1855: 234) pointed out that *Peronia* slugs differ from *Onchidium* and *Onchidella* because of “arbusculiform and other appendages of the mantle, which have sometimes been mistaken for gills.” Because they did not believe that gills were distinct from other dorsal papillae, Adams and Adams (1855: 234) classified in *Peronia* some names that belong to both *Peronia* (*P. ferruginea*, *P. mauritiana*, *P. peronii*, *P. punctata*, *P. tongana*) and to *Onchidella* (*O. celtica*, *O. indolens*, *O. marginata*, and *O. parthenopeia*).

JE Gray’s (1850) classification was adopted by Keferstein (1865a) but, until Labbé’s (1934a) work, all authors have ignored the genus *Peronia* and simply used the genus *Onchidium* for slugs with and without dorsal gills (Stoliczka 1869; Semper 1880–1885; Plate 1893; Bretnall 1919; Hoffmann 1928). Stoliczka (1869: 100–102), who was the first one to re-examine live slugs of *O. typhae* since Buchannan (1800), firmly argued that slugs with “dorsal tufts” were anatomically so similar to *Onchidium* and *Onchidella* that only one name, *Onchidium*, was needed. Stoliczka (1869: 98) also clarified that *O. typhae* is not a terrestrial species but that, instead, it lives in “damp places, generally close to tanks or ditches, especially those which are supplied during high tide with brackish water.”

Stoliczka’s (1869) strong influence can be seen in Semper’s (1880–1885) study of the onchidiids from the Philippines (and other parts of the Indo-West Pacific) in which all onchidiids are in *Onchidium*, with the exception of a single species in his new genus *Onchidina* Semper, 1882; for a collation of Semper’s work, see Johnson (1969). Plate (1893) adopted a classification with five genera, but the four species of slugs with dorsal gills recognized by Plate are classified in *Onchidium* with thirteen species of slugs without dorsal gills. Hoffmann (1928) adopted a classification with six genera, six species of slugs with dorsal gills being classified in *Onchidium* with 34 species without dorsal gills.

Then, suddenly, in 1934, the number of onchidiid taxon names for slugs with dorsal gills dramatically increased. Based on the onchidiid collection at the Paris Museum, Labbé (1934a) created fourteen new species-group names for slugs with dorsal gills (all but one name are species names) and four new generic names: *Lessonia* (later replaced by *Lessonina*), *Paraperonia*, *Quoya*, and *Scaphis*. Below, the nomenclatural status of Labbé’s generic names is justified first (they all are junior synonyms of *Peronia*), followed by opinions in the secondary literature.

The generic name *Paraperonia* was created by Labbé (1934a: 196) for four species similar to *Peronia* but with intestinal loops of type V (instead of type I). The type species is *Paraperonia gondwanae* Labbé, 1934a, by subsequent designation (Starobogatov 1976: 211). Labbé’s description of *Paraperonia gondwanae* was based on 38 individuals with intestinal loops of types I and V which belong to different species. The application of the name *P. gondwanae* is clarified through the designation of a lectotype (see *P. verruculata*): *Paraperonia gondwanae* is a junior synonym of *Peronia verruculata*, and *Paraperonia* is a junior synonym of *Peronia*.

The generic name *Scaphis* was created by Labbé (1934a: 203) for nine species similar to *Peronia* but supposedly with an oblique, almost vertical hyponotum. The type species is *Onchidium astridae* Labbé, 1934b, by subsequent designation (Starobogatov 1976: 211). *Onchidium astridae* is a junior synonym of *Peronia verruculata*, and *Scaphis* is a junior synonym of *Peronia*.

*Lessonia* Labbé, 1934a is objectively invalid because it is the junior homonym of *Lessonia* Swainson, 1832 [Aves]. Starobogatov (1976: 211) replaced it by *Lessonina*. Labbé (1934a: 213–216, figs 48–50) described *Lessonia* based on a single species, *Onchidium ferrugineum* Lesson, 1831a, of which he examined no other material than the four syntypes (MNHN-IM-2000-22951). The examination of the three remaining syntypes (one syntype was lost by or after Labbé) revealed that the lectotype (Goulding et al. 2018b: 75) belongs to a *Peronia* species and that the two paralectotypes belong to *Wallaconchis ater* (Lesson, 1831a). Both Lesson’s original description of *Onchidium ferrugineum* and Labbé’s re-description of *Lessonina ferruginea* are a confusing combination of traits that characterize species from two distinct genera. For instance, the dorsal gills mentioned by both authors, are characteristic of *Peronia*, while the absence of an accessory penial gland mentioned by Labbé (even though there is a penial gland in the lectotype) is characteristic of *Wallaconchis*. Thanks to the designation of a lectotype with dorsal gills, the name *Onchidium ferrugineum* clearly applies to a *Peronia* species and *Lessonina* becomes a junior synonym of *Peronia*.

Starobogatov (1976: 211) created *Quoyella* as a replacement name of *Quoya* Labbé, 1934a, which he treated as a junior homonym of “*Quoya* Deshayes, 1843” [Mollusca, Gastropoda, Planaxidae]. In the second edition of Lamarck’s *Histoire naturelle des animaux sans vertèbres*, Deshayes indicates that he originally thought of creating a new genus *Quoya* but that, after all, he decided not to (Deshayes and Milne-Edwards 1845: 236). Deshayes still used the binomen “*Planaxis decollata* Quoy” (Deshayes and Milne-Edwards 1845: 238). However, in the *Explication des planches* of his *Traité élémentaire de conchyliologie*, Deshayes (1853: 50) used *Quoya* for two valid species names: *Quoya decollata* and *Quoya grateloupi*. Regardless, according to Gray (1847: 138), the generic name *Quoya* by Deshayes is an incorrect subsequent spelling of his *Quoyia* JE Gray, 1839. As an incorrect subsequent spelling, *Quoya* Deshayes is not available (ICZN 1999: Article 33.3) and, as a result, *Quoyella* is an unnecessary replacement name. Ironically, Gray (1847: 138) indicated that he originally found the generic name *Quoyia* in a manuscript by Deshayes in 1830 (“Quoyia, *Desh*. MSS. 1830; *Gray*, 1839 (...) Quoya, *Desh*. 1843”). According to Baker (1938: 87), *Quoya* Agassiz, 1862 [Coelenterata] is another homonym of *Quoya* Labbé, 1834a. However, the spelling of that generic name is not *Quoya* but *Quoyia* (Agassiz, 1862: 173). So, *Quoyia* Agassiz, 1862 is a junior homonym of *Quoyia* Gray, 1839, but *Quoya* Labbé, 1934a is not a junior homonym of *Quoyia*. *Quoya indica* Labbé, 1934a, type species of *Quoya* by monotypy, is regarded here as a *nomen dubium* even though it applies to a species with dorsal gills and thus belongs to *Peronia* (see general discussion).

Nothing is ever simple in onchidiid taxonomy. Indeed, Labbé (1935a, b) also described what he called “microgills” in *Elophilus* Labbé, 1935a, a name preoccupied by *Elophilus* Meigen, 1803 (Diptera) and replaced by *Labbella* Starobogatov, 1970. Labbé’s (1935a, b) microgills consolidated the old idea of a gradual continuum between regular dorsal papillae and dorsal gills. So, for instance, Marcus and Marcus (1960: 875) argued that one cannot say for sure whether a papilla is a dorsal gill or not. However, Dayrat et al. (2016, 2019d) demonstrated that there are no gills at all (not even microgills) on the notum of the type material of the type species of *Labbella* which actually belongs to *Onchidium stuxbergi* (Westerlund, 1883). *Labbella* is a junior synonym of *Onchidium*. Contrary to regular papillae, dorsal gills are distinctively branched, which is striking if specimens are fully relaxed before preservation but otherwise difficult to see. Finally, note that Labbé (1935b: 320) claimed that he observed rudimentary eyes on dorsal gills, which, to our knowledge, has never been confirmed.

Labbé (1934a: 187, 188) rightly recognized the importance of dorsal gills for classification and he separated all five genera of slugs with dorsal gills from all other onchidiids. According to Labbé, onchidiids deserved their own order, the Silicodermatae, composed of two suborders: Dendrobranchiatae (onchidiids with dorsal gills) and Abranchiatae (onchidiids without dorsal gills). Our phylogenetic analyses clearly demonstrate that all species of slugs with dorsal gills belong to a single clade, and that only one generic name (*Peronia*) is necessary (Figs 2–4). However, the species of slugs with no dorsal gills do not form a natural group (Figs 2–4). In other words, the absence of dorsal gills is a plesiomorphic trait for the onchidiids and the presence of dorsal gills is a synapomorphy for the genus *Peronia*.

Labbé’s (1934a: 187) distinction between the tribes Peroniidae (*Peronia* and *Paraperonia*) and Scaphidae (*Scaphis*, *Lessonina*, *Quoya*) based on the orientation of the hyponotum (horizontal versus oblique) is meaningless. This trait obviously varies depending on preservation, and Labbé exclusively studied preserved material from the collections of the MNHN without access to live animals.

Labbé’s (1934a: 187) distinction between *Peronia* and *Paraperonia* based on the intestinal types (type I in *Peronia* and type V in *Paraperonia*) is unwarranted because *Peronia* species with intestinal loops of type V are not more closely-related to each other (Table 4, Figs 2–4). Also, Labbé often made mistakes with respect to intestinal types: for instance, the type material of *Paraperonia gondwanae* includes individuals with loops of both types I and V, even though Labbé described it as a species with loops of type V. Labbé asserted that the position of the pneumostome and the size of the muscular sac differ between *Peronia* and *Paraperonia*. However, the position of the pneumostome varies between individuals and is not consistently on the right side of the median axis in species he classified as *Paraperonia*.

Labbé’s (1934a: 187) distinction between *Scaphis*, *Quoya*, and *Lessonina*, is also unwarranted. Again, the position of the pneumostome (on the right of a median line in *Scaphis* according to Labbé) varies between individuals. Labbé’s (1934a) re-description of *Lessonina ferruginea* (the type species of *Lessonina*, by monotypy) was based on individuals of two different species (see above). The male opening of the lectotype, which bears dorsal gills, is on the left of the right ocular tentacle, exactly as in all *Peronia* species, while the male opening of the two paralectotypes, which belong to *Wallaconchis ater*, is under the right ocular tentacle (Goulding et al. 2018: 75). Labbé (1934a: 216, fig. 51) described a double male opening in *Quoya indica* (the openings of the penis and of the accessory penial gland being supposedly separated), but this could not be confirmed in the type material. Regardless, male openings occasionally appear separated due to preservation (when the vestibule is everted) and that is by no means a trait of generic value.

Authors completely rejected Labbé’s (1934a) idea that the presence or absence of dorsal gills could be of any use in onchidiid classification (e.g., Marcus and Marcus 1960; Starobogatov 1976). Britton (1984: 180) even asserted that “the division of the group into two subordinate taxa based on this character is no longer admissible.” As for the status of Labbé’s (1934a, 1935a) generic names for slugs with dorsal gills, authors were not in agreement. Marcus and Marcus (1970: 213) regarded *Peronia* and *Paraperonia* “at most as subgenera.” Starobogatov (1976) regarded all names as valid: *Lessonina*, *Paraperonia*, *Peronia*, *Quoyella* (unnecessary replacement name for *Quoya*), *Scaphis*, and *Labbella* (supposedly with micro-gills). Britton (1984: 182–183) suggested that *Paraperonia*, *Eudrastus* and *Scaphis* should be regarded as junior synonyms of *Peronia* because they seemed to be based on “unimportant characters,” but treated *Labbella* (supposedly with micro-gills), *Lessonina*, and *Quoyella* (for *Quoya*) as valid. In a recent review of the application of onchidiid generic names, Dayrat et al. (2017: 1861) made it clear that all slugs with dorsal gills belong to one clade and that *Eudrastus*, *Lessonina*, *Onchis*, *Paraperonia*, *Peronia*, *Quoyella* (for *Quoya*), and *Scaphis* all refer to that clade. Note that the application of *Lessonina* was fully clarified when a lectotype was designated for its type species *Onchidium ferrugineum* (Goulding et al. 2018: 75).

##### Peronia peronii

Taxon classificationAnimaliaSystellommatophoraOnchidiidae

(Cuvier, 1804)

Figs 7 , 8 , 9 , 10 , 11 , 12 , 13 , 14 , 15 , 16

**Figure 7.**
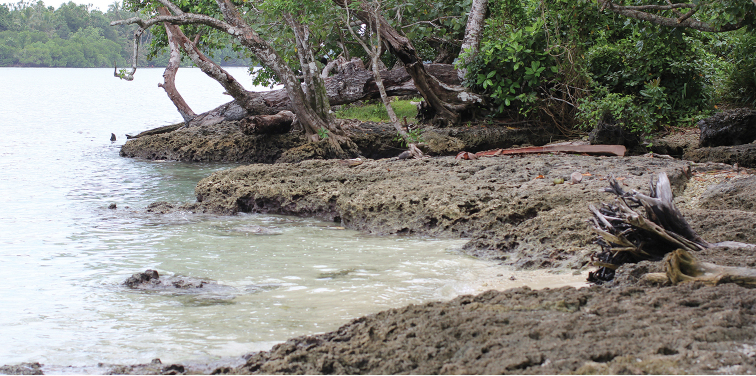
Habitat, *Peronia peronii*, Papua New Guinea, Madang, limestone rocky intertidal (st PM 12).

**Figure 8.**
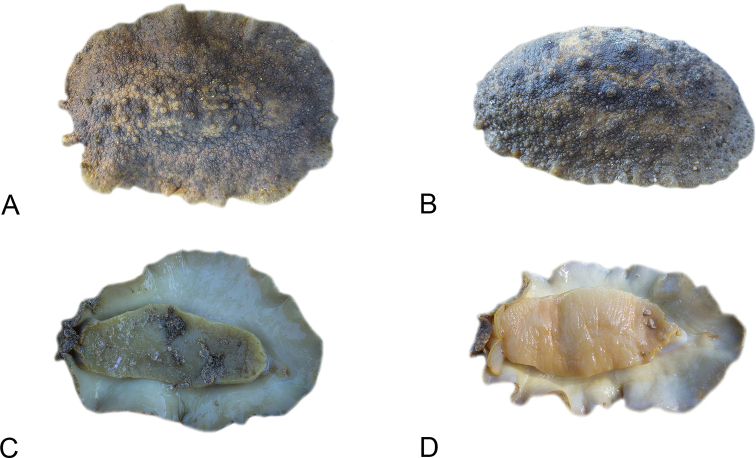
Live animals, *Peronia peronii*, Mauritius **A** dorsal view, 140 mm long [5872] (MNHN-IM-2019-1605) **B** dorsal view, 125 mm long [5874] (MNHN-IM-2019-1605) **C** ventral view, same as **A**; **D** ventral view, same as **B**.

**Figure 9.**
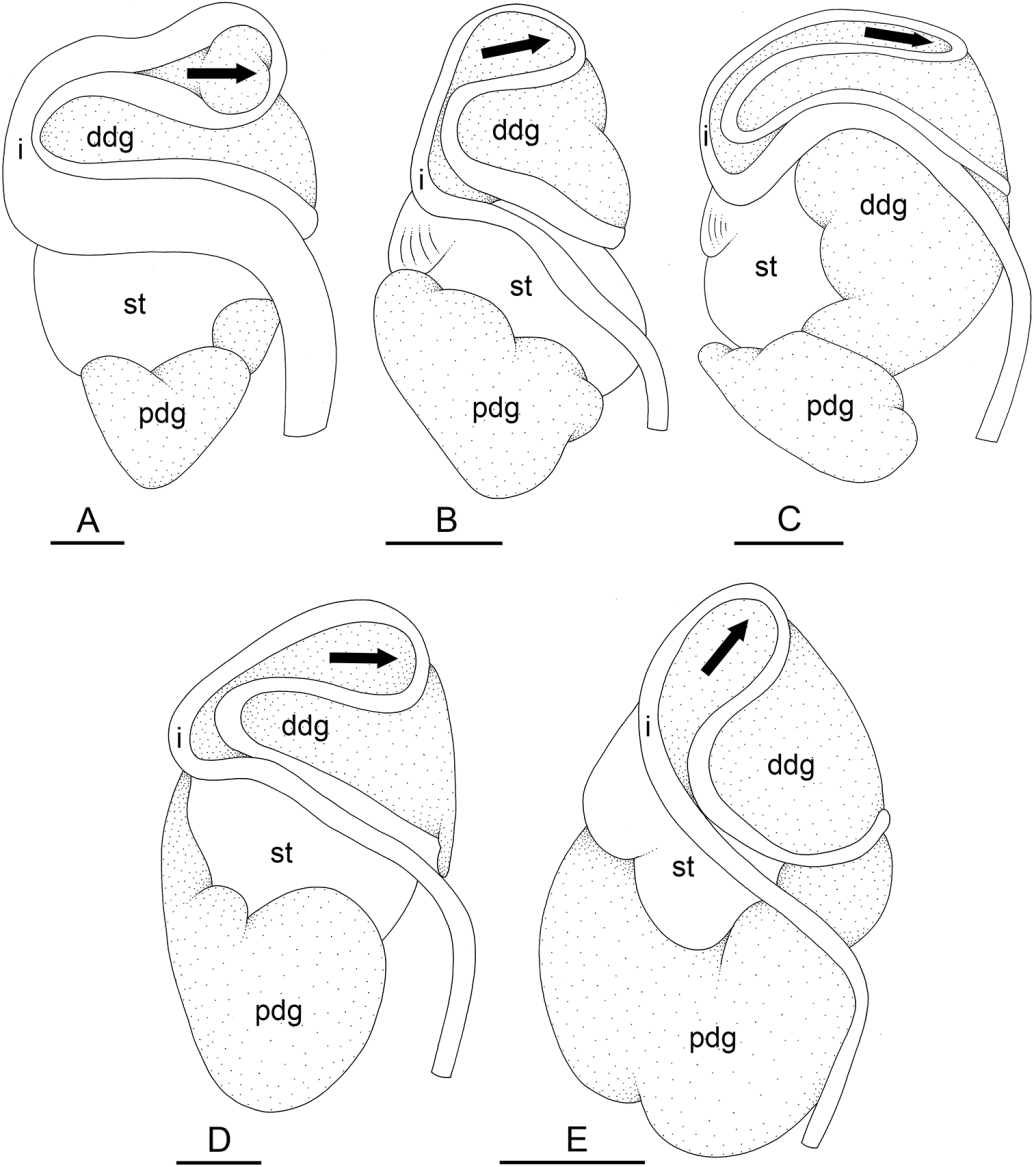
Digestive system, dorsal view, *Peronia peronii*, type specimens. The arrow indicates the orientation of the transitional loop **A** paralectotype, *Onchidium peronii*, Timor (MNHN-IM-2000-22938) **B** lectotype, *Onchidium tonganum*, Tonga (MNHN-IM-2000-22937) **C** lectotype, *Onchidium punctatum*, Indonesia, West Papua (MNHN-IM-2000-22966) **D** possible paralectotype, *Paraperonia gondwanae*, Mauritius (MNHN-IM-2000-33686) **E** lectotype, *Paraperonia fidjiensis*, Fiji (MNHN-IM-2000-33692). Scale bars: 5 mm (**A, D**), 10 mm (**B, C, E**). Abbreviations: ddg dorsal digestive gland, i intestine, pdg posterior digestive gland, st stomach.

**Figure 10.**
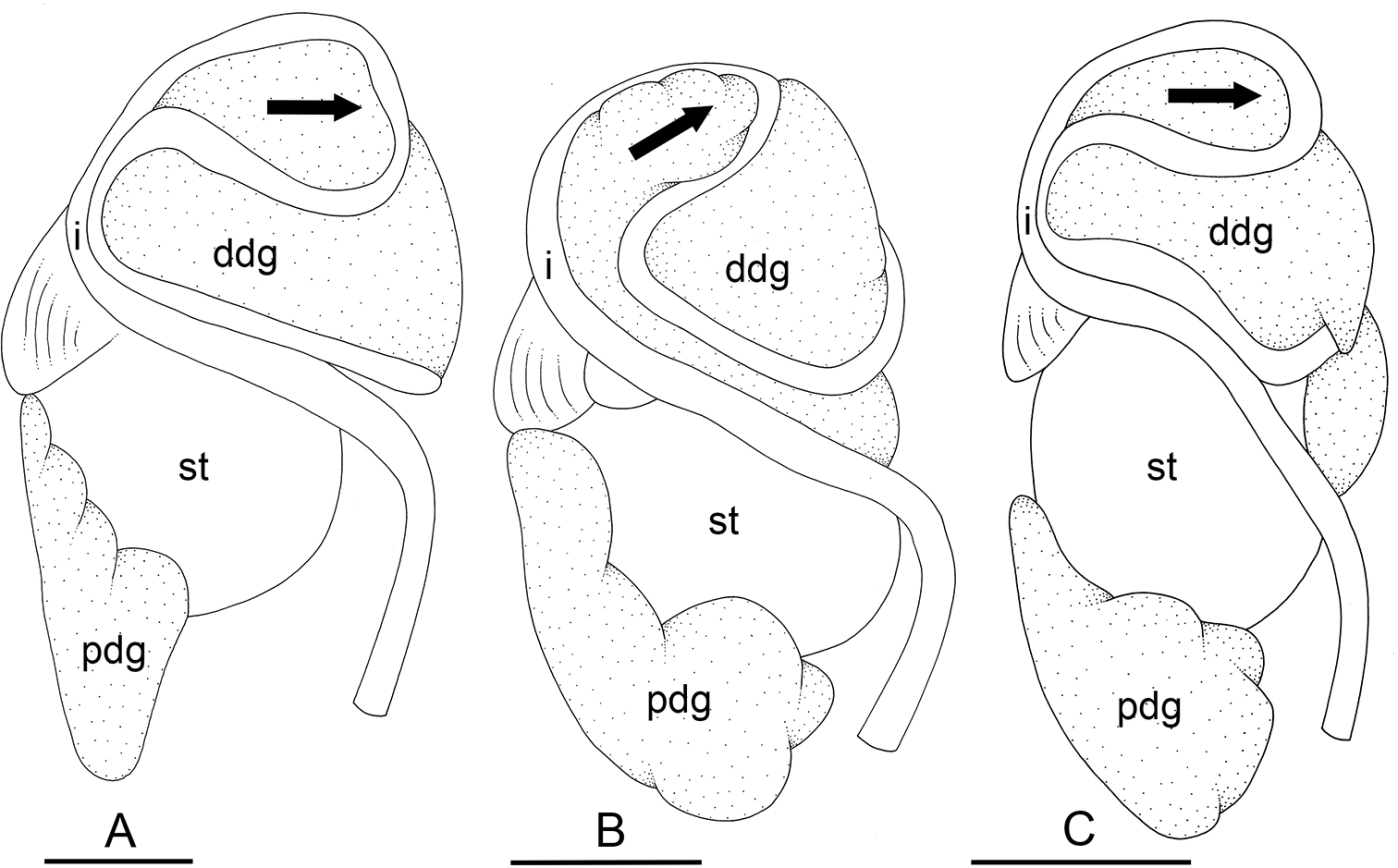
Digestive system, dorsal view, *Peronia peronii*. The arrow indicates the orientation of the transitional loop **A** Mauritius [5874] (MNHN-IM-2019-1605) **B** Papua New Guinea, Madang [5472] (MNHN-IM-2013-14052) **C** Guam [5840] (MNHN-IM-2019-1609). Scale bars: 10 mm (**A–C**). Abbreviations: ddg dorsal digestive gland, i intestine, pdg posterior digestive gland, st stomach.

**Figure 11.**
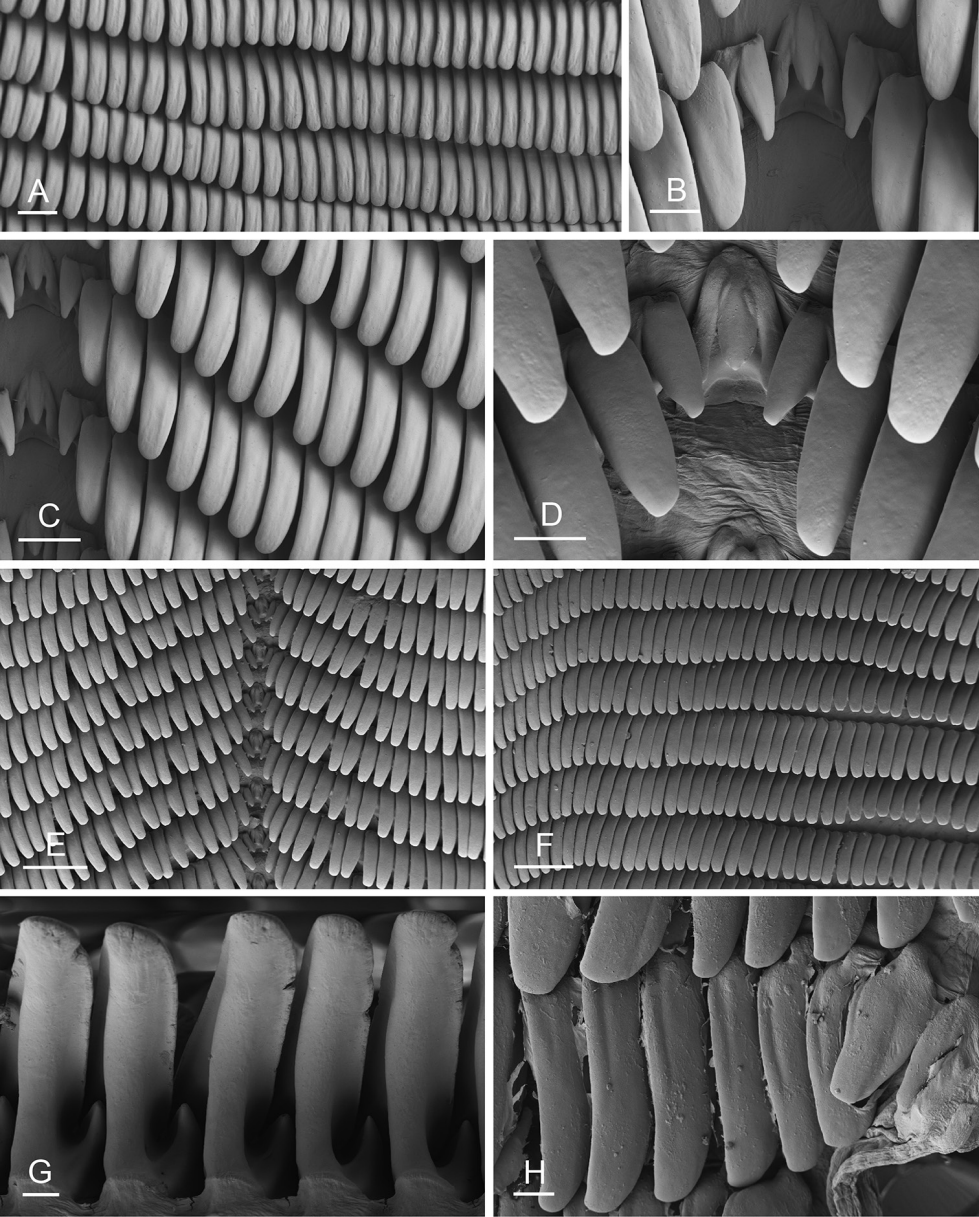
Radula, *Peronia peronii***A–C** Papua New Guinea [5476] (MNHN-IM-2013-16260) **D–H** Guam [5840] (MNHN-IM-2019-1609) **A** lateral teeth **B** rachidian and innermost lateral teeth **C** rachidian and innermost lateral teeth **D** rachidian and innermost lateral teeth **E** rachidian and lateral teeth **F** lateral teeth **G** lateral teeth, frontal view **H** outermost lateral teeth. Scale bars: 100 μm (**A**), 40 μm (**B, D**), 80 μm (**C**), 200 μm (**E, F**), 100 μm (**G, H**).

**Figure 12.**
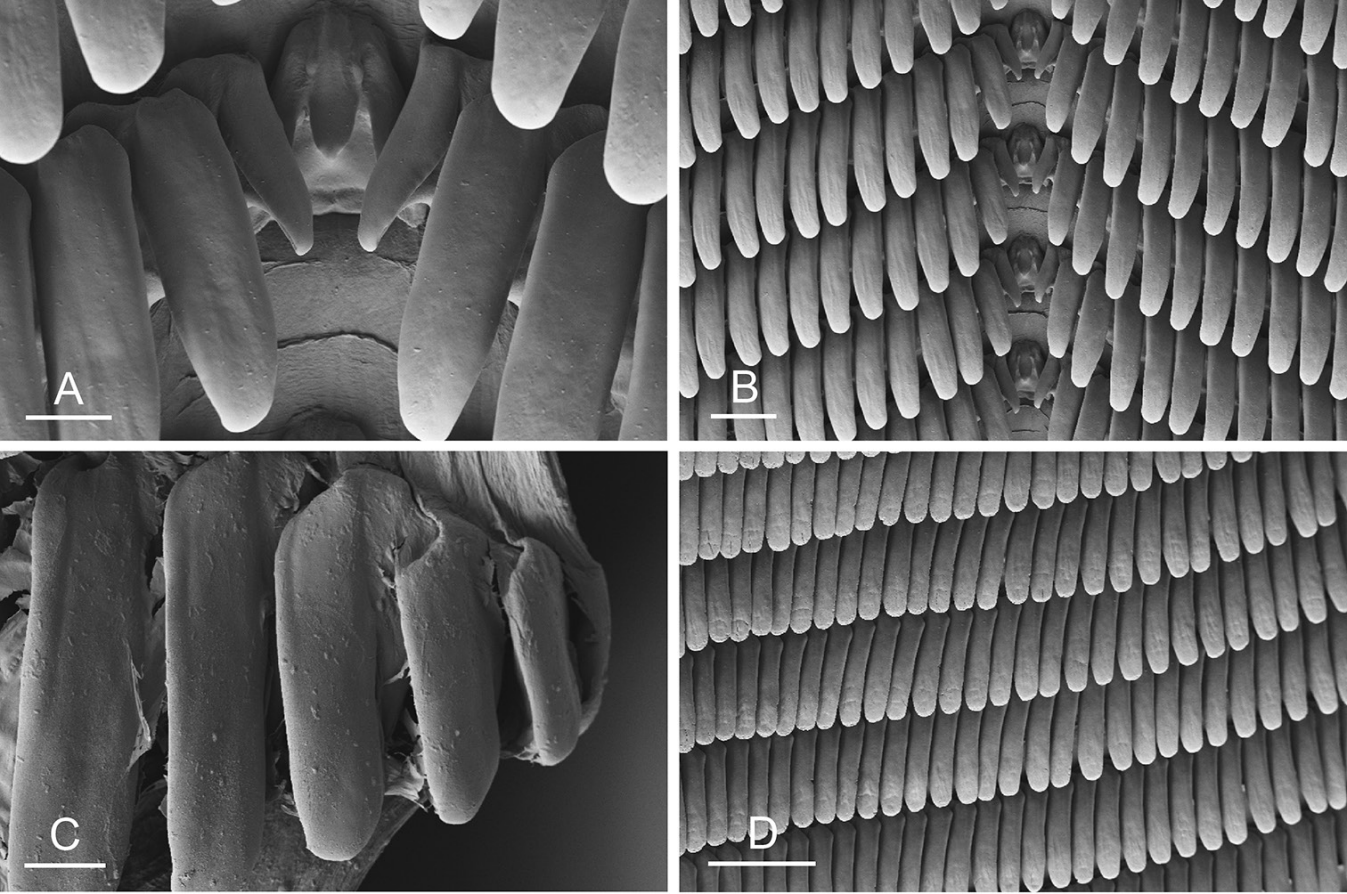
Radula, *Peronia peronii*, Mauritius [5872] (MNHN-IM-2019-1605) **A** rachidian and innermost lateral teeth **B** rachidian and lateral teeth **C** outermost lateral teeth **D** lateral teeth. Scale bars: 40 μm (**A**), 100 μm (**B**), 20 μm (**C**), 200 μm (**D**).

**Figure 13.**
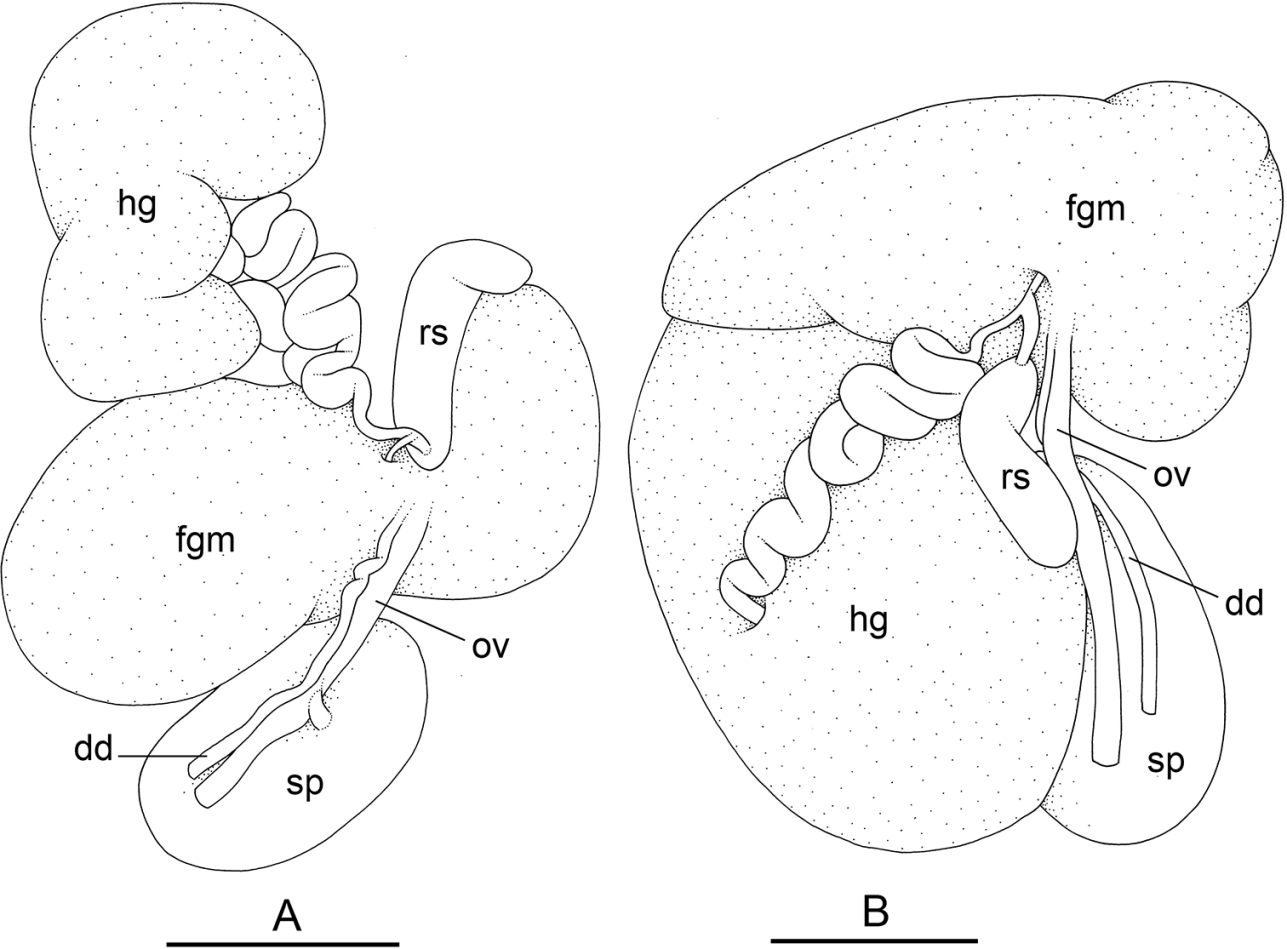
Posterior, hermaphroditic (female) reproductive system, *Peronia peronii***A** Mauritius [3605] (MNHN-IM-2019-1606) **B** Papua New Guinea, Madang [5472] (MNHN-IM-2013-14052). Scale bars: 10 mm (**A**), 5 mm (**B**). Abbreviations: dd deferent duct, fgm female gland mass, hg hermaphroditic gland, ov oviduct, rs receptaculum seminis, sp spermatheca.

**Figure 14.**
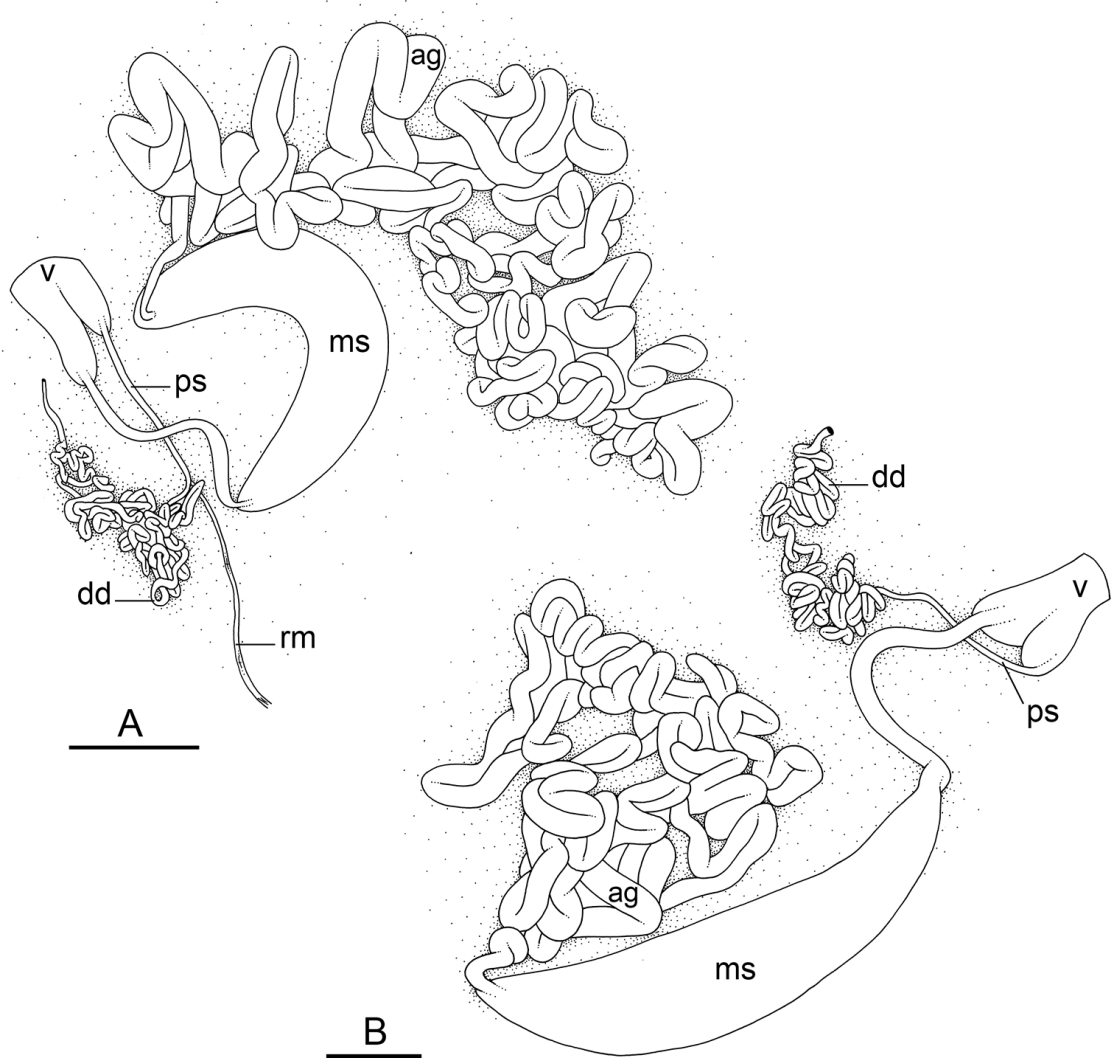
Anterior, male, copulatory apparatus, *Peronia peronii***A** Mauritius [3605] (MNHN-IM-2019-1606) **B** Papua New Guinea, Madang [5472] (MNHN-IM-2013-14052). Scale bars: 10 mm (**A**), 5 mm (**B**). Abbreviations: ag accessory penial gland, dd deferent duct, ms muscular sac, ps penial sheath, rm retractor muscle, v vestibule.

**Figure 15.**
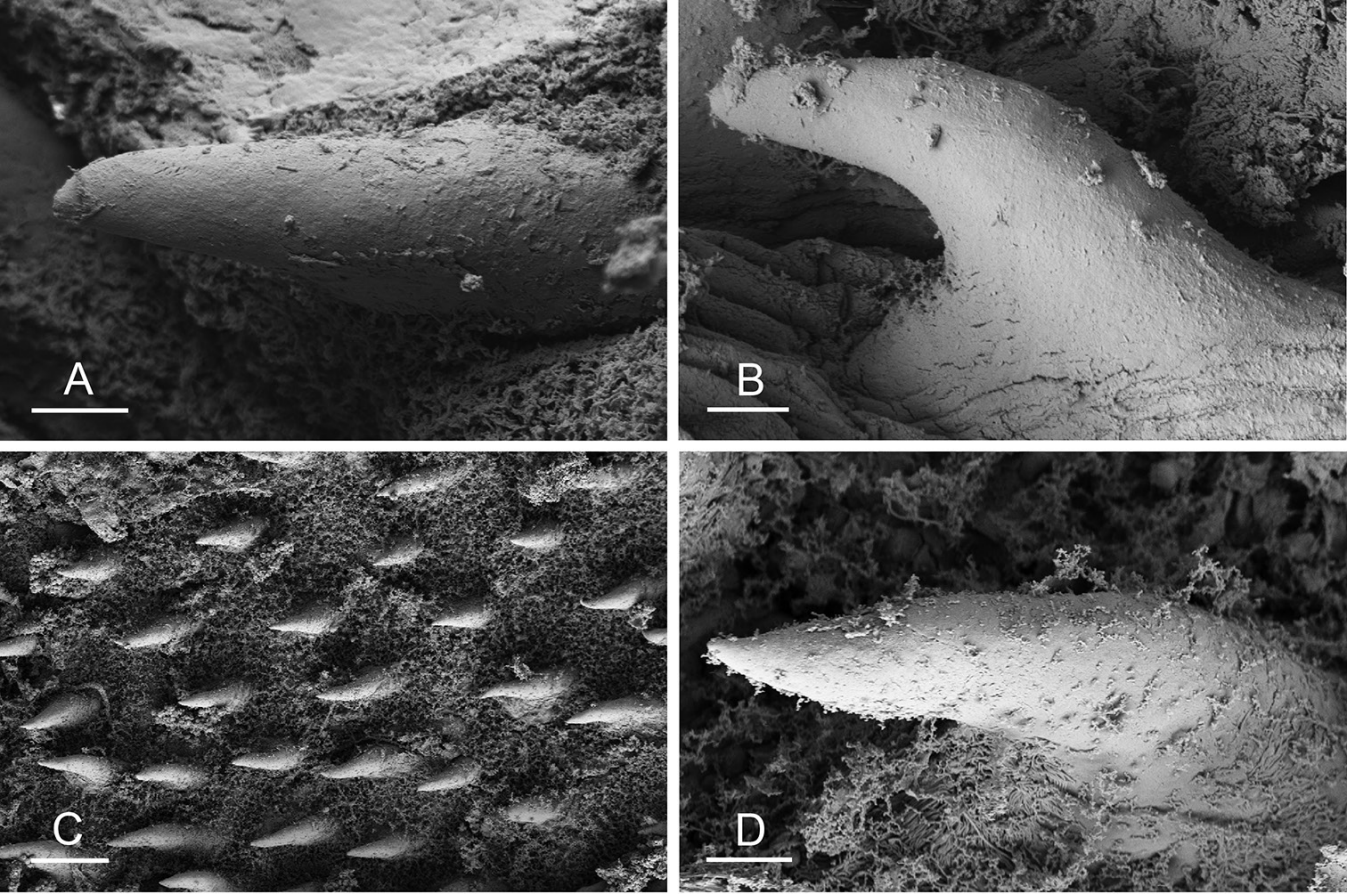
Penial hooks, *Peronia peronii***A** Mauritius [5872] (MNHN-IM-2019-1605) **B** Mauritius [3605] (MNHN-IM-2019-1606) **C, D** Guam [5840] (MNHN-IM-2019-1609). Scale bars: 6 μm (**A, B, D**), 40 μm (**C**).

**Figure 16.**
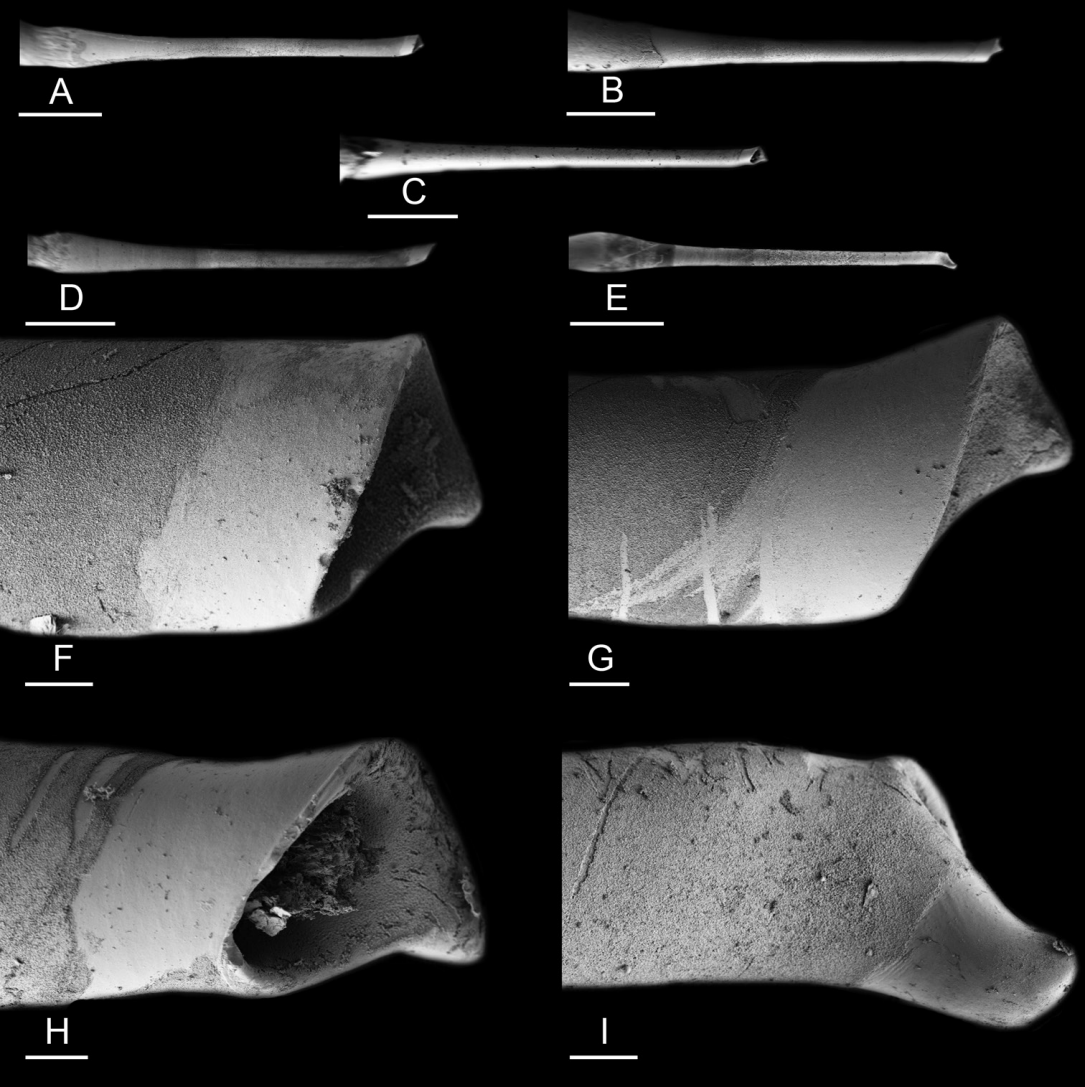
Accessory penial gland spine, *Peronia peronii***A, B, F, G** Mauritius **C, D, H** Papua New Guinea **E, I** Guam **A** [3605] (MNHN-IM-2019-1606) **B** [5872] (MNHN-IM-2019-1605) **C** [5471] (MNHN-IM-2013-12500) **D** [5472] (MNHN-IM-2013-14052) **E** [5840] (MNHN-IM-2019-1609) **F** same as **A**; **G** same as **B**; **H** same as **C**; **I** same as **E**. Scale bars: 800 μm (**A–D**), 1 mm (**E**), 40 μm (**F–I**).

**Figure 17.**
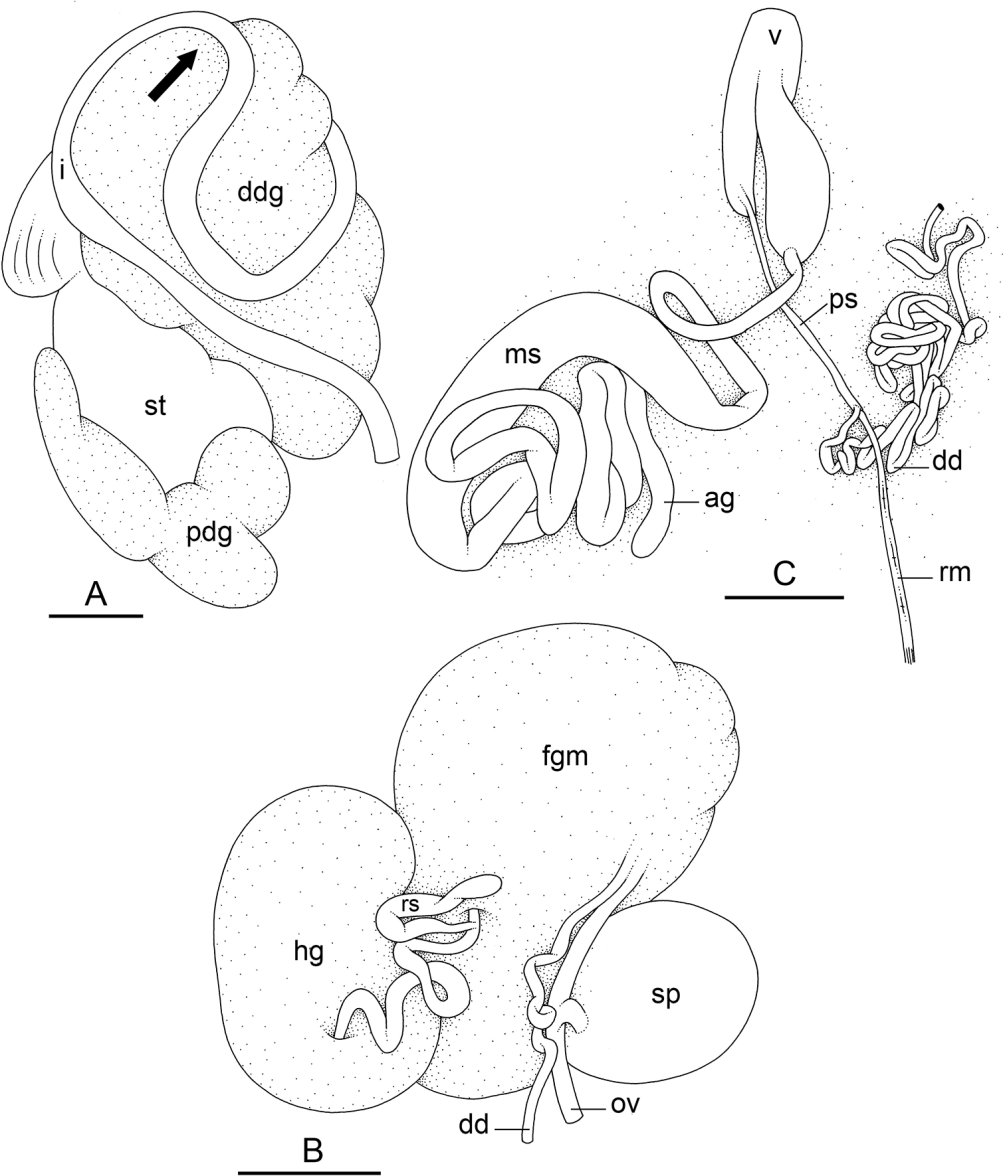
*Peronia okinawensis*, Japan, Okinawa, holotype [696-4 H] (UF 352288) **A** digestive system, dorsal view, the arrow indicates the orientation of the transitional loop **B** posterior, hermaphroditic (female) reproductive system **C** anterior, male, copulatory apparatus. Scale bars: 3 mm (**A–C**). Abbreviations: ag accessory penial gland, dd deferent duct, ddg dorsal digestive gland, fgm female gland mass, hg hermaphroditic gland, i intestine, ms muscular sac, ov oviduct, pdg posterior digestive gland, ps penial sheath, rm retractor muscle, rs receptaculum seminis, sp spermatheca, st stomach, v vestibule.

**Figure 18.**
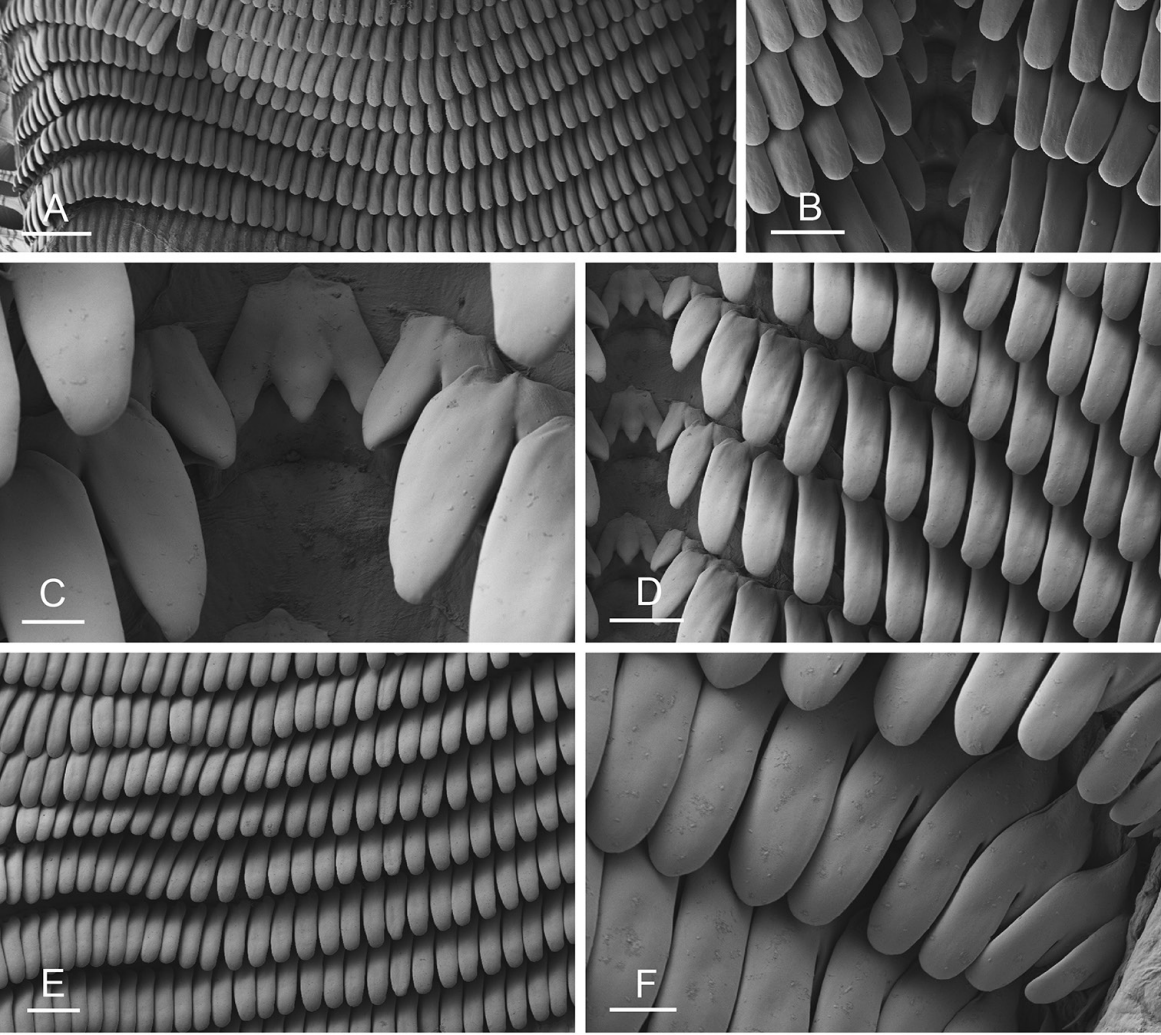
Radula, *Peronia okinawensis*, Japan, Okinawa **A, B** holotype [696-4 H] (UF 352288) **C–F** [696-2] (UF 352288) **A** left half rows of teeth **B** rachidian and innermost lateral teeth **C** rachidian and innermost lateral teeth **D** rachidian and lateral teeth **E** lateral teeth **F** outermost lateral teeth. Scale bars: 200 μm (**A**), 80 μm (**B**), 20 μm (**C, F**), 60 μm (**D**), 100 μm (**E**).

**Figure 19.**
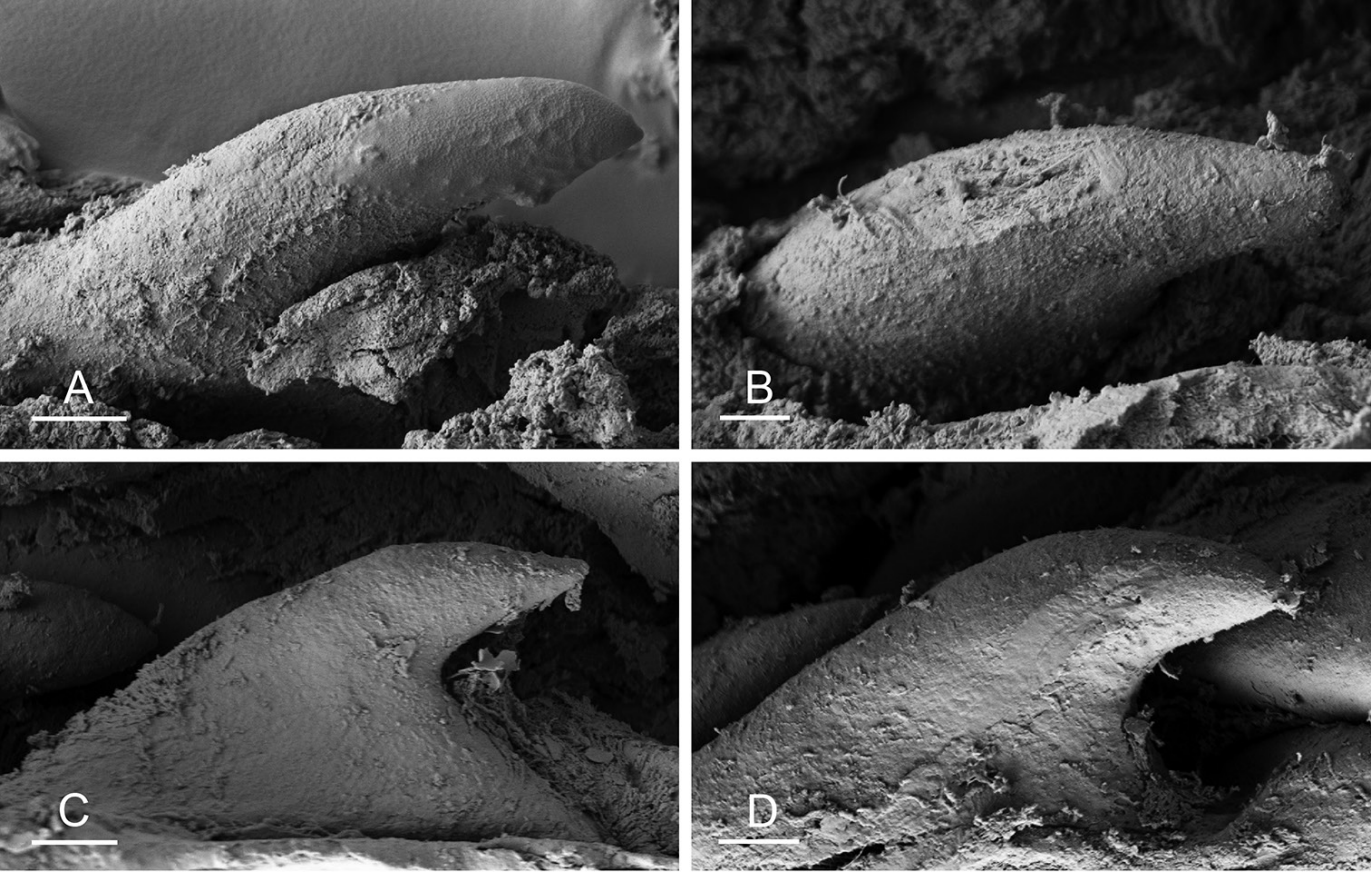
Penial hooks, *Peronia okinawensis*, Japan, Okinawa **A, B** holotype [696-4] (UF 352288) **C, D** [696-3] (UF 352288). Scale bars: 4 μm (**A, C, D**), 2 μm (**B**).

**Figure 20.**
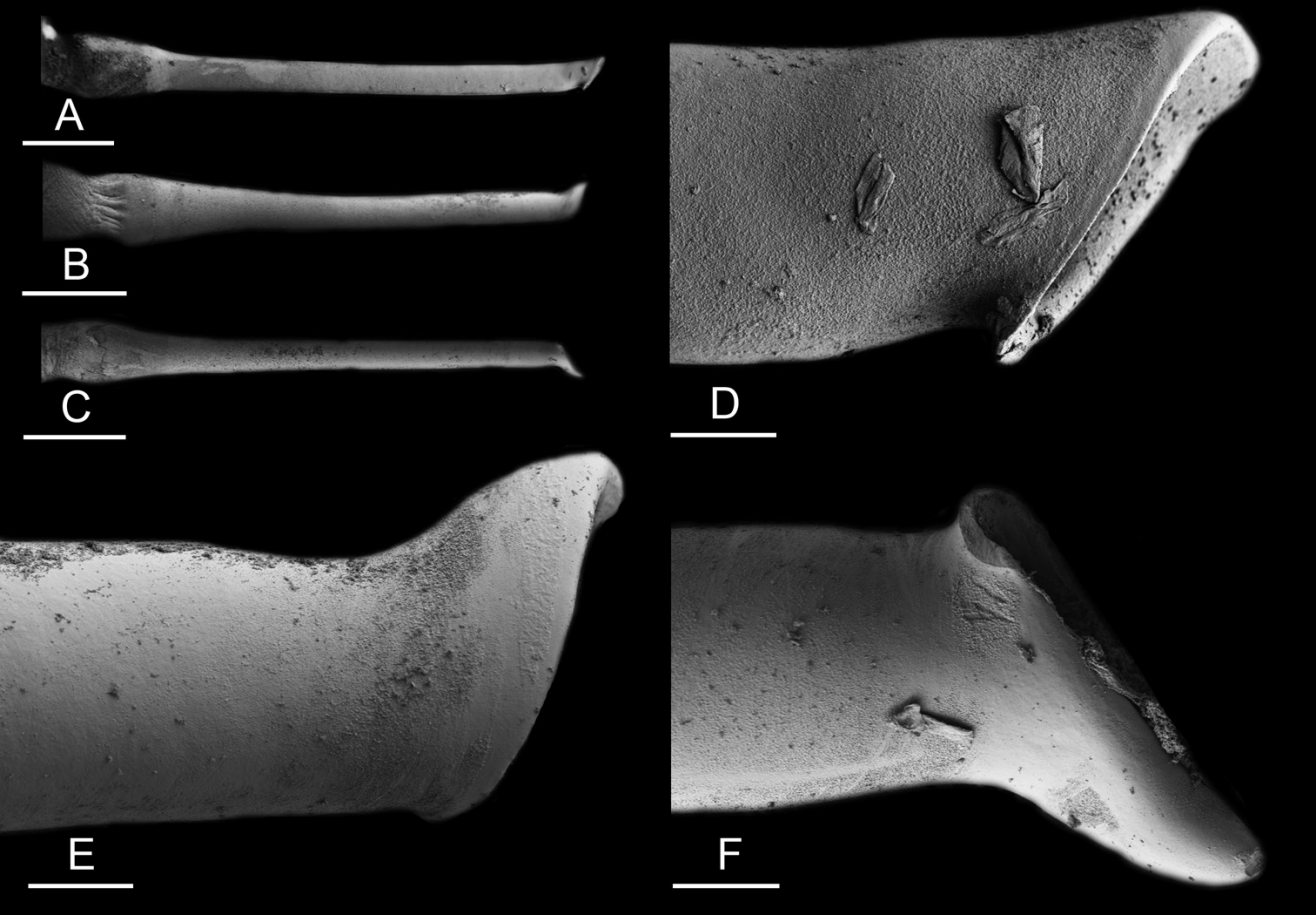
Accessory penial gland spine, Peronia okinawensis, Japan, Okinawa **A** holotype [696-4] (UF 352288) **B** [696-3] (UF 352288) **C** [696-2] (UF 352288) **D** same as **A**; **E** same as **B**; **F** same as **C**. Scale bars: 400 μm (**A–C**), 40 μm (**D–F**).

**Figure 21.**
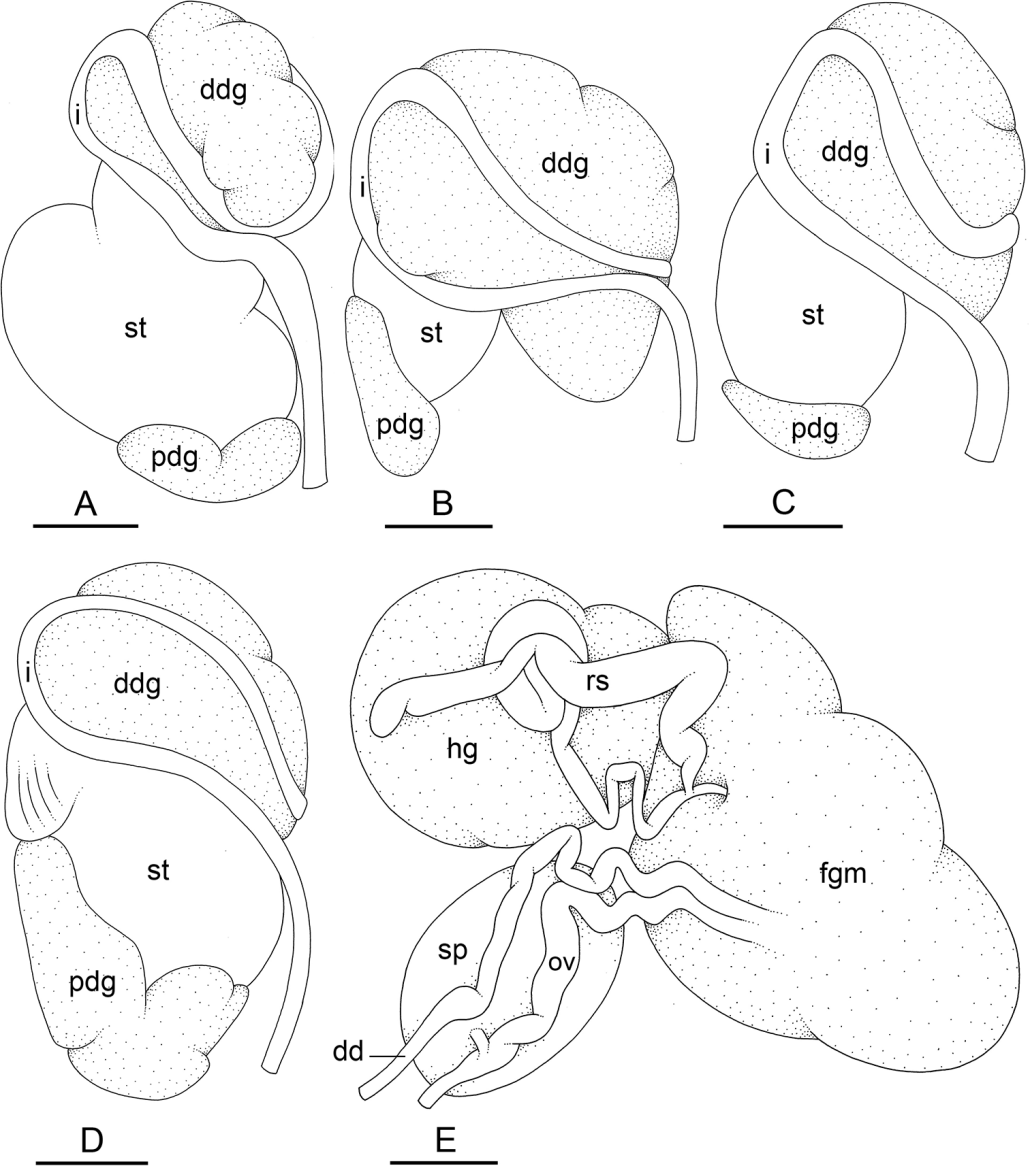
*Peronia madagascariensis***A–D** digestive system, dorsal view, with intestinal loops of type V **E** posterior, hermaphroditic (female) reproductive system **A** holotype, *Paraperonia madagascariensis*, Madagascar (MNHN-IM-2000-33680) **B** paralectotype, *Paraperonia gondwanae*, Mumbai, western India (MNHN-IM-2000-33682) **C** paralectotype, *Paraperonia gondwanae*, Red Sea (MNHN-IM-2000-33683) **D** Madagascar, [5501] (MNHN-IM-2009-16392) **E** same as D. Scale bars: 5 mm (**A–D**), 2 mm (**E**). Abbreviations: dd deferent duct, ddg dorsal digestive gland, fgm female gland mass, hg hermaphroditic gland, i intestine, ov oviduct, pdg posterior digestive gland, rs receptaculum seminis, sp spermatheca, st stomach.

Onchidium peronii Cuvier, 1804: 37–51, pl. 6, figs 1–9; Cuvier 1816: 411; Lamarck 1822: 46; Cuvier 1830: 46; Voigt 1834: 101; Deshayes 1836–1845: pl. 26, fig. 2; Deshayes and Milne-Edwards 1836: 709; JE Gray 1850: 117; ME Gray 1850: pl. 181, fig. 7; Berge 1855: 124, pl. 16, fig. 8; Plate 1893: 172–173, pl. 12, figs 85, 87, 91; Odhner 1919: 42; Hoffmann 1928: 44–45, 71–72 [in part only].
Peronia peronii (Cuvier, 1804): Fleming 1822a: 574; Fleming 1822b: 463; Keferstein 1865a: pl. CIII, fig. 1; Labbé 1934a: 190–191 [in part only]; Marcus and Marcus 1960: 877; Marcus and Marcus 1970: 213 [in part only]; Dayrat et al. 2011: 428; White et al. 2011: 4.
Onchis peronii (Cuvier, 1804): Férussac 1822: xxxi.
Peronia mauritiana Blainville, 1824: 281; Adams and Adams 1855: 235.
Onchidium tonganum Quoy & Gaimard, 1832: 210–211, pl. 15, figs 17, 18; Semper 1880: 258–260, pl. XIX, figs 2, 9, pl. XXII, figs 1, 2, 10 [in part only]; Bergh 1884a: 142–148, pl. VI, fig. 19, pl. VII, figs 1–6.
Peronia tongana (Quoy & Gaimard, 1832): Oken 1834a: 287; JE Gray 1850: 117; ME Gray, 1850: pl. 182, fig. 1, as tongensis; Adams and Adams 1855: 235, pl. LXXXI, fig. 3; Keferstein 1865a: pl. CII, fig. 20; Tapparone Canefri 1883: 214 [in part only]; Labbé 1934a: 191–192, figs 4–7 [in part only].
Onchidium punctatum Quoy & Gaimard, 1832: 215–216, pl. 15, figs 27, 28. Syn. nov.
Peronia punctata (Quoy & Gaimard, 1832): Oken 1834a: 287; JE Gray 1850: 117; ME Gray 1850: pl. 183, fig. 3; Adams and Adams 1855: 235; Chenu 1859: 474, fig. 3505; Tapparone Canefri 1883: 214.
Onchidium melanopneumon Bergh, 1884a: 129–142, pl. IV, figs 25–27, pl. V, figs 1–27, pl. VI, figs 5–18, 20–21; Joyeux-Laffuie 1885: viii–xi.
Paraperonia fidjiensis Labbé, 1934a: 197–198, figs 9–11. Syn. nov.
Peronia verruculata : Mörch 1872a: 28; Mörch 1872b: 325, as vermiculata [non Peronia verruculata (Cuvier, 1830)].

###### Type material.

***Lectotype* and *paralectotype*** (*Onchidium peronii*). Mauritius • lectotype, hereby designated, by means of Cuvier’s (1804: pl. 6) anatomical drawings. Timor • 1 paralectotype, 60/40 mm; F Péron leg.; MNHN-IM-2000-22938. The fact that the specimen illustrated by Cuvier cannot be located does not invalidate the lectotype designation (ICZN 1999: Article 74.4). That individual, according to Cuvier, measured approximately 140 mm long (preserved). Cuvier’s (1804: pl. 6) detailed anatomical drawings are exclusively based on the individual collected by Péron in Mauritius. Note that, although Cuvier’s (1804: pl. 6) illustrations are truly remarkable, they are flipped at 180° because, for instance, the heart and the male anterior parts are on the left. Something must have happened during the engraving or the printing. Hoffmann (1928: 71) referred to Mauritius as the “Typ-Lokalität” of *Onchidium peronii* but did not formally designate a lectotype for *O. peronii*. In case of syntypes, “the place of origin of the lectotype becomes the type locality of the nominal species-group taxon, despite any previously published statement of the type locality.” (ICZN 1999: Article 76.2)

The original description of *Onchidium peronii* was based on two specimens collected by Péron: the lectotype from Mauritius, of which the internal anatomy was illustrated in detail by Cuvier (1804: pl. 6), could not be located and is likely lost; the paralectotype from Timor (MNHN-IM-2000-22938) was very briefly mentioned by Cuvier (1804: 39) who merely wrote that another specimen was brought from Timor by Péron and that *Onchidium peronii* is present “at the two extreme ends of the Indian Ocean.” The paralectotype (60/40 mm) is well preserved even though dorsal papillae with eyes cannot be counted because their color faded. It is obvious that Cuvier did not actually use it for his detailed anatomical description and illustrations on plate 6, because it was never opened prior to the present study, except for a tiny cut near the lung. It was carefully opened on its side to draw a dorsal view of its intestinal loops of type I (Fig. 9A) and measure the length (4.5 mm) of the spine of the accessory penial gland (by transparency, so that the male copulatory apparatus was not dissected).

***Lectotype*** (*Peronia mauritiana*). Mauritius • lectotype, hereby designated, by means of Cuvier’s (1804: pl. 6) anatomical drawings. The species name *Peronia mauritiana* was introduced by Blainville (1824: 281) for a species originally illustrated by Cuvier (1804: pl. 6) in the *Annales du Muséum d’Histoire naturelle* and which Cuvier named *Onchidium peronii*. Blainville’s reference to Cuvier’s (1804) plate 6 (“La Péronie de l’Isle-de-France [Mauritius]. *Peronia mauritiana*. Blainv., Cuv., Ann. du Mus., 5, pl. 6.”) serves as an indication, and *Peronia mauritiana* is an available binomen (ICZN 1999: Article 12.2.1). However, Cuvier’s original description of *Onchidium peronii* was based on two specimens, a lectotype from Mauritius and a paralectotype from Timor, but Cuvier’s (1804: pl. 6) plate of anatomical drawings exclusively illustrates the lectotype from Mauritius (see above). Because the lectotype of *Peronia mauritiana* also is the lectotype of *Onchidium peronii*, *Peronia mauritiana* remains what it always was, i.e., a junior objective synonym of *Onchidium peronii*.

Blainville also mentioned the name *Peronia mauritiana* in his *Manuel de Malacologie et de Conchyliologie* (Blainville 1825: 490) and in the article “Péronie” of the *Dictionnaire des Sciences Naturelles* (Blainville 1826: 523). The illustration published by Blainville (1827: pl. 46, fig. 7) in the *Atlas* of the *Manuel* differs from that published by Cuvier (1804: pl. 6, fig. 1). The specimen used by Blainville for that illustration could not be located, which does not matter much since it does not have any name-bearing function. However, it also means that, because there are two species of *Peronia* in Mauritius, Blainville’s (1827: pl. 46, fig. 7) illustration may or may not refer to *Peronia mauritiana*.

***Lectotype*** (*Onchidium tonganum*). Tonga • lectotype, hereby designated, 100/60 mm; Panhi-Motou [possibly the small island of Pangaimotu]; MNHN-IM-2000-22937. It is unclear how many specimens Quoy and Gaimard (1832: 210–211, pl. 15, figs 17, 18) examined for the original description of *Onchidium tonganum*. They may have examined more than one individual. Regardless, it is clear that *Onchidium tonganum* applies to a *Peronia* species because the notum of the lectotype bears gills which were also illustrated in the original description. Its notum also bears fifteen dorsal papillae with eyes but others probably faded. The lectotype was dissected prior to the present study. The accessory penial gland and the penial apparatus are missing (pieces of the deferent duct remain). The intestinal loops are of type I with a transitional loop between 2 and 3 o’clock (Fig. 9B). Quoy and Gaimard (1832: 216) briefly mentioned the presence of *O. tonganum* in Manokwari, West Papua, Indonesia, but that record could not be confirmed (although *P. peronii* is known to be present there because Manokwari is the type locality of *O. punctatum*).

***Lectotype* and *paralectotypes*** (*Onchidium punctatum*). Indonesia • lectotype, hereby designated, 70/60 mm; dans le port de Dorey [Manokwari harbor, West Papua]; 1829; JRC Quoy and JP Gaimard leg.; MNHN-IM-2000-22966. • 2 paralectotypes, 35/25 and 32/30 mm; same collection data as for the lectotype; MNHN-IM-2000-33701. An old label of the lectotype says “Onchidium punctatum, Q. G, Ast. pl. 15, fig. 27, de la Nouvelle Guinée, Quoy et Gaimard 1829.” That old label does not say “Dorey” (for the locality), which is only mentioned in the original description, but it clearly indicates that the lectotype was part of the type series of *Onchidium punctatum*. The lectotype bears dorsal gills, as illustrated by Quoy and Gaimard (1832: pl. 15, figs 27, 28). It was dissected prior to the present study, likely by Labbé (1934a: 203–204) and its penis is missing but its intestinal loops are of type I with a transitional loop at 3 o’clock (Fig. 9C). Its spine of the accessory penial gland, still in place in the animal, is 3.7 mm long.

A second jar was found with two paralectotypes (MNHN-IM-2000-33701). An old label for that second jar says “Onchidium piquetée, Q G. MM Quoy Gaimard, 1829” with no locality data. The name “Peronia” was added on the label. The number “51” also appears on another old label, which corresponds to an unknown numbering system. There also is a more recent label saying “*Peronia picta* QG, M. Quoy et Gaimard, 1829.” Quoy and Gaimard did not describe any onchidiid species with the specific name *picta*. However, the French vernacular name of *Onchidium punctatum* in Quoy and Gaimard’s (1832: 215) original description is “Onchidium piquetée.” So, it is likely that these two additional specimens were part of the type series of *Onchidium punctatum*. Both paralectotypes (35/25 and 32/30 mm) bear dorsal gills. The largest paralectotype was dissected prior to the present study, possibly by Labbé (1934a: 203, 204), and its penis is missing but its accessory penial gland remains. The small paralectotype was not dissected. Labbé (1934a: 203) listed three individuals from Port-Dorey which he (implicitly) regarded as part of the original series of *Onchidium punctatum*. Labbé gave the measurements for only two individuals: “*a*” (35/25 mm), likely the largest paralectotype; “*b*” (77/56 mm), likely the lectotype. In addition, in his re-description of *Scaphis punctata*, Labbé (1934a: 204–205) mentioned two individuals identified as *Peronia* and collected by Quoy and Gaimard in 1829, from an unknown locality. Those two individuals are likely within another jar found at the MNHN with the old number “48” and a label saying “Peronia M. Quoy et Gaimard 1829.” There is no reason to consider that those two unidentified individuals from the collection of Quoy and Gaimard were part of the type series of *Onchidium punctatum*. Finally, there is no other old material at the MNHN which could be assigned to the type series of *O. punctatum*. There are only three other old specimens from Port Dorey at the MNHN: the two syntypes (MNHN-IM-2000-22950) of *Wallaconchis ater* (Goulding et al. 2018: 63), and one specimen collected by Raffray in 1878 (with numbers “22” and “75” on the label).

***Holotype*** (*Onchidium melanopneumon*). Fiji • holotype, by monotypy, 65/40 mm; Kandavu [Kadavu]; Aug 1874; HMS Challenger leg.; NHMUK 1888.5.30.39. The holotype was entirely dissected by Bergh and is now empty. Given the presence of dorsal gills, *Onchidium melanopneumon* clearly applies to a *Peronia* species.

***Lectotype* and *paralectotypes*** (*Paraperonia fidjiensis*). Fiji • lectotype, hereby designated, 60/50 mm; 1876; Filhol leg.; MNHN-IM-2000-33692. No jar clearly labeled as the type material of *Paraperonia fidjiensis* was found at the MNHN, but the lectotype could be traced, and six paralectotypes could not be found at the MNHN. Labbé (1934a: 197–198, figs 9–11) described *Paraperonia fidjiensis* based on seven individuals from Fiji (“Iles Fidji”) collected by Filhol (Henri Filhol [1843–1902]) in 1876 and with the following sizes: 75/50 mm for six “*a*” individuals and 70/50 mm for a seventh “*b*” individual. Two jars of material collected in Fiji by Filhol in 1876 were found at the MNHN. The first jar, labeled as “Peronia [written over Oncidium] I. Fidji M^r^. Filhol n°11 1876” and “71,” contains a single *Peronia* specimen which, given its size (60/50 mm), very likely is part of the type series of *P. fidjiensis*, and which is designated as the lectotype (MNHN-IM-2000-33692). Its radula and all reproductive parts are missing. Its intestinal loops are clearly of type I, with a transitional loop at ~ 1 o’clock (Fig. 9E). The second jar, labeled as “Oncidiella I. Fidji M^r^. Filhol n°11 1876” and “101,” contains four poorly-preserved specimens which do not even appear to belong to *Peronia*, with a size (less than 30 mm) not compatible with the original description of *P. fidjiensis*, and which, therefore, cannot be regarded as part of the type series.

###### Additional material examined.

Mauritius • 2 specimens 140/100 mm [5872] and 125/75 mm [5874]; La Mivoie; 20°20.659'S, 57°21.763'E; 11 Jun 2014; TC Goulding leg.; st 177, basalt rocks, at night; MNHN-IM-2019-1605. • 1 specimen 110/100 mm [3605]; Mahebourg, waterfront; 20°24.317'S, 57°42.605'E; 13 Jun 2014; TC Goulding leg.; st 178, rocky intertidal, with algae, just before sunrise; MNHN-IM-2019-1606. • 1 specimen 100/90 mm [1553]; Grand Port, east side of île Marianne; 20°22.828'S, 57°47.220'E; May 2003; O Griffiths leg.; A2518, out of water on limestone platform; MNHN-IM-2019-1607.

Mariana Islands • 1 specimen 115/80 mm [443]; Guam Island, Bile Bay; 13°17.124'N, 144°39.742'E; 23 Mar 2007; C Carlson leg.; reef margin; CASIZ 180486. • 1 specimen 85/70 mm [5840]; Guam Island, Bile Bay; 13°16.582'N, 144°39.752'E; 27 Nov 2007; C Carlson leg.; shoreline; MNHN-IM-2019-1609.

Papua New Guinea – **Madang** • 1 specimen 70/60 mm [5476]; Wonad Island; 05°08.1'S, 145°49.3'E; 29 Nov 2012; MNHN Expedition Papua Niugini leg.; st PM43, night tide, sandy beach and intertidal rocks; MNHN-IM-2013-16260. • 1 specimen 65/45 mm [5477]; Wonad Island; 05°08.1'S, 145°49.3'E; 27 Nov & 09 Dec 2012; MNHN Expedition Papua Niugini leg.; st PM41, sandy beach and intertidal rocks; MNHN-IM-2013-15872. • 1 specimen 55/40 mm [5474]; Rempi Area, Barag Island; 05°01.1'S, 145°47.9'E; 15 Nov 2012; MNHN Expedition Papua Niugini leg.; st PM25, fringing reef on narrow barrier island; MNHN-IM-2013-14054. • 1 specimen 80/60 mm [5472]; same collection data as for the preceding; MNHN-IM-2013-14052. • 1 specimen 80/70 mm [5471]; Rempi Area, South Dumduman Island; 05°00.2'S, 145°47.6'E; 9 Nov 2012; MNHN Expedition Papua Niugini leg.; st PM 12, limestone rocky intertidal; MNHN-IM-2013-12500. – **New Ireland** • 1 specimen 50/40 mm [6086]; Kavieng, Lemus Island; 02°38'S, 150°37.5'E; 12–14 Jun 2014; MNHN Expedition Kavieng 2014 leg.; st KM24, mixed platform with seagrass; MNHN-IM-2013-53482.

###### Additional material examined

**(historical museum collections).** Chagos Archipelago • 1 specimen 95/65 mm; Ye Ye, Peros Banhos atoll; 24 Feb 1996; M Spalding (from N Yonow’s personal collection) leg.; exposed on shallow reef flat on rocks; MNHN-IM-2014-7992.

Fiji • 1 specimen 75/50 mm; Viti Isles; A Garrett leg.; ANSP 57967. • 1 specimen 28/25 mm; Viti Levu, Namuka; 18°08'S, 177°23'E; 18 Apr 1917; S Bock’s Pacific Expedition 1917–1918 leg.; barrier reef; SMNH 180357. • 2 specimens 23/20 mm and 15/15 mm; Viti Levu, SW Suva, Namuka; 18°08'S, 177°23'E; 16 Jun 1917; S Bock’s Pacific Expedition 1917–1918 leg.; barrier reef; SMNH 180374. • 1 specimen 37/30 mm; Viti Levu, Namuka; 18°08'S, 177°23'E; 19 Jun 1917; S Bock’s Pacific Expedition 1917–1918 leg.; SMNH 180375. • 1 specimen 80/65 mm; Viti Levu, Bau Island; 17°58'S, 178°36'E; 2 Jul 1917; S Bock’s Pacific Expedition 1917–1918 leg.; reef; SMNH 180373.

India • 2 specimens 85/55 mm and 70/50 mm; Nicobar Islands, Pulo Milo, Little Nicobar; Reinhardt, Galathea 305 leg.; NHMD 613753.

Indonesia – **Java** • 1 specimen 90/55 mm; Batavia [Jakarta]; 1899; C Aurivillius leg.; SMNH 180355. – **Sumatra** • 1 specimen 100/70 mm; Sumatra; Deshayes leg.; MNHN-IM-2012-25150. • 1 specimen 65/50 mm; west coast of Sumatra, Pulo Pasu [or Pulu Pasu]; 1891; C Aurivillius leg.; SMNH 180354. – **Tanimbar** • 1 specimen 60/50 mm; Jamdena Straits, West side of Mitak Island; 07°11'S, 131°28'E; 22 Jun 1970; Mariel King Memorial Expedition Moluccas MV “Pele” 1970 leg.; WAM S26723.

Kiribati • 1 specimen 35/35 mm; Gilbert Islands, Apaiang [Abaiang]; 01°49'N, 172°57'E; 12 Aug 1917; S Bock’s Pacific Expedition 1917–1918 leg.; outer reef; SMNH 180353. • 1 specimen 70/65 mm; Gilbert Islands, Tarawa; 01°26'N, 173E; 16–20 Aug 1917; S Bock’s Pacific Expedition 1917–1918 leg.; reef; SMNH 180382. • 1 specimen 65/50 mm; Gilbert Islands, Aranuka; 00N, 174E; 6 Oct 1917; S Bock’s Pacific Expedition 1917–1918 leg.; reef; SMNH 180376. • 1 specimen 70/50 mm; Gilbert Islands, Aranuka; 00N, 174E; 22 Oct 1917; S Bock’s Pacific Expedition 1917–1918 leg.; outer reef; SMNH 180377. • 1 specimen 30/25 mm; Gilbert Islands, Aranuka; 00N, 174E; 1 Nov 1917; S Bock’s Pacific Expedition 1917–1918 leg.; SMNH 180378. • 1 specimen 15/15 mm; Gilbert Islands, Aranuka; 00N, 174E; 26 Oct 1917; S Bock’s Pacific Expedition 1917–1918 leg.; outer reef; SMNH 180383. • 2 specimens 20/15 mm and 17/13 mm; Gilbert Islands, Aranuka; 00N, 174E; 26 Oct 1917; S Bock’s Pacific Expedition 1917–1918 leg.; outer reef east; SMNH 180384. • 1 specimen 15/12 mm; Gilbert Islands, Aranuka; 00°09'N, 173°35'E; 1917; S Bock’s Pacific Expedition 1917–1918 leg.; outer reef; SMNH 180478. • 1 specimen 65/50 mm; Gilbert Islands; Oct 1917; S Bock’s Pacific Expedition 1917–1918 leg.; outer reef; SMNH 180475. • 1 specimen 80/65 mm; Gilbert Islands, Apamama [Abemama]; 00°24'N, 173°55'E; 1917; S Bock’s Pacific Expedition 1917–1918 leg.; entrance reef; SMNH 180380. • 1 specimen 45/35 mm; Gilbert Islands, Apamama [Abemama]; 00N, 173E; 1917; S Bock’s Pacific Expedition 1917–1918 leg.; at low tide; SMNH 180379.

Madagascar • 1 specimen 65/50 mm; Tulear [Toliara]; 23°22'S, 43°39'E; Feb 1913; K Afzelius leg.; coral reef; SMNH 180381.

Maldive Islands • 1 specimen 85/55 mm; Tiladummati Atoll, Faro Islet, on reef NW of Fildau Island; 06°55.333'S, 73°11.833'E; 30 & 31 Mar 1964; R Robertson, International Indian Ocean Expedition leg.; st R021, intertidal, on dead coral rubble; ANSP 304860.

Marshall Islands • 1 specimen 37/30 mm; Jaluit; 06N, 170E; 20 Oct 1917; C Hessle, S Bock’s Pacific Expedition 1917–1918 leg.; west shore southeast of entrance; SMNH 180356.

Mauritius • 10 specimens up to 90/60 mm; probably Mauritius according to a new label (the original label was destroyed); 1929–1930; T Mortensen leg.; NHMD 613752.

New Caledonia • 1 specimen 100/60 mm; Touho, NW, Koë Reef, 2 mi. SSE; 16–20 Jan 1961; Kline & Orr leg.; 0–4 feet, live and dead coral, sand, weed; ANSP 270221.

Palau • 1 specimen 80/65 mm; ANSP 203028.

Seychelles • 1 specimen 90/70 mm; 1830; Dussumier leg.; MNHN-IM-2012-25149. • 1 specimen 85/60 mm; 1841; L. Rousseau leg.; MNHN-IM-2012-25148.

Tanzania • 1 specimen 65/50 mm; Zanzibar; 1902; C Eliot leg.; ANSP 84336. • 1 specimen 80/65 mm; west coast; Jun 1995; M Richmond & M Toni (from N Yonow’s personal collection) leg.; sheltered, on limestone rock, intertidal exposed at low tide, common at night; MNHN-IM-2014-7991.

###### GenBank sequence.

One COI sequence was obtained from GenBank (LC390402) for an individual identified as *Peronia* sp. and collected from Okinawa, Japan (Takagi et al. 2019), which is the northernmost confirmed locality for *Peronia peronii*.

###### Distribution

(Fig. 6). Given that our fresh molecular samples of *P. peronii* from the West Pacific (Guam, Papua New Guinea) are conspecific with those from Mauritius, it is assumed here that all individuals with a long spine of the accessory penial gland belong to the same species. Strictly speaking, however, the presence of *P. peronii* from places like Zanzibar, the Maldives, Nicobar Islands, West Papua, Timor, Palau, New Caledonia, and Tonga, would still need to be validated with fresh material.

Interestingly, but for unclear reasons, *Peronia peronii* seems to be only recorded from relatively small islands, the largest ones being Timor, New Caledonia, and Fiji. Even in Papua New Guinea, it was found on small islands close to the mainland but not on the mainland. *Peronia peronii* seems to be transported across vast distances from the western Pacific Ocean to the western Indian Ocean, but which does not seem to settle on the coasts of large land masses. We did not find it in any of the many localities we visited in the Philippines, Vietnam, Malaysia, Borneo, Sulawesi, Halmahera, Sumatra, etc. It is possible that we occasionally missed it in a few places (obviously we missed it in Timor and New Caledonia where it is present), but it is unlikely that we missed it everywhere.

The presence of *P. peronii* is confirmed in the following locations (Fig. 6): Chagos Archipelago (new record); Fiji (type locality of *O. melanopneumon* and *P. fidjiensis*; Hoffmann, 1928; present study); India, Nicobar Islands (Mörch 1872a, b: 325, as *P. verruculata*; Bergh, 1884a, as *O. tonganum*; Hoffmann, 1928; present study); Indonesia, Java (Hoffmann 1928; present study), Sumatra (Hoffmann 1928; Labbé 1934a; present study), Tanimbar (new record), Timor (Cuvier 1804, paralectotype of *O. peronii*), West Papua (type locality of *O. punctatum* Quoy & Gaimard, 1832); Japan, Okinawa (Takagi et al. 2019, as onchidiids of “Group II”; new record); Kiribati, Gilbert Islands (Hoffmann 1928; present study); Madagascar (Odhner 1919; present study); Maldive Islands (Marcus and Marcus 1960, 1970; present study); Mariana Islands, Guam (Dayrat et al. 2011; White et al. 2011; present study); Marshall Islands (Hoffmann 1928; present study); Mauritius (type locality of *O. peronii* and *P. mauritiana*; Semper 1880; Bergh 1884a, as *O. tonganum*; Plate 1893; present study); New Caledonia (new record); Palau (new record); Papua New Guinea, Madang (new record), New Ireland (new record); Seychelles (Labbé 1934a; present study); Tanzania, Zanzibar (new record); and Tonga (type locality of *O. tonganum* Quoy & Gaimard, 1832). The most western records of *P. peronii* are Zanzibar and southwestern Madagascar; its most eastern records are Okinawa, Guam, Kiribati, and Tonga. Note that *P. peronii* is most likely also present in Tokara Islands (Baba 1958: 144, as *O. verruculatum*), just south Kyushu, ca. 30N, which would be its most northern record.

The following records from the literature are not confirmed here, because authors did not provide enough information supporting the identification: Djibouti (Vayssière 1912; O’Donoghue 1929; Labbé 1934a); India, Nicobar (Godwin-Austen 1895, as *O. mauritianum*; Patil & Kulkarni, 2013); Indonesia, West Papua (Quoy and Gaimard 1832, as *O. tonganum*); Japan (Arimoto et al. 1993); Kenya (Martens 1897); Madagascar (Marcus and Marcus 1970); Mariana Islands, Guam (Biskupiak and Ireland 1985); Mozambique (Martens 1879; Connolly 1912, 1939; Macnae and Kalk 1958); Papua New Guinea, New Ireland (Labbé 1934a, as *P. tongana*); Persian Gulf (White 1951; Bitaab et al. 2015); Philippines (Casto de Elera 1896, as *O. tonganum*), Bohol (Semper 1880, as *O. tonganum*); Red Sea (Sturany 1904); Samoa (Semper 1880, as *O. tonganum*); South Africa, Natal (Krauss 1848; Sturany 1898; Collinge 1910; Connolly 1912, 1939; Morrisey et al. 2010); Australia, Lord Howe Island (Bretnall 1919; Hoffmann 1928), Torres Strait (Smith 1884, as *Onchidium punctatum*), Western Australia (Bretnall 1919).

###### Etymology.

*Onchidium peronii* was named after François Péron [1775–1810] who collected the two slugs described by Cuvier during the Baudin expedition [1800–1803]. *Peronia mauritiana*, *Onchidium tonganum*, and *Paraperonia fidjiensis* were named after type localities. *Onchidium punctatum* was named after the speckled (*punctatum* in Latin) dorsal notum of live animals. *Onchidium melanopneumon* was named after the black (*melas* in Greek) lung (*pneumon* in Greek) tissue of the holotype.

###### Habitat

(Fig. 7). Live slugs of *Peronia peronii* are found in the rocky intertidal, like most other *Peronia* slugs. Many of our specimens were collected at night or just before sunrise, suggesting that *P. peronii* is, at least partly, a nocturnal species. This could explain why we missed it at some localities where we only collected during the day. *Peronia peronii* is not rare, but it is definitely not as common as some other species. The fact that collecting it at night seems necessary, at least in some localities, might explain why collections of *P. peronii* are not as abundant as collections of *P. verruculata*.

###### Color and morphology of live animals

(Fig. 8). No picture of live animals was available for individuals from the West Pacific (Guam and Papua New Guinea). The description of the color of live animals is based on the Mauritius individuals. The dorsal notum is brown, with a greenish hue, light to dark, mottled with darker and lighter areas. The color of the dorsal papillae varies a s that of the background itself. The ventral surface (foot and hyponotum) is yellowish-greenish and can change rapidly in any given individual. The ocular tentacles are brown-grey, like the head. The dorsal notum of live animals is covered by dozens of papillae of various sizes. Dorsal papillae can be particularly tall (easily up to 4 mm), even in preserved specimens, and are evenly distributed over the entire notum. Preserved, they are difficult to distinguish from retracted dorsal gills in the posterior half of the notum. Some papillae bear black dorsal eyes at their tip. The number of papillae with dorsal eyes is variable (15–20). The longest animals are 140 mm long in Mauritius and 115 mm long in the West Pacific.

###### Digestive system

(Figs 9–12). Examples of radular formulae are presented in Table 5. The median cusp of the rachidian teeth is approximately 75 μm long. The hook of the lateral teeth is approximately 160–200 μm long. The intestinal loops are of type I, with a transitional loop oriented between 12 to 3 o’clock.

**Table 5.** Radular formulae in *Peronia* species. Each formula follows the same format: number of rows × number of lateral teeth per left half row - 1 (rachidian tooth) - number of lateral teeth per right half row. Each DNA extraction number corresponds to one individual. The letter H next to an extraction number indicates a holotype.

Species	Radular formula	Spm length (mm)	-	Locality	DNA extraction number
***P. verruculata*** (unit #1)	63 × 70-1-70	45	UMIZ 00170	Sulawesi	2127
	73 × 92-1-92	40	ITBZC IM 00021	Vietnam	5621
	56 × 60-1-60	25	MTQ	Queensland	2622
	74 × 75-1-75	45	UMIZ 00166	Halmahera	5068
	52 × 57-1-57	40	UMIZ 00162	Ambon	2729
	60 × 60-1-60	40	UMIZ 00168	Lombok	2987
	70 × 72-1-72	50	UMIZ 00169	Seram	2870
	70 × 75-1-75	35	MNHN-IM-2013-12010	Papua New Guinea	5469
	56 × 56-1-56	17	MNHN-IM-2013-62393	Vanuatu	5481
***P. verruculata*** (unit #2)	70 × 75-1-75	50	UMIZ 00178	Sumatra	1746
	70 × 78-1-78	50	UMIZ 00180	Sumatra	1797
	60 × 68-1-68	45	UMIZ 00180	Sumatra	1795
	64 × 70-1-70	55	UMIZ 00180	Sumatra	1796
***P. verruculata*** (unit #3)	68 × 78-1-78	35	USMMC 00051	Peninsular Malaysia	976
	70 × 92-1-92	40	USMMC 00065	Peninsular Malaysia	2547
	65 × 85-1-85	25	ZRC.MOL.10496	Singapore	990
	72 × 86-1-86	35	USMMC 00064	Peninsular Malaysia	975
***P. verruculata*** (unit #4)	73 × 85-1-85	50	MNHN-IM-2019-1384	Pakistan	6164
	75 × 95-1-95	50	MNHN-IM-2019-1385	Pakistan	6165
	75 × 92-1-92	40	MNHN-IM-2019-1386	Pakistan	6166
***P. verruculata*** (unit #5)	65 × 75-1-75	40	MNHN-IM-2019-1610	Madagascar	3231
	55 × 63-1-63	35	MNHN-IM-2019-1611	Madagascar	3144
	55 × 65-1-65	25	MNHN-IM-2013-62398	Mozambique	5510
***P. verruculata*** (Red Sea)	66 × 86-1-86	35	ZMH 27472	Red Sea	#1
	80 × 95-1-95	40	ZMH 27472	Red Sea	#2
	67 × 80-1-80	35	ZMH 27472	Red Sea	#3
	70 × 80-1-80	35	ZMH 27472	Red Sea	#4
***P. madagascariensis***	85 × 90-1-90	40	MNHN-IM-2009-16392	Madagascar	5501
	78 × 90-1-90	40	MNHN-IM-2009-16412	Madagascar	5504
	47 × 50-1-50	10	UF 332088	Oman	703
	70 × 72-1-72	35	NMSA W7547	South Africa	5841
***P. peronii***	90 × 115-1-115	110	MNHN-IM-2019-1606	Mauritius	3605
	90 × 105-1-105	140	MNHN-IM-2019-1605	Mauritius	5872
	95 × 105-1-105	80	MNHN-IM-2013-14052	Papua New Guinea	5472
	90 × 100-1-100	70	MNHN-IM-2013-16260	Papua New Guinea	5476
	100 × 110-1-110	85	MNHN-IM-2019-1609	Guam	5840
***P. platei***	75 × 75-1-75	12	UF 303653	Hawaii	706
	65 × 78-1-78	12	UF 303653	Hawaii	5380
	70 × 90-1-90	30	BPBM 284527	Hawaii	6160
	70 × 80-1-80	30	BPBM 284528	Hawaii	6161
	67 × 75-1-75	14	MNHN-IM-2013-13762	Papua New Guinea	5405
	70 × 72-1-72	20	MNHN-IM-2013-13351	Papua New Guinea	5412
***P. sydneyensis***	56 × 60-1-60	30	AM C468916.001	New South Wales	1516 H
	51 × 60-1-60	23	AM C468915.001	New South Wales	1517
	47 × 45-1-45	6	MTQ	Queensland	2667
	58 × 70-1-70	50	MTQ	Queensland	2680
	35 × 35-1-35	12	MNHN-IM-2019-1594	New Caledonia	6189
	55 × 70-1-70	41	MNHN-IM-2019-1595	New Caledonia	6195
	45 × 46-1-46	25	MNHN-IM-2019-1598	New Caledonia	6220
***P. okinawensis***	60 × 60-1-60	20	UF 352288	Okinawa, Japan	696-2
	60 × 60-1-60	25	UF 352288	Okinawa, Japan	696-3
	65 × 65-1-65	27	UF 352288	Okinawa, Japan	696-4 H
***P. setoensis***	50 × 50-1-50	20	NSMT-Mo 78985	Wakayama, Japan	5383 H
	55 × 55-1-55	15	NSMT-Mo 78987	Wakayama, Japan	3753
	50 × 50-1-50	15	NSMT-Mo 78987	Wakayama, Japan	3754
***P. griffithsi***	50 × 45-1-45	18	UMIZ 00177	Kei	2934
	50 × 50-1-50	17	UMIZ 00176	Kei	2936
	50 × 45-1-45	25	UMIZ 00177	Kei	3566
	45 × 52-1-52	20	MNHN-IM-2019-1608	Mauritius	3153
	50 × 52-1-52	20	MNHN-IM-2019-1608	Mauritius	3156
	50 × 50-1-50	15	MNHN-IM-2000-35265	Mauritius	3157 H
***P. willani***	58 × 80-1-80	50	NTM P.57625	Northern Territory	1628 H
	53 × 60-1-60	40	NTM P.57627	Northern Territory	1668
	65 × 80-1-80	60	NTM P.57627	Northern Territory	1626
	70 × 90-1-90	65	NTM P.57626	Northern Territory	1620

###### Reproductive system

(Figs 13–16). In the anterior (male) parts, the muscular sac of the accessory penial gland is at least 30 mm long in specimens from Mauritius and at least 25 mm long in specimens from the West Pacific (Guam & Papua New Guinea). Note that, in some additional museum specimens, the muscular sac was only 20 mm long, and, even exceptionally 17 mm long (see remarks below). The hollow spine of the accessory penial gland is narrow, elongated, and straight or slightly curved, and its shape (including at its tip) varies between individuals. Its length ranges from 3.4 mm ([5872] MNHN-IM-2019-1605) to 3.6 mm ([3605] MNHN-IM-2019-1606) in Mauritius, and from 3.5 mm ([5472] MNHN-IM-2013-14052) to 4 mm ([5471] MNHN-IM-2013-12500) in the West Pacific (Guam and Papua New Guinea). Its diameter at the conical base is approximately 400 μm in specimens from Mauritius and between 400 and 500 μm in specimens from the West Pacific (Guam and Papua New Guinea). Its diameter at the tip measures 160–170 μm in specimens from the West Pacific, and from 180 to 200 μm in specimens from Mauritius. Note that, in some additional museum specimens, the spine was only 3 mm long (see remarks below).

The retractor muscle is shorter or longer than the penial sheath and inserts near the heart. Exceptionally, the retractor muscle can even be vestigial ([5472] MNHN-IM-2013-14052). Inside the penial sheath, the penis is a narrow, elongated, soft, hollow tube. Its distal end bears conical hooks which are less than 50 μm long.

###### Diagnostic features

(Table 4). *Peronia peronii* is the only *Peronia* species which is easy to identify anatomically. Indeed, it is characterized by a very long spine (at least 3 mm) of the accessory penial gland, which is distinctive and easily accessible (one just needs to pull on the flagellum of the penial gland or, even, in some cases, measure the spine by transparency). The two longest spines were found in the lectotype of *P. fidjiensis* (MNHN-IM-2000-33692) from Fiji (5 mm), and in an old historical specimen (ANSP 304860) from the Maldives (4.8 mm).

*Peronia peronii* is additionally characterized by a unique combination of anatomical traits: muscular sac longer than 20 mm, intestinal loops of type I (with a transitional loop oriented between 12 and 3 o’clock), retractor muscle inserting near the heart. Also, no individual larger than 80 mm was found in any other *Peronia* species so far. Animal size can be useful when several *Peronia* species are found at the same site. For instance, the two individuals of *P. verruculata* (unit #1) found at the station PM 12 (near Madang, Papua New Guinea) are 35 and 38 mm long while the individual of *P. peronii* from the same station is 80 mm long. The type I of its intestinal loops (with a transitional loop oriented between 12 and 3 o’clock) is only shared by *P. okinawensis*, a species endemic to Japan with which it is most closely related.

###### Remarks.

***Synonymies.*** There is no doubt that Cuvier’s (1804)*Onchidium peronii* applies to the species described here, just based on animal size alone. According to Cuvier, the lectotype from Mauritius measured approximately 140 mm long and our molecular data show that all individuals of that size from Mauritius belong to a single species (Table 4). Cuvier’s (1804: pl. 6) detailed anatomical description and drawings are exclusively based on the lectotype (he did not dissect the paralectotype from Timor). Cuvier (1804: 48, pl. 6, fig. 8) described the spine of the accessory penial gland as a “very sharp, brown spike” but unfortunately did not provide its length. However, Cuvier’s (1804: pl. 6, fig. 4) illustration of the intestinal loops is identical to some of our Mauritius individuals here: intestinal loops of type I with a transitional loop at 3 o’clock. The paralectotype of *Onchidium peronii* from Timor (MNHN-IM-2000- 22938) is only briefly mentioned by Cuvier in the original description. The length (4.5 mm) of the spine of its accessory penial gland (checked for the present study) indicates that it also belongs to *P. peronii*. Its intestinal loops are also identical to those of the lectotype (Fig. 9A).

*Peronia mauritiana* is a junior objective synonym of *Onchidium peronii* because they share the same name-bearing type.

*Onchidium tonganum* was described by Quoy and Gaimard (1832: 210–211, pl. 15, figs 17, 18) from “Panhi-Motou,” possibly the small island of Pangaimotu, Tonga, based on an unspecified number of individuals. The illustrations of the dorsal gills by Quoy and Gaimard (1832: pl. 15, figs 17, 18) and their presence on the notum of the lectotype (MNHN-IM-2000-22937) clearly indicate that *Onchidium tonganum* belongs to *Peronia*. The lectotype was dissected prior to the present study and most of the male copulatory parts are missing (only the deferent duct remains). As a result, the length of the spine of the accessory penial gland, which is diagnostic of *P. peronii*, cannot be checked. Labbé (1934a: 191) listed the lectotype in the material he examined for his re-description of *P. tongana*, but he did not point out that it was part of the type material of *O. tonganum* and he did not describe it anatomically. It is possible but not certain that Labbé dissected the lectotype. At any rate, its intestinal loops are of type I with a transitional loop between 2 and 3 o’clock (Fig. 9B). Both the length (100 mm) of the lectotype as well as its intestinal loops indicate that *Peronia tongana* is a junior synonym of *P. peronii* (Table 4).

*Onchidium punctatum* is regarded here as a junior synonym of *P. peronii* because the length (3.7 mm) of the spine of the accessory penial gland of the lectotype (MNHN-IM-2000-22966) is only compatible with *P. peronii* (Table 4). The length of the lectotype (70 mm, preserved) is also far more compatible with *P. peronii* than with *P. verruculata*, another species found in West Papua. Our many individuals of *P. verruculata* are all less than 60 mm long (alive), except a single individual from New Caledonia (73 mm alive). Given their small size, the two paralectotypes (MNHN-IM-2000-33701) likely belong to *P. verruculata* (unit #1) instead of *P. peronii*, which would not be surprising at all because the type locality of *O. ferrugineum* (a junior synonym of *P. verruculata*) is the same as that of *O. punctatum* (Manokwari, West Papua, Indonesia). At the end of the description of *O. punctatum*, Quoy & Gaimard (1832: 216) also mention in passing that they also found *Onchidium tonganum* in Port Dorey (i.e., Manokwari, West Papua, Indonesia) and they even point out that local inhabitants know how to distinguish both species. Both *O. punctatum* and *O. tonganum* are regarded here as junior synonyms of *P. peronii*. However, it remains true that there are two sympatric *Peronia* species in West Papua, *P. verruculata* and *P. peronii*, which can be distinguished in the field based on animal length (except, of course, for individuals measuring less than 60 mm long).

Bergh (1884a: 129–142, pl. IV, figs 25–27, pl. V, figs 1–27, pl. VI, figs 5–18, 20, 21) described *Onchidium melanopneumon* from a single individual (65/40 mm) from Fiji. This specimen was completely dissected by Bergh and is now empty (NHMUK 1888.5.30.39). *Onchidium melanopneumon* applies to a *Peronia* species due to the presence of dorsal gills, and the length (4 mm) of the spine of the accessory penial gland indicates that it applies to *P. peronii* (Table 4). Its intestinal loops (Bergh 1884a: pl. V, fig. 27) are also similar to those found in *P. peronii*, although the transitional loop is slightly past the 3 o’clock limit. As a result, *O. melanopneumon* is regarded as a junior synonym of *P. peronii*. Bergh (1884b: 263; 1885: 176) briefly mentioned again *O. melanopneumon* in a comparative study on the affinities of onchidiids.

Labbé (1934a: 197–198, figs 9–11) described *Paraperonia fidjiensis* based on seven individuals from Fiji, one of which could be found and is designated as the lectotype (MNHN-IM-2000-33692). Because all reproductive parts are missing, the length of the spine of the accessory penial gland cannot be checked. However, according to Labbé (1934a: 197, fig. 10), the spine of the accessory penial gland is 5 mm long, which is only compatible with *P. peronii* (Table 4), and is the longest spine known in *P. peronii*. The intestinal loops of the lectotype of *P. fidjiensis* are clearly of type I, with a transitional loop oriented at ~ 1 o’clock (Fig. 9E), even though Labbé (1934a: 197) erroneously described them a type V, which is a mistake he often made. Given the length of the lectotype (60 mm) and, most importantly, the length of the spine of the accessory penial gland, *P. fidjiensis* is regarded as a junior synonym of *P. peronii*.

***Secondary literature*.** Several early authors mentioned Cuvier’s *Onchidium peronii* without any new material (Cuvier 1816: 411; Cuvier 1830: 46; Férussac 1822: xxxi; Fleming 1822a: 574; Fleming 1822b: 463; Lamarck 1822: 46; Voigt 1834: 101). Oken (1834a: 287) transferred Quoy and Gaimard’s (1832) *Onchidium tonganum* and *O. punctatum* to *Peronia* but with no justification.

In the seventh volume of the second edition of Lamarck’s *Histoire naturelle des animaux sans vertèbres*, which was revised by Deshayes and Milne-Edwards (1836), *P. mauritiana* is proposed as a synonym of *Onchidium peronii*. However, as a reference for *P. mauritiana*, the authors mentioned the illustration published by Blainville (1827: pl. 46, fig. 7) in the *Atlas* of his *Manuel* which differs from that published by Cuvier (1804: pl. 6, fig. 1) and may or may not refer to *Peronia mauritiana*.

John Edward Gray (1850: 117) listed *Onchidium peronii* as a synonym of *P. mauritiana* and his wife Maria Emma Gray (1850: pl. 181, fig. 7) reproduced Cuvier’s (1804: pl. 6, fig. 1) original figure of the dorsal notum of *Onchidium peronii*. As a result, JE Gray (1850: 117) and ME Gray (1850: pl. 181, fig. 7) are listed above as correct references of *O. peronii*. In the same work, JE Gray (1850: 117) regarded *P. punctata* and *P. tongana* (as spelling mistake *tongensis*) as valid, and ME Gray (1850: pl. 182, fig. 1, pl. 183, fig. 3) reproduced the original illustrations by Quoy and Gaimard (1832: pl. 15, figs 17, 18, 27, 28). As a result, those names are also listed above as correct references. According to JE Gray (1850: 117), *P. mauritiana* (as *mauriciana*) was a valid *Peronia* species name but ME Gray (1850: pl. 183, fig. 2) reproduced Blainville’s (1827: pl. 46, fig. 7) illustration which differs from that published by Cuvier (1804: pl. 6, fig. 1) and which may or may not refer to *P. peronii* because there are two *Peronia* species in Mauritius (Fig. 6). Therefore, ME Gray’s (1850: pl. 183, fig. 2) *Peronia mauritiana* is not listed above as a correct reference of *P. peronii*. And, finally, ME Gray’s (1850: pl. 183, figs 4, 4a, 5) reproductions of Savigny’s (1817: pl. II, figs 3.1–3.3) illustrations of *Onchidium peronii* from the Red Sea do not represent *P. peronii* (see remarks on *P. verruculata*).

Adams and Adams (1855: 235) merely listed *Peronia mauritiana*, *P. peronii*, *P. punctata*, and *P. tongana* as *Peronia* species names. Note that for *P. peronii*, they refer to Savigny’s illustrations of individuals from the Red Sea misidentified as *P. peronii* by Audouin instead of Cuvier’s original description of *P. peronii*, which means that Adams and Adams refer to *P. verruculata* instead of *P. peronii* (see remarks on *P. verruculata*). Adams and Adams (1855: pl. LXXXI, fig. 3) also reproduced the original illustration of *O. tonganum* by Quoy and Gaimard (1832: pl. 15, fig. 17).

Berge (1855: 124) mentioned “*Onchidium peronii* Cuv.” from the East Indies and the Red Sea but with no new material or literature reference except for a German translation of Cuvier’s *Règne Animal* by Voigt (1834: 101) as well as “Cuvier, Règ. anim. pl. 26, fig. 2.” Berge (1855: 124) followed Voigt (1834: 101) and accepted *P. mauritiana* as a synonym of *O. peronii*. Cuvier’s illustration (pl. 26, fig. 2) mentioned by Berge was actually published after Cuvier’s death in the Disciples’ edition of the *Règne Animal* which was accompanied by beautiful illustrations. According to Cowan (1976), the authorship for the mollusks should be attributed to Deshayes (1836–1845) who prepared the volume of text and the atlas published in livraisons between 1836 and 1845. However, the exact dates of publication are still unknown for most pages and plates, including for the page 69 and the plate 26 where *Onchidium* is mentioned and illustrated. Note the spelling mistake *Unchidium* on the figure caption of plate 26. The illustrations in both Deshayes (1836–1845: pl. 26, fig. 2) and Berge (1855: pl. 16, fig. 8) are mere reproductions of Cuvier’s (1804: pl. 6, fig. 5) anatomical drawing of *O. peronii*. Berge (1855: 124) also mentioned “*Onchidium punctatum* Quoy” from Australia (as Neuholland) but with no new material or literature reference except for another illustration (pl. 26, fig.1) from the Disciples’ edition of the *Règne Animal*. Again, the illustrations in both Deshayes (1836–1845: pl. 26, fig. 1) and Berge (1855: pl. 21, fig. 7) are mere reproductions of Quoy and Gaimard’s (1832: pl. 15, fig. 27) original illustration of *O. punctatum*. It cannot be determined which species Berge referred to exactly (because the localities mentioned by Berge are not the type localities).

The record of *Onchidium peronii* from Natal, South Africa (Krauss 1848: 72) likely is a record of *P. madagascariensis*, the only *Peronia* species known in South Africa so far (see remarks on *P. madagascariensis*). However, *P. verruculata* (unit #5) could also be present in northeastern South Africa because its southernmost known locality is in Maputo, Mozambique (ca. 26°S). This record by Krauss was mentioned again by a few authors (Sturany 1898: 73; Collinge 1910: 171; Connolly 1912: 224–225; Connolly 1939: 454).

Chenu (1859: 474, fig. 3505) mentioned *Peronia punctata* with no additional material or records, and with a reference to Quoy and Gaimard’s (1832: pl. 15, fig. 27) original illustration of *O. punctatum*.

Keferstein (1865a: pl. CII, fig. 20) reproduced Quoy and Gaimard’s (1832: pl. 15, fig. 17) original illustration of *Onchidium tonganum*, which he classified in *Peronia*. Keferstein (1865a: pl. CIII, fig. 1) also reproduced Cuvier’s (1804: pl. 6, fig. 4) original illustration of the internal anatomy of *O. peronii*, which he also classified in *Peronia*.

Based on the collections of the Galathea Expedition preserved in Copenhagen, Mörch (1872a: 28; 1872b: 325) mentioned *Peronia verruculata* (as spelling mistake *vermiculata* in 1872b) from Pulo Milu [Pulo Milo, Little Nicobar] and Nancouri [Nancowry, Nicobar Islands], where he says it is common. Given the animal size (up to 133 mm long alive), we agree with Hoffmann (1928: 71) that Mörch’s record is very likely a record of *P. peronii*. The preserved specimen (88/38 mm) reported by Mörch (1872a: 28; 1872b: 325) from the Galathea collections most likely is the specimen identified by Semper as *Onchidium tonganum*, described by Bergh (1884a: 142–148, pl. VI, fig. 19, pl. VII, figs 1–6), examined by Hoffmann (1928: 44) for his description of *O. peronii*, and finally re-examined for the present study (NHMD 613753). Bergh (1884b: 264; 1885: 177) briefly mentioned *O. tonganum* again in a comparative study on the affinities of onchidiids. Mörch (1872a: 28; 1872b: 325) mentioned *Peronia mauritiana* from Sambelong, Great Nicobar, Nicobar Islands, based on much smaller individuals from the collections of the Galathea Expedition in Copenhagen, which are a record for *Peronia verruculata* (see remarks on that species).

The record of *Onchidium mauritianum* from the Red Sea by Pagenstecher (1877: 62) refers to either *Peronia verruculata* or *P. madagascariensis* (Fig. 6).

The records of *Onchidium peronii* from Mozambique by Martens (1879: 735) in Ibo Island (ca. 12°21'S) and Inhambane (ca. 23°52'S) are within the geographical range of both *P. verruculata* (unit #5) and *P. madagascariensis* (Fig. 6). It is not possible to know to what species Martens was referring; this record by Martens was mentioned twice by Connolly (1912: 225; 1939: 454).

Semper (1880: 258–260, pl. XIX, figs 2, 9, pl. XXII, figs 1, 2, 10) referred to huge onchidiid slugs (from 50 to 105 mm, preserved) as Quoy and Gaimard’s (1832) *Onchidium tonganum* and merely suggested, with a question mark, that *O. peronii* could refer to the same species. Semper (1880: 258) listed five geographical records for *O. tonganum*: Tonga and West Papua (as Port Dorey), from Quoy and Gaimard (1832); Mauritius, based on some material from the Vienna and Kiel museums; Samoa, based on some material from the Museum Godeffroy; and Bohol, Philippines, based on his own collections. Semper (1880: 258) indicated that the specimens he examined were from 50 to 105 mm long, preserved, and that the smallest individual was found in Mauritius. His anatomical description perfectly matches the anatomy of *P. peronii*. In particular, a spine of an accessory penial gland measuring 4 mm long is only compatible with *P. peronii* (Table 4). However, he did not clearly indicate whether he observed a long spine in every specimen. It cannot be excluded that he measured the length of the accessory penial gland spine only in a specimen from Mauritius. Therefore, the records of *P. peronii* in Bohol and in Samoa are regarded here as questionable, even though it is very possible that *P. peronii* lives in Samoa, given that it is so close to Tonga (800 km) and Fiji (1100 km).

Semper (1882: 290) thought that Cuvier’s (1804) original description of *P. peronii* was problematic because his drawing of the dorsal notum did not match the internal anatomy. Because Semper was convinced that Cuvier used specimens that did not belong to the same species, he thought that the name *P. peronii* should not be used. Plate (1893: 172–173) disagreed with Semper even without examining the type material of *P. peronii*. It is demonstrated here that the two type specimens described by Cuvier as *P. peronii* both belong to the same species (see above). Semper (1882: 268) was undecided about the nomenclatural status of what he called *Onchidium mauritianum* (then a new combination), which he listed as one of the names for which a “closer inspection of the originals” was needed. Like most authors, he cited Blainville’s (1827: pl. 46, fig. 7) illustration (which is not part of Blainville’s original description) as a reference without realizing that it may or may not correspond to *P. mauritiana*, a junior objective synonym of *P. peronii*. Semper (1882: 289) was also undecided about the status of *Onchidium punctatum*, for which he erroneously thought that the type locality was unknown. He suggested that it might refer to the same species as *Onchidium tumidum*, which is not possible because *O. tumidum* was recently transferred to *Paromoionchis* (Dayrat et al. 2019a).

Tapparone Canefri (1883: 214) listed all of Semper’s geographic records for *Peronia tongana* with no new material or anatomical observations (see above). Tapparone Canefri (1883: 214) also regarded *Peronia punctata* as a valid species name, but with no other reference or material than the original description by Quoy and Gaimard (1832). Tapparone Canefri’s suggestion that *Peronia punctata* could refer to the same species as *Onchidium tumidum* must be rejected because *O. tumidum* was recently transferred to *Paromoionchis* (Dayrat et al. 2019a).

Smith (1884: 92) mentioned Onchidium (Peronia) punctatum from Albany Island and Thursday Island, in the Torres Strait, without any description. This is likely a record of *Peronia verruculata* (unit #1), the only species thought to be present in the Torres Strait, although our study does not include any fresh material from the Torres Strait and *P. peronii* could also live there (Fig. 6). Note that Thursday Island also happens to be the type locality of *Scaphis viridis*, a junior synonym of *Peronia verruculata*.

Bergh (1884a: 142–148, pl. VI, fig. 19, pl. VII, figs 1–6) described *as Onchidium tonganum* a specimen from the collections of the Copenhagen Museum which was collected in the Nicobar Islands during the Galathea Expedition (station 305). That specimen (85/55 mm), dissected by Bergh, is in a jar (NHMD 613753) with a second specimen (70/50 mm) which is still entire and not dissected by Bergh. Both specimens were re-examined for the present study, although Bergh’s measurement of the penial gland spine in the largest specimen (4.25 mm) could not be checked because internal organs are missing. Given the specimen sizes, their intestinal loops (type I with a transitional loop at 3 o’clock in the second specimen), and the spine of their accessory penial glands (4.25 mm in the largest specimen according to Bergh, and 4 mm in the second specimen), those two specimens belong to *P. peronii*.

Joyeux-Laffuie (1885: viii–xi) merely mentioned *O. melanopneumon* in a summary of Bergh’s (1884a) work.

Plate (1893: 172–173, pl. 12, figs 85, 87, 91) re-described *Onchidium peronii* based on at least one specimen from Mauritius for which he did not provide any size. However, given the length of the spine of the accessory penial gland (7 mm long), there is no doubt that he examined *P. peronii*. It is possible that he included a part of the duct of the accessory penial gland in that measurement because the longest spine observed in the present study was 5 mm, in the lectotype of *P. fidjiensis* (MNHN-IM-2000-33692). According to Plate, the retractor muscle inserts near the central nervous system, which does fit in the variation observed here (Table 4). Plate listed several synonyms: *Peronia mauritiana*, *Onchidium tonganum*, *O. melanopneumon*, and possibly (with a question mark) *P. corpulenta*. Note that Plate (1893: 172) rightly regarded *O. melanopneumon* as a junior synonym of *O. peronii* but for a weak reason (a similar pigmentation of the lung). These synonymies are all accepted here, except for *P. corpulenta* which is regarded as a *nomen dubium* (see general discussion). Plate (1893) did not comment on *O. punctatum*.

Godwin-Austen (1895: 443) listed *Onchidium mauritianum* in Little Nicobar. It is impossible to know what species was referred to. However, *P. peronii* (of which *P mauritiana* is a junior synonym), is known to be present in Nicobar Islands.

Casto de Elera (1896: 629) mentioned the presence of several species in the Philippines, without description or new material, mostly based on Semper’s (1880–1885) work: *Onchidium verruculatum*, *O. tonganum*, and *O. savignyi* (as *savigngi*). Our data suggest that *Peronia verruculata* (unit #1) lives in the Philippines (Fig. 6).

Martens (1897: 126) listed *Peronia mauritiana* as a synonym of *Onchidium peronii* with a reference to Blainville’s (1827: pl. 46, fig. 7) illustration of *P. mauritiana* which may or may not refer to *P. mauritiana*. Martens (1897: 126) also claimed that *O. tonganum* is a synonym of *O. peronii*, with a reference to the original description by Quoy and Gaimard (1832) as well as to Semper’s (1880: 258–260, pl. XIX, figs 2, 9, pl. XXII, figs 1, 2, 10) re-description, which may only partly correspond to *P. peronii* (see above). The record of *O. peronii* in Mombasa (Mombas, Ostküste Afrika) by Martens (1897: 126) is not supported by any description and is therefore not accepted here, even though it is possibly correct (*P. peronii* is present in Zanzibar).

Collinge (1900: 7) and Connolly (1912: 225) mentioned *Onchidium peronii* from Green Point, Cape Peninsula, South Africa. Those specimens were later used by Watson (1925: 283–284, pl. XXVII, figs 4–11, pl. XXVIII, figs 12, 14, pl. XXXI, fig. 58) to describe *Onchidella capensis* Watson, 1925.

Sturany (1904: 269) mentioned the presence of *Onchidium peronii* in Massawa, Eritrea, Red Sea. This record most likely refers to either *Peronia verruculata* or *P. madagascariensis* (Fig. 6).

*Onchidium punctatum* is one of the eight onchidiid species mentioned by Hedley (1909: 369) from Queensland, Australia, without any reference to any material. It is impossible to know what species Hedley refers to. Our data show that there are two *Peronia* species in Queensland (Fig. 6).

The record of *Onchidium peronii* from Durban, Natal, South Africa by Collinge (1910: 171) likely is a record of *P. madagascariensis*, the only *Peronia* species known in South Africa so far (see remarks on *P. madagascariensis*). However, *P. verruculata* (unit #5) could also be present in northeastern South Africa because its southernmost known locality is in Maputo, Mozambique (ca. 26°S). This record was mentioned again by Connolly (1912: 225; 1939: 454).

According to Connolly (1912: 224–225), *Onchidium peronii* is a valid name and *Peronia mauritiana* (as spelling mistake *mauritziana*) and *O. tonganum* are its synonyms. The references listed by Connolly are all commented on above. Let us briefly emphasize here, however, that the localities of *Onchidium peronii* mentioned by Connolly in South Africa and Mozambique are problematic. Connolly (1939: 454) later admitted that “it is open to question (...) whether the true *O. peronii* Cuv. really exists in South Africa.” Connolly (1939:453), who did not cite Labbé’s (1934a) work, considered that *Peronia* was a subgenus of *Onchidium* and should include onchidiid slugs with dorsal gills.

Vayssière (1912: 125–129) recorded seven individuals of *Peronia peronii* shipped to him from Moucha Islands (Djibouti) by Charles Gravier and Félix Pierre Jousseaume, two of the people who also collected many specimens studied by Labbé (1934a). Vayssière mostly focused on the description of the radula, which is not useful to identify species. Vayssière reported a wide range of animal sizes (from 10 to 80 mm long and from 6 to 60 mm wide). Thus, it is very possible that he examined more than one species. Instead of *P. peronii*, which has never been positively recorded from Djibouti, Vayssière likely examined *P. verruculata*, *P. madagascariensis*, or both (Fig. 6). His specimens of large size most likely were *P. madagascariensis* because *P. verruculata* individuals rarely are longer than 60 mm (Table 4). Note that the number of rows of teeth and the number of teeth per half row mentioned by Vayssière (95 to 100 rows on average) are higher than what was observed here, although they are more compatible with *P. madagascariensis* than *P. verruculata* (Table 5), acknowledging that radular formulae are expected to vary.

It is not possible to determine to what species Odhner (1919: 42) was referring solely based on his brief, external description of *Onchidium peronii* from Toliara, Madagascar. However, his material, dissected here, clearly belongs to *P. peronii*: a single individual (65/50 mm) is characterized by intestinal loops of type I with a transitional loop at 3 o’clock, a spine of the accessory penial gland of 3 mm long, and a muscular sac of 25 mm long (SMNH 180381).

Bretnall (1919) uncritically took for granted every species record ever published, without considering that species often are misidentified. Bretnall (1919) accepted *O. peronii* as a valid name, with *Onchidium tonganum* and *Peronia mauritiana* as synonyms, and *P. corpulenta* as a potential synonym (with a question mark). The references listed by Bretnall (1919: 311–312) for *O. peronii* are all commented on above already. However, Bretnall’s (1919: 313) list of geographic records needs to be discussed, especially because Bretnall did not mention the key characters supporting a proper identification of *P. peronii* (Table 4). The presence of *P. peronii* in Samoa, which Bretnall obtained from Semper (see above), should not be taken for granted, even if it is quite possible. The presence of *P. peronii* in the Buccaneer Archipelago, northern Western Australia (16S, 123E), based on specimens from the Australian Museum, should not be taken for granted, even though it is quite possible. The identification of *P. peronii* in the Santa Cruz Islands, Solomon Islands, based on specimens from the Australian Museum, should not be taken for granted (specimens may have been misidentified), even if the Santa Cruz Islands are within the known geographical range of *P. peronii* (Fig. 6). Bretnall (1919: 315–316) also regarded *O. melanopneumon* as a valid name, for which he cited Bergh’s (1884a) original description and its French summary by Joyeux-Laffuie’s (1885), and indicated Plate’s (1893) proposed synonymy (with *O. peronii*) with a question mark. Bretnall (1919: 316) listed Lord Howe Island, off southeastern Australia (based on specimens from the Australian Museum), as a locality for *O. melanopneumon*, but without description of key characters. Thus, the presence of *P. peronii* in Lord Howe Island, which is 1350 km south of the southernmost known locality of *P. peronii* (New Caledonia), is not taken for granted here. As for *Onchidium punctatum*, Bretnall (1919: 316–317) followed Semper (1882: 289) and Tapparone Canefri (1883: 214) who both thought that it could be a synonym of *Onchidium tumidum* (see above), which is not possible because *O. tumidum* refers to a *Paromoionchis* species (Dayrat et al. 2019a).

Hoffmann (1928: 71), following most of Plate’s (1893) nomenclatural decisions, accepted *Peronia mauritiana*, *P. corpulenta*, *Onchidium tonganum* and *O. melanopneumon* as junior synonyms of *O. peronii*. Hoffmann, like other authors, did not mention the key anatomical characters that allow a reliable identification of *P. peronii* and uncritically accepted most geographical records published before him. As a result, his proposed distribution for *O. peronii* should not be taken for granted. For instance, the presence of *P. peronii* in Lord Howe Island, off southeastern Australia, obtained from Bretnall (1919) is questionable. Hoffmann (1928: 44) examined a specimen from the Nicobar Islands (NHMD 613753) which was originally mentioned by Mörch (1872a: 28; 1872b: 325; see above). Hoffmann (1928: 44–45) also provided several geographical records (Sumatra, Java, Marshall Islands, Kiribati, Fiji) for *O. peronii* based on material preserved at the SMNH in Stockholm. His material was re-examined and all records are confirmed. Hoffmann only dissected two individuals, one from Sumatra (SMNH 180354) and one from Kiribati (SMNH 180379). The other eighteen specimens were dissected for the present study.

Eight large specimens (longer than 65 mm) examined by Hoffmann from Sumatra (SMNH 180354), Java (SMNH 180355), Kiribati (SMNH 180376, 180377, 180380, 180382, 180475), and Fiji (SMNH 180373) share the diagnostic characteristics of *P. peronii*: a spine of the accessory penial gland between 3 and 4 mm long, intestinal loops of type I with a transitional loop at 3 o’clock, and a muscular sac between 20 and 25 mm long (exceptionally 17 mm, SMNH 180354). Seven smaller specimens (between 15 and 37 mm long) examined by Hoffmann from the Marshall Islands (SMNH 180356), Fiji (SMNH 180374), and Kiribati (SMNH 180353, 180383, 180384) are immature: the anterior male reproductive parts are barely developed, and, if present, the spine of the accessory penial gland is still soft (SMNH 180353). Given their intestinal loops (type I with a transitional loop at 3 o’clock), they are regarded as individuals of *P. peronii*. In other species, individuals of that size are already fully mature. Two smaller specimens (between 28 and 37 mm long) examined by Hoffmann from Fiji (SMNH 180357, 180375) belong to *P. peronii* because of several characteristics (retractor muscle inserting near the heart, intestinal loops of type I with a transitional loop at 3 o’clock, a spine of 3 mm long). Their muscular sacs (11 and 15 mm) are shorter than in other specimens, suggesting that they likely are not fully mature. Two specimens from Kiribati (SMNH 180378, 180478), poorly preserved, could not be confidently identified. Finally, the male reproductive parts are missing in a specimen from Kiribati dissected by Hoffmann (SMNH 180379), but its intestinal loops (type I with a transitional loop oriented between 1 and 2 o’clock) confirm that it belongs to *P. peronii*.

Note that the locality of the specimen from Sumatra (SMNH 180354) is problematic. The label and Hoffmann’s publication both say “Pulu Pasu, west coast of Sumatra,” but there is no such place on the west coast of Sumatra. There are two small islands off the west coast of Sumatra called Pulau Asu (Hinako Islands) and Pulau Pasumpahan (south of Padang). There also is a small island called Pulau Pasu in the Riau Islands, but that archipelago is located north of Sumatra, in the South China Sea. So, it is unclear where that specimen was collected exactly in Sumatra.

O’Donoghue (1929: 833) reported one specimen (30/21 mm) of *Peronia peronii* from Port Taufiq, Suez, Egypt. A radular formula (65 × 72–1-72) is not enough to identify a *Peronia* species, and he most likely examined *P. verruculata* or *P. madagascariensis* (Fig. 6).

Two names accepted as valid by Labbé (1934a) are regarded as synonyms of *Peronia peronii*: *P. tongana*, and *P. fidjiensis*. Labbé (1934a: 191) himself acknowledged that differences between *P. peronii* and *P. tongana* were weak. The traits that he mentioned (position of the pneumostome with respect to the anus, head longer than the foot) vary greatly due to preservation. Labbé (1934a: 197–198) did not compare *Paraperonia fidjiensis* with *Peronia peronii* probably because he classified them in two distinct genera. However, there are no differences between the type material of *P. fidjiensis* and the type material of *P. peronii*. Labbé (1934a: 190) agreed with most authors that *P. mauritiana* and *O. melanopneumon* were synonyms of *P. peronii*. Like Plate (1893), Labbé (1934a: 190) thought that *P. corpulenta* was simply a potential synonym of *P. peronii* but in fact it is a *nomen dubium* (see general discussion).

All references cited by Labbé for *P. peronii* and *P. tongana* have been commented on above, but Labbé’s (1934a) proposed distribution ranges need additional clarification. Labbé’s (1934a: 190–191) re-description of *P. peronii* was based on one individual (100/70 mm) from Sumatra (MNHN-IM-2012-25150), one individual (90/70 mm) from the Seychelles (MNHN-IM-2012-25149), and ten individuals from the Red Sea (not found in the MNHN collections). At least one of those specimens belongs to *P. peronii* because of the length of the spine of the accessory penial gland mentioned by Labbé as 6 to 7 mm. The specimens from Sumatra and the Seychelles were fully dissected by Labbé (the Sumatra individual is basically empty): the male parts are missing, and it is not possible to determine the type of intestinal loops. However, given their huge size, they most likely belong to *P. peronii*. The presence of *P. peronii* in the Red Sea is possible but, at this stage, questionable: the size mentioned by Labbé for those specimens (17/12 mm) strongly suggests that he did not examine *P. peronii* from the Red Sea. Those specimens from the Red Sea identified as *P. peronii* by Labbé could not be located at the MNHN (there are no specimens collected by Clot-Bey in the collections, and there are too many jars of specimens collected by Jousseaume to determine which jar corresponds to the species description in Labbé’s monograph).

Labbé’s (1934a: 191–192, figs 4–7) re-description of *P. tongana* was based on one individual from Djibouti (Obock), one individual (85/60 mm) from the Seychelles (MNHN-IM-2012-25148), two individuals from New Ireland, and one individual from Tonga which happens to be part of the type series by Quoy and Gaimard (MNHN-IM-2000-22937) even though Labbé does not mention it. The specimen from the Seychelles was re-examined for the present study and, given its huge size, it is confirmed that it belongs to *P. peronii*: its intestinal loops are of type I, with a transitional loop at 3 o’clock; the male parts are missing. There are two specimens (60/50 mm) from New Ireland at the MNHN which could potentially be the two specimens mentioned by Labbé, but the collecting dates do not match. At any rate, it does not matter much since our fresh specimens demonstrate that *P. peronii* is present in New Ireland (Fig. 6). The specimen from Obock could not be traced back at the MNHN; there is a specimen (80/60 mm) which could possibly correspond to it but it is a problematic specimen as it could also be a type specimen for *P. gaimardi*, and is now an empty notum (see below, remarks on the type material of *P. gaimardi* in *P. verruculata*). Thus, the presence of *P. peronii* in Djibouti is not accepted here and would need to be supported by positive evidence.

Risbec (1935: 415) illustrated the eggs of an onchidiid individual from New Caledonia which he called “*Oncidium tonga* Q et G,” clearly a spelling mistake for *Onchidium tonganum* Quoy & Gaimard, 1832. It is not possible to know what species Risbec was referring to because there are three *Peronia* species in New Caledonia (Fig. 6).

White (1951: 241) reported a single specimen (53/38 mm) of *Onchidium peronii* from the Persian Gulf. The radular formula (88 × 88–1-88) is not enough to identify a *Peronia* species. White’s record referred either to *P. verruculata* (unit #4) or *P. madagascariensis* (Fig. 6).

In Japan, Baba (1958: 144) indicated that some specimens of *Onchidium verruculatum* from Tokara Islands, south of Kyushu (ca. 30°N) are very large (up to 120 mm long), suggesting that *P. peronii* is found there, which would be its most northern record.

Macnae and Kalk (1958: 34, 44, 128) mentioned *Onchidium peronii* from Inhaca Island, Mozambique (ca. 26°S). Given that no information is provided for species identification, this record is not taken for granted. *Onchidium peronii* was likely confused with *P. verruculata* (unit #5), which our material indicates is present in Inhaca, or even *P. madagascariensis*, known from South Africa to western India (Fig. 6). The fact that the slugs were found on sand (Macnae and Kalk 1958: 128) could suggest that they saw *P. verruculata* (unit #5).

Solem (1959: 39) did not report any new material or localities for *P. peronii*. The references that he mentioned (e.g., Bretnall 1919; Hoffmann 1928) are already commented on above. His proposed distribution (“from the Red Sea and Mauritius to New Caledonia, Samoa, and the Marshall Islands”) is not fully accurate because it is based on the assumption that people never made any mistakes when identifying *P. peronii*, which is unfortunately not true. Solem (1959: 38) mentioned what he thought were the three “most obvious” of the “numerous differences” between *O. peronii* and *O. verruculatum*: distribution of branchial plumes (dorsal gills) on the notum, relative position of the pneumostome and the anus, and relative width of the hyponotum and pedal sole. But those features vary among individuals and should not be used for species identification.

Marcus and Marcus (1960: 877) described *Peronia peronii* from the Maldives based on eight specimens. Given that they report a maximum animal length of 155 mm, a long (4.5 mm) spine of the accessory penial gland, as well as a retractor muscle inserting near the heart, there is little doubt that they did examine *P. peronii* (Table 4). Later, Marcus and Marcus (1970: 213) added that they observed a retractor muscle inserting near the nerve ring in another of their specimens from the Maldives, which also is compatible with our present observations: a vestigial retractor muscle was even observed here in *P. peronii* (Table 4). Some of the material examined from historical museum collections for the present work also came from the Maldives (ANSP 304860).

Webb et al. (1969: 107–112) described copulatory mechanisms in specimens they identified as *O. peronii*. It is unclear from where those specimens were, possibly South Africa. At any rate, given that they illustrate a spine of the accessory penial gland which is only 1.4 mm long (Webb et al. 1969: 110, fig. 3), they did not examine individuals of *P. peronii*.

It is not possible to determine whether Marcus and Marcus (1970: 213) examined an individual of *Peronia peronii* from Madagascar because they do not provide the key features that characterize it. They could have seen a large individual of *P. madagascariensis* instead. Britton (1984: 183) merely mentioned the fact that Marcus and Marcus (1970) accepted only two valid species names (*P. peronii* and *P. verruculata*), which is not strictly exact because Marcus and Marcus (1970) did not address the nomenclatural status of *P. tongana* and did say that *P. branchifera* was close to *P. verruculata* but not that it was its synonym.

Patil and Kulkarni (2013) reported *Onchidium peronii* from Uran City, near Mumbai, India, but it is impossible to determine what species they saw (most likely it was *P. madgascariensis* or the unit #4 of *P. verruculata*, or both).

Many chemical studies have mentioned *P. peronii* in the past few decades. However, the name *P. peronii* was used arbitrarily. The individuals used for the extraction of natural products may not have been properly identified. Biskupiak and Ireland (1985) extracted peroniatriols from specimens identified as *P. peronii* from Guam. *Peronia peronii* is undeniably present in Guam. However, it is possible that *P. verruculata* (unit #1) could be present there as well. Pietra (1990: 145) mentioned peroniatriols in *Peronia peronii* from Micronesia where more than one species may be found. Arimoto et al. (1993) did not indicate where specimens of *P. peronii* and *O. verruculatum* were collected. In Japan, where the individuals used by Arimoto et al. (1993) possibly came from, there are four *Peronia* species which are all cryptic externally. Pietra (2002: 290) briefly cited peroniatriols in *P. peronii* based on the work by Arimoto et al. (1993). Finally, the antibacterial peptide extracted from individuals of *Peronia peronii* from the Persian Gulf (Bitaab et al. 2015) was most likely extracted from individuals of either *P. verruculata* (unit #4), or *P. madagascariensis*, or both (Fig. 6). The same general remark applies to ecological studies: Morrisey et al. (2010: 72) listed (with no justification for species identification) the presence of *Peronia peronii* in mangroves of the estuary of the Mtata River (31°57'S), South Africa; most likely, Morrisey et al. (2010: 72) encountered *P. madagascariensis* instead.

Finally, a few last words on *P. peronii* in phylogenetic studies. Dayrat et al. (2011: 428) and White et al. (2011: 4) identified a specimen from Guam (CASIZ 180486) as *Peronia peronii*, which is specimen [443] in the present study (Fig. 2). The specimen tentatively identified as *Peronia cf. peronii* from Mozambique (NHMUK 20060414) by Dayrat et al. (2011: 428) belongs to *P. madagascariensis*, which is specimen [735] in the present study (Fig. 2). The DNA sequences of the specimen from Guam were used again in several studies (e.g., Gaitán-Espitia et al. 2013; Harasewych et al. 2015).

##### Peronia okinawensis

Taxon classificationAnimaliaSystellommatophoraOnchidiidae

Dayrat & Goulding sp. nov.

http://zoobank.org/39BCC2F7-6530-4F13-8662-BDCF803C5452

Figs 17 , 18 , 19 , 20

###### Type material.

***Holotype*.** Japan • holotype, hereby designated, 27/25 mm [696-4 H]; Okinawa, Kunigami, Bay just SE of Cape Hedo; 26°51.803'N, 128°15.863'E; 3 Jul 2004; G Paulay, J Jeller, M Malay & Y Hiratsuka leg.; reef flat; UF 352288.

###### Additional material examined.

Japan • 2 specimens 25/20 mm [696-3] and 20/17 mm [696-2]; same collection data as for the holotype; UF 352288.

###### Distribution

(Fig. 6). Endemic to Okinawa, Japan.

###### Etymology.

*Peronia okinawensis* is named after its type locality: *okinawensis* is a latinized adjective that agrees in gender (feminine) with the generic name (ICZN 1999: Article 31.2).

###### Habitat.

The only specimens known were found on a reef flat. *Peronia okinawensis* seems to be rare compared to *P. verruculata* (unit #1) but may be more abundant at some other sites in Okinawa. It would be interesting, in the future, to map in detail at what exact sites the three *Peronia* species that are sympatric in Okinawa (*P. okinawensis*, *P. peronii*, and *P. verruculata*) overlap or not, in Okinawa and possibly in the rest of the Ryukyu Islands.

###### Color and morphology.

No picture of live animals is available. The color of preserved specimens is beige mottled with darker areas dorsally and whitish ventrally. The dorsal notum of live animals is covered by dozens of papillae of various sizes. Some papillae bear black dorsal eyes at their tip. The number of papillae with dorsal eyes is variable (8–15). The largest specimens are 27 mm long.

###### Digestive system

(Figs 17A, 18). Examples of radular formulae are presented in Table 5. The median cusp of the rachidian teeth is approximately 45 μm long. The hook of the lateral teeth is approximately 110 μm long. The intestinal loops are of type I, with a transitional loop oriented between 12 to 3 o’clock.

###### Reproductive system

(Figs 17B, C, 19, 20). In the anterior (male) parts, the muscular sac of the accessory penial gland is less than 15 mm long. The hollow spine of the accessory penial gland is narrow, elongated, and straight or slightly curved, and its shape (including at its tip) varies between individuals. Its length ranges from 1.8 mm ([696-3] UF 352288) to 2.3 mm ([696-4 H] UF 352288). Its diameter at the conical base ranges from 240 to 300 μm. Its diameter at the tip ranges from 115 to 150 μm. The retractor muscle is shorter or longer than the penial sheath and inserts near the heart. Inside the penial sheath, the penis is a narrow, elongated, soft, hollow tube. Its distal end bears conical hooks which are less than 35 μm long.

###### Diagnostic features

(Table 4). *Peronia okinawensis* is characterized by a unique combination of anatomical traits: muscular sac shorter than 15 mm, intestinal loops of type I (with a transitional loop oriented between 12 and 3 o’clock), retractor muscle inserting near the heart.

###### Remarks.

A new species name is needed because no existing name applies to the species described here. The specimen [696-2] was tentatively identified as Peronia cf. verruculata by Dayrat et al. (2011). This identification should be disregarded because the specimen [696-2] belongs to the species described here (Figs 2–4). *Peronia okinawensis* is one of the four *Peronia* species in Japanese waters (Fig. 6). For a comparison of their geographic range, see remarks on *P. setoensis*. For their identification, see the identification key as well as Table 4. It is possible that *P. okinawensis* is not strictly endemic to Okinawa.

##### Peronia madagascariensis

Taxon classificationAnimaliaSystellommatophoraOnchidiidae

(Labbé, 1934a)

**Figure 22.**
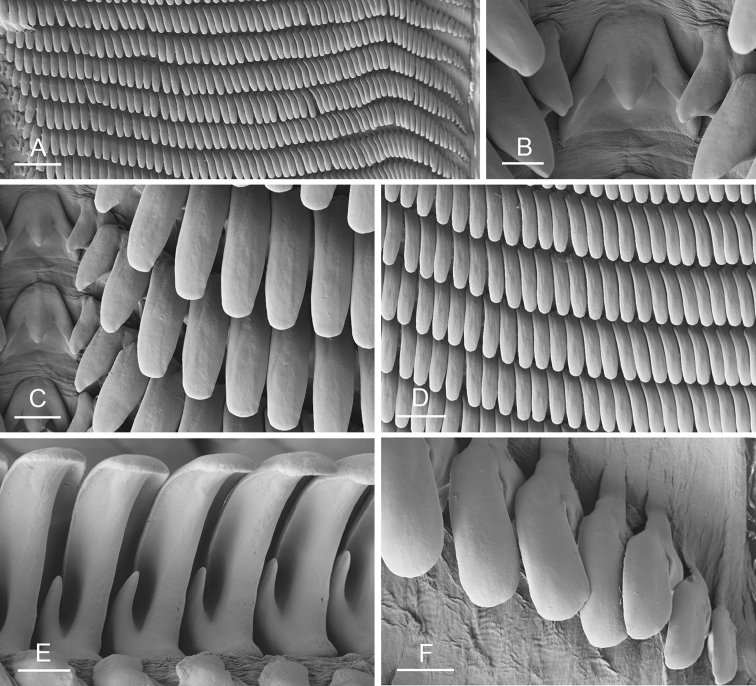
Radula, *Peronia madagascariensis*, South Africa, [5841] (NMSA W7547) **A** right half rows of teeth **B** rachidian and innermost lateral teeth **C** rachidian and innermost lateral teeth **D** lateral teeth **E** lateral teeth, frontal view **F** outermost lateral teeth. Scale bars: 200 μm (**A**), 20 μm (**B, E, F**), 40 μm (**C**), 100 μm (**D**).

**Figure 23.**
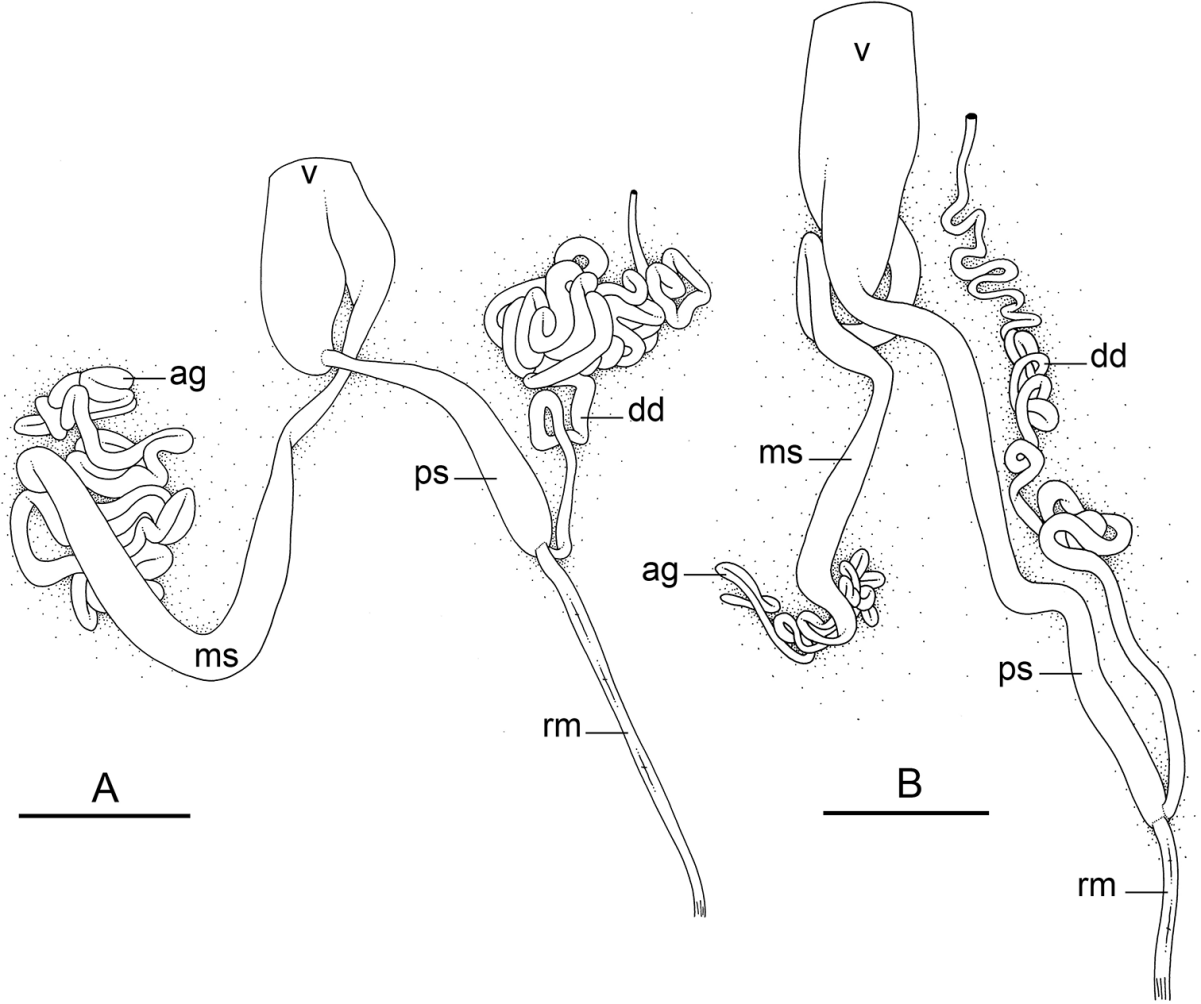
Anterior, male, copulatory apparatus, *Peronia madagascariensis***A** Madagascar, [5501] (MNHN-IM-2009-16392) **B** South Africa, [5841] (NMSA W7547). Scale bars: 5 mm (**A**), 2 mm (**B**). Abbreviations: ag accessory penial gland, dd deferent duct, ms muscular sac, ps penial sheath, rm retractor muscle, v vestibule.

**Figure 24.**
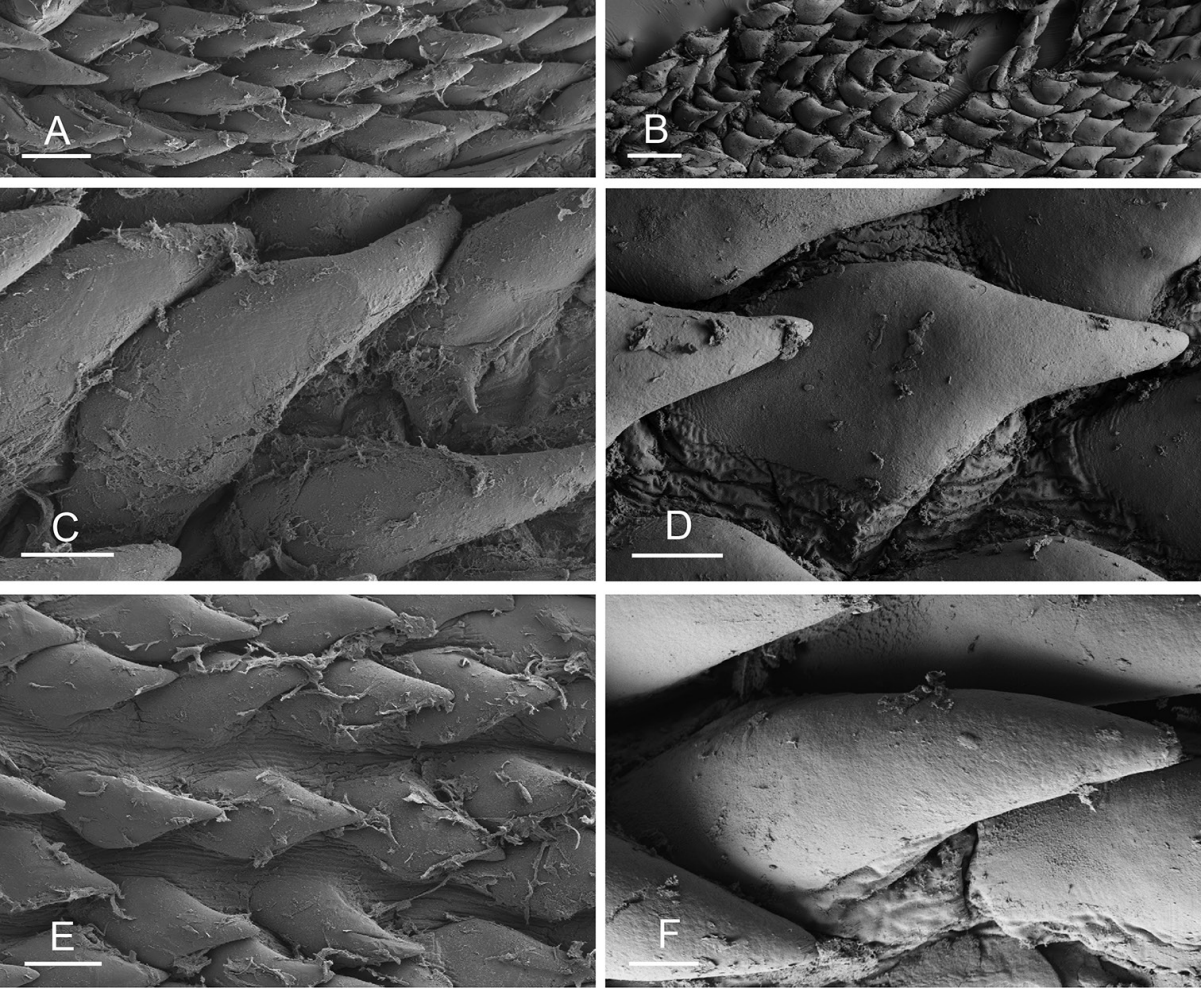
Penial hooks, *Peronia madagascariensis*, Madagascar **A, C** [5500] (MNHN-IM-2009-16391) **B, D** [5504] (MNHN-IM-2009-16412) **E** [5506] (MNHN-IM-2009-16418) **F** [5501] (MNHN-IM-2009-16392). Scale bars: 60 μm (**A**), 100 μm (**B**), 20 μm (**C, D**), 40 μm (**E**), 10 μm (**F**).

**Figure 25.**
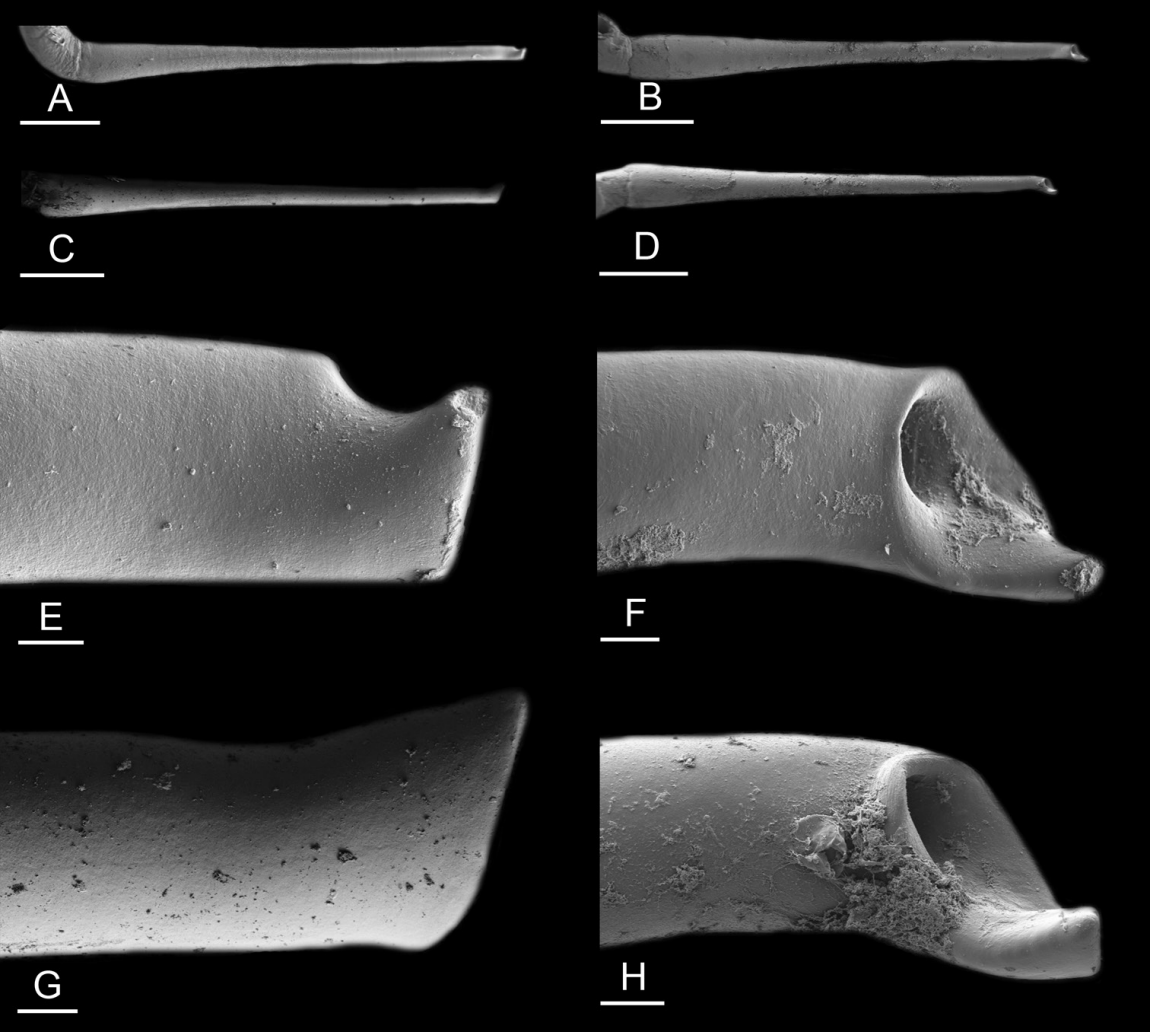
Accessory penial gland spine, *Peronia madagascariensis*, Madagascar **A, E** [5500] (MNHN-IM-2009-16391) **B, F** [5502] (MNHN-IM-2009-16393) **C, G** [5504] (MNHN-IM-2009-16412) **D, H** [5506] (MNHN-IM-2009-16418). Scale bars: 400 μm (**A–D**), 20 μm (**E–H**).

Figs 21 , 22 , 23 , 24 , 25

Paraperonia madagascariensis Labbé, 1934a: 199, fig. 15.
Paraperonia jousseaumei Labbé, 1934a: 198, figs 12–14. Syn. nov.

###### Type material.

***Holotype*** (*Paraperonia madagascariensis*). Madagascar • holotype, by monotypy, 40/40 mm; Fort Dauphin [Taolagnaro]; 1932; Décary leg.; MNHN-IM-2000-33680. Originally, no jar clearly labeled as the type material of *P. madagascariensis* was found at the MNHN, but the holotype could be traced back. The original description of *P. madagascariensis* is based on a single individual (40/38 mm) from Fort-Dauphin collected by Décary (the French botanist Raymond Décary [1891–1973]) in 1932. Only one old jar was found at the MNHN with a specimen collected from Fort-Dauphin (MNHN-IM-2000-33680). The information on the label (specimen collected by Décary in 1932) matches the information provided in Labbé’s original description of *P. madagascariensis*, and even the specimen size matches. Therefore, that specimen is considered to be the holotype by monotypy of *P. madagascariensis*. The holotype was dissected by Labbé. The radula, the posterior (hermaphroditic) reproductive parts, and the male parts are all missing. The intestinal loops are of type V (Fig. 21A).

***Syntypes*** (*Paraperonia jousseaumei*). The type material of *Paraperonia jousseaumei* could not be located at the MNHN. The original description of *P. jousseaumei* was based on ten individuals (45/38 to 40/30 mm) from the Red Sea (“Mer Rouge”) collected by Jousseaume in 1892. Only two old jars were found at the MNHN with that collecting information. One of them contains specimens that are part of the type series of *P. gondwanae* because the specific name “*gondwanae*” is written on an old label (MNHN-IM-2000-33683). The three labels of the other jar (MNHN-IM-2014-7993) say: “Peronia Mer Rouge Mr Jousseaume n°15, 1892,” “Oncidium [written over “Oncidiella”] peronii Cuvier Mer Rouge M. Jousseaume n°15-1892,” and, for unknown reasons, “60.” This jar contains six specimens of *Peronia*, from 60/45 to 25/15 mm, two of which were dissected, possibly by Labbé. The intestinal loops of the two dissected specimens are of type I and thus are not in agreement with Labbé’s (1934a: fig. 12) original illustration of the intestinal loops of type V in *P. jousseaumei*. Also, the sizes and the number of individuals do not match the original description of *P. jousseaumei*. Those specimens could possibly be some of the eight non-type specimens that Labbé (1934a: 190) mentioned in his re-description of *Peronia peronii* collected by Jousseaume from the Red Sea (“Mer Rouge”) in “1852” (likely a mistake for 1892). Given that Labbé does not specify their size, it is not possible to know to what species Labbé thought those specimens belong exactly (MNHN-IM-2014-7993).

###### Additional material examined.

South Africa • 2 specimens 35/23 mm [5841] and 18/13 mm [5842]; KwaZulu-Natal, Durban, Treasure Beach; 29°57.294'S, 30°59.514'E; 18 Nov 2010; D Herbert and L Davis leg.; rocky intertidal zone; NMSA W7547.

Mozambique • 1 specimen 42/37 mm [735]; Cabo Delgado Province, Pemba, Wimbi Beach, Pemba Beach Hotel; 12°58'S, 40°32'E; 14 Jul 2006; DG Reid leg.; on shady rock at base of limestone cliff, in upper eulittoral behind intertidal platform; NHMUK 20060414.

Madagascar • 1 specimen 55/40 mm [5500]; Ambatobe, près Soamanitse; 25°27.4'S, 44°57.4'E; 24 May & 7 Jun 2010; MNHN Expedition Atimo Vatae leg.; st BM02, 0–1 m; MNHN-IM-2009-16391. • 1 specimen 40/35 mm [5504]; same collection data as for the preceding; MNHN-IM-2009-16412. • 1 specimen 40/30 mm [5501]; Ambatomainty; 25°26.3'S, 44°56.5'E; 25 May 2010; MNHN Expedition Atimo Vatae leg.; st BM03, 0–1 m; MNHN-IM-2009-16392. • 1 specimen 55/40 mm [5502]; same collection data as for the preceding; MNHN-IM-2009-16393. • 1 specimen 55/40 mm [5503]; Ambatobe, Bavarama; 25°27.9'S, 44°57.6'E; 28 & 29 May 2010; MNHN Expedition Atimo Vatae leg.; st BM06, 0–1 m; MNHN-IM-2009-16396. • 1 specimen 40/35 mm [5506]; same collection data as for the preceding; MNHN-IM-2009-16418.

Oman • 1 specimen 10/7 [703]; Muscat, Cemetery Bay; 23°37.250'N, 58°36.016'E; 9 Feb 2004; G Paulay & M Claereboudt leg.; coral community, reef slope, on ophiolitic bedrock and rubble; UF 332088.

###### Additional material examined

**(historical museum collections).** Oman • 3 specimens 80/60 mm; Qurm Beach, near Muscat; 23°37.56'N, 58°28.86'E; 26 Jan 2005; V Bonito, M Claereboudt & G Paulay leg.; intertidal rocky shore; UF 368019.

Iran • 3 specimens 80/65 mm to 75/65 mm; Persian Gulf, Strait of Hormuz, Qeshm Island; 18 Apr 1937; G Thorson leg.; st 69; NHMD 635302.

Yemen • 1 specimen 55/55 mm; Socotra, off Quadub; 12°39.015'N, 53°55.730'E; 18 Mar 1999; Salim Al-Moghrabi (from N Yonow’s personal collection) leg.; intertidal, ST-064 SAM-1; SMF 358305.

South Africa • 1 specimen 70/45 mm; Port Natal, Durban; 30S, 31E; Wahlberg leg.; littoral rocky bottom; SMNH 180711.

###### GenBank and BOLD sequences.

One COI sequence was obtained from BOLD (LGEN099-14) for an individual identified as *Onchidium verruculatum* and collected from Dwarka, Gujarat, on the western coast of India (ca. 22°N), which is the easternmost known locality for *P. madagascariensis*. A second COI sequence was obtained from GenBank (LC027608) for an individual identified as *Peronia* sp. and collected from the coast of Iran in the Persian Gulf. Both sequences were unpublished.

###### Distribution

(Fig. 6). From South Africa to the Red Sea and western India (ca. 22°N): South Africa, Mozambique, Madagascar (type locality of *P. madagascariensis*), Gulf of Oman, Iran (Strait of Hormuz), Yemen (Socotra), India (Mumbai, Gujarat), Red Sea (type locality of *P. jousseaumei*). All records are new except for the type locality in Madagascar. *Peronia madagascariensis* is, so far, not present in Mauritius.

###### Etymology.

*Peronia madagascariensis* was named after its type locality, Madagascar. *Peronia jousseaumei* was named after Félix Pierre Jousseaume [1835–1921], a medical doctor and malacologist who collected many specimens from the Red Sea preserved at the MNHN and which Labbé (1934a) studied for his monograph on onchidiids.

###### Habitat.

*Peronia madagascariensis* is found in the rocky intertidal, like most other *Peronia* slugs.

###### Color and morphology.

No picture of live animals was available. The color of preserved specimens is not different from other species (greyish brown and mottled with darker and lighter areas dorsally, and light brown greyish ventrally). The dorsal notum of live animals is covered by dozens of papillae of various sizes. In large individuals, dorsal papillae can be particularly tall (easily up to 4 mm), even in preserved specimens, and are evenly distributed over the entire notum. Preserved, they are very difficult to distinguish from retracted dorsal gills in the posterior half of the notum, but they are regular papillae with or without eyes. Some papillae bear black dorsal eyes at their tip. The number of papillae with dorsal eyes is variable (from 12 to 18). Dorsal gills seem taller and denser than in other species. The largest specimens in our fresh material are 55 mm long but two additional museum specimens are much longer (80 mm).

###### Digestive system

(Figs 21A–D, 22). Examples of radular formulae are presented in Table 5. The median cusp of the rachidian teeth is approximately 55 μm long. The hook of the lateral teeth is approximately 100 to 130 μm long. The intestinal loops are of type V.

###### Reproductive system

(Figs 21E, 23–25). In the anterior (male) parts, the muscular sac of the accessory penial gland is less than 15 mm long. The hollow spine of the accessory penial gland is narrow, elongated, and straight or slightly curved, and its shape (including at its tip) varies between individuals. Its length ranges from 2 mm ([5502] MNHN-IM-2009-16393) to 2.4 mm ([5500] MNHN-IM-2009-16391). Its diameter at the conical base ranges from 200 to 230 μm. Its diameter at the tip ranges from 70 to 80 μm. The retractor muscle is shorter or longer than the penial sheath and inserts near the heart. Inside the penial sheath, the penis is a narrow, elongated, soft, hollow tube. Its distal end bears conical hooks which are less than 100 μm long.

###### Diagnostic features

(Table 4). *Peronia madagascariensis* is characterized by a unique combination of two anatomical traits: intestinal loops of type V and a spine of the accessory penial gland longer than 2 mm.

###### Remarks.

The name *Paraperonia madagascariensis* clearly applies to a *Peronia* species because of the dorsal gills on the notum of the holotype. The holotype was entirely dissected by Labbé. The radula, the posterior (hermaphroditic) reproductive parts, and the anterior copulatory apparatus are missing. The intestinal loops are of type V (Fig. 21A), as illustrated by Labbé (1934a: fig. 17). The name *Peronia madagascariensis* applies to the species described here because it is, according to our molecular data, the only *Peronia* species with intestinal loops of type V along the eastern African coast, from South Africa to the Persian Gulf and western India, including Madagascar. Note that some of our fresh material was collected only 150 km east of the type locality in southern Madagascar. Some internal characters described by Labbé (1934a: 199) could not be verified on the holotype because most internal parts are missing, but they are similar to the species described here. In particular, the length of the spine of the accessory penial gland (2 mm) is compatible with what was observed in our material.

Additional, non-type specimens were found in historical museum collections which could be identified as *P. madagascariensis* due to the presence of intestinal loops of type V, from Oman (UF 368019), the Strait of Hormuz (NHMD 635302), and Socotra (SMF 358305). Those localities, however, are all already included within the known distribution of *P. madagascariensis* based on our DNA sequences, as the Strait of Hormuz is very close to the Gulf of Oman. Finally, one of the “*a*” paralectotypes of Labbé’s (1934a: 199) *Paraperonia gondwanae* from Bombay (MNHN-IM-2000-33682), with intestinal loops of type V (Fig. 21B), belongs to *P. madagascariensis*. Note that two of those museum specimens are longer (80 mm) than our fresh material (less than 55 mm).

*Peronia* slugs with intestinal loops of type V are without doubt present in the Red Sea. For instance, one of the “*c*” paralectotypes of Labbé’s (1934a: 200) *Paraperonia gondwanae* from Suez (MNHN-IM-2000-33683) is characterized by intestinal loops of type V (Fig. 21C), which means that it does not belong to *P. verruculata* (characterized by intestinal loops of type I). Labbé’s (1934a)*Paraperonia jousseaumei*, with the Red Sea as type locality, is also characterized by intestinal loops of type V. Even though the type material of *P. jousseaumei* could not be located at the MNHN, Labbé’s (1934a: fig. 12) drawing of the internal anatomy of *P. jousseaumei* clearly illustrates intestinal loops of type V. Given that *P. madagascariensis* is widespread from South Africa all the way to western India, including the Strait of Hormuz, it is accepted here that it also is distributed in the Red Sea. That, however, will still need to be confirmed with fresh material from both the Red Sea and the Gulf of Aden. If it appears that the populations of *Peronia* slugs with intestinal loops of type V from the Red Sea are a distinct species, then the name *P. jousseaumei* could apply to them and be valid. Finally, given that *P. madagascariensis* is present in the Strait of Hormuz, it most likely also is distributed in the rest of the Persian Gulf, which hopefully will be confirmed at some point with fresh material.

Even though the names *Peronia madagascariensis* and *Peronia jousseaumei* were never used prior to the present contribution, they are not regarded as new combinations because *Paraperonia* has already been regarded as a synonym of *Peronia* by Britton (1984: 182) and because it has also been made clear that the genus *Peronia* included all species of slugs with dorsal gills (e.g., Dayrat et al. 2017: 1861).

The specimen [703] from Oman was tentatively identified as *Peronia* sp. 2 by Dayrat et al. (2011) but it clearly belongs to *P. madagascariensis* (Fig. 2). Also, note that its COI sequence was resubmitted to GenBank because the old one (GenBank HQ660044) was inaccurate. The specimen [735] from Mozambique was tentatively identified as Peronia cf. peronii by Dayrat et al. (2011). This identification should be disregarded because the specimen [735] belongs to *P. madagascariensis* (Fig. 2).

A specimen from Durban (30°S), South Africa, preserved in Stockholm (SMNH 180711) identified as *O. verruculatum* by Hoffmann (1928: 44, 73) is identified here as *P. madagascariensis* because of its intestinal loops of type V (Table 4). Various records of *Onchidium peronii*, *O. savignyi*, and *Onchidium verruculatum* from Natal, South Africa (Krauss 1848: 72; Sturany 1898: 73; Collinge 1910: 171–172; Connolly 1912: 224–225, 1939: 454; Webb 1969) most likely are records of *Peronia madagascariensis*, although *P. verruculata* (unit #5) could also be present in northeastern South Africa because it is known in Maputo, southern Mozambique (ca. 26°S).

##### Peronia platei

Taxon classificationAnimaliaSystellommatophoraOnchidiidae

(Hoffmann, 1928)

**Figure 26.**
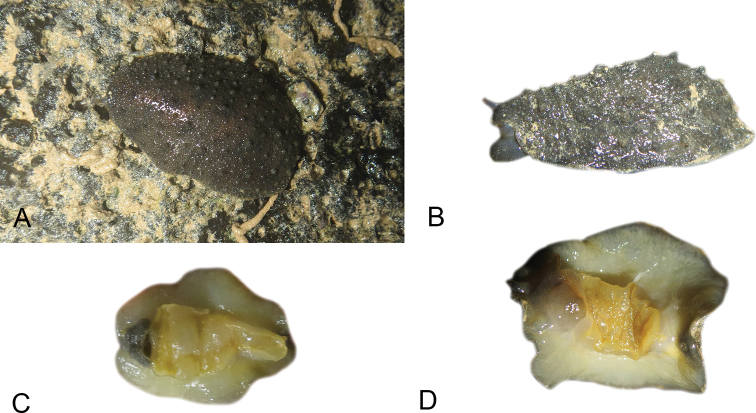
Live animals, *Peronia platei*, Hawaii, Oahu **A** dorsal view, 30 mm long [6161] (BPBM 284528) **B** dorsal view, 30 mm long [6160] (BPBM 284527) **C** ventral view, same as **A**; **D** ventral view, same as **B**.

**Figure 27.**
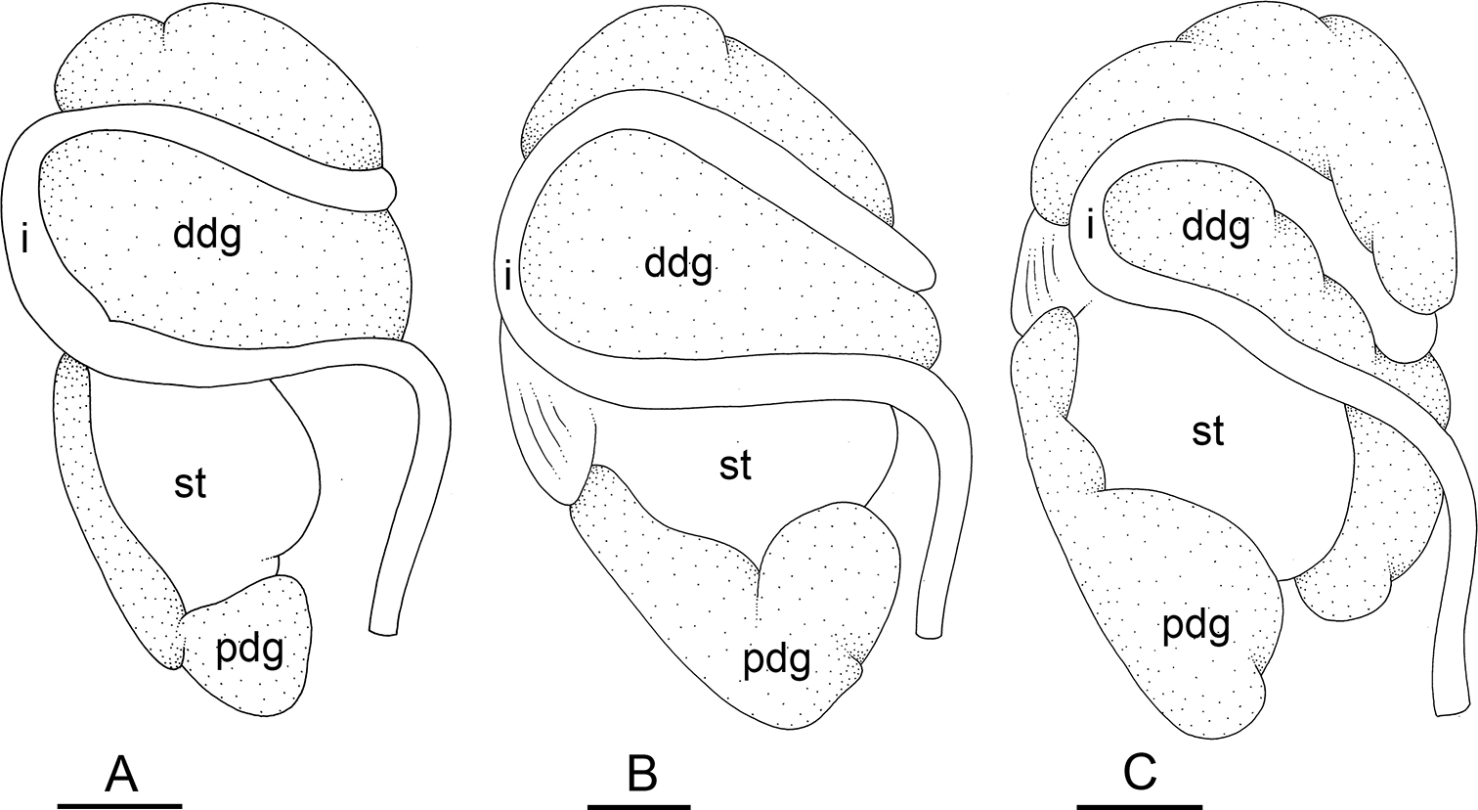
Digestive system, dorsal view, *Peronia platei*, with intestinal loops of type V **A** lectotype, French Polynesia, Moorea (SMNH-Type-7537) **B** Hawaii, Oahu [6160] (BPBM 284527) **C** Papua New Guinea, Madang [5412] (MNHN-IM-2013-13351). Scale bars: 2 mm (**A–C**). Abbreviations: ddg dorsal digestive gland, i intestine, pdg posterior digestive gland, st stomach.

**Figure 28.**
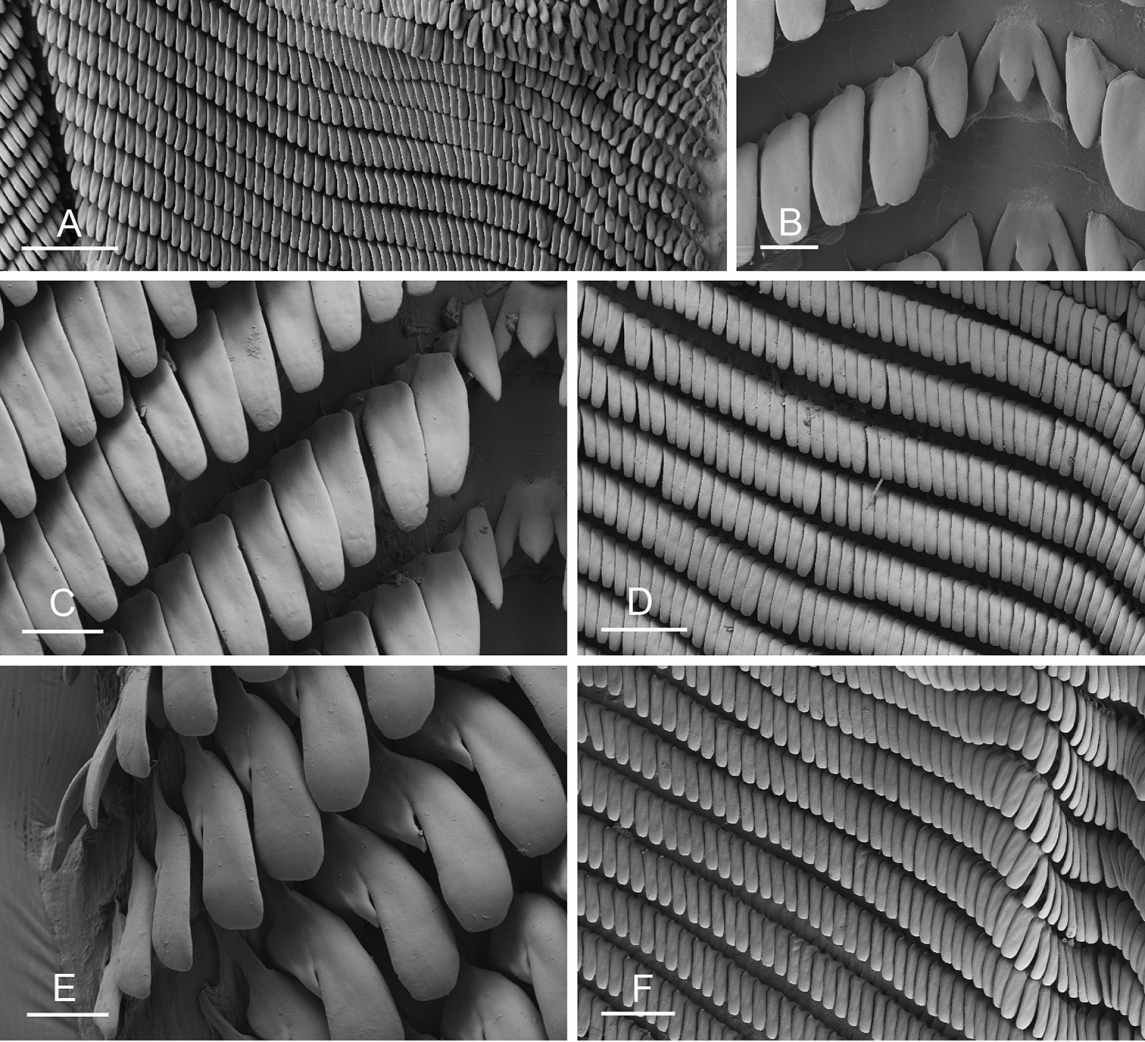
Radula, *Peronia platei***A** Papua New Guinea, Madang [5405] (MNHN-IM-2013-13762) **B** Hawaii, Oahu [6161] (BPBM 284528) **C–E** Hawaii, Molokai [706] (UF 303653) **F** [5380] (UF 303653) **A** right half rows of teeth **B** rachidian and innermost lateral teeth **C** rachidian and innermost lateral teeth **D** lateral teeth **E** outermost lateral teeth **F** lateral teeth. Scale bars: 200 μm (**A**), 20 μm (**B, E**), 30 μm (**C**), 100 μm (**D**), 80 μm (**F**).

**Figure 29.**
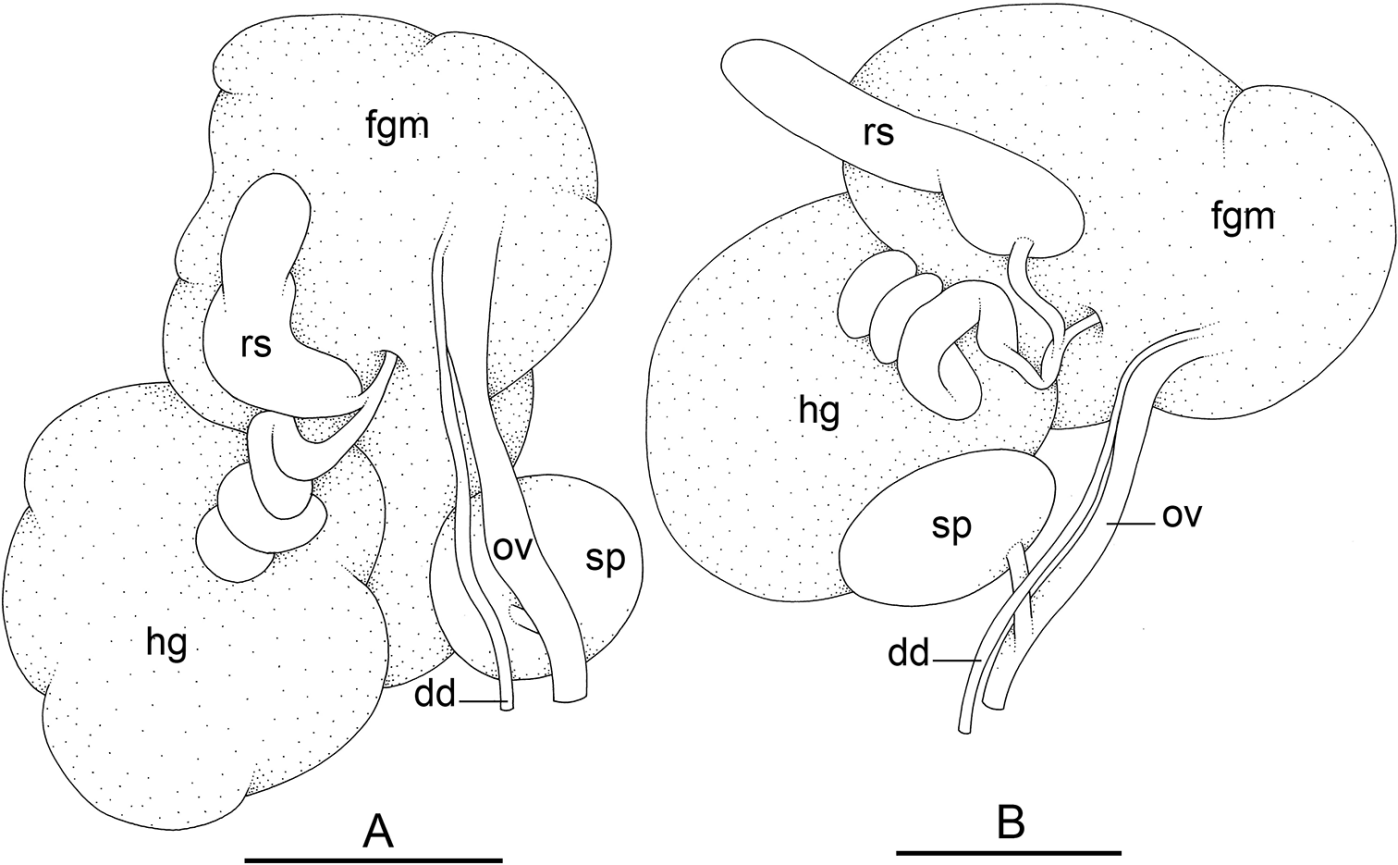
Posterior, hermaphroditic (female) reproductive system, *Peronia platei***A** Papua New Guinea, Madang [5412] (MNHN-IM-2013-13351) **B** Hawaii, Oahu [6160] (BPBM 284527). Scale bars: 3 mm (**A, B**). Abbreviations: dd deferent duct, fgm female gland mass, hg hermaphroditic gland, ov oviduct, rs receptaculum seminis, sp spermatheca.

**Figure 30.**
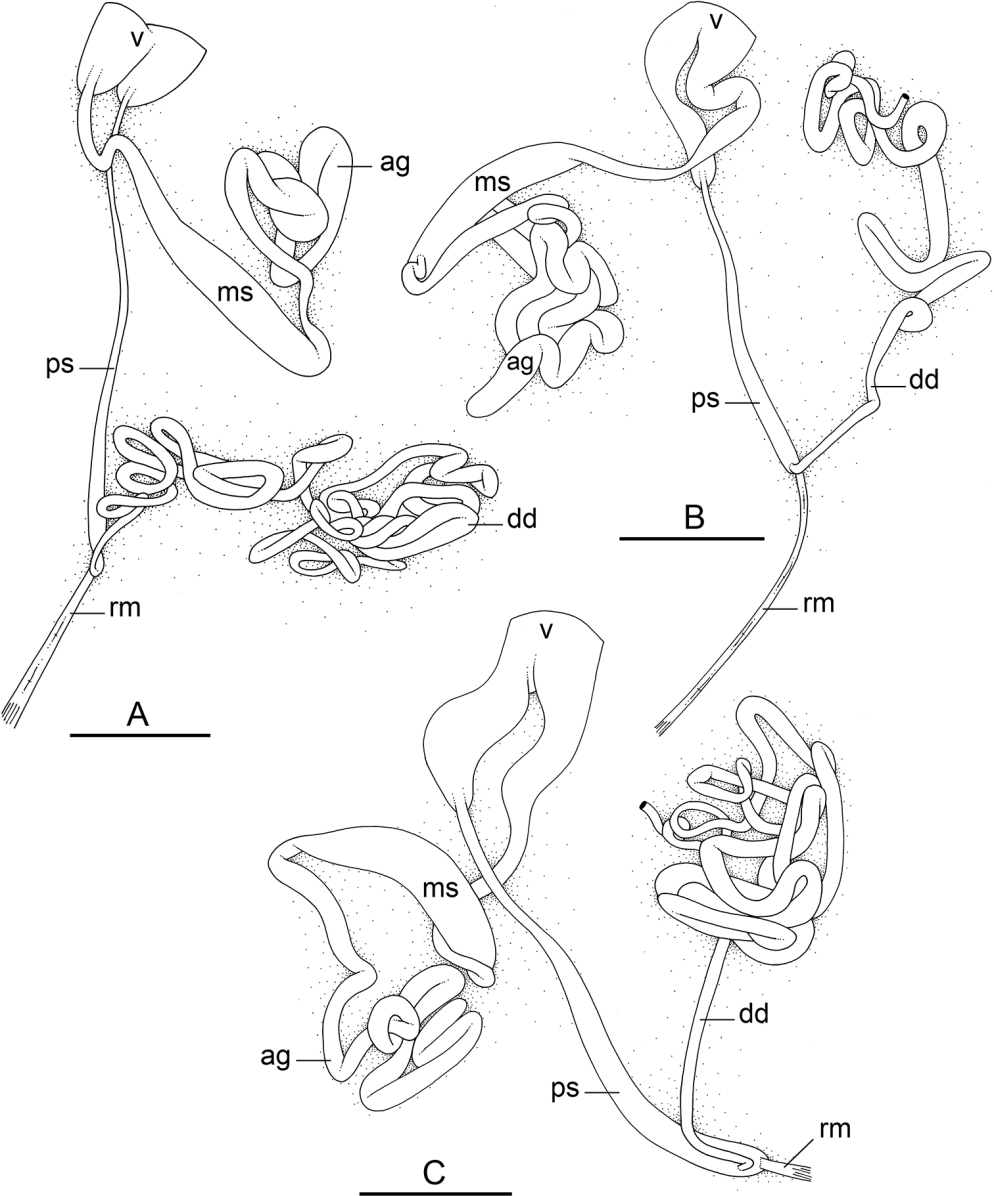
Anterior, male, copulatory apparatus, *Peronia platei***A** lectotype, French Polynesia, Moorea (SMNH-Type-7537) **B** Papua New Guinea, Madang [5412] (MNHN-IM-2013-13351) **C** Hawaii [706] (UF 303653). Scale bars: 5 mm (**A**), 3 mm (**B**), 2 mm (**C**). Abbreviations: ag accessory penial gland, dd deferent duct, ms muscular sac, ps penial sheath, rm retractor muscle, v vestibule.

**Figure 31.**
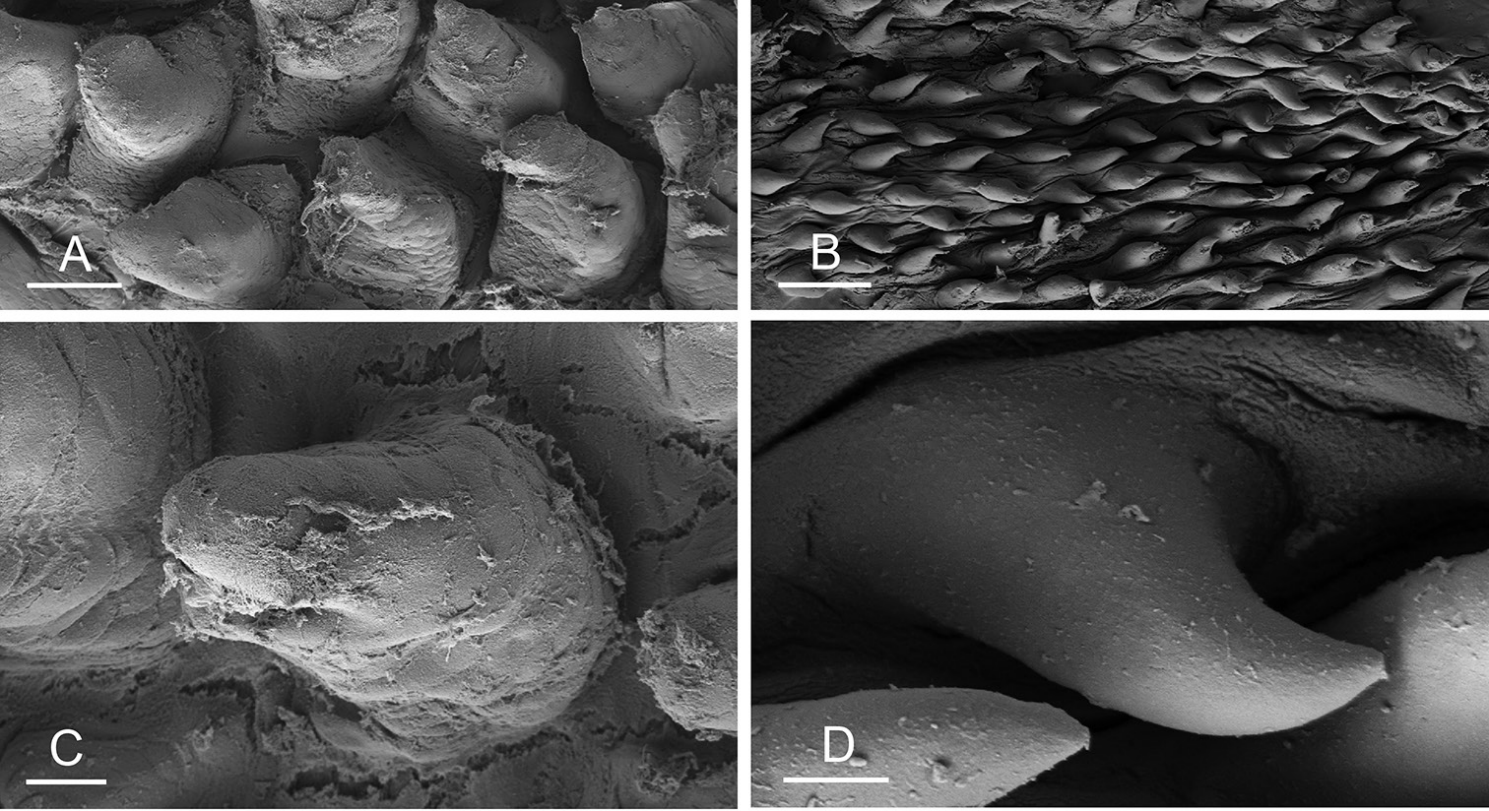
Penial hooks, *Peronia platei***A, C** Hawaii, Oahu [6161] (BPBM 284528) **B, D** Papua New Guinea, Madang [5412] (MNHN-IM-2013-13351). Scale bars: 40 μm (**A**), 20 μm (**B**), 10 μm (**C**), 2 μm (**D**).

**Figure 32.**
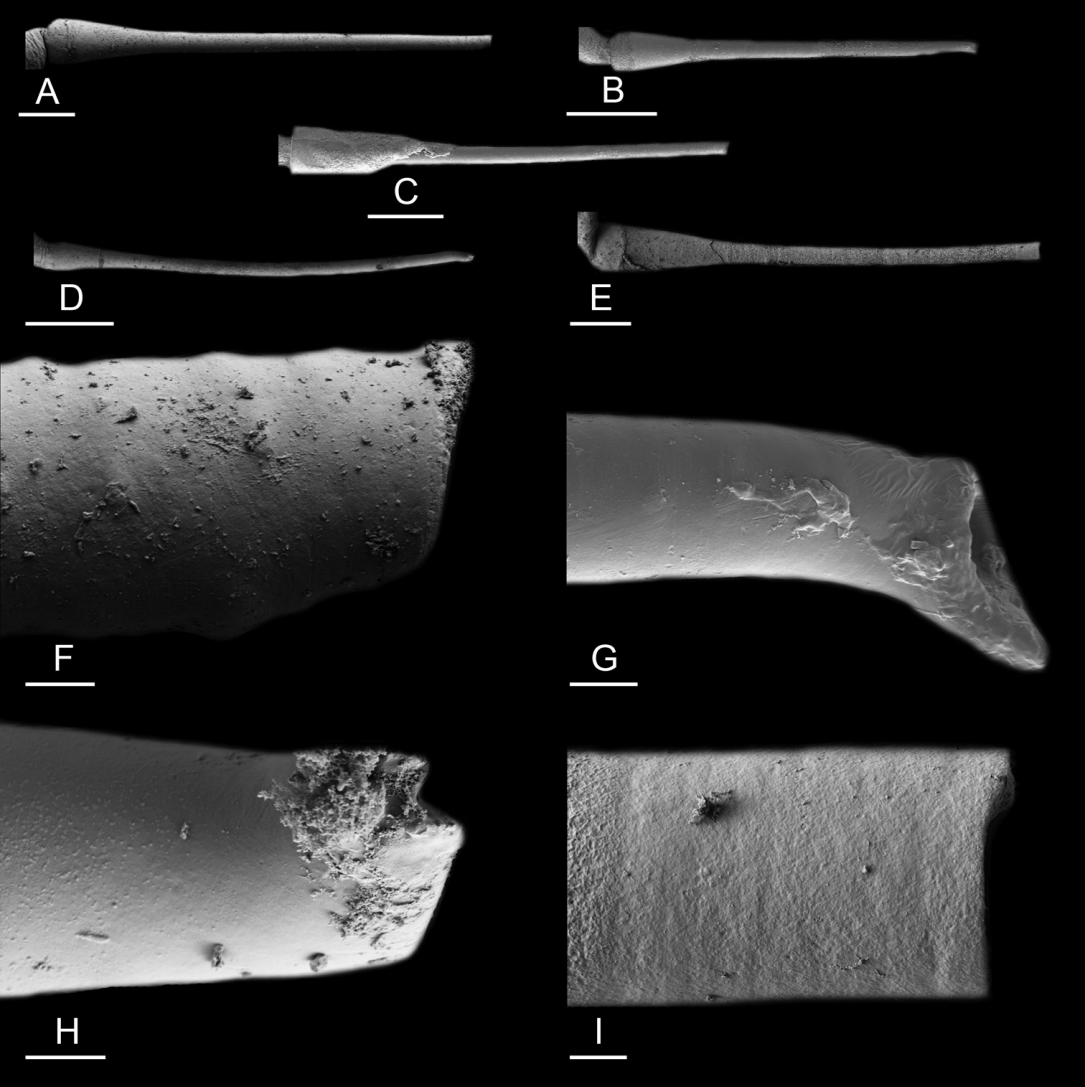
Accessory penial gland spine, *Peronia platei***A–C, F, G** Hawaii, Oahu **D, E, H, I** Papua New Guinea, Madang **A** [706] (UF 303653) **B** [6160] (BPBM 284527) **C** [6161] (BPBM 284528) **D** [5412] (MNHN-IM-2013-13351) **E** [5405] (MNHN-IM-2013-13762) **F** same as **A**; **G** same as **B**; **H** same as **D**; **I** same as **E**. Scale bars: 100 μm (**A, E**), 200 μm (**B, D**), 150 μm (**C**), 6 μm (**F, H, I**), 10 μm (**G**).

Figs 26 , 27 , 28 , 29 , 30 , 31 , 32

Onchidium platei Hoffmann, 1928: 51–53, figs 9, 10, pl. 3, figs 11, 12.

###### Type material.

***Lectotype* and *paralectotypes***. French Polynesia • lectotype, hereby designated, 18/10 mm; Eimeo [Moorea], Tahiti; 1851–1853; Eugenie Expedition leg.; st 1245–9, in the barrier reef; SMNH-Type-7537. • 2 paralectotypes, 16/10 mm and 16/10 mm; same collection data as for the lectotype; SMNH-Type-7537. • 4 paralectotypes, 17/10 mm, 16/12 mm, 15/11 mm, and 10/7 mm; Tahiti; Dec 1846; Reinhardt, Galathea Expedition 470 leg.; NHMD 613754. • 1 paralectotype, 7/5 mm; Tahiti; Reinhardt, Galathea Expedition 471 leg.; NHMD 613755. • 1 paralectotype, 15/10 mm; Tahiti; Reinhardt, Galathea Expedition 472 leg.; NHMD 613756.

###### Additional material examined.

Hawaii • 2 specimens 12/10 mm [706] and 12/12 mm [5380]; Molokai, Puko’o; 21°04.313'N, 156°48.001'W; 27 Jan 2003; V Bonito leg.; on rocks; UF 303653. • Oahu, Ala Moana Beach Park; 21°17.158'N, 157°50.827'W; 1 specimen 30/20 mm [6160]; 7 Oct 2018; TC Goulding leg.; st 264, intertidal rocks, night tide; BPBM 284527. • 1 specimen 30/20 mm [6161]; same collection data as for the preceding; BPBM 284528.

Papua New Guinea – **Madang** • 1 specimen 14/12 mm [5405]; Rempi Area, SW Hargun Island; 05°01.6'S, 145°47.9'E; 15 & 20 Nov 2012; MNHN Expedition Papua Niugini leg.; st PM24, night tide; MNHN-IM-2013-13762. • 1 specimen 20/17 mm [5412]; Rempi Area, Barag Island; 05°01.1'S, 145°47.9'E; 15 Nov 2012; MNHN Expedition Papua Niugini leg.; st PM25, fringing reef on narrow barrier island; MNHN-IM-2013-13351. • 1 specimen 12/10 mm [5410]; Riwo Waters; 05°08.9'S, 145°48.2'E; 26 Nov 2012; MNHN Expedition Papua Niugini leg.; st PM40, sandy beach and intertidal rocks; MNHN-IM-2013-15765. • 1 specimen 14/12 mm [5464]; Wonad Island; 05°08.1'S, 145°49.3'E; 27 Nov 2012; MNHN Expedition Papua Niugini leg.; st PM41, sandy beach and intertidal rocks; MNHN-IM-2013-15871.

###### Additional material examined

**(historical museum collections).** French Polynesia • 2 specimens 15/10 mm and 13/8 mm; Tuamotu Archipelago, NE side, Anaa Atoll; 17°20'S, 145°30'W; 27 Oct 1967; NGS-SBM Marquesa Expedition MV “Pele” 1967 leg.; WAM S26717. • 2 specimens 7/5 mm and 5/4 mm; Tuamotu Archipelago, Marutea Atoll; 17S, 143°10.02'E; Aug 1903; LG Seurat leg.; AM C.17073.

Hawaii • 15 specimens from 18/15 mm to 8/8 mm; Oahu, Kailua Bay, Mokapu Point; 21°28.02'N, 157°43.98'E; WF Ponder and EA Kay leg.; 7 Apr 1974; on rocks, semi-sheltered and exposed platforms; AM C.214245.

Kiribati • 8 specimens from 10/9 mm to 3/3 mm; Gilbert Islands, Apamama [Abemama]; 00S, 173E; 1917–1918; S Bock’s Pacific Expedition leg.; sand, inside lagoon; SMNH 106488.

###### Distribution

(Fig. 6). West Pacific: Papua New Guinea, French Polynesia (Tuamotu and Tahiti), Kiribati, and Hawaii. All records are new except for the type locality in Tahiti.

###### Etymology.

*Peronia platei* was named after German zoologist Ludwig Hermann Plate [1862–1937], professor of zoology at the University of Jena and author of a monograph on onchidiids (Plate 1893).

###### Habitat.

*Peronia platei* is found primarily in the rocky intertidal. According to the label, specimens from Kiribati were collected on sand inside a lagoon (*P. sydneyensis* and *P. willani* are also known to be found on sand).

###### Color and morphology of live animals

(Fig. 26). No picture of live animals was available for specimens from the West Pacific. The description of the color of live animals is based on Hawaii individuals. The dorsal notum is uniformly very dark grey, almost black, including papillae. The hyponotum is light yellowish. The foot is light yellowish to orange. The ocular tentacles are grey, like the head. The dorsal notum of live animals is covered by dozens of papillae of various sizes. Some papillae bear black dorsal eyes at their tip. The number of papillae with dorsal eyes is variable (from 7 to 10). The papillae with dorsal eyes cannot be counted in specimens from Hawaii because the notum is too dark and because eye pigmentation tends to fade in preservation. The largest specimens are 30 mm long in Hawaii and 20 mm in Papua New Guinea.

###### Digestive system

(Figs 27, 28). Examples of radular formulae are presented in Table 5. The median cusp of the rachidian teeth is approximately 30 to 35 μm long. The hook of the lateral teeth is approximately 60 to 90 μm long. The intestinal loops are of type V.

###### Reproductive system

(Figs 29–32). In the posterior (hermaphroditic) parts, the deferent duct and the oviduct are straight. In the anterior (male) parts, the muscular sac of the accessory penial gland is less than 5 mm long. The hollow spine of the accessory penial gland is narrow, elongated, and straight or slightly curved, and its shape (including at its tip) varies between individuals. Its length ranges from 0.8 mm ([706] UF 303653) to 0.9 mm ([6161] BPBM 284528) in Hawaii and from 0.7 mm ([5405] MNHN-IM-2013-13762) to 1 mm ([5412] MNHN-IM-2013-13351) in Papua New Guinea. Its diameter at the conical base ranges from 95 to 100 μm (Hawaii) and from 65 to 80 μm (Papua New Guinea). Its diameter at the tip ranges from 25 to 30 μm (Hawaii) and from 20 to 30 μm (Papua New Guinea). The retractor muscle is shorter or longer than the penial sheath and inserts at the posterior end of the visceral cavity. Inside the penial sheath, the penis is a narrow, elongated, soft, hollow tube. Its distal end bears conical hooks which are less than 60 μm long in Hawaii and less than 20 μm long in Papua New Guinea.

###### Diagnostic features

(Table 4). *Peronia platei* is cryptic with *P. setoensis*. Both species share the same combination of anatomical traits: intestinal loops of type V, retractor muscle inserting at the posterior end of the visceral cavity, a spine of the accessory penial gland from 0.8 to 1 mm long (*P. platei*) and from 0.9 to 1.2 mm long (*P. setoensis*). The diameter of the spine of the accessory penial gland at its tip is larger in *P. platei* (25 to 30 μm) than in *P. setoensis* (less than 25 μm) but that may be simply due to limited sampling. *Peronia platei* and *P. setoensis* are both distributed in the West Pacific but they are not sympatric based on current data (Fig. 6).

###### Remarks.

*Onchidium platei* applies to the species described here because the anatomy of the lectotype is identical to the anatomy of our material (Table 4): gills on the dorsal notum; muscular sac of the accessory penial gland less than 5 mm long; spine of the accessory penial gland 0.9 mm long (observed by transparency); intestinal loops of type V; seven dorsal papillae with eyes. Our molecular analyses show that the species described here is widespread across the West Pacific, from Papua New Guinea to Hawaii. There is no reason to think that the populations in French Polynesia (type locality of *O. platei*) are a distinct species. This, however, will have to be confirmed with fresh material from French Polynesia, preferably from Moorea, the type locality. All eight paralectotypes (also from Tahiti) also belong to the same species.

Hoffmann’s (1928: 51–53, figs 9, 10, pl. 3, figs 11, 12) original description, which is quite detailed, needs to be briefly commented on. Hoffmann mentions that dorsal gills are lacking but they are undoubtedly present in the lectotype and all paralectotypes (dorsal gills are often hard to see in preserved animals). The anatomical traits he describes agree with our observations on the type material. The intestinal loops, Hoffmann says, are of type I but slightly different from the regular type I due to the absence of a loop. Hoffmann calls it a type Ia. His illustration of it clearly represents a type V (Hoffmann 1928: pl. 3, fig. 11). The spine of the accessory penial gland is 1 mm long and the retractor muscle attaches to the posterior end of the visceral cavity. According to Hoffmann (1928: 53), *O. platei* is most closely related to *O. tumidum* Semper, 1880 and *O. nebulosum* Semper, 1880 but differs from them based on the penis size. *Onchidium tumidum* was recently transferred to *Paromoionchis* (Dayrat et al. 2019a), and *O. nebulosum* (type locality in Palau) applies to a *Peronia* species but is regarded here as a *nomen dubium* (see general discussion).

Additional specimens were found in historical museum collections which could be identified as *P. platei* mostly based on the intestinal loops of type V, the specimen size, and their geographic origin. Specimens from Kiribati (SMNH 106488) are especially interesting because they confirm the presence of specimens similar to *P. platei* far from Hawaii and Papua New Guinea, which strongly supports the assumption that *P. platei* is widespread across the entire West Pacific. Note that those specimens from Kiribati are not identified as *P. setoensis* (which is anatomically cryptic with *P. platei*) because *P. setoensis* is found in much colder waters (33°N) in Japan (Fig. 6).

Labbé (1934a: 224) merely mentioned *Onchidium platei* as one of the valid *Onchidium* species names. Ruthensteiner (1997) briefly commented on the anatomy of the lung of Onchidium cf. branchiferum, based on specimens from Hawaii. Those were most likely specimens of *Peronia platei*, the only *Peronia* species found in Hawaii. Finally, note that the specimen [706] (UF 303653) was tentatively referred to as *Peronia* sp. 1 by Dayrat et al. (2011).

No *Peronia* slug from Hawaii was positively demonstrated to belong to *P. verruculata* (unit #1), which is characterized by intestinal loops of type I. Therefore, Hoffmann’s (1928: 44, 73) record of *O. verruculatum* from Hawaii is interpreted here as a misidentification of *P. platei*. Labbé (1934a: 193), Solem (1959: 39), and Marcus and Marcus (1970: 213) all assumed that *P. verruculata* was present in Hawaii based on Hoffmann’s (1928) study, without collecting or examining any new material.

*Onchidella evelinae* Marcus & Burch, 1965 was described based on small specimens (average length 6 mm) from Eniwetok Atoll, Marshall Islands (ca. 11°N, 162°E). The type material was deposited at the Museum of Zoology, University of Michigan, but could not be located there (personal communication from the collection manager, Dr. Taehwan Lee). *Onchidella evelinae* is a misidentification for one of the onchidiid species present in the Marshall Islands: it cannot refer to *Onchidella* slugs because an accessory penial gland is mentioned in the original description and because *Onchidella* is not present in the middle of the West Pacific. The Marshall Islands are within the distribution range of *P. platei* (Fig. 6), but a detail from the original description (the internal organs can be seen through the dorsal notum) suggests that *O. evelinae* does not refer to *Peronia* slugs because their notum is too thick for internal organs to be seen through it. *Peronia peronii* is also present in the Marshall Islands (Fig. 6), but, given the very small size of the specimens and that they were sexually mature, it is most unlikely that *O. evelinae* is a junior synonym of *P. peronii* (Fig. 6). The size of the spine of the accessory penial gland (1.3 mm) reported in the original description of *O. evelinae* is compatible with what is currently known (< 1 mm) for *P. platei* (Table 4). *Onchidella evelinae* is regarded here as a new junior subjective synonym of *Marmaronchis vaigiensis* (Quoy & Gaimard, 1825): first, because internal organs can occasionally be seen through its thin notum (e.g., Dayrat et al. 2018: fig. 5E); second, because there are known records (Dayrat et al. 2018: fig. 9) of *M. vaigiensis* in Pohnpei, Micronesia (ca. 6°N, 158°E), just a few degrees west of the Marshall Islands, and it is very possible that *M. vaigiensis* also is in the Marshall Islands. The size of the spine of the accessory penial gland (1.3 mm) reported in the original description of *O. evelinae* is higher than what is currently known for *M. vaigiensis* (< 1 mm), but that trait does vary intra-specifically.

##### Peronia setoensis

Taxon classificationAnimaliaSystellommatophoraOnchidiidae

Dayrat & Goulding sp. nov.

http://zoobank.org/AF7DC925-3FCB-4AA3-8EFA-6345D4FA0C2B

**Figure 33.**
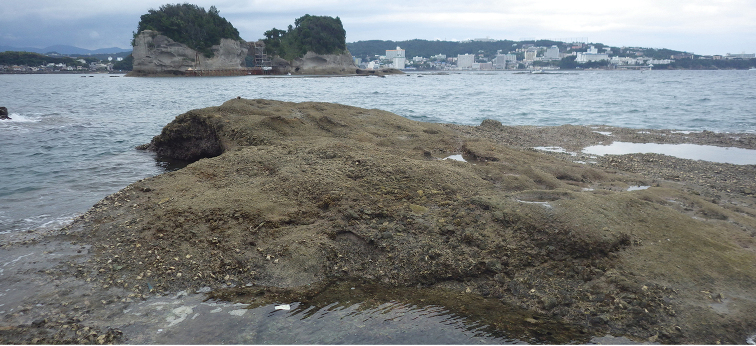
Habitat, *Peronia setoensis*, Japan, Honshu, near the Seto Marine Laboratory, type locality, exposed rock wall and platform.

**Figure 34.**
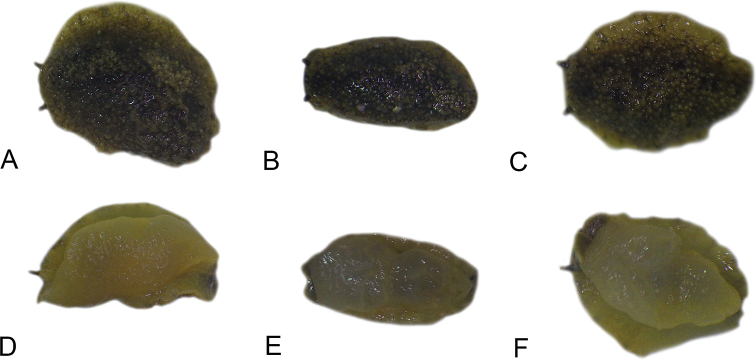
Live animals, *Peronia setoensis*, Japan, Honshu **A** holotype, dorsal view, 20 mm long [5383] (NSMT-Mo 78985) **B** dorsal view, 10 mm long [5384] (NSMT-Mo 78986) **C** dorsal view, 12 mm long [5385] (NSMT-Mo 78986) **D** ventral view, same as **A**; **E** ventral view, same as **B**; **F** ventral view, same as **C**.

**Figure 35.**
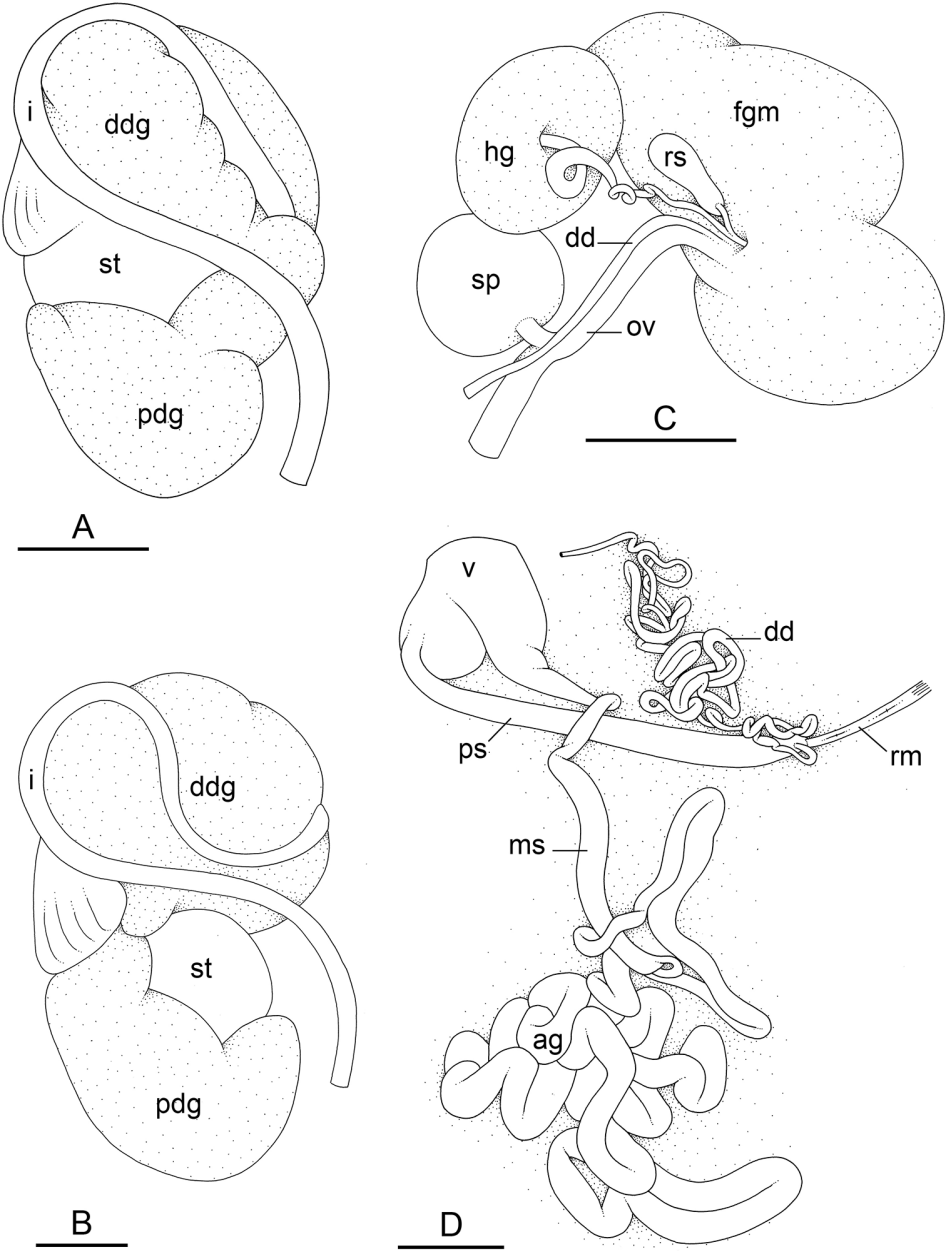
*Peronia setoensis*, Japan, Honshu **A** [3753] (NSMT-Mo 78987) **B–D** holotype [5383 H] (NSMT-Mo 78985) **A** digestive system, dorsal view, with intestinal loops of type V **B** digestive system, dorsal view, with intestinal loops of type V **C** posterior, hermaphroditic (female) reproductive system **D** anterior, male, copulatory apparatus. Scale bars: 2 mm (**A–D**). Abbreviations: ag accessory penial gland, dd deferent duct, ddg dorsal digestive gland, fgm female gland mass, hg hermaphroditic gland, i intestine, ms muscular sac, ov oviduct, pdg posterior digestive gland, ps penial sheath, rm retractor muscle, rs receptaculum seminis, sp spermatheca, st stomach, v vestibule.

**Figure 36.**
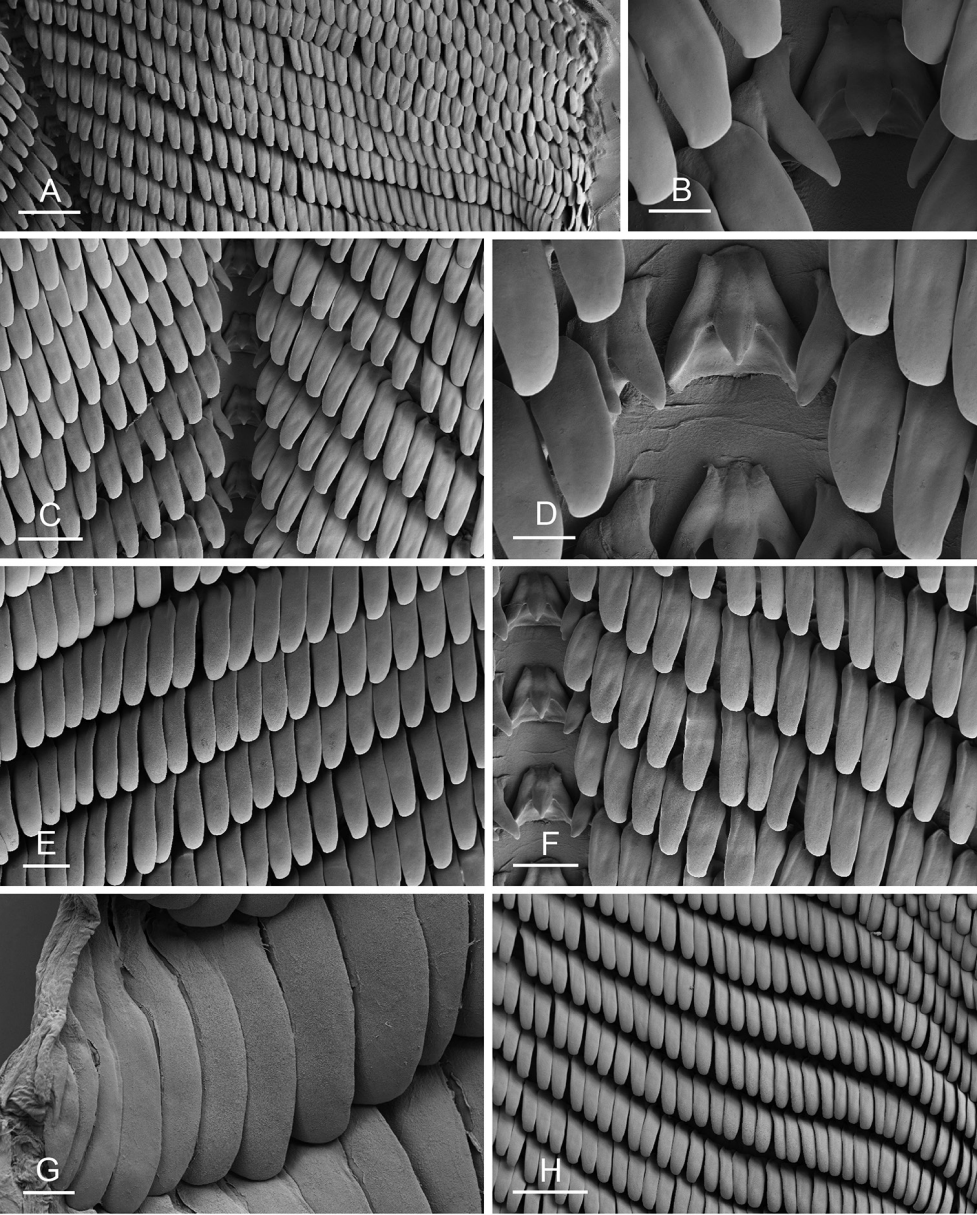
Radula, *Peronia setoensis*, Japan, Honshu **A–C** holotype [5383 H] (NSMT-Mo 78985) **D–F** [3754] (NSMT-Mo 78987) **G, H** [3753] (NSMT-Mo 78987) **A** right half rows of teeth **B** rachidian and innermost lateral teeth **C** rachidian and lateral teeth **D** rachidian and innermost lateral teeth **E** lateral teeth **F** rachidian and lateral teeth **G** outermost lateral teeth **H** lateral teeth. Scale bars: 100 μm (**A, H**), 15 μm (**B**), 60 μm (**C**), 20 μm (**D**), 40 μm (**E, F**), 10 μm (**G**).

**Figure 37.**
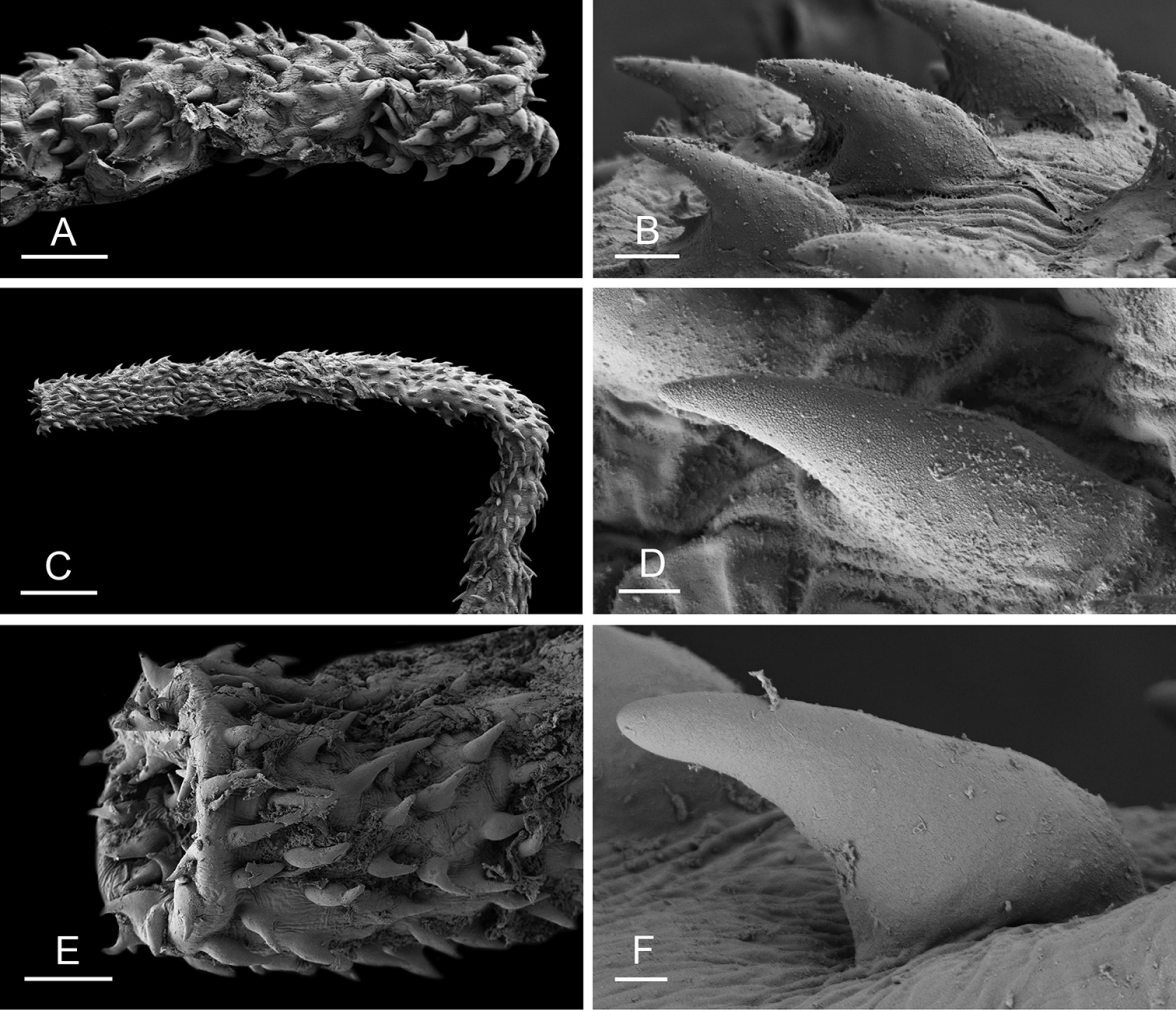
Penis and penial hooks, *Peronia setoensis*, Japan, Honshu **A, B** holotype [5383 H] (NSMT-Mo 78985) **C, D** [3753] (NSMT-Mo 78987) **E, F** [3754] (NSMT-Mo 78987). Scale bars: 40 μm (**A**), 4 μm (**B**), 100 μm (**C**), 2 μm (**D, F**), 20 μm (**E**).

**Figure 38.**
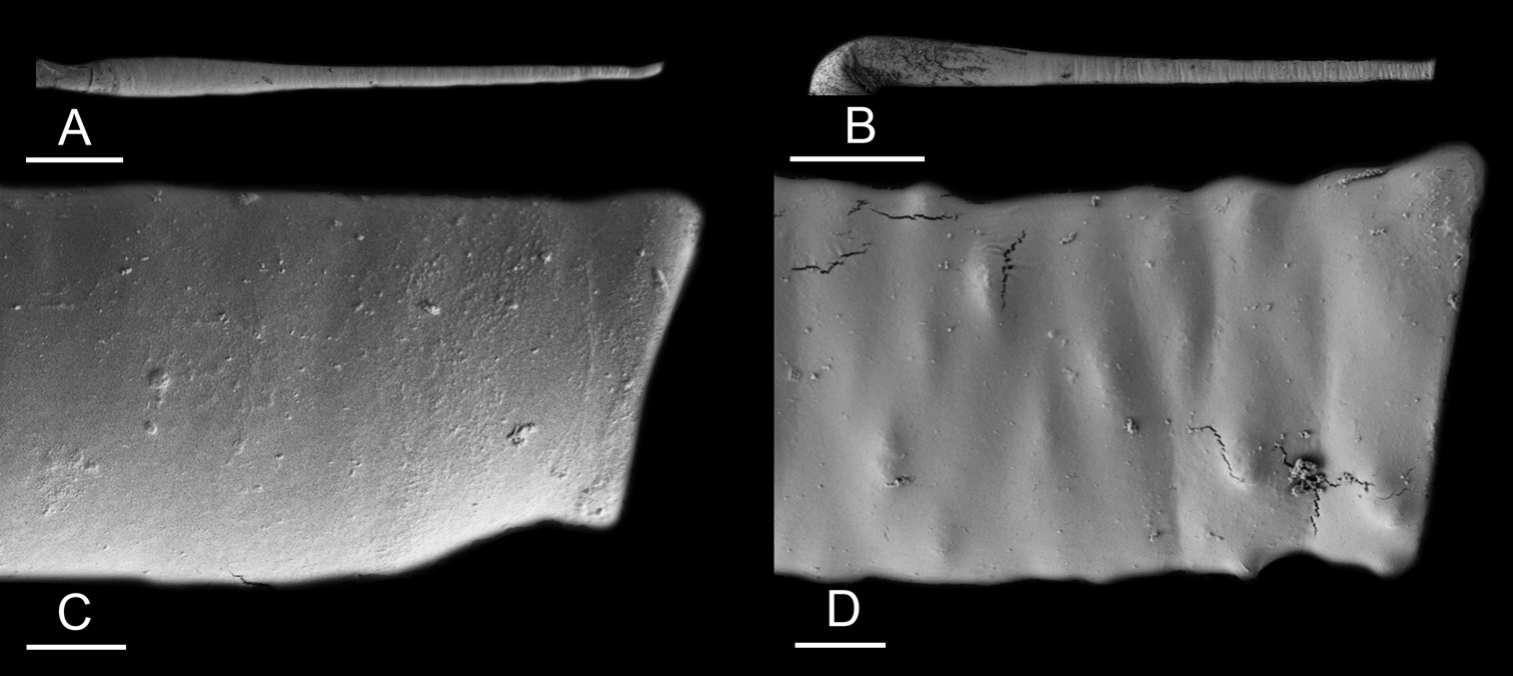
Accessory penial gland spine, *Peronia setoensis*, Japan, Honshu **A, C** [3753] (NSMT-Mo 78987) **B, D** [3754] (NSMT-Mo 78987). Scale bars: 200 μm (**A, B**), 6 μm (**C, D**).

Figs 33 , 34 , 35 , 36 , 37 , 38

###### Type material.

***Holotype*.** Japan • holotype, hereby designated, 20/15 mm [5383]; Honshu, Wakayama, Nishimuro, near Seto Marine Biological Laboratory; 33°41.504'N, 135°20.179'E; 30 Aug 2014; R. Cumming leg.; exposed rock wall and platform; NSMT-Mo 78985.

###### Additional material examined.

Japan • 3 specimens 13/8 mm [5382], 10/5 mm [5384], and 12/10 mm [5385]; same collection data as for the holotype; NSMT-Mo 78986. • 2 specimens 15/10 mm [3753] and 15/10 mm [3754]; Honshu, Wakayama, Nishimuro, near Seto Marine Biological Laboratory; 33°41.533'N, 135°20.265'E; 2014; T Nakano leg.; NSMT-Mo 78987.

###### Additional material examined

**(historical museum collections).** Japan • 1 specimen 23/20 mm; Sagami Bay, Misaki; 1930–1931; Gislén’s Pacific Expedition 1930–1931 leg.; littoral rocky bottom; SMNH 180725.

###### Distribution

(Fig. 6). Endemic to subtropical waters of Japan: Honshu, Nishimuro, near Seto Marine Biological Laboratory (33N, type locality), Sagami Bay (35°N), and possibly Boso Peninsula, near Sagami Bay (35°N); Kyushu, Nagasaki, 32N (Keferstein 1865a, b, as *P. verruculata*).

###### Etymology.

*Peronia setoensis* is named after its type locality, near the Seto Marine Biological Laboratory: *setoensis* is a latinized adjective that agrees in gender (feminine) with the generic name (ICZN 1999: Article 31.2).

###### Habitat

(Fig. 33). *Peronia setoensis* is found in the rocky intertidal. Few individuals are currently known but it may be discovered in additional localities in the future.

###### Color and morphology of live animals

(Fig. 34). The dorsal notum is greenish brown, light to dark, mottled with darker and lighter areas, occasionally with yellowish sides. The color of the dorsal papillae varies as that of the background itself. The ventral surface (foot and hyponotum) is yellowish or greyish and can change rapidly in any given individual. The ocular tentacles are brown-grey, like the head. The dorsal notum of live animals is covered by dozens of papillae of various sizes. Some papillae bear black dorsal eyes at their tip. The number of papillae with dorsal eyes is variable (from 8 to 12). The largest specimens are 20 mm long.

###### Digestive system

(Figs 35A, B, 36). Examples of radular formulae are presented in Table 5. The median cusp of the rachidian teeth is approximately 35 μm long. The hook of the lateral teeth is approximately 90 μm long. The intestinal loops are of type V.

###### Reproductive system

(Figs 35C, D, 37, 38). In the anterior (male) parts, the muscular sac of the accessory penial gland is less than 5 mm long. The hollow spine of the accessory penial gland is narrow, elongated, and straight or slightly curved, and its shape (including at its tip) varies between individuals. Its length ranges from 0.9 mm ([3754] NSMT-Mo 78987) to 1.2 mm ([3753] NSMT-Mo 78987). Its diameter at the conical base ranges from 80 to 85 μm. Its diameter at the tip ranges from 15 to 25 μm. The retractor muscle is shorter or longer than the penial sheath and inserts near the heart. Inside the penial sheath, the penis is a narrow, elongated, soft, hollow tube. Its distal end bears conical hooks which are less than 25 μm long.

###### Diagnostic features

(Table 4). *Peronia setoensis* is cryptic with *P. platei*. Both species share the same combination of anatomical traits: intestinal loops of type V, retractor muscle inserting at the posterior end of the visceral cavity, a spine of the accessory penial gland from 0.9 to 1.2 mm long (*P. setoensis*) and from 0.7 to 1 mm long (*P. platei*). *Peronia setoensis* and *P. platei* are anatomically very similar to *P. griffithsi*, in which, however, the spine of the accessory penial gland is slightly shorter (less than 0.62 mm long). All three species are distributed in the West Pacific but *Peronia setoensis* is adapted to much colder waters than *P. platei* and *P. griffithsi* (Fig. 6).

###### Remarks.

A new species name is needed because no existing name applies to the species described here. A specimen from Sagami Bay (35°N), preserved in Stockholm (SMNH 180725), not included by Hoffmann (1928: 73) in his list of material for *O. verruculatum*, is identified here as *P. setoensis* because of its intestinal loops of type V (Table 4). This specimen indicates that *P. setoensis* is distributed on the eastern Pacific coast of Japan north of the type locality.

Keferstein (1865b) described as *P. verruculata* three slugs from Nagasaki, Kyushu, Japan (ca. 32°44'N). His written description (Keferstein 1865b) was also based on an individual from Java but his figure captions clearly indicate that his drawings illustrated an individual from Nagasaki (Keferstein 1865b: pl. VI, figs 14–16): Keferstein’s (1865b: pl. VI, fig. 16) drawing of the internal anatomy unmistakably illustrates intestinal loops of type V. Therefore, it is very likely that *P. setoensis*, the only one species of *Peronia* slugs with intestinal loops of type V in Japan, is also distributed in Kyushu. It is unclear whether Keferstein’s (1865a: pl. CII, figs 20*, 20**, pl. CV, figs 1, 2) drawings illustrate the same Nagasaki individual as the one with intestinal loops of type V (Keferstein 1865b: pl. VI, fig. 16). It cannot be excluded that Keferstein examined several species found in Japan (Fig. 6). The Java individual cannot be identified.

The molecular data presented here indicate that there are four *Peronia* species in Japanese waters, but their geographic ranges need to be explored in better detail (Fig. 6). *Peronia setoensis* is definitely (our DNA sequences) present in southern Honshu (Wakayama Prefecture) and very likely in Kyushu based on Keferstein’s (1865b: pl. VI, fig. 16) drawing of intestinal loops of type V. *Peronia verruculata* (unit #1) is definitely (our DNA sequences) present in Wakayama Prefecture (ca. 33°N), southern Honshu, and is thus expected to be present in all Japanese waters south of Wakayama Prefecture. Also, *Peronia verruculata* is present in Sakurajima, Kyushu (ca. 31°N) and Okinawa (ca. 26°N) based on sequences that Takagi et al. (2019) recently published (see remarks on *P. verruculata*). *Peronia peronii* is also present in Okinawa based on COI sequences that Takagi et al. (2019) recently published (see remarks on *P. peronii*). And, finally, our new species *P. okinawensis* is only known from Okinawa so far.

Besides Keferstein (1865a, b), several authors mentioned onchidiids from Japan but, in most cases, species cannot be identified based on the limited information provided. Stimpson (1855: 380) described *Onchis fruticosa* based on slugs with dorsal gills from Kikaijima (28°30'N), between Kyushu and Okinawa, which could potentially belong to any of the four species present in Japanese waters. As a result, *Onchis fruticosa* is regarded as a *nomen dubium* (see general discussion).

Baba (1958) illustrated onchidiid slugs from three different places: Tokara Islands, just south of Kyushu (ca. 30°N); Amakusa, near Nagasaki, Kyushu (ca. 32°30'N); and Misaki, Osaka, Honshu (ca. 34°N). Baba (1958: 144) indicates that some specimens of *Onchidium verruculatum* from Tokara Islands were very large (up to 120 mm long), suggesting that *P. peronii* is found there, which would be its northernmost record (see remarks on *P. peronii*). The smaller specimens that Baba (1958: 144) mentions from Tokara Islands could be a combination of *P. verruculata* (unit #1) and possibly *P. setoensis*. The two species which Baba (1958: 21) seems to distinguish (as *Onchidium* and *Onchidium verruculatum*) in Misaki, near Osaka, could be *P. verruculata* (unit #1) and *P. setoensis*, which, based on our DNA sequences, are sympatric near the Seto Marine Laboratory, which is close to Osaka. And, finally, the slugs crawling on mud in Amakusa, near Nagasaki, are not *Peronia* slugs (Baba 1958: 51) but most likely belong to *Paromoionchis tumidus*, a species which is present nearby, in Kumamoto Uki, as the COI sequences from the slugs of “Group I” in Takagi et al. (2019) cluster with our sequences of *P. tumidus* (Dayrat et al. 2019a).

Katagiri and Katagiri (2007) distinguished two *Peronia* species (both as *Onchidium verruculatum*) in the waters of the Boso Peninsula (near Sagami Bay, Honshu, ca. 35°N) based on external appearance and development. One species, called Isowamochi, is characterized by planktotrophic development, and the other, called Minneawamochi, by direct development. Most likely, these slugs belong to *P. verruculata* (unit #1) and *P. setoensis*, which are the only two *Peronia* species found north of 30N. However, this assumption would have to be confirmed with fresh collections and DNA sequences. Ueshima (2007) commented that the external distinction between the two species recognized by Katagiri and Katagiri (2007) is far more subtle and problematic, and he rightly suggested that molecular data could determine the relationships between those two species and *P. verruculata* (erroneously said to be from the Mediterranean). Note that Ueshima’s (2007) material, which covered a broad latitudinal range from the Kanagawa Prefecture (near Sagami Bay, ca. 35°N) all the way to Ishigaki Island (Okinawa, ca. 24°N), potentially included slugs from all four *Peronia* species found in Japan.

##### Peronia griffithsi

Taxon classificationAnimaliaSystellommatophoraOnchidiidae

Dayrat & Goulding sp. nov.

http://zoobank.org/61BB1B61-C9FC-43A8-AB92-5C4AEB7411E0

Figs 39 , 40 , 41 , 42 , 43 , 44 , 45 , 46 , 47 , 48

###### Type material.

***Holotype*.** Mauritius • holotype, hereby designated, 15/10 mm [3157 H]; Mahebourg, waterfront; 20°24.317'S, 57°42.605'E; 13 Jun 2014; TC Goulding leg.; st 178, rocky intertidal, with algae, just before sunrise; MNHN-IM-2000-35265.

###### Additional material examined.

Mauritius • 7 specimens 8/5 mm [3606], 20/15 mm [3153], 22/17 mm [3154], 17/15 mm [3155], 20/14 mm [3156], 7/4 mm [3607], and 6/4 mm [3608]; same collection data as for the holotype; MNHN-IM-2019-1608.

Indonesia • 1 specimen 17/12 mm [2936]; Kei Islands, Fiditan; 05°35.957'S, 132°45.112'E; 28 Feb 2014; M Khalil and field party leg.; st 144, rocks behind muddy *Rhizophora* mangrove; UMIZ 00176. • 2 specimens 18/10 mm [2934] and 25/16 mm [3566]; same collection data as for the preceding; UMIZ 00177.

Papua New Guinea • 1 specimen 6/3 mm [6095]; New Ireland, east coast, Povalval; 02°41'S, 150°57'E; 11 & 13 Jun 2014; MNHN Expedition Kavieng 2014 leg.; st KM05, mixed hard platform and seagrass bed at outlet of rivulet; MNHN-IM-2013-53535.

###### Additional material examined

**(historical museum collections).** Indonesia • 161 specimens from 2/2 to 23/14 mm; Kei Islands, Toeal; 18 Mar 1922; T Mortensen leg.; NHMD 635303.

###### Distribution

(Fig. 6). Indo-West Pacific: Mauritius (type locality), Indonesia (Kei Islands), and Papua New Guinea (New Ireland).

###### Etymology.

*Peronia griffithsi* is named after Owen Griffiths, who kindly and generously hosted and guided one of us (Tricia Goulding) in Mauritius.

###### Habitat

**Figure 39.**
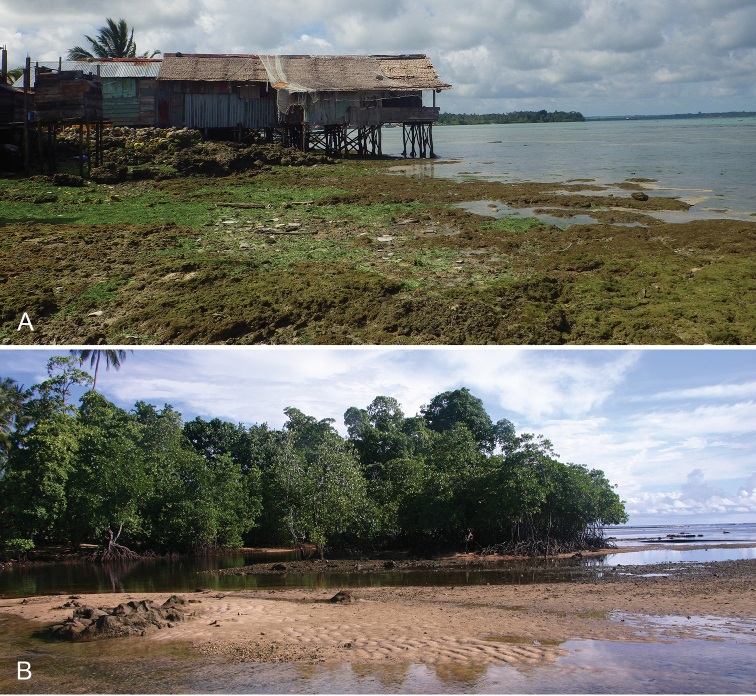
Habitats, *Peronia griffithsi***A** Indonesia, Kei Islands, rocks behind muddy *Rhizophora* mangrove (st 144) **B** Papua New Guinea, New Ireland, mixed hard platform and seagrass bed at outlet of rivulet (st KM 05).

(Fig. 39). *Peronia griffithsi* is found in the rocky intertidal, like most other *Peronia* slugs. Our specimens from Mauritius were collected just before sunrise, suggesting that *P. griffithsi* is, at least partly, a nocturnal species.

###### Color and morphology of live animals

**Figure 40.**
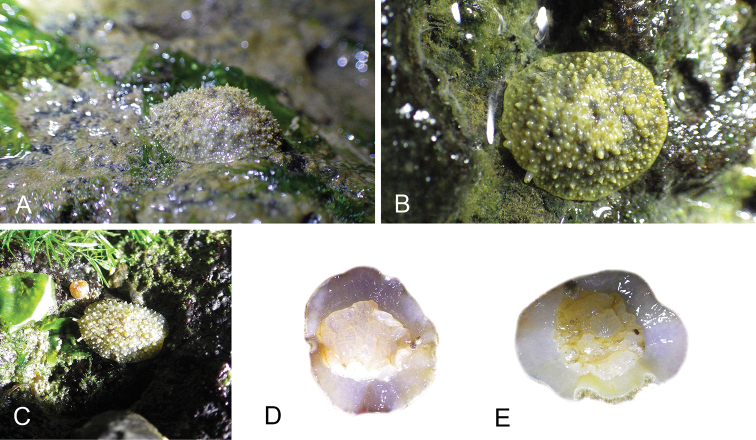
Live animals, *Peronia griffithsi*, Mauritius **A** dorsal view, 20 mm long [3153] (MNHN-IM-2019-1608) **B** dorsal view, 17 mm long [3155] (MNHN-IM-2019-1608) **C** dorsal view, 8 mm long [3606] (MNHN-IM-2019-1608) **D** holotype, ventral view, 15 mm long [3157 H] (MNHN-IM-2000-35265) **E** ventral view, 22 mm long [3154] (MNHN-IM-2019-1608).

**Figure 41.**
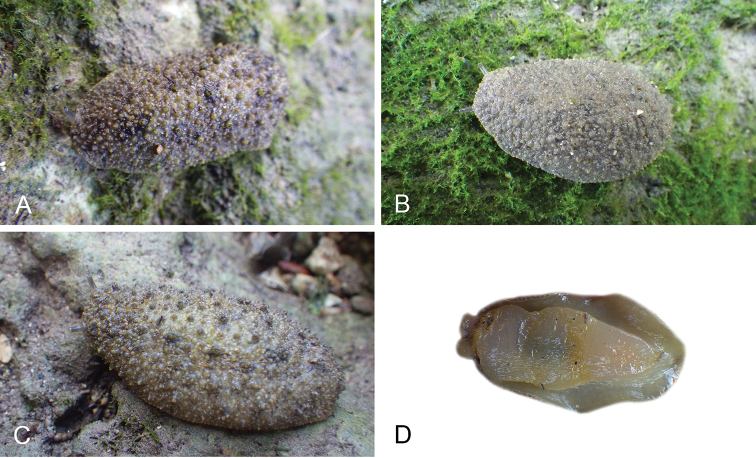
Live animals, *Peronia griffithsi*, Indonesia, Kei Islands **A** dorsal view, 18 mm long [2934] (UMIZ 00177) **B** dorsal view, 17 mm long [2936] (UMIZ 00176) **C** dorsal view, 25 mm long [3566] (UMIZ 00177) **D** ventral view, same as **A**.

(Figs 40, 41). No picture of live animals was available for specimens from Kavieng. The description of the color of live animals is based on Mauritius and Kei individuals. The dorsal notum is greenish brown, light to dark, mottled with darker and lighter areas. The color of the dorsal papillae varies as that of the background itself, but dorsal papillae can also be yellowish-greenish. The ventral surface (foot and hyponotum) varies from whitish to yellowish and can change rapidly in any given individual. The ocular tentacles are brown-grey, like the head. The dorsal notum of live animals is covered by dozens of papillae of various sizes. Some papillae bear black dorsal eyes at their tip. The number of papillae with dorsal eyes is variable (from 6 to 10). The largest specimens are 25 mm long.

###### Digestive system

**Figure 42.**
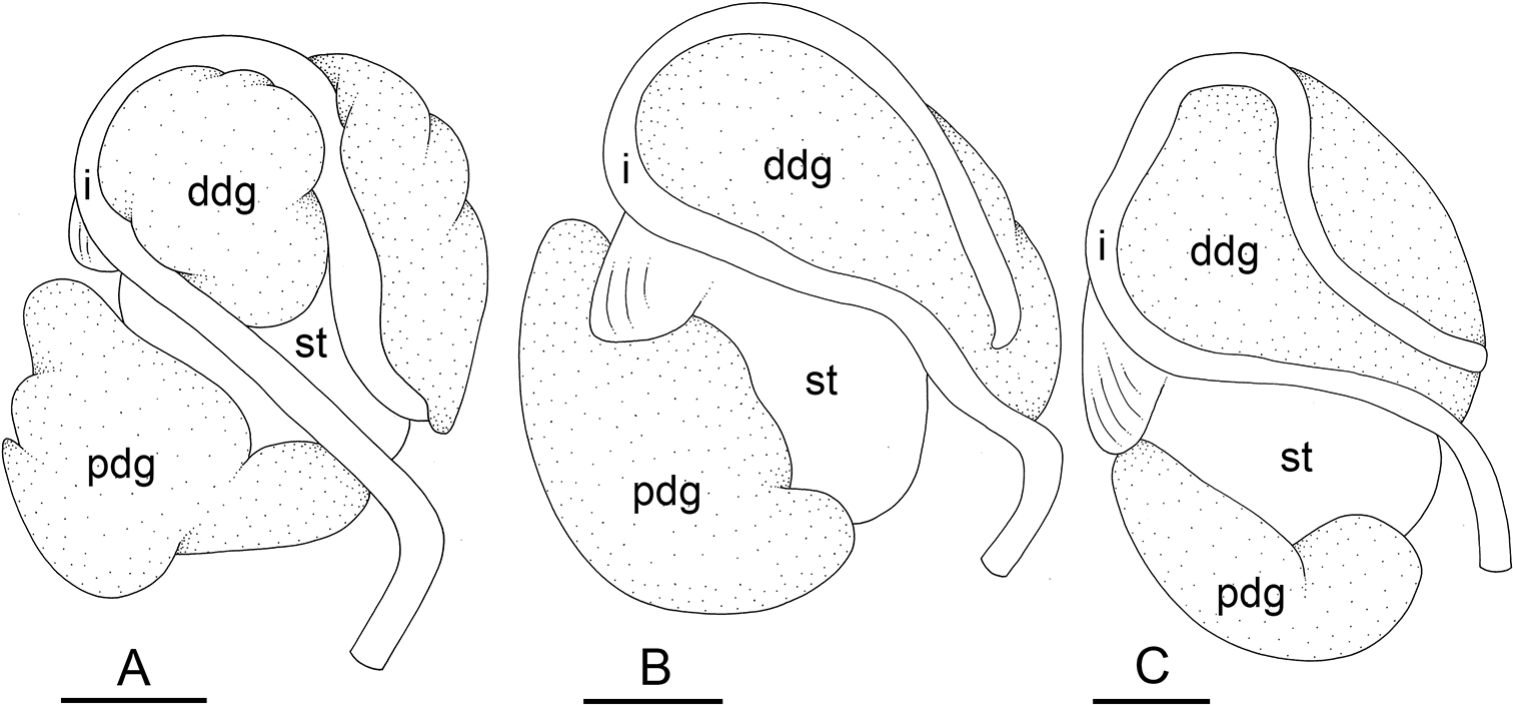
Digestive system, dorsal view, *Peronia griffithsi*, with intestinal loops of type V **A** holotype, Mauritius [3157 H] (MNHN-IM-2000-35265) **B** Mauritius [3153] (MNHN-IM-2019-1608) **C** Indonesia, Kei Islands [2936] (UMIZ 00176). Scale bars: 2 mm (**A–C**). Abbreviations: ddg dorsal digestive gland, i intestine, pdg posterior digestive gland, st stomach.

**Figure 43.**
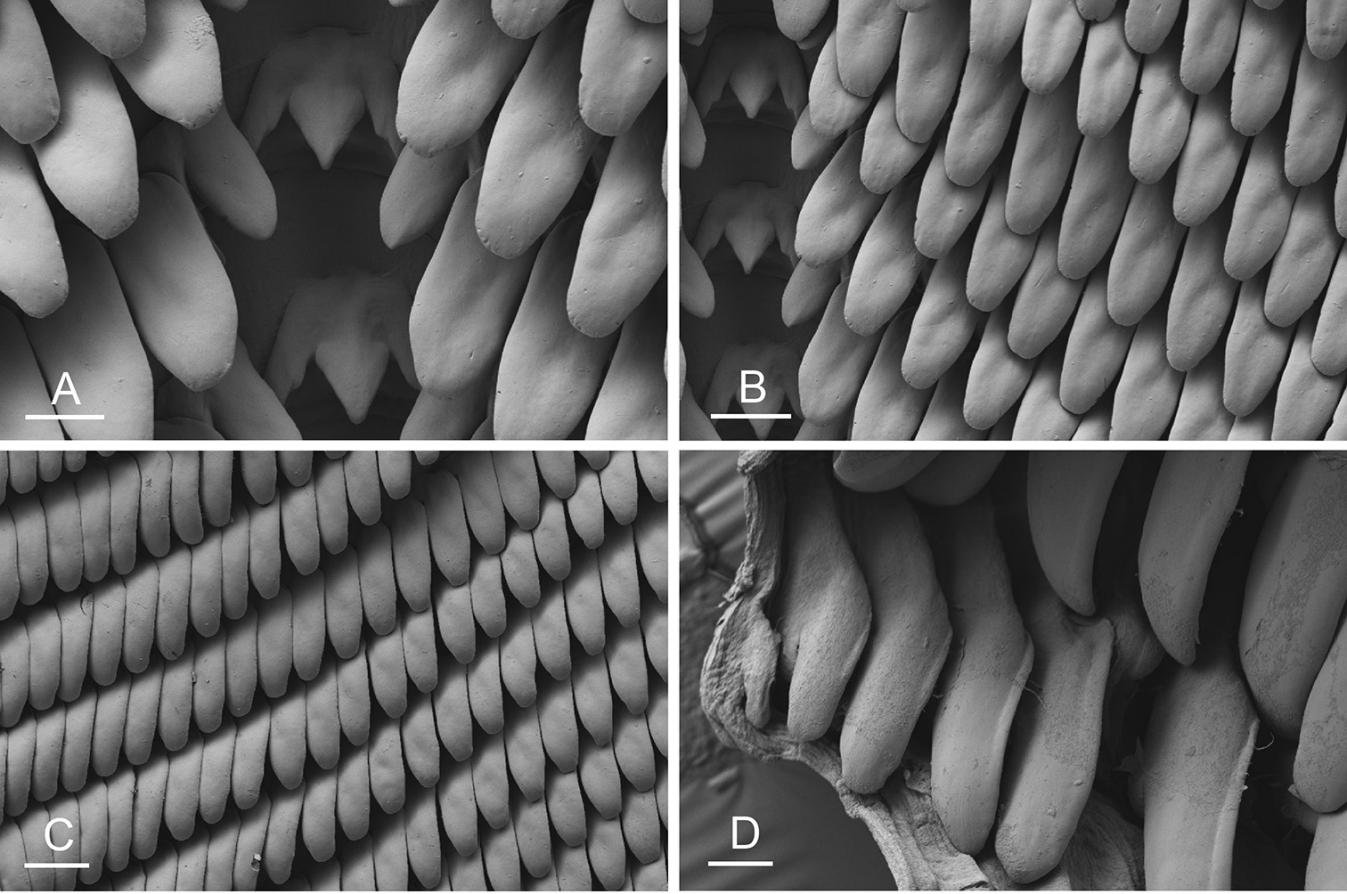
Radula, *Peronia griffithsi*, Indonesia, Kei Islands, [3566] (UMIZ 00177) **A** rachidian and innermost lateral teeth **B** rachidian and lateral teeth **C** lateral teeth **D** outermost lateral teeth. Scale bars: 20 μm (**A**), 30 μm (**B**), 40 μm (**C**), 10 μm (**D**).

**Figure 44.**
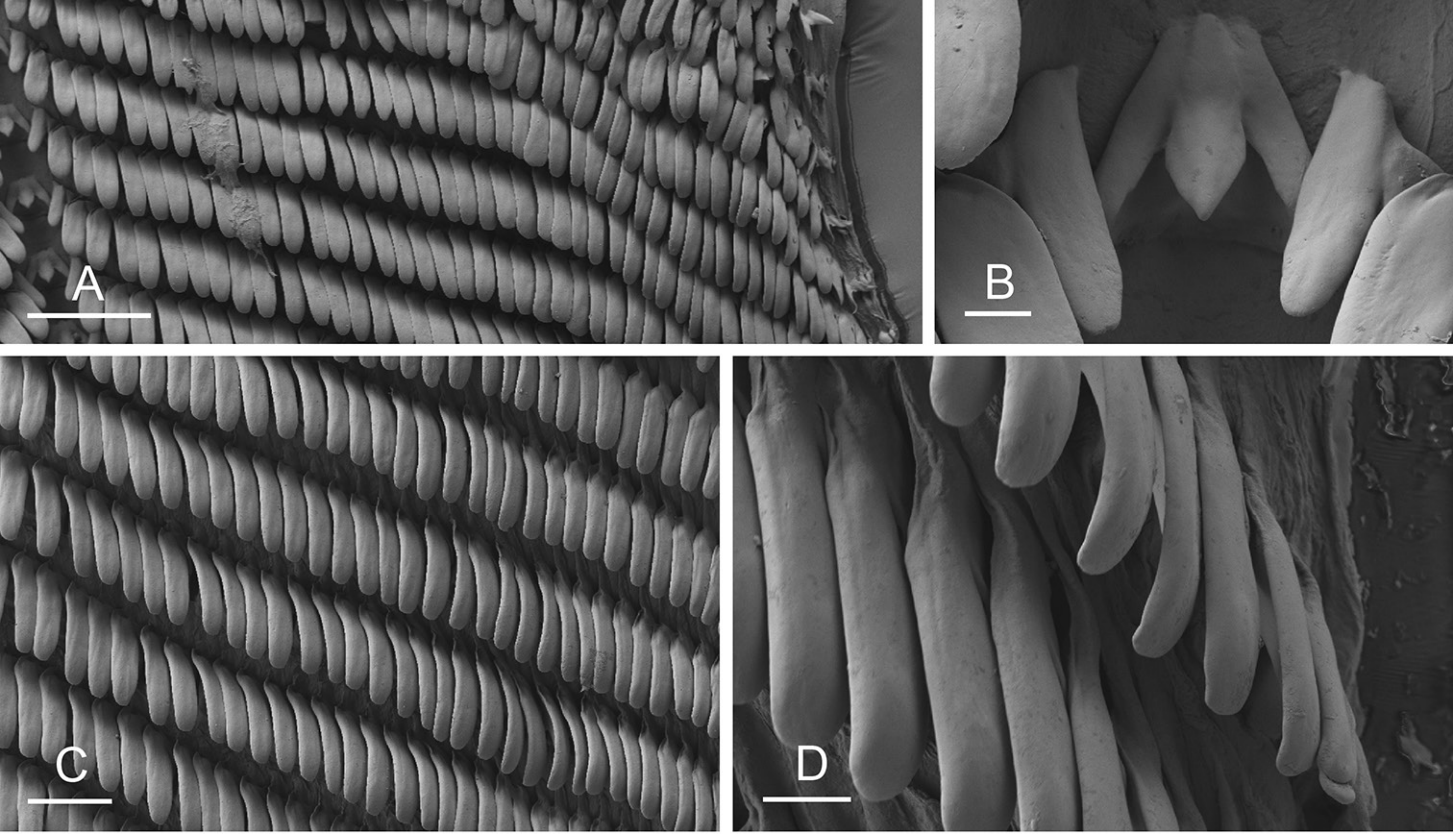
Radula, *Peronia griffithsi*, Mauritius **A** holotype [3157 H] (MNHN-IM-2000-35265) **B–D** [3153] (MNHN-IM-2019-1608) **A** right half rows of teeth **B** rachidian and lateral teeth **C** lateral teeth **D** outermost lateral teeth. Scale bars: 100 μm (**A**), 10 μm (**B, D**), 60 μm (**D**).

(Figs 42–44). Examples of radular formulae are presented in Table 5. The median cusp of the rachidian teeth is approximately 35 μm long. The hook of the lateral teeth is approximately 70 μm long. The intestinal loops are of type V.

###### Reproductive system

**Figure 45.**
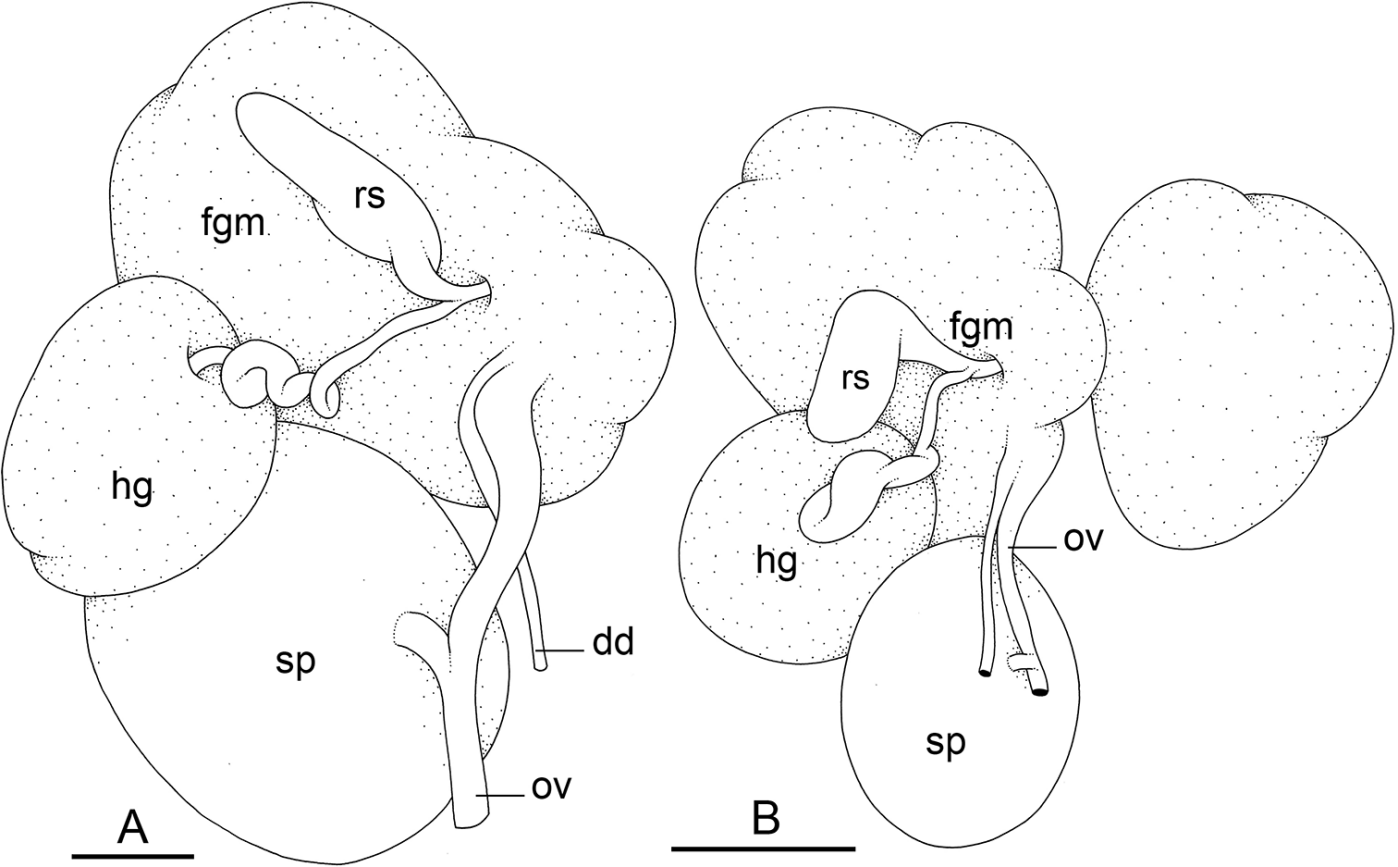
Posterior, hermaphroditic (female) reproductive system, *Peronia griffithsi***A** holotype, Mauritius [3157 H] (MNHN-IM-2000-35265) **B** Indonesia, Kei Islands [2936] (UMIZ 00176). Scale bars: 1 mm (**A**), 2 mm (**B**). Abbreviations: dd deferent duct, fgm female gland mass, hg hermaphroditic gland, ov oviduct, rs receptaculum seminis, sp spermatheca.

**Figure 46.**
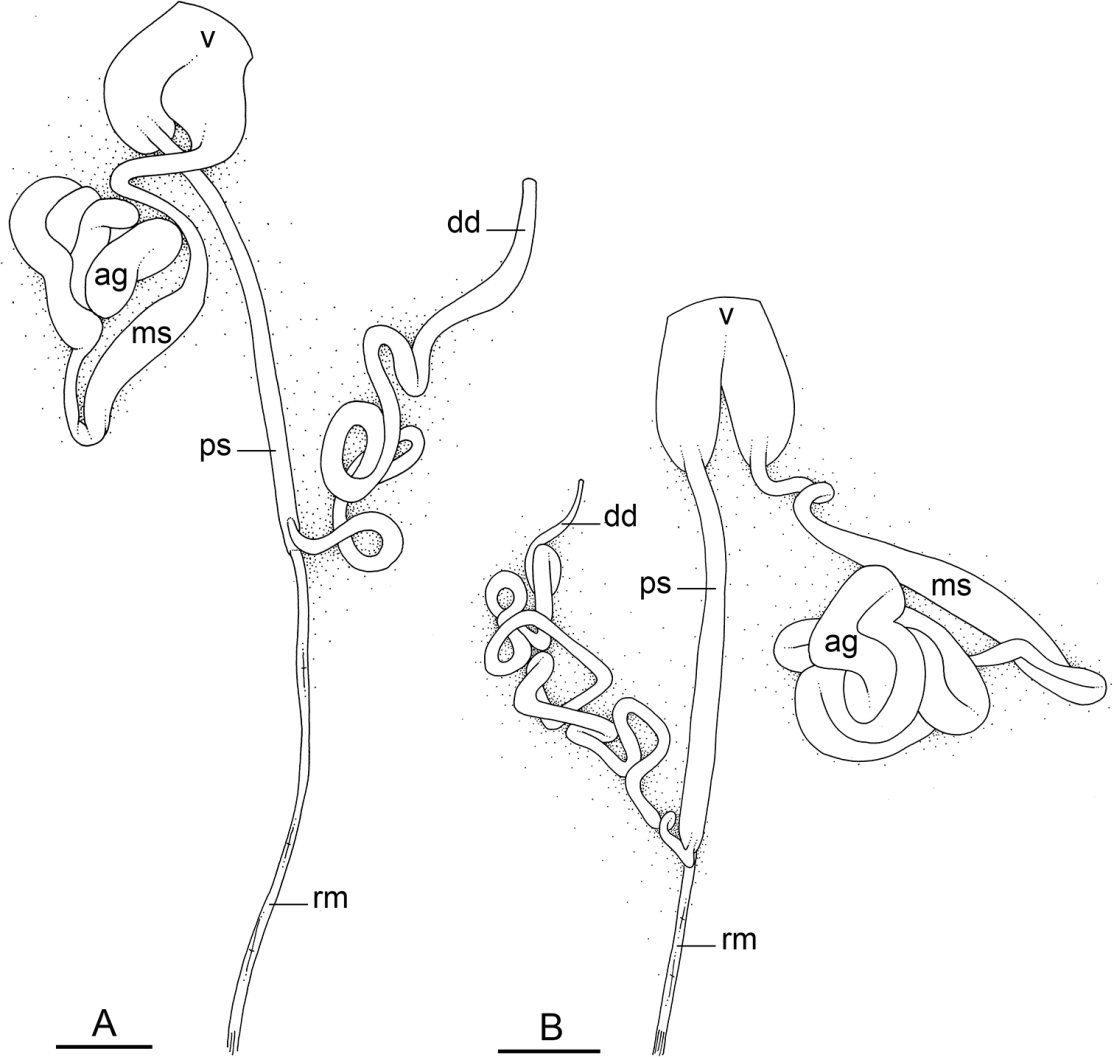
Anterior, male, copulatory apparatus, *Peronia griffithsi***A** holotype, Mauritius [3157 H] (MNHN-IM-2000-35265) **B** Indonesia, Kei Islands [2936] (UMIZ 00176). Scale bars: 1 mm (**A, B**). Abbreviations: ag accessory penial gland, dd deferent duct, ms muscular sac, ps penial sheath, rm retractor muscle, v vestibule.

**Figure 47.**
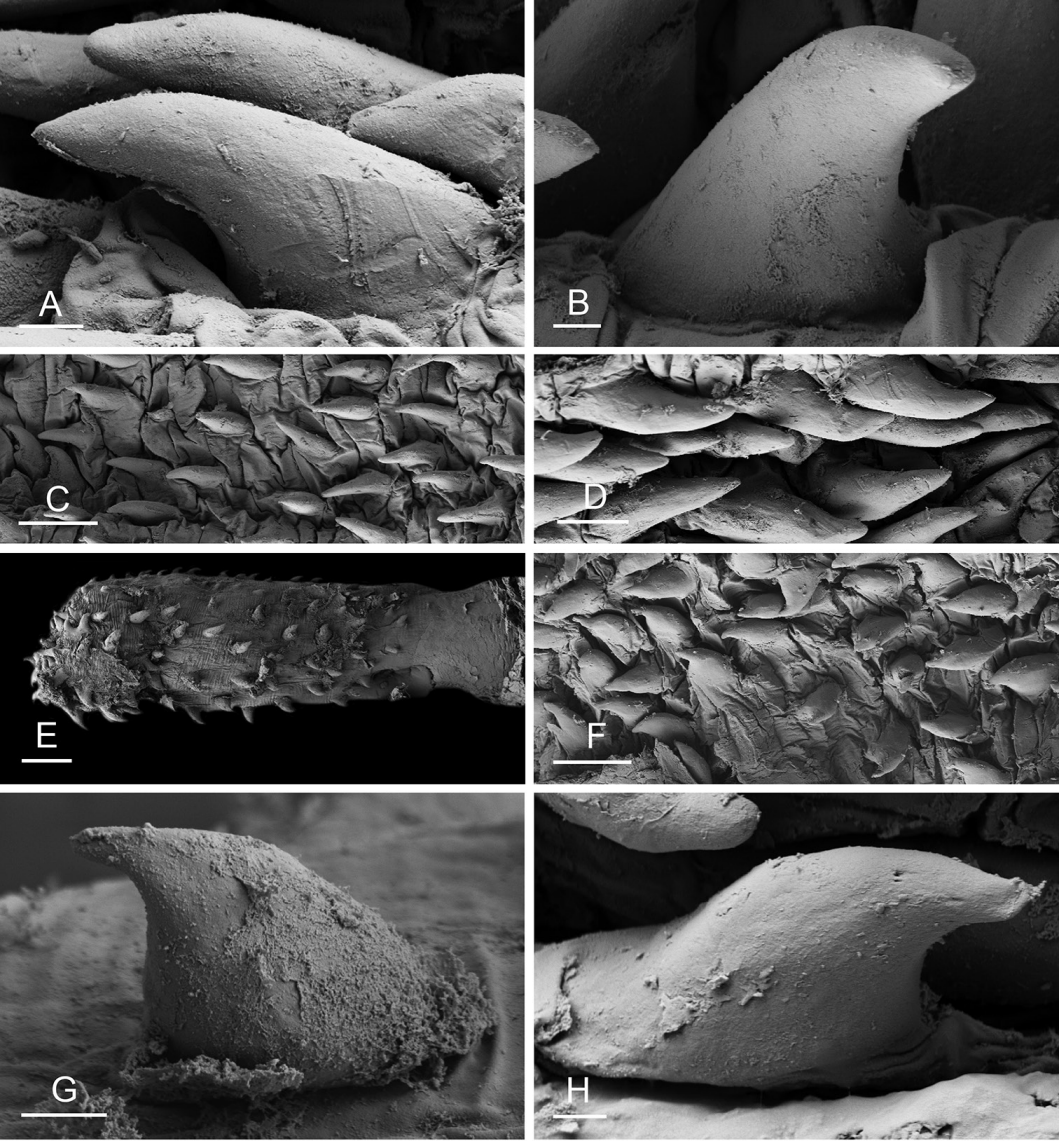
Penis and penial hooks, *Peronia griffithsi***A–D** Indonesia, Kei Islands [2936] (UMIZ 00176) **E, G** holotype, Mauritius [3157 H] (MNHN-IM-2000-35265) **F** Mauritius [3156] (MNHN-IM-2019-1608) **H** [3153] (MNHN-IM-2019-1608). Scale bars: 3 μm (**A**), 2 μm (**B, H**), 20 μm (**C, F**), 10 μm (**D**), 40 μm (**E**), 4 μm (**G**).

**Figure 48.**
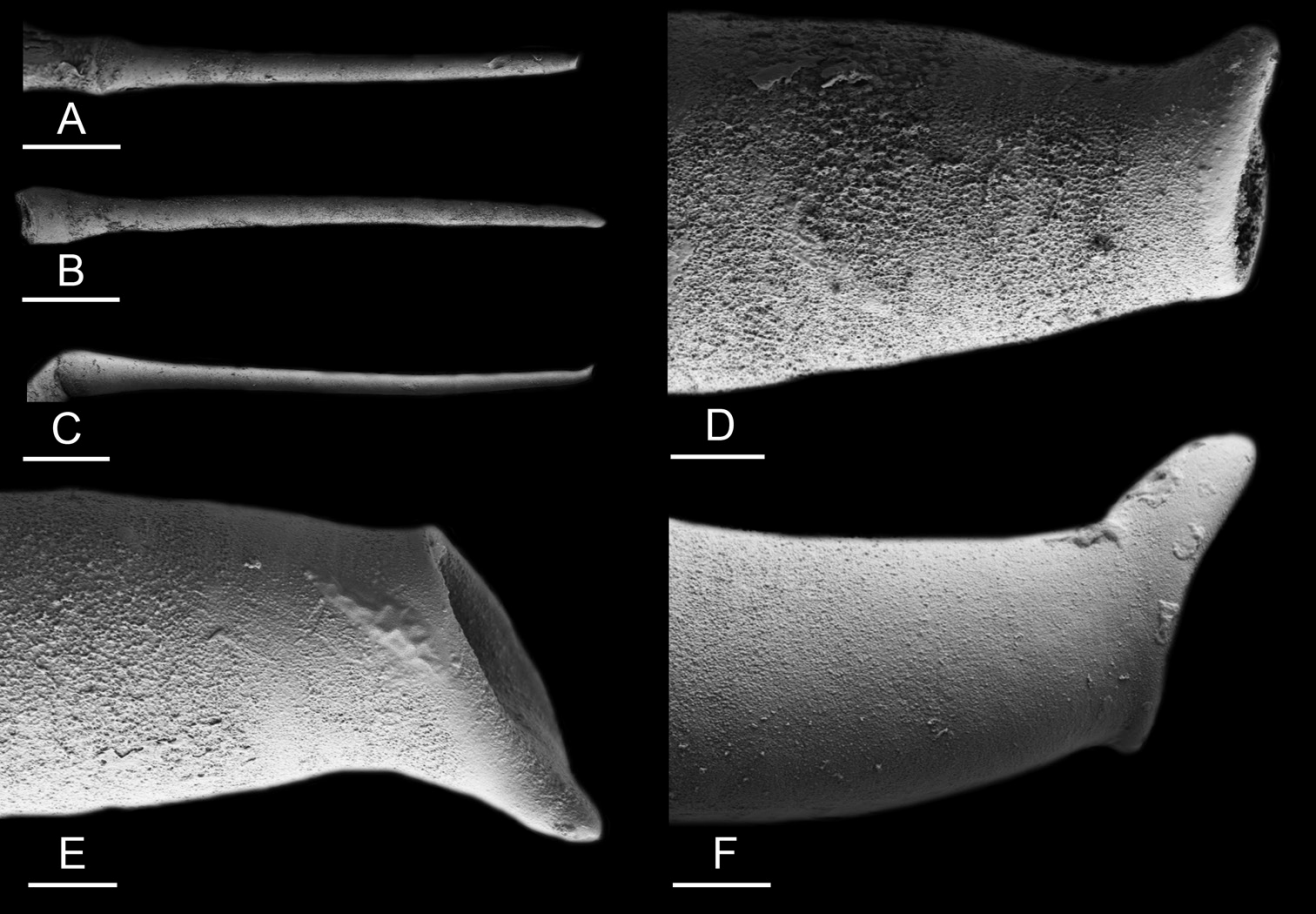
Accessory penial gland spine, *Peronia griffithsi***A** holotype, Mauritius [3157 H] (MNHN-IM-2000-35265) **B** Mauritius [3153] (MNHN-IM-2019-1608) **C** Indonesia, Kei [2934] (UMIZ 00177) **D** same as **A**; **E** Mauritius [3156] (MNHN-IM-2019-1608) **F** same as **C**. Scale bars: 100 μm (**A–C**), 5 μm (**D–F**).

(Figs 45–48). In the anterior (male) parts, the muscular sac of the accessory penial gland is less than 5 mm long. The hollow spine of the accessory penial gland is narrow, elongated, and straight or slightly curved, and its shape (including at its tip) varies between individuals. Its length is 0.62 mm ([2934] UMIZ 00177) in unit Kei and ranges from 0.5 mm ([3157 H] MNHN-IM-2000-35265) to 0.61 mm ([3153] MNHN-IM-2019-1608) in Mauritius. Its diameter at the conical base ranges from 60 to 65 μm. Its diameter at the tip ranges from 15 to 20 μm. The retractor muscle is shorter or longer than the penial sheath and inserts near the heart. Inside the penial sheath, the penis is a narrow, elongated, soft, hollow tube. Its distal end bears conical hooks which are less than 25 μm long.

###### Diagnostic features

(Table 4). *Peronia griffithsi* is characterized by a unique combination of anatomical traits: intestinal loops of type V, muscular sac of the accessory penial gland less than 5 mm long, spine of the accessory penial gland less than 0.62 mm long. In *P. platei* and *P. setoensis*, which are anatomically similar to *P. griffithsi*, the spine of the accessory penial gland is longer than 0.7 mm (*P. platei*) and 0.9 mm (*P. setoensis*).

###### Remarks.

A new species name is needed because no existing name applies to the species described here. A large population (161 specimens) from Kei Islands and identified by Hoffmann as *Onchidium verruculatum* was found in the collections of the Copenhagen Museum (NHMD 635303). Those specimens most likely belong to *P. griffithsi* because their intestinal loops are of type V (only a few individuals were dissected). Also, the retractor muscle of the few individuals dissected inserts near the end of the visceral cavity, as in specimens from Mauritius, suggesting that an insertion near the heart is not as common. Interestingly, Hoffmann (1928: 44) did not include those specimens in his list of material for *O. verruculatum*, possibly because he realized that they were different from *O. verruculatum*, with intestinal loops of type I.

##### Peronia sydneyensis

Taxon classificationAnimaliaSystellommatophoraOnchidiidae

Dayrat & Goulding sp. nov.

http://zoobank.org/7B0A9ED7-421A-4FF0-A7CE-20473AF249C1

Figs 49 , 50 , 51 , 52 , 53 , 54 , 55 , 56 , 57 , 58

###### Type material.

***Holotype*.** Australia • holotype, hereby designated, 30/20 mm [1516 H]; New South Wales, Sydney, Pittwater, Church Point; 33°39.107'S, 151°17.363'E; 24 Nov 2011; B Dayrat, R Golding & WF Ponder leg.; st 39, sand, next to a small patch of mangrove, and rocks on sandy beach; AM C.468916.001.

###### Additional material examined.

Australia – **New South Wales** • 1 specimen 23/15 mm [1517]; same collection data as for the holotype; AM C.468915.001. • 1 specimen 16/12 mm [1513]; same collection data as for the holotype; AM C.468912.004. – **Queensland** • 1 specimen 20/17 mm [1539]; Shoalwater Bay, off Canoe Passage between Townshend & Marquis Islands; 22°18.235'S, 150°27.543'E; 9 & 10 Sep 2002; I Loch, DL Beechey & AC Miller leg.; st M2002/52, rocky shoal with coarse muddy sand; AM C.459510. • 2 specimens 30/20 [1540] mm and 20/20 mm [734]; Port Clinton, beach SW of Mt Flinders; 22°32.76'S, 150°45.54'E; 1 Sep 2002; I Loch, DL Beechey & AC Miller leg.; under and on rocks, sheltered muddy sand shore; AM C.459511. • 1 specimen 50/30 mm [2680]; Mackay, Campwin Beach; 21°22.455'S, 149°18.753'E; 5 Jul 2013; TC Goulding and field party leg.; st 121, by boat ramp, mangrove margin with large rocks by creek, *Rhizophora* and soft mud; MTQ. • 1 specimen 10/6 mm [2653]; Bowen, Doughty Creek; 20°01.376'S, 148°14.351'E; 2 Jul 2013; TC Goulding and field party leg.; st 118, across Doughty’s creek, coarse sandy area; MTQ. • 1 specimen 12/9 mm [2656]; same collection data as for the preceding; MTQ. • 1 specimen 50/30 mm [2661]; Bowen; 20°01.478'S, 148°14.224'E; 3 Jul 2013; TC Goulding and field party leg.; st 119, rocks on beach near a *Rhizophora* and *Avicennia* mangrove; MTQ. • 1 specimen 15/10 mm [2662]; same collection data as for the preceding; MTQ. • 1 specimen 15/10 mm [2664]; same collection data as for the preceding; MTQ. • 1 specimen 6/4 mm [2667]; same collection data as for the preceding; MTQ. • 1 specimen 9/6 mm [2646]; Bowen, Doughty Creek; 20°01.264'S, 148°14.345'E; 2 Jul 2013; TC Goulding and field party leg.; st 117, narrow *Avicennia* and *Rhizophora* mangrove, by creek, some muddy areas and some very sandy; MTQ.

New Caledonia • 1 specimen 12/7 mm [6189]; Baie de Taaré; 22°15.286'S, 167°00.808'E; 19 Sep 2018; Our Planet Reviewed Koumac 2018 expedition leg.; st KM524, intertidal sandy coral rubble flat in front of mangroves; MNHN-IM-2019-1594. • 1 specimen 41/25 mm [6195]; same collection data as for the preceding; MNHN-IM-2019-1595. • 1 specimen 33/19 mm [6209]; Nouméa, Pointe des Dorades; 22°11.507'S, 166°25.951'E; 22 Sep 2018; Our Planet Reviewed Koumac 2018 expedition leg.; st KM530, firm mud amongst muddy rocks and gravel in front of a seaward fringing Rhizophora forest; MNHN-IM-2019-1596. • 1 specimen 21/12 mm [6213]; Pointe Sauveur, Presqu’île de Ouano; 21°52.006'S, 165°49.195'E; 26 Sep 2018; Our Planet Reviewed Koumac 2018 expedition leg.; st KM538, muddy intertidal rocky flat in front of mangroves; MNHN-IM-2019-1597. • 1 specimen 25/11 mm [6220]; Pointe Vidoire, Bourail; 21°37.572'S, 165°27.595'E; 27 Sep 2018; Our Planet Reviewed Koumac 2018 expedition leg.; st KM539, landlocked coastal mangrove, small pocket of *Rhizophora stylosa* and *Avicennia marina* with a large brackish pool set behind a stony beach; MNHN-IM-2019-1598. • 1 specimen 26/16 mm [6222]; same collection data as for the preceding; MNHN-IM-2019-1599.

###### Distribution

(Fig. 6). Southern West Pacific: New South Wales (type locality) and Queensland (up to 20°S), Australia, and New Caledonia.

###### Etymology.

*Peronia sydneyensis* is named after its type locality in Sydney, New South Wales, Australia: *sydneyensis* is a latinized adjective that agrees in gender (feminine) with the generic name (ICZN 1999: Article 31.2).

###### Habitat

**Figure 49.**
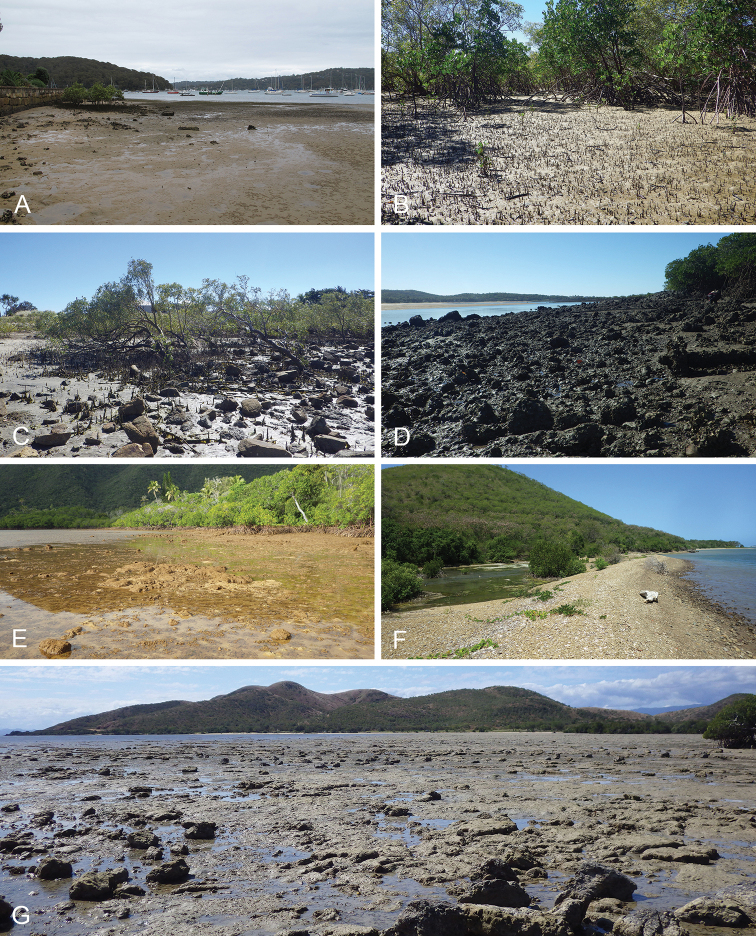
Habitats, *Peronia sydneyensis***A** Australia, New South Wales, sand, next to a small patch of mangrove, and rocks on sandy beach (st 39, type locality) **B** Australia, Queensland, *Rhizophora*, across Doughty’s creek, coarse sandy area (st 118) **C** Australia, Queensland, *Sonneratia*, rocks on beach near a *Rhizophora* and *Avicennia* mangrove (st 119) **D** Australia, Queensland, by boat ramp, mangrove margin with large rocks by creek (st 121) **E** New Caledonia, intertidal sandy coral rubble flat in front of a mangrove (st KM 524) **F** New Caledonia, landlocked coastal mangrove, small pocket of *Rhizophora* and *Avicennia* with a large brackish pool set behind a stony beach (st KM 539) **G** New Caledonia, muddy intertidal rocky flat in front of mangroves (st KM 538).

(Fig. 49). Unlike most other *Peronia* species, which are found in the rocky intertidal, *P. sydneyensis* is primarily found on muddy or coarse sand.

###### Color and morphology of live animals

**Figure 50.**
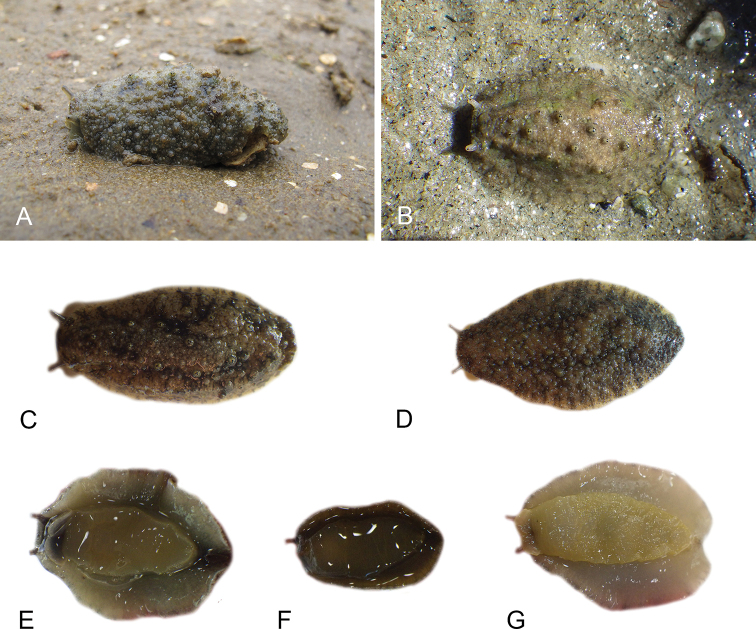
Live animals, *Peronia sydneyensis***A** holotype, dorsal view, 30 mm long [1516 H], Australia, New South Wales (AM C.468916.001) **B** dorsal view, 15 mm long [2664], Australia, Queensland (MTQ) **C** dorsal view, 9 mm long [2646], Australia, Queensland (MTQ) **D** dorsal view, 50 mm long [2661], Australia, Queensland (MTQ) **E** ventral view, 15 mm long [2662], Australia, Queensland (MTQ) **F** Ventral view, 6 mm long [2667], Australia, Queensland (MTQ) **G** Ventral view, same as **B**.

**Figure 51.**
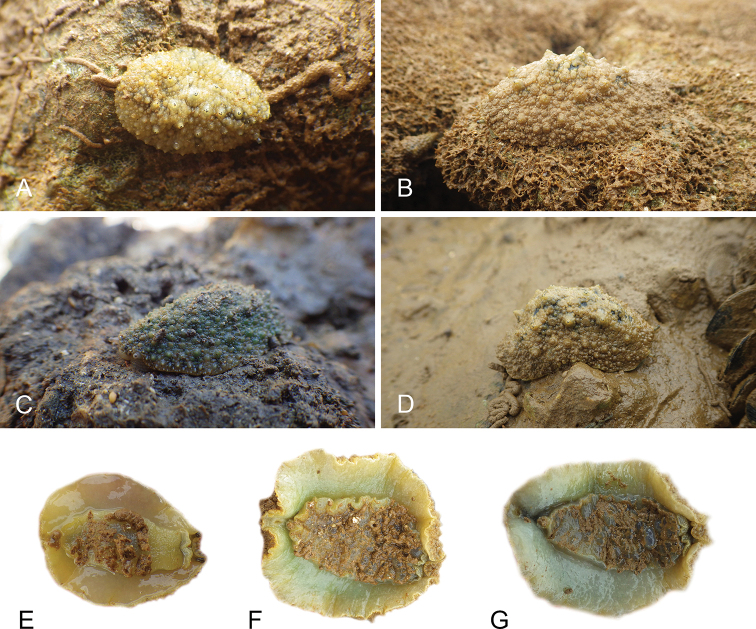
Live animals, *Peronia sydneyensis*, New Caledonia **A** dorsal view, 12 mm long [6189] (MNHN-IM-2019-1594) **B** dorsal view, 41 mm long [6195] (MNHN-IM-2019-1595) **C** dorsal view, 26 mm long [6222] (MNHN-IM-2019-1599) **D** dorsal view, 33 mm long [6209] (MNHN-IM-2019-1596) **E** ventral view, same as **A**; **F** ventral view, same as **B**; **G** ventral view, same as **D**.

(Figs 50, 51). The dorsal notum is greenish brown, light to dark, mottled with darker and lighter areas. The color of the dorsal papillae varies as that of the background itself. The ventral surface (foot and hyponotum) varies from whitish to dark grey, including yellowish, bluish, and greenish, and can change rapidly in any given individual. The ocular tentacles are brown-grey, like the head. The dorsal notum of live animals is covered by dozens of papillae of various sizes. Some papillae bear black dorsal eyes at their tip. The number of papillae with dorsal eyes is variable (from 8 to 16). The largest specimens are 30 mm long (New South Wales), 50 mm long (Queensland), and 41 mm long (New Caledonia).

###### Digestive system

**Figure 52.**
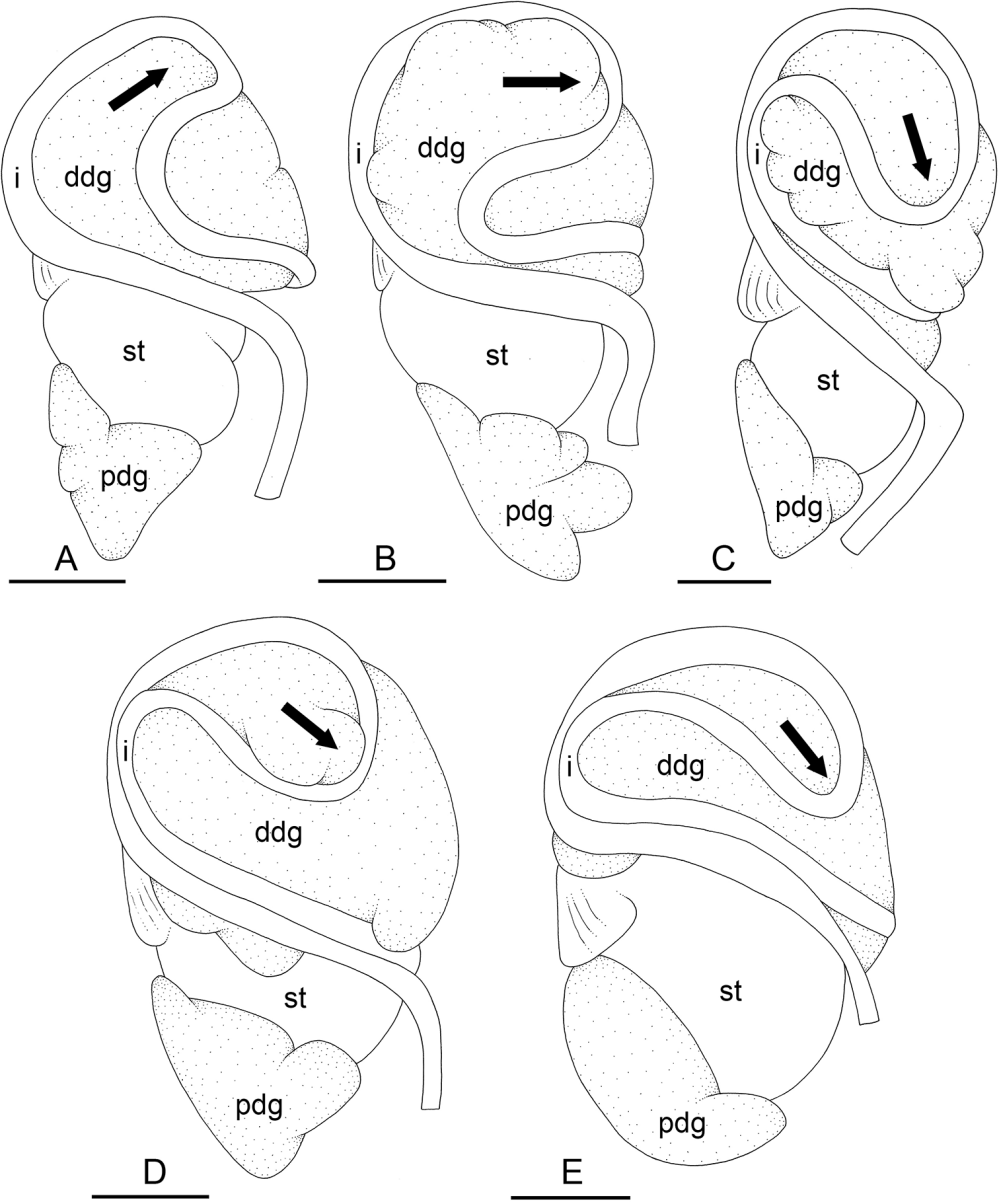
Digestive system, dorsal view, *Peronia sydneyensis*. The arrow indicates the orientation of the transitional loop **A** holotype, Australia, New South Wales, [1516 H] (AM C.468916.001) **B** Australia, New South Wales, [1517] (AM C.468915.001) **C** Australia, Queensland, [2680] (MTQ) **D** New Caledonia, [6195] (MNHN-IM-2019-1595) **E** New Caledonia, [6209] (MNHN-IM-2019-1596). Scale bars: 4 mm (**A, C**), 3 mm (**B, D, E**). Abbreviations: ddg dorsal digestive gland, i intestine, pdg posterior digestive gland, st stomach.

**Figure 53.**
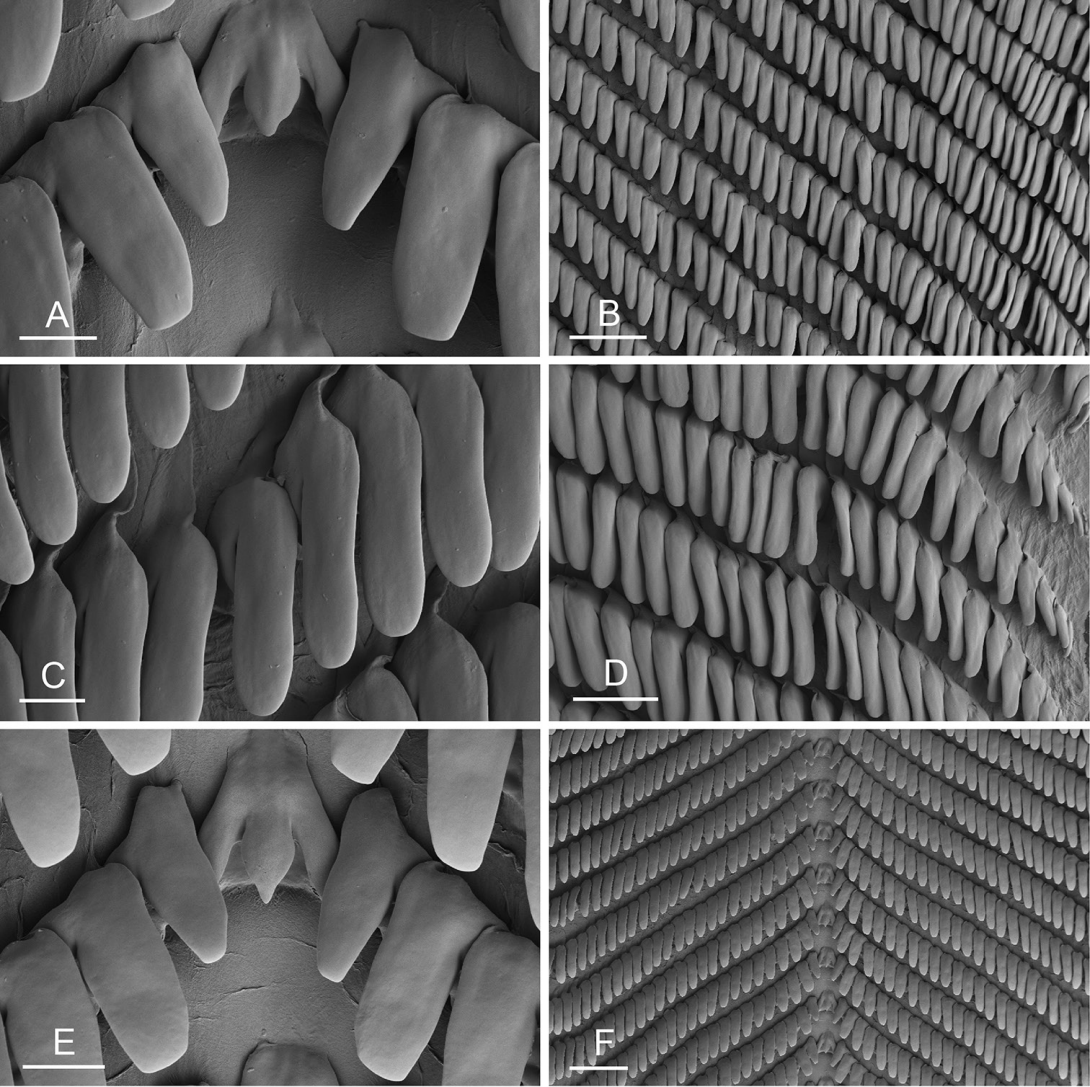
Radula, *Peronia sydneyensis*, Australia, New South Wales **A–D** holotype [1516 H] (AM C.468916.001) **E, F** [1517] (AM C.468915.001) **A** rachidian and innermost lateral teeth **B** lateral teeth **C** lateral teeth **D** lateral and outermost lateral teeth **E** rachidian and innermost lateral teeth **F** rachidian and lateral teeth. Scale bars: 20 μm (**A, C, E**), 50 μm (**D**), 100 μm (**B, F**).

**Figure 54.**
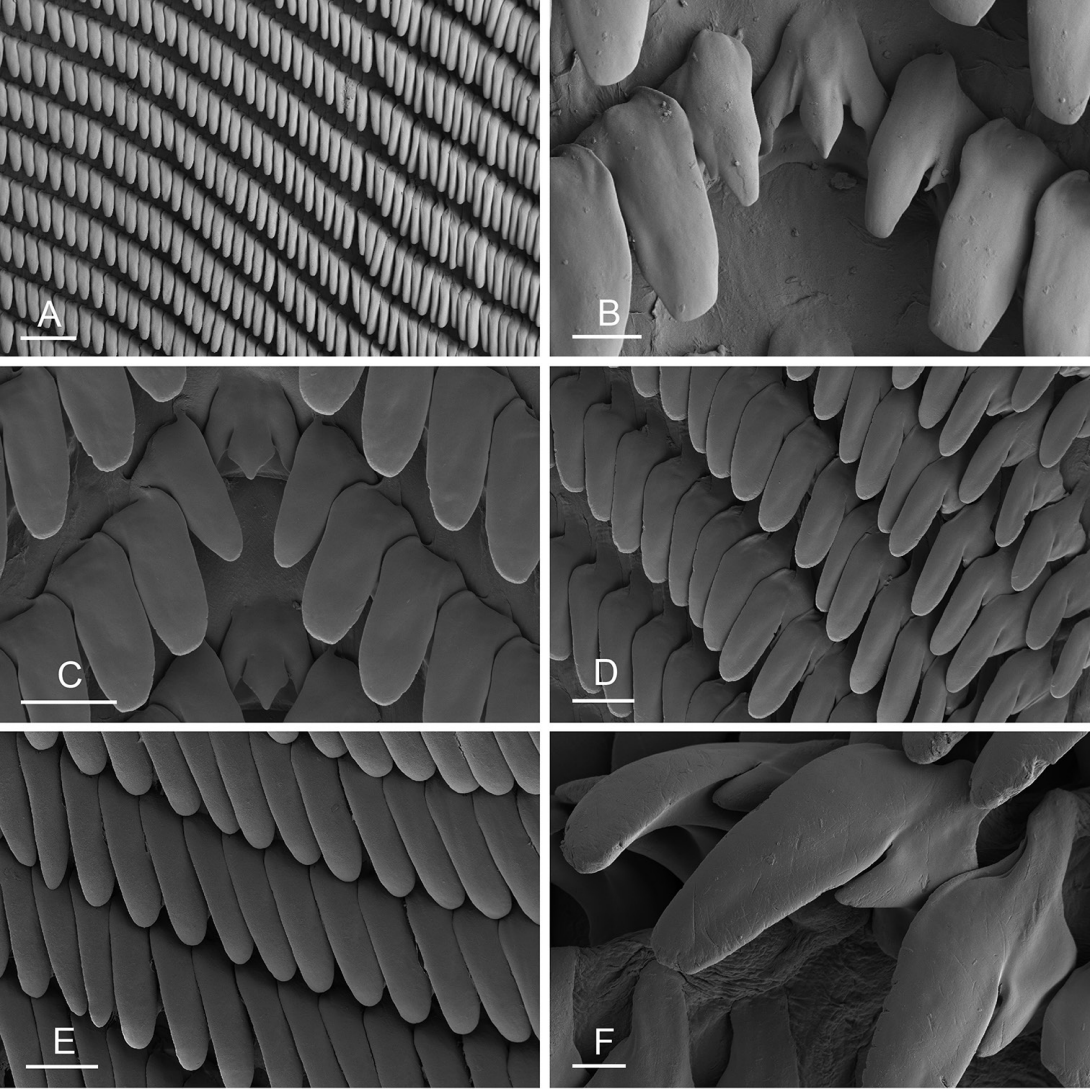
Radula, *Peronia sydneyensis***A, B** Australia, Queensland **C–F** New Caledonia **A** lateral teeth, [2680] (MTQ) **B** rachidian and innermost lateral teeth, [2680] (MTQ) **C** rachidian and innermost lateral teeth, [6189] (MNHN-IM-2019-1594) **D** lateral and outermost lateral teeth, [6189] (MNHN-IM-2019-1594) **E** lateral teeth, [6195] (MNHN-IM-2019-1595) **F** lateral teeth, [6220] (MNHN-IM-2019-1598). Scale bars: 100 μm (**A**), 20 μm (**B–D**), 40 μm (**E**), 10 μm (**F**).

(Figs 52–54). Examples of radular formulae are presented in Table 5. The median cusp of the rachidian teeth is approximately 40 μm long. The hook of the lateral teeth is approximately 80 μm long. The intestinal loops are of type I, with a transitional loop oriented between 3 and 6 o’clock; exceptionally, the transitional loop is oriented at 2 o’clock.

###### Reproductive system

**Figure 55.**
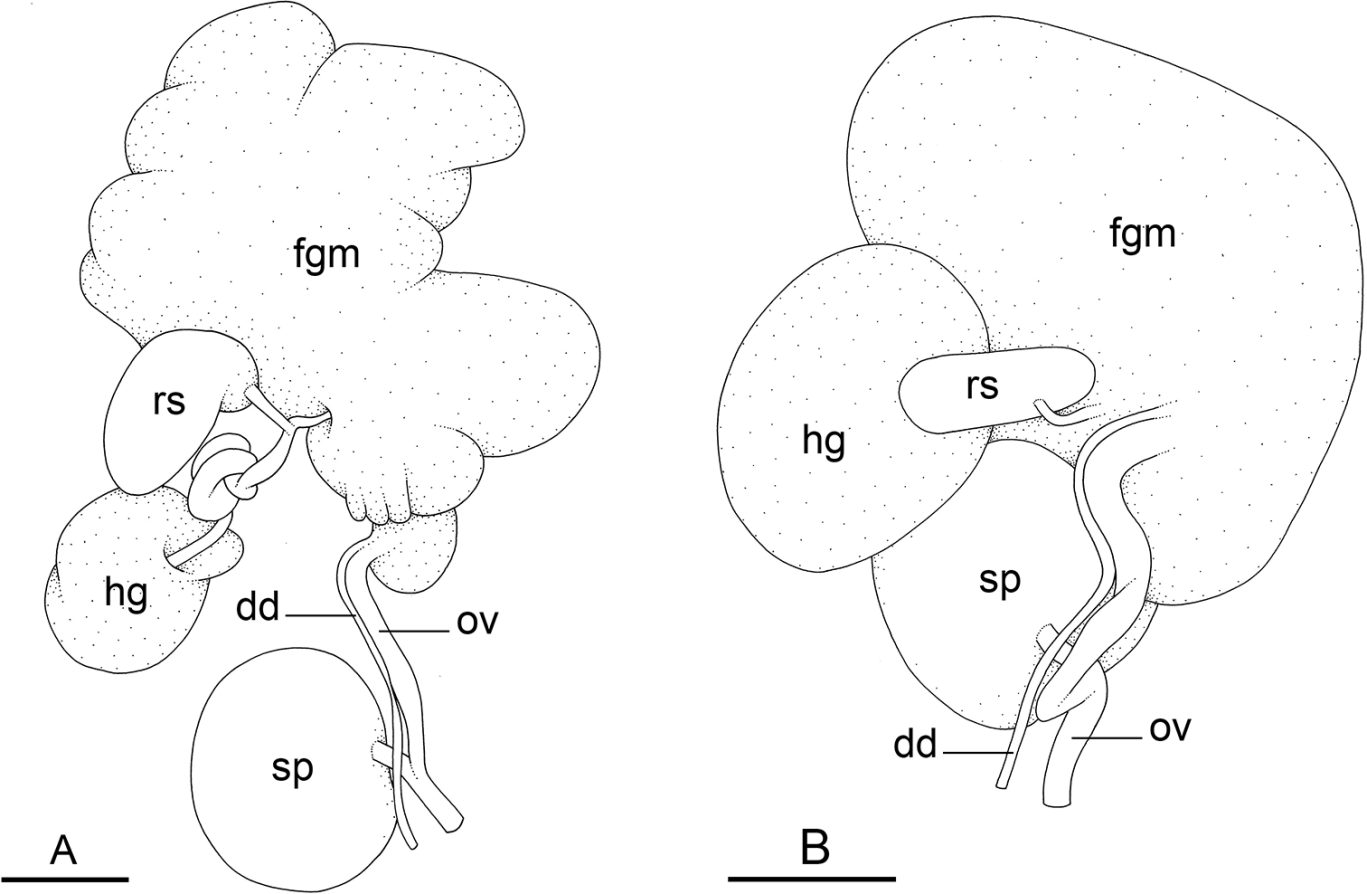
Posterior, hermaphroditic (female) reproductive system, *Peronia sydneyensis***A** holotype, Australia, New South Wales, [1516 H] (AM C.468916.001) **B** New Caledonia, [6195] (MNHN-IM-2019-1595). Scale bars: 2 mm (**A, B**). Abbreviations: dd deferent duct, fgm female gland mass, hg hermaphroditic gland, ov oviduct, rs receptaculum seminis, sp spermatheca

**Figure 56.**
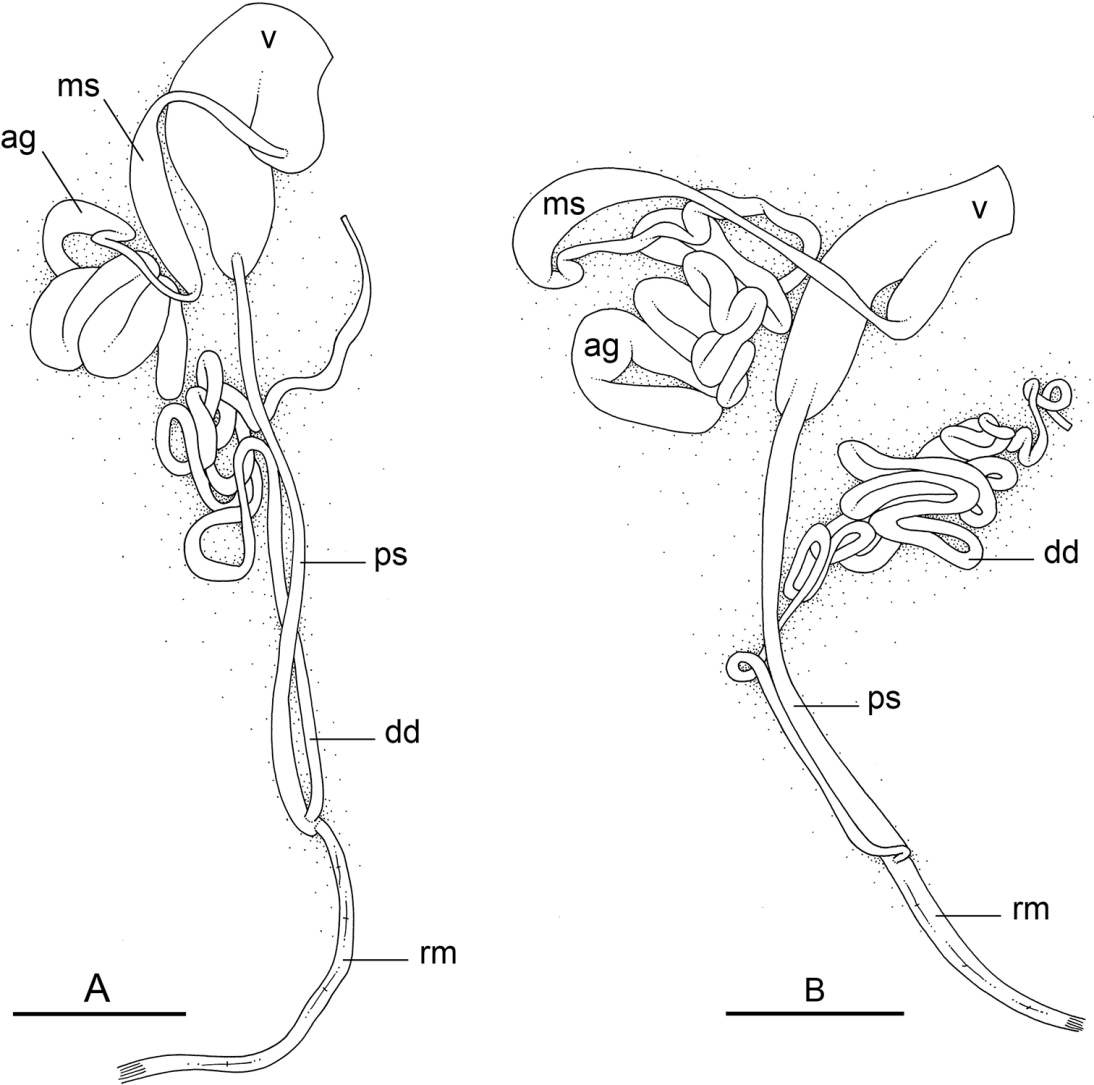
Anterior, male, copulatory apparatus, *Peronia sydneyensis***A** holotype, Australia, New South Wales, [1516 H] (AM C.468916.001) **B** New Caledonia, [6195] (MNHN-IM-2019-1595). Scale bars: 3 mm (**A, B**). Abbreviations: ag accessory penial gland, dd deferent duct, ms muscular sac, ps penial sheath, rm retractor muscle, v vestibule.

**Figure 57.**
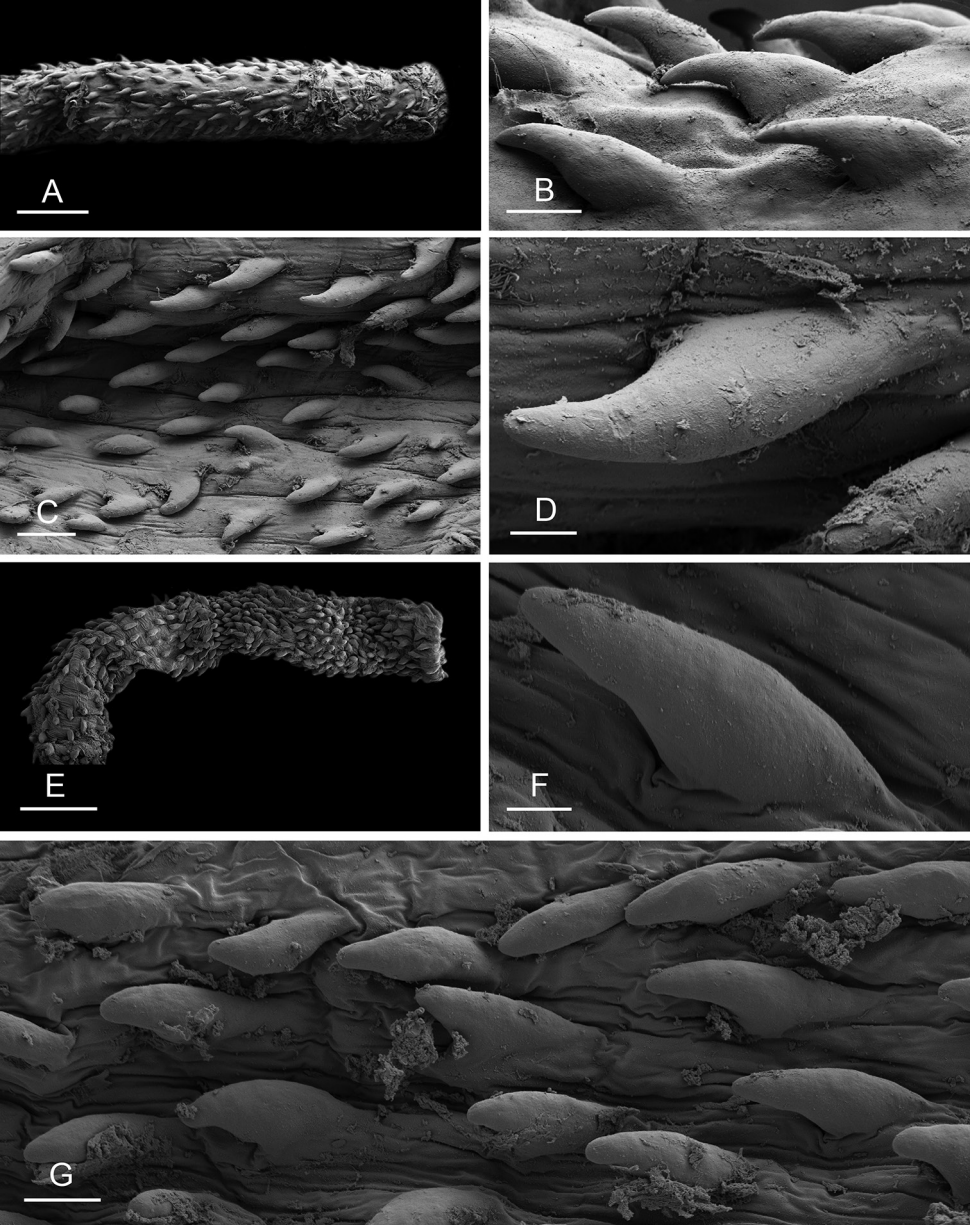
Penis and penial hooks, *Peronia sydneyensi***A** holotype, [1516 H], Australia, New South Wales (AM C.468916.001) **B** same as **A**; **C** [2680], Australia, Queensland (MTQ) **D** same as **C**; **E** [6220], New Caledonia (MNHN-IM-2019-1598) **F** [6195], New Caledonia (MNHN-IM-2019-1595) **G** same as **F**. Scale bars: 100 μm (**A**), 10 μm (**B, G**), 20 μm (**C**), 5 μm (**D**), 80 μm (**E**), 4 μm (**F**).

**Figure 58.**
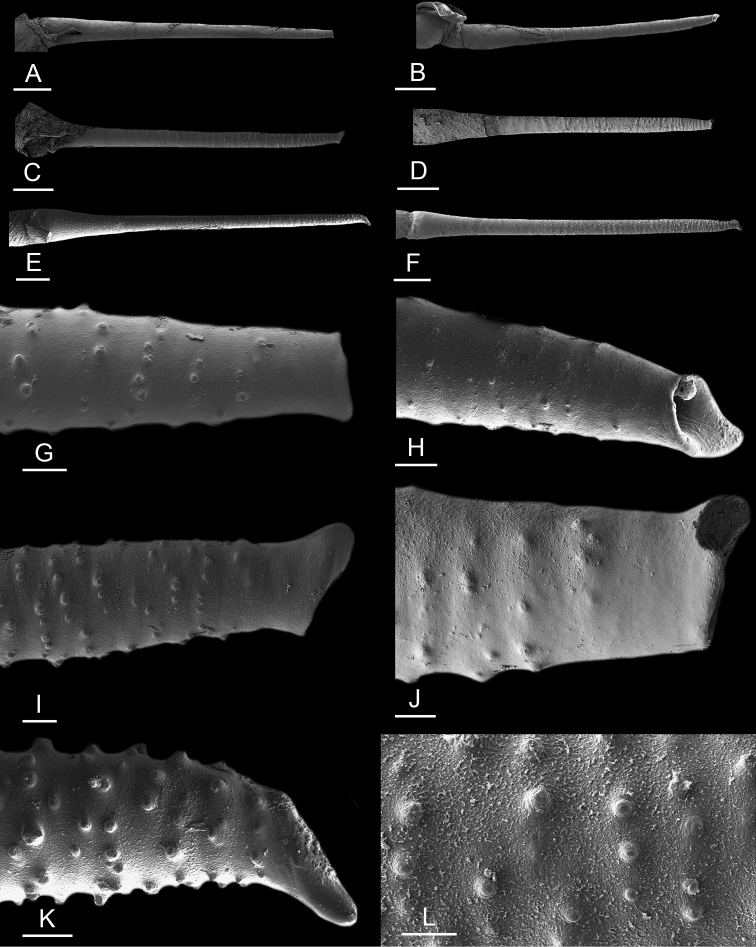
Accessory penial gland spine, *Peronia sydneyensis***A–C, G–I** New Caledonia **D, J** Australia, New South Wales **E, F, K, L** Australia, Queensland **A** [6209] (MNHN-IM-2019-1596) **B** [6213] (MNHN-IM-2019-1597) **C** [6222] (MNHN-IM-2019-1599) **D** holotype, [1516 H] (AM C.468916.001) **E** [2661] (MTQ) **F** [2664] (MTQ) **G** same as **A**; **H** same as **B**; **I** same as **C**; **J** same as **D**; **K** same as **E**; **L** same as **F**. Scale bars: 100 μm (**A–F**), 10 μm (**G–I, K**), 6 μm (**J, L**).

(Figs 55–58). In the anterior (male) parts, the muscular sac of the accessory penial gland is less than 10 mm long. The hollow spine of the accessory penial gland is narrow, elongated, and straight or slightly curved, and its shape (including at its tip) varies between individuals. Its length ranges from 0.6 mm ([2680] MTQ) to 1 mm ([2661] MTQ). Its diameter at the conical base ranges from 90 to 100 μm. Its diameter at the tip measures 20–50 μm. The retractor muscle is shorter or longer than the penial sheath and inserts near the heart. Inside the penial sheath, the penis is a narrow, elongated, soft, hollow tube. Its distal end bears conical hooks which are less than 30 μm long.

###### Diagnostic features

(Table 4). *Peronia sydneyensis* is characterized by unique and distinctive protuberances on the spine of the accessory penial gland (Fig. 58). These strong protuberances were observed in all individuals. Protuberances can also be observed (as exceptional cases) in other species but they are always much smaller in size (Figs 37D, 104B, 105F). In addition, *Peronia sydneyensis* is characterized by a unique combination of anatomical traits: intestinal loops of type I (with a transitional loop oriented between 3 and 6 o’clock), retractor muscle inserting at the posterior end of the visceral cavity, spine of the accessory penial gland less than 1 mm long. *Peronia sydneyensis* is distinct anatomically from *P. willani*, with which it is most closely related (Figs 2–4), and from *P. verruculata*, with which it overlaps geographically in Queensland and New Caledonia (Fig. 6).

###### Remarks.

A new species name is needed because no existing name applies to the species described here. The records of *Onchidium verruculatum* from New South Wales (Bretnall 1919: 310; Dakin 1947: 144; Smith and Kershaw 1979: 92; Hutchings and Recher 1982: 119; Hyman 1999) are most likely records of *Peronia sydneyensis*, the only *Peronia* species known in New South Wales based on current data (Fig. 6). Some of these records (or even all of them) could be a combination of both *P. sydneyensis* and *P. verruculata*: the southernmost locality of *P. verruculata* (unit #1) is in MacKay, Queensland (21°22'S), but given that *P. verruculata* tolerates colder waters in Japan (up to at least 33°40'N), it is possible that it is also present in New South Wales. *Peronia sydneyensis* was collected only in Sydney (33°39'S), but it is not excluded that both species are sympatric as far south as Sydney. Additional fresh material between southern Queensland and New South Wales is needed to determine more precisely the geographic range of each species. Note that the intestinal loops of type II by Hyman (1999: fig. 7B) illustrate the digestive system of a misidentified individual (most likely *Paromoionchis daemelii*, easily confused in the field with *Peronia sydneyensis*). Finally, note that the specimen [734] (AM C.459511) was tentatively referred to as *Peronia* sp. 3 by Dayrat et al. (2011).

##### Peronia willani

Taxon classificationAnimaliaSystellommatophoraOnchidiidae

Dayrat & Goulding sp. nov.

http://zoobank.org/FE5553D0-C9E2-4C5A-80D7-08A8C0AFC46A

**Figure 59.**
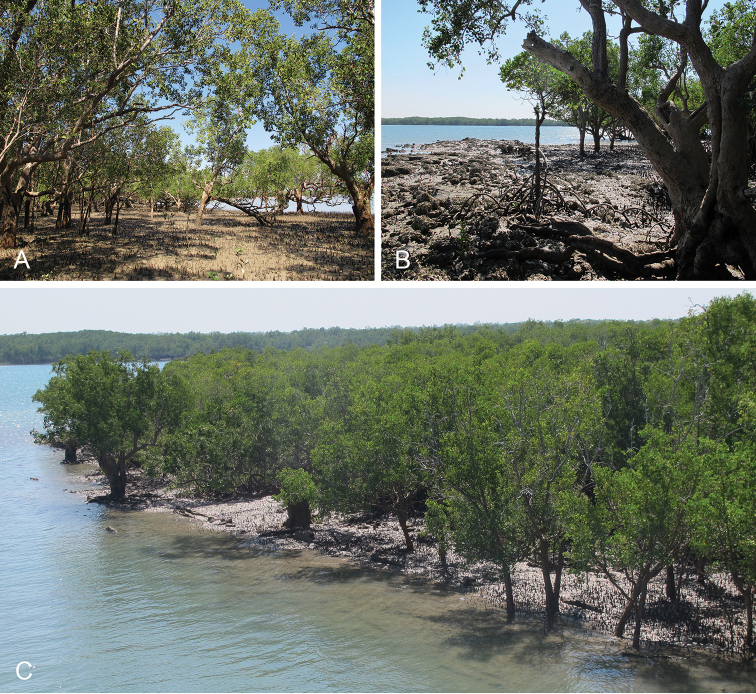
Habitats, *Peronia willani*, Australia, Northern Territory **A** large and open forest of *Sonneratia alba* with soft mud (st 62, type locality) **B***Avicennia* mangrove with sandy mud (st 61) **C** view from the bridge to Channel Island, same as **B**.

**Figure 60.**
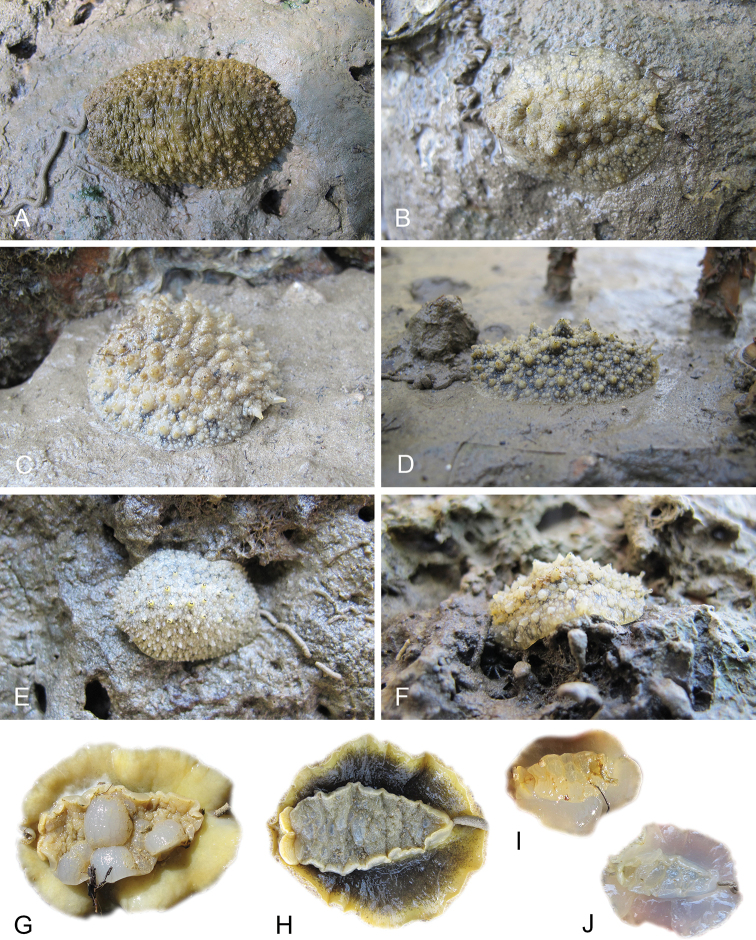
Live animals, *Peronia willani*, Australia, Northern Territory **A** holotype, dorsal view, 50 mm long [1628 H] (NTM P.57625) **B** dorsal view, 35 mm long [1655] (NTM P.57626) **C** dorsal view, 65 mm long [1620] (NTM P.57626) **D** dorsal view, 40 mm long [1668] (NTM P.57627) **E** dorsal view, 15 mm long [1670] (NTM P.57627) **F** dorsal view, 15 mm long [1629] (NTM P.57627) **G** ventral view, same as **A**; **H** ventral view, 60 mm long [1626] (NTM P.57627) **I** ventral view, 10 mm long [1625] (NTM P.57627) **J** ventral view, 18 mm long [1653] (NTM P.57626).

**Figure 61.**
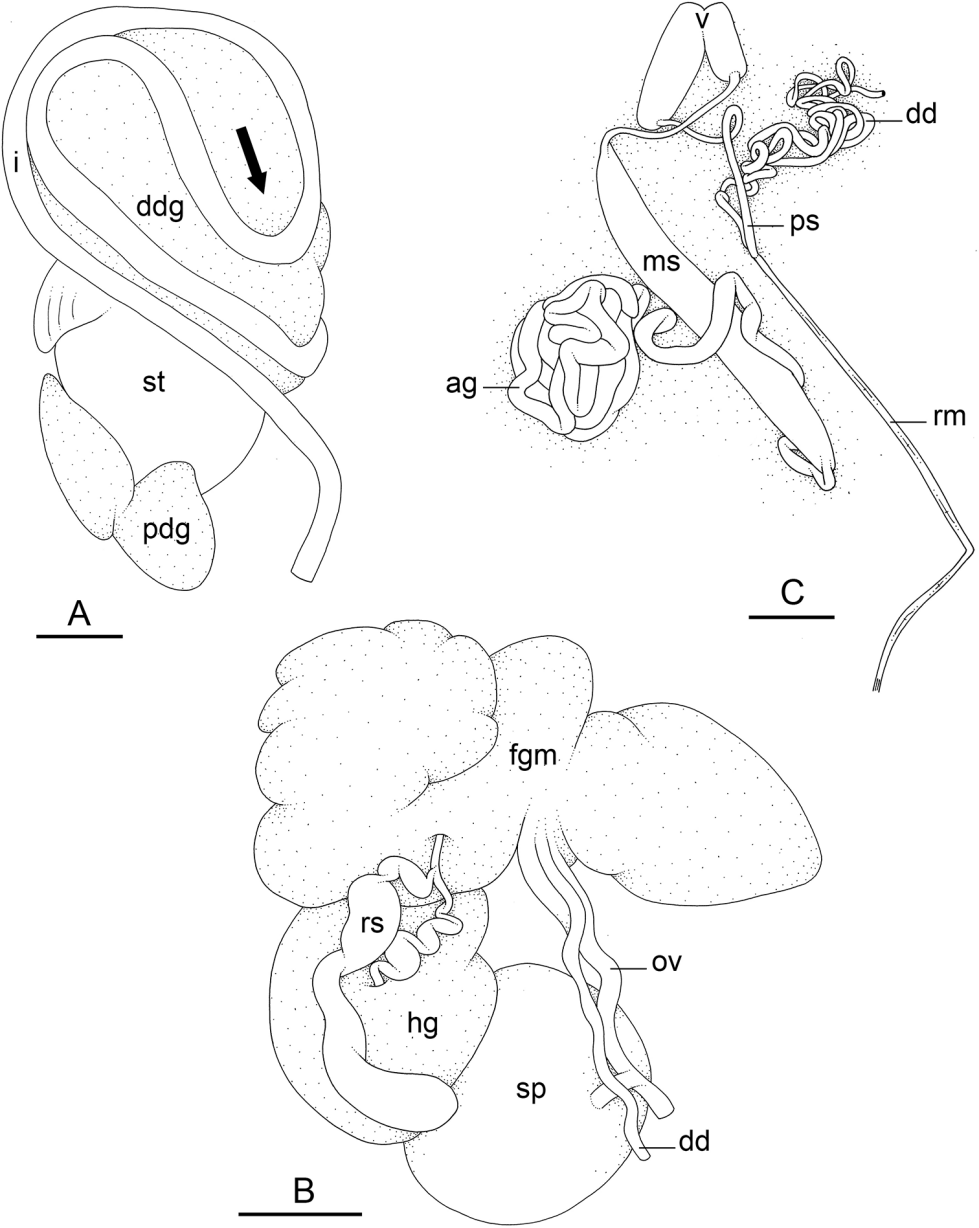
*Peronia willani*, Australia, Northern Territory, holotype [1628 H] (NTM P.57625) **A** digestive system, dorsal view, the arrow indicates the orientation of the transitional loop **B** posterior, hermaphroditic (female) reproductive system **C** anterior, male, copulatory apparatus. Scale bars: 5 mm (**A, C**), 4 mm (**B**). Abbreviations: ag accessory penial gland, dd deferent duct, ddg dorsal digestive gland, fgm female gland mass, hg hermaphroditic gland, i intestine, ms muscular sac, ov oviduct, pdg posterior digestive gland, ps penial sheath, rm retractor muscle, rs receptaculum seminis, sp spermatheca, st stomach, v vestibule.

**Figure 62.**
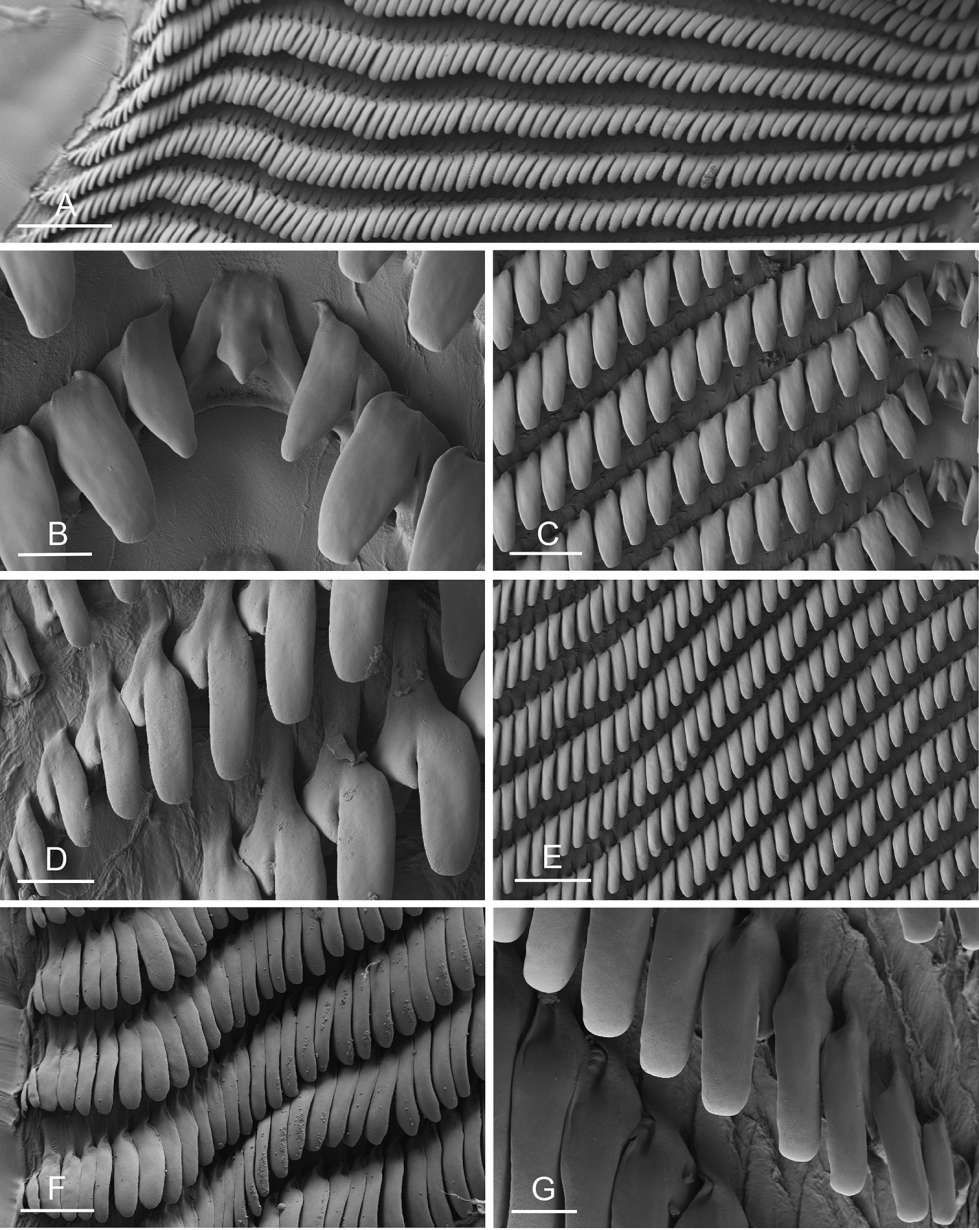
Radula, *Peronia willani*, Australia, Northern Territory **A** holotype [1628 H] (NTM P.57625) **B–E** [1668] (NTM P.57627) **F** [1620] (NTM P.57626) **G** [1626] (NTM P.57627) **A** left half rows of teeth **B** rachidian and innermost lateral teeth **C** rachidian and innermost lateral teeth **D** outermost lateral teeth **E** lateral teeth **F** outermost lateral teeth **G** outermost lateral teeth. Scale bars: 200 μm (**A**), 20 μm (**B, D, G**), 60 μm (**C, F**), 100 μm (**E**).

**Figure 63.**
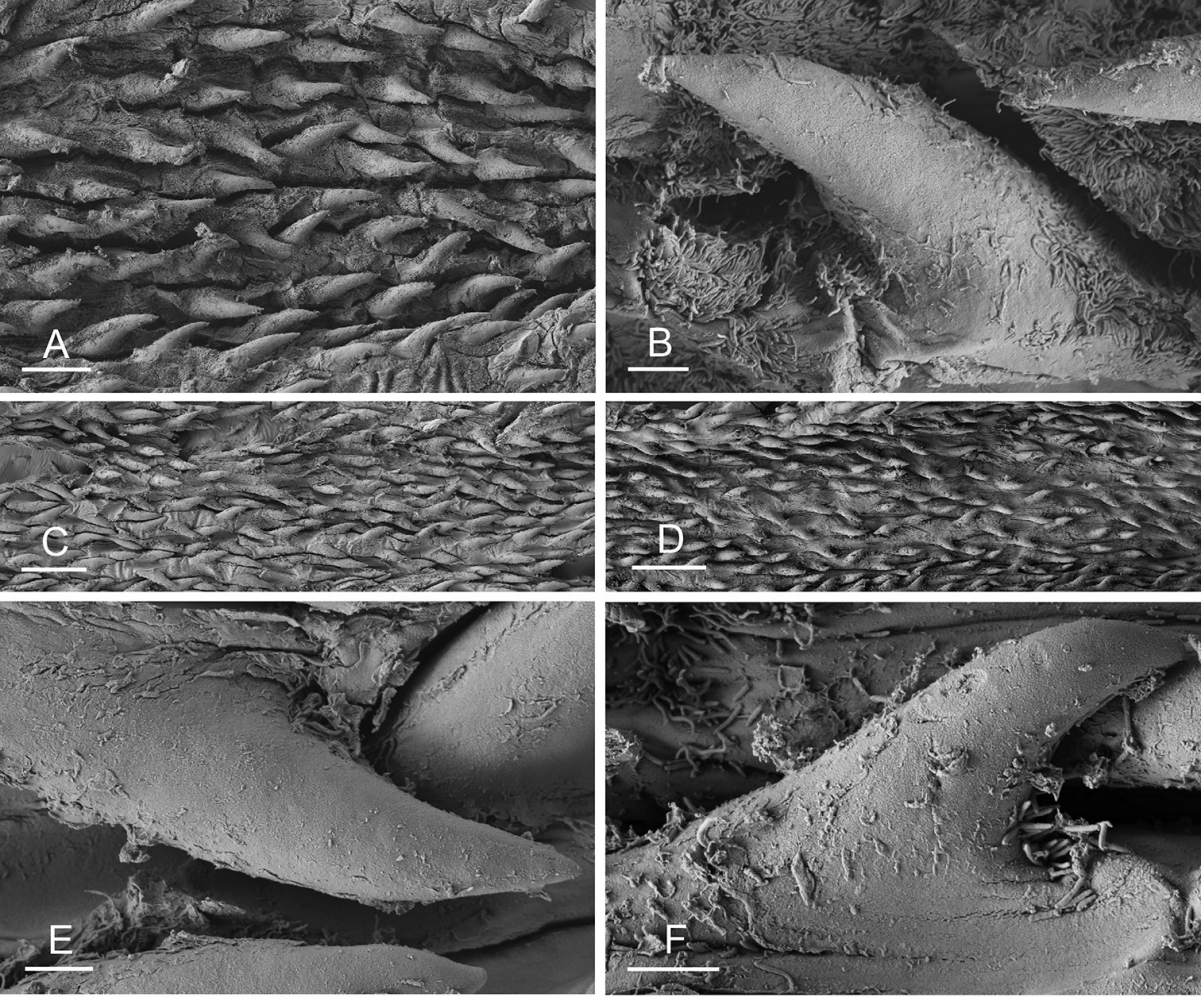
Penial hooks, *Peronia willani*, Australia, Northern Territory **A** holotype, [1628 H] (NTM P.57625) **B** same as **A**; **C** [1620] (NTM P.57626) **D** [1626] (NTM P.57627) **E** same as **C**; **F** same as **D**. Scale bars: 40 μm (**A**), 4 μm (**B, E, F**), 60 μm (**C, D**).

**Figure 64.**
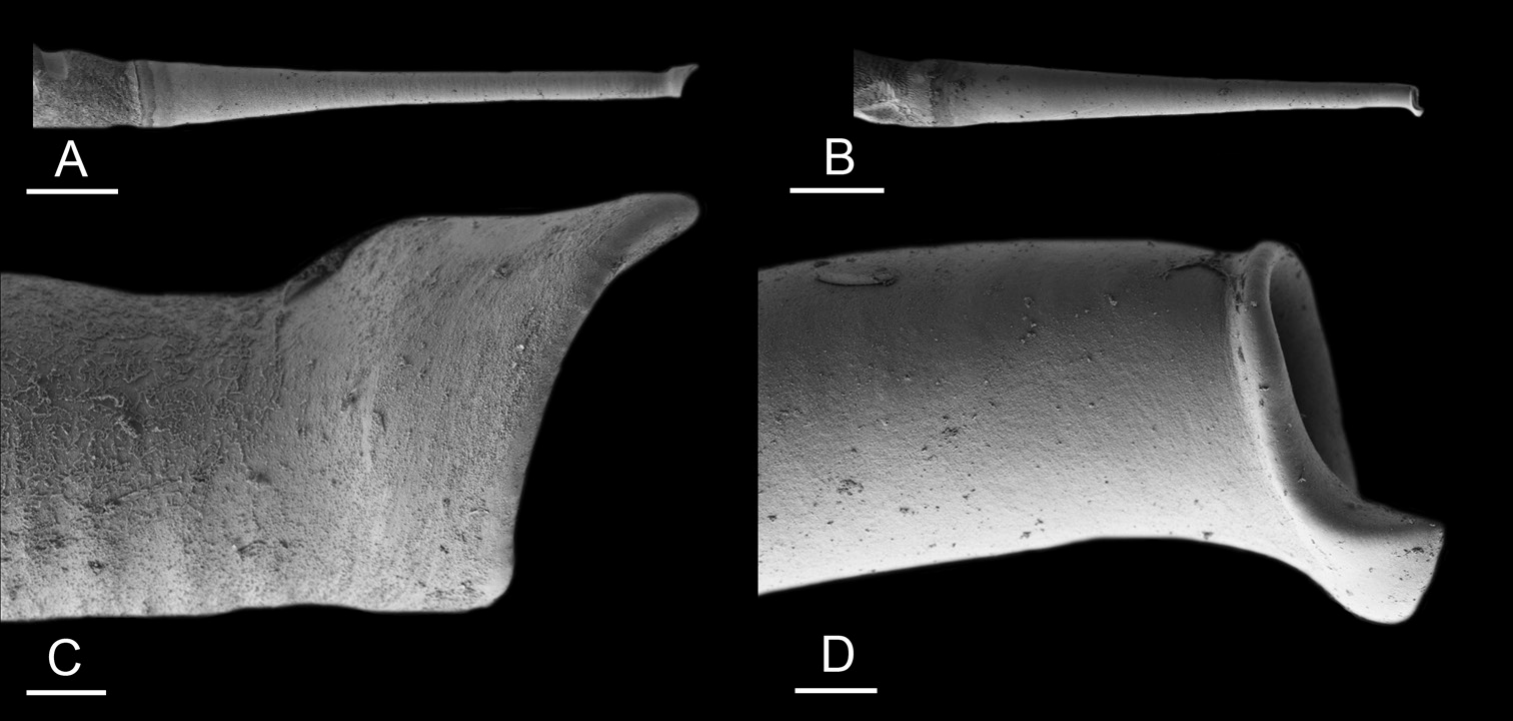
Accessory penial gland spine, *Peronia willani*, Australia, Northern Territory **A, C** holotype, [1628 H] (NTM P.57625) **B, D** [1620] (NTM P.57626). Scale bars: 300 μm (**A, B**), 20 μm (**C, D**).

Figs 59 , 60 , 61 , 62 , 63 , 64

###### Type material.

***Holotype.*** Australia • holotype, hereby designated, 50/35 mm [1628 H]; Northern Territory, Darwin, Talc Head; 12°28.765'S, 130°46.297'E; 15 Aug 2012; B Dayrat and field party leg.; station 62, large and open forest of *Sonneratia alba* with soft mud; NTM P.57625.

###### Additional material examined.

Australia • 4 specimens 65/45 mm [1620], 18/14 mm [1653], 60/50 mm [1654], and 35/25 mm [1655]; Northern Territory, Darwin, on the right side of the road just before bridge to Channel Island; 12°33.228'S, 130°52.580'E; 14 Aug 2012; B Dayrat and field party leg.; station 61, *Avicennia* mangrove with sandy mud; NTM P.57626. • 9 specimens 35/25 mm [1667], 60/50 mm [1623], 40/25 mm [1668], 22/18 mm [1669], 8/5 mm [1624], 10/7 mm [1625], 15/10 mm [1670], 60/40 mm [1626], and 15/12 mm [1629]; same collection data as for the holotype; NTM P.57627.

###### Additional material examined

**(historical museum collections).** Australia • 1 specimen 38/30 mm; Northern Territory, Port Darwin; Mac Leay leg.; 12°30'S, 130°50'E; 1 Jan 1881; SMNH 180715.

###### Distribution

(Fig. 6). Endemic to Darwin, Northern Territory, Australia.

###### Etymology.

*Peronia willani* is named after Richard Willan, senior curator of mollusks at the Museum and Art Gallery of the Northern Territory, Darwin, Australia, who kindly and generously helped us during our field expedition around Darwin.

###### Habitat

(Fig. 59). Unlike most other *Peronia* species, which are usually found in the rocky intertidal, *P. willani* is primarily found on sandy mud or even directly on mud.

###### Color and morphology of live animals

(Fig. 60). The color of the dorsal notum is highly variable, from nearly whitish to dark brown and greenish, most often mottled with darker and lighter areas. The color of the dorsal papillae varies as that of the background itself, but dorsal papillae can also be lighter (yellowish-greenish) than the background. The ventral surface (foot and hyponotum) varies from whitish (almost transparent) to yellowish and can change rapidly in any given individual. Occasionally, a black ring is present on the hyponotum around the pedal sole. The ocular tentacles are brown-grey, like the head. The dorsal notum of live animals is covered by dozens of papillae of various sizes. Some papillae bear black dorsal eyes at their tip. The number of papillae with dorsal eyes is variable (from 10 to 25). The largest specimens are 65 mm long.

###### Digestive system

(Figs 61A, 62). Examples of radular formulae are presented in Table 5. The median cusp of the rachidian teeth is approximately 30 μm long. The hook of the lateral teeth is approximately 100 μm long. The intestinal loops are of type I, with the transitional loop oriented between 3 to 6 o’clock.

###### Reproductive system

(Figs 61B, C, 63, 64). In the anterior (male) parts, the muscular sac of the accessory penial gland is less than 25 mm long. The hollow spine of the accessory penial gland is narrow, elongated, and straight or slightly curved, and its shape (including at its tip) varies between individuals. Its length ranges from 1.5 mm ([1620] NTM P.57626) to 1.9 mm ([1628 H] NTM P.57625). Its diameter at the conical base ranges from 240 to 250 μm. Its diameter at the tip ranges from 80 to 100 μm. The retractor muscle is shorter or longer than the penial sheath and inserts near the heart. Inside the penial sheath, the penis is a narrow, elongated, soft, hollow tube. Its distal end bears conical hooks which are less than 37 μm long.

###### Diagnostic features

(Table 4). *Peronia willani* is characterized by a unique combination of anatomical traits: intestinal loops of type I (with a transitional loop oriented between 3 and 6 o’clock), retractor muscle inserting at the posterior end of the visceral cavity, muscular sac up to 25 mm, spine of the accessory penial gland between 1.5 and 1.9 mm long. *Peronia willani* is anatomically distinct from *P. sydneyensis*, with which it is most closely related (Figs 2–4), and from *P. verruculata*, from which it is close geographically (Fig. 6).

###### Remarks.

A new species name is needed because no existing name applies to the species described here. A specimen from Darwin, Northern Territory, preserved in Stockholm (SMNH 180715) identified as *O. verruculatum* by Hoffmann (1928: 73) is identified here as *P. willani* because of its massive (18 mm long) muscular sac (Table 4). Also, to our knowledge, *P. verruculata* is not present in Northern Territory (Fig. 6).

##### Peronia verruculata

Taxon classificationAnimaliaSystellommatophoraOnchidiidae

(Cuvier, 1830)

Figs 65 , 66 , 67 , 68 , 69 , 70 , 71 , 72 , 73 , 74 , 75 , 76 , 77 , 78 , 79 , 80 , 81 , 82 , 83 , 84 , 85 , 86 , 87 , 88 , 89 , 90 , 91 , 92 , 93 , 94 , 95 , 96 , 97 , 98 , 99 , 100 , 101 , 102 , 103 , 104 , 105 , 106 , 107 , 108 , 109

Onchidium verruculatum Cuvier, 1830: 281; Semper 1880: 255–257, pl. 22, figs 3, 4; 1882: pl. 21, fig. 1 [only in part]; Bergh 1884a: 148–151, pl. VII, figs 7–12, pl. VIII, fig. 14; Farran 1905: 358–359, pl. VI, figs 13–22; Odhner 1919: 23; Hoffmann 1928: 44, 72–75 [only in part].
Peronia verruculata (Cuvier, 1830): Keferstein 1865a: pl. CIV, figs 9–12; Britton 1984: 183–184, fig. 2 [only in part]; Sun et al. 2014: 63; Liu et al. 2015: 753–754; Chang et al. 2018: 149–165, figs 1–8; Xu et al. 2018: 3.
Onchidium ferrugineum Lesson, 1831a: 128–130; Lesson 1831b: 300–302; Lesson 1832: 36–37, fig. 32.
Peronia ferruginea (Lesson, 1831a): Lesson 1833: 3 pp. with no pagination, pl. 19, figs 1, 2; Oken 1834b: 269–270; Gray 1850: 117; Adams and Adams 1855: 235; Tapparone Canefri 1883: 214.
Peronia savignii Récluz, 1869: 61. Syn. nov.
Peronia mauritiana : Mörch 1872a: 28; 1872b: 325 [non Peronia mauritiana Blainville, 1824].
Onchidium branchiferum Plate, 1893: 141, 183–185, pl. 11, figs 63, 64; Hoffmann 1928: 68, 75. Syn. nov.
Peronia branchifera (Plate, 1893): Labbé 1934a: 194.
Onchidium elberti Simroth, 1920: 297–298, pl. XX, figs 51–54.
Onchidium astridae Labbé, 1934b: 77–78, figs 18, 38, pl. I, fig. 5. Syn. nov.
Scaphis astridae (Labbé, 1934b): Labbé 1934a: 213, fig. 46.
Peronia gaimardi Labbé, 1934a: 194–195, fig. 8. Syn. nov.
Peronia anomala Labbé, 1934a: 195–196. Syn. nov.
Paraperonia gondwanae Labbé, 1934a: 199–200, figs 19–22 [only in part]. Syn. nov.
Scaphis viridis Labbé, 1934a: 207–208, figs 31–34. Syn. nov.
Scaphis carbonaria Labbé, 1934a: 208–209, figs 35, 36. Syn. nov.
Scaphis gravieri Labbé, 1934a: 209–211, figs 37–40. Syn. nov.
Scaphis tonkinensis Labbé, 1934a: 211–212, figs 41–43. Syn. nov.
Scaphis lata Labbé, 1934a: 212, figs 44–45. Syn. nov.
Onchidium durum Labbé, 1934a: 220–221, figs 55–57. Syn. nov.
Peronia sp. (“group V”): Tagaki et al. 2019: 34.
Peronia persiae Maniei et al. 2020a: 507–514, figs 2–10. Syn. nov.

###### Type material.

***Lectotype*** (*O. verruculatum*). Red Sea • lectotype, hereby designated, 30/26 mm; [locality not specified in the original description but most likely from the Red Sea]; MNHN-IM-2000-22941. One paralectotype was not found at the MNHN. Cuvier (1830: 46) did not accompany the name *Onchidium verruculatum* with a description but referred to a series of eight drawings by Savigny (1817: pl. 2, figs 3.1–3.8) in the famous *Description de l’Egypte* (“Descr. de l’Eg., moll. gaster., pl. II, f. 3”). For a collation of the *Description de l’Egypte*, including the text by Audouin (1826), see Baring (1838) and Sherborn (1897). Cuvier’s reference to Savigny’s illustrations is an indication (ICZN 1999: Article 12.2). *Onchidium verruculatum* is available and the type series consists of the specimens illustrated by Savigny (ICZN 1999: Article 72.4). Savigny illustrated two individuals which could belong to two distinct species given that there are (at least) two *Peronia* species in the Red Sea. One specimen (figs 3.1, 3.2) is much smaller than the other (fig. 3.3). One of the two individuals illustrated by Savigny is preserved at the MNHN in a jar with a label reading: “Savigny Description Egypte, Mer Rouge [Red Sea], syntype, pl. 2, fig. 3” (MNHN-IM-2000-22941). The specimen preserved at the MNHN looks like the individual illustrated on Savigny’s figures 3.1, 3.2. The ventral surface (fig. 3.2) looks exactly like the MNHN specimen (without the male parts outside, which were subsequently removed). The figure 3.3 illustrates a much larger individual which could not be located. No information was provided on sizes, except that the illustrations were of “natural length” (figures 3.1, 3.2) and “likely of natural length” (figure 3.3) according to Audouin (1826: 19). Given that it is unclear whether Savigny (unknowingly) illustrated one or two species, it is appropriate to designate the specimen preserved at the MNHN as the lectotype (MNHN-IM-2000-22941). The animals illustrated by Savigny (1817) were not accompanied by any species name, but they were named and described ten years later by Audouin (1826: 18–20) who referred to the figures 3.1–3.8 on Savigny’s plate 2 as *Onchidium peronii*. Interestingly, Audouin (1826: 19, our translation) wrote that this identification was suggested to him by Cuvier himself: “Mr. Cuvier, to whom we communicated Mr. Savigny’s drawing, believed he recognized Péron’s onchidie.” Cuvier likely changed his mind and later decided that, for some reason, the specimens illustrated by Savigny were a distinct species he called *Onchidium verruculatum*. The lectotype is still well preserved, considering how old it is. The radula and the posterior (female) reproductive parts are still inside but only the deferens duct remains for the male copulatory parts. Its intestinal loops are of type I (Fig. 86A). The number of papillae with eyes can hardly be counted on the dorsal notum of the lectotype because it has faded (only four papillae with eyes were counted).

***Lectotype* and *paralectotypes*** (*Onchidium ferrugineum*). Indonesia • lectotype, 35/25 mm; havre de Doréry [spelling mistake for Dorey], à la Nouvelle-Guinée [now Manokwari harbor, West Papua]; MNHN-IM-2000-22951. • 2 paralectotypes, 33/20 and 26/18 mm; same collection data as for the lectotype; MNHN-IM-2000-22951. The lectotype was designated by Goulding et al. (2018: 75) to clarify the application of *Onchidium ferrugineum*. The two paralectotypes belong to *Wallaconchis ater* (Lesson, 1831a) because they lack dorsal gills, lack an accessory penial gland, and are characterized by a highly coiled penis. Labbé re-examined four specimens from the original type series but there are only three specimens left in the jar, so one specimen was lost by or after Labbé. The lectotype is well preserved. Its dorsal notum bears obvious gills. Its male opening is located below and to the left of the right ocular tentacle. Pieces of the deferent duct and of the flagellum of the accessory penial gland remain, but the muscular sac and the spine of the accessory gland are missing. The posterior (female) part of the reproductive system is still in place inside the lectotype. Its radula is missing. Its intestinal loops of type I (with a transitional loop at 4 o’clock) are illustrated here (Fig. 80A).

***Lectotype*** (*Peronia savignii*). Red Sea • lectotype, hereby designated, 30/26 mm; MNHN-IM-2000-22941. Récluz (1869: 61) created the species name *Peronia savignii* with a reference to Savigny’s (1817) illustrations as indication (“Descr. de l’Egypte, pl. II, f. 1–5”). There are only three figures on Savigny’s (1817) plate 2. Obviously, Récluz did not mean to refer to *Tritonia* (figs 1.1–1.12) or *Bursatella* (figs 2.1–2.13) but only to *Peronia* (figs 3.1–3.8). There is no easy explanation for the exclusion of figures 3.6–3.8 except that Audouin’s (1826) captions for Savigny’s (1817) figures 3.1–3.5 are on page 19 and those for figures 3.6–3.8 are on page 20. At any rate, *Peronia savignii* is available and the type series consists of the individuals illustrated by Savigny. The lectotype of *Onchidium verruculatum* (MNHN-IM-2000-22941) is logically part of the type series of *P. savignii* (in fact, a label already indicates that it is a syntype of *P. savignii*). In order to clarify the application of *P. savignii*, the lectotype of *O. verruculatum* is also designated as the lectotype of *P. savignii*, and *P. savignii* remains what it has always been, a junior objective synonym of *O. verruculatum*.

***Syntypes*** (*Onchidium branchiferum*). Philippines • 2 syntypes, 27/18 and 24/15 mm; Cavite, Manila [Luzon]; ZMB/Moll 11614. Both syntypes were completely dissected prior to the present study, likely by Plate himself, and all internal organs are either missing or destroyed. The type of intestinal loops could not be verified. Dorsal gills are present on the notum. The type series also includes six histological slides.

***Holotype*** (*Onchidium elberti*). Indonesia • holotype, by monotypy, 24/20 mm; Südost-Celebes, Moena, Raha [now Raha, Muna Island, Sulawesi]; SMF 45248. The holotype was never dissected prior to the present study. The animal is more or less hemispherical. It was carefully opened dorsally to check and illustrate its intestinal loops of type I (Fig. 80B). Dorsal gills are present on the notum.

***Holotype*** (*Onchidium astridae*). Indonesia • holotype, by monotypy, 20/18 mm; Sorong door, Nouvelle-Guinée [Sorong, West Papua]; RBINS I.G.9223/MT.3822. The holotype, clearly labeled as “*Oncidium Astridae* Labbé,” was dissected by Labbé for the original description but is relatively well preserved. The radula, the posterior (female) reproductive parts, and the intestinal loops of type I (Fig. 80C) are still in place inside the specimen. Male parts are missing. Dorsal gills are present on the notum (partly cut by Labbé). Note that the locality on the label of the holotype is indicated as Sorong, but with a question mark.

***Lectotype* and *paralectotypes*** (*Peronia gaimardi*). Solomon Islands • lectotype, hereby designated, 44/27 mm; Vanikoro; 1829; JRC Quoy & JP Gaimard leg.; MNHN-IM-2000-33705. • 1 paralectotype, 35/30 mm; same collection data as for the lectotype; MNHN-IM-2000-33705. The type material also includes a paralectotype from Djibouti which could not be located with certainty (see below). Originally, no jar clearly labeled as the type material of *Peronia gaimardi* was found at the MNHN. The original description of *P. gaimardi* is based on three individuals, two individuals identified as *Onchidium*, from “Vanikoro (Quoy and Gaimard 1829),” and one individual identified as “*OncidiumPeronii*,” from “Obock, Récif de Clochettins (Gravier 1904).”

The two specimens from Vanikoro were found at the MNHN in a jar with three labels. One old label says “Onchidium [subsequently replaced by Peronia] de Vanikoro, mm Quoy et Gaimard 1829.” Another label only says “44” for unknown reasons. And a more recent label says “Peronia Vanikoro, M. Quoy et Gaimard 1829.” There is no indication that those two specimens are part of the type series of *P. gaimardi*. However, there is only one jar of specimens collected by “Quoy et Gaimard 1829” from Vanikoro at the MNHN and, given that the size of the largest specimen (42/22 mm) provided by Labbé matches the size of the lectotype designated here, there is little doubt that those two individuals from Vanikoro were originally used by Labbé to describe *P. gaimardi*. The lectotype was dissected by Labbé. Its radula and male apparatus are missing. The female parts are still inside the animal. Its intestinal loops of type I are illustrated here (Fig. 80E). Dorsal gills are present on the notum. The paralectotype from Vanikoro was not dissected by Labbé.

As for the paralectotype from Obock, Djibouti, it could not be traced with certainty, which does not matter given that it has no name-bearing function. Based on the original description (Labbé 1934a: 194), the paralectotype from Djibouti was collected by Gravier in 1904 at the “Récif de Clochettins, Obock,” that it measured 80/57 mm, and that its body was “very flattened.” There is a jar at the MNHN with a label saying “OncidiumPeronii, Cuv. Obock M. Gravier 1904 – A Labbé, dét [for “déterminé,” i.e., identified] 1933.” Another label says “F” for unknown reasons. All the information on the label matches the information provided by Labbé in the original description of *P. gaimardi*, and the size (80/60 mm) of the specimen perfectly matches the size of the paralectotype of *P. gaimardi*. That specimen is just an empty notum with dorsal gills (all internal organs are missing). However, for two reasons, it is extremely unclear whether that specimen is the paralectotype of *P. gaimardi* from Obock. First, there is yet a third label (which was covered by the “Obock, Gravier, 1904” label) saying that the specimen was, instead, collected by Jousseaume from the Red Sea (“Mer Rouge”). And, second, Labbé (1934a: 192) listed a specimen from the same locality (“Récif de Clochettein [for Clochettins] (Obock)”), also collected by “Gravier 1904” and also identified as “*Onchidium peronii*” in his re-description of *Peronia tongana*. Therefore, given that there is only one jar at the MNHN with a specimen collected by Gravier in 1904 from Obock (there are other specimens from Obock at the MNHN, but not collected in 1904 by Gravier), and that the specimen may not even have been collected by Gravier, it is not possible to know whether that specimen is the paralectotype of *P. gaimardi*, a non-type material used by Labbé for a re-description of *Peronia tongana*, or even something completely different.

***Lectotype* and *paralectotype*** (*Peronia anomala*). Red Sea • lectotype, hereby designated, 10/8 mm; 1893; Jousseaume leg.; MNHN-IM-2000-33678. • 1 paralectotype, 6/3 mm; same collection data as for the lectotype; MNHN-IM-2000-33678. Originally, no jar clearly labeled as the type material of *Peronia anomala* was found at the MNHN, but it could be traced back. The original description of *P. anomala* is based on two individuals (10/9 and 5/5 mm) from the Red Sea (“Mer Rouge”) collected by Jousseaume in 1893. Several old jars were found at the MNHN with material collected from the Red Sea by Jousseaume. Most jars are labeled as “1892” for collecting date, one jar is labeled as “1893” (MNHN-IM-2000-33678), and another as “1823” (MNHN-IM-2000-33698). The jar with the (erroneous) collecting date of 1823 is the type series of *Onchidium durum* (see below). The jar with a collecting date of 1893 matches perfectly the information provided in Labbé’s original description of *P. anomala* and even the animal sizes match (MNHN-IM-2000-33678): these two specimens are considered to be the type series of *P. anomala*, and the largest specimen is designated as the lectotype. Both the lectotype and the paralectotype were dissected by Labbé. The radula and female and male reproductive parts of the lectotype are missing (the lack of penis and accessory penial gland, mentioned by Labbé, but likely due to the lectotype being not fully mature, cannot be checked). Dorsal gills are present on the notum. Its intestinal loops are not of type II (Labbé 1934a: 195), but of type I instead (Fig. 86B). The paralectotype is largely destroyed but bears dorsal gills on the notum.

***Lectotype* and *paralectotypes*** (*Paraperonia gondwanae*). India • lectotype, hereby designated, 29/25 mm; Bombay [Mumbai]; MNHN-IM-2000-33681. • 1 paralectotype, 26/25 mm; same collection data as for the lectotype; MNHN-IM-2000-33681. • 1 paralectotype, 50/35 mm; same collection data as for the lectotype; MNHN-IM-2000-33682. Red Sea • 4 paralectotypes, 40/30 mm; 1892; Jousseaume leg.; MNHN-IM-2000-33683. • 13 paralectotypes, 32/25 to 25/20 mm; Red Sea; 1892; Jousseaume leg.; MNHN-IM-2000-33688. • 15 paralectotypes, 40/30 to 22/20 mm; Suez [Egypt, Red Sea]; 1878; Letourneux leg.; MNHN-IM-2000-33684. The type material mentioned in the original description also includes a paralectotype from Mauritius which could not be located with certainty at the MNHN, a paralectotype from the Red Sea which could not be located at the MNHN, and another individual missing from one of the jars from the Red Sea (see below). Most importantly, the type specimens belong to more than one species, so a lectotype is designated to clarify the application of the name *P. gondwanae*.

Originally, no jar clearly labeled as the type material of *P. gondwanae* was found at the MNHN, but most of the type material could be traced back. The original description of *P. gondwanae* is based on 38 individuals which Labbé, as often, listed in his article using italicized letters: *a*) three individuals from Bombay and one individual from the Red Sea (“mer Rouge”), for which Labbé gives the sizes 29/23 and 50/30 mm; *b*) one individual (60/50 mm) from Mauritius (“île de France”) collected by Mathieu; *c*) five individuals (40/27 mm) from the Red Sea (“mer Rouge”) collected by Jousseaume in 1892; *d*) 15 individuals from Suez (Red Sea) collected by Letourneur in 1878; and *e*) 13 individuals from the Red Sea (“mer Rouge”) collected by Jousseaume in 1892, for which Labbé gives the size 32/25 mm (for both *d* and *e*).

The specific name “gondwanae” was written in pencil only on two old jars at the MNHN. One jar contains four of the five “*c*” individuals collected from the Red Sea by Jousseaume in 1892 (MNHN-IM-2000-33683); the name “*gondwanae*” is written on the small label with the number “59;” the size of the four specimens (40/30 mm) matches the size provided by Labbé. Another jar contains the 13 “*e*” individuals collected from the Red Sea by Jousseaume in 1892 (MNHN-IM-2000-33688); this jar was found only labeled as “57 gondwanae,” i.e., with no locality, collector name, or collecting year, but the number of individuals and their size (32/25 to 25/20 mm) matches the size provided by Labbé (35/25 mm).

No other jar labeled as *P. gondwanae* was found at the MNHN, but most of the remaining type material could be traced back thanks to the matching of collector’s name, collecting date, specimen sizes, and the number of old jars from any given locality at the MNHN. There are only three old jars with specimens from Bombay at the MNHN. One jar contains seven *Platevindex* individuals collected by Roux in 1826. The two other jars contain the three “*a*” individuals from Bombay: one jar contains two individuals (29/25 and 26/25 mm) (MNHN-IM-2000-33681) and the other jar contains one individual (50/35 mm) (MNHN-IM-2000-33682), which sufficiently matches the sizes in Labbé’s original description (50/30 and 29/23 mm). There is only one old jar at the MNHN with 15 specimens (from 40/30 to 22/20 mm) from Suez collected by Letourneux (“Letourneur” in the original description) in 1878 (there is another old jar of *Peronia* from Suez but collected by Jousseaume in 1889). That jar contains the “*d*” individuals of *P. gondwanae* from Suez (MNHN-IM-2000-33684). The “*b*” individual from Mauritius could not be traced with certainty at the MNHN. Indeed, there are two jars, each with a single specimen from Mauritius collected by Mathieu and identified as *OncidiumPeronii* by Labbé in 1933: one 65/40 specimen (MNHN-IM-2000-33687), and one 60/40 specimen (MNHN-IM-2000-33686). Both specimens match the size provided by Labbé for the “*b*” individual (60/50 mm). Labbé (1934a) listed only once a specimen from Mauritius by Mathieu in his entire work, and that specimen could be the one in either jar (i.e., MNHN-IM-2000-33686 or MNHN-IM-2000-33687). Finally, the “*a*” individual identified from the Red Sea could not be located.

The 29 mm long “*a*” individual from Bombay, dissected by Labbé, is designated here as the lectotype of *Paraperonia gondwanae* (MNHN-IM-2000-33681). Its radula and male parts are missing. Its intestinal loops are clearly of type I (Fig. 84A) even though Labbé described loops of type V. The 50 mm long “*a*” individual from Bombay was also dissected by Labbé (MNHN-IM-2000-33682). Its radula and male parts are missing but its intestinal loops are of type V (Fig. 21B), as in the original description, so it does not belong to *P. verruculata* but *P. madagascariensis* instead. Labbé dissected only two of the 15 specimens from Suez (MNHN-IM-2000-33684): the radula and the male parts are missing from both specimens (38/32 and 35/28 mm) but their intestinal loops are both of type I (Fig. 86D), suggesting that they belong to *P. verruculata*, even though Labbé described loops of type V. Labbé dissected only one (40/30 mm) of the four specimens from Suez (MNHN-IM-2000-33683), acknowledging that maybe one specimen was lost: the radula and the male parts are missing, but its intestinal loops are of type V (Fig. 21C), as in the original description, suggesting that it belongs to *P. madagascariensis*. Labbé dissected seven of the 13 specimens (assumed to be) from the Red Sea (MNHN-IM-2000-33688). Those specimens are all completely destroyed and extremely poorly preserved. An undissected individual (35/25 mm) from the same lot was dissected for the present study and its intestinal loops are of type I, suggesting that it belongs to *P. verruculata* (Fig. 86E). Finally, according to Labbé, the intestinal loops of the specimen from Mauritius (collected by Mathieu) are of type V. One specimen collected by Mathieu from Mauritius is completely empty inside (MNHN-IM-2000-33687). The loops of the other specimen are of type I (Fig. 9D), suggesting that it belongs to *P. peronii* (MNHN-IM-2000-33686).

***Lectotype* and *paralectotypes*** (*Scaphis viridis*). Australia • lectotype, hereby designated, 50/20 mm; Thursday (Océanie) [Thursday Island, Torres Strait]; 1892; Lix leg.; MNHN-IM-2000-22964. • 2 paralectotypes, 45/30 mm and 45/25 mm; same collection data as for the lectotype; MNHN-IM-2000-22964. Originally, no jar clearly labeled as the type material of *Scaphis viridis* was found at the MNHN. However, only one old jar was found at the MNHN with specimens collected from Thursday Island, and the collecting information on the label (specimens collected by M. Lix in 1892) matches the information provided in Labbé’s original description of *S. viridis* (even though, according to Labbé, the specimens were collected in 1890). The sizes provided by Labbé (48/20, 47/30, and 42/25 mm) match the sizes of the three specimens here and their notum clearly bears dorsal gills, as in the original description of *S. viridis*. Labbé mentioned four specimens but, given that he provided measurements for only three specimens, it is possible that he only examined three specimens. Or he examined four specimens and one is now missing. The three type specimens are largely destroyed inside (due to Labbé’s dissections). The male parts and radula are missing in both paralectotypes but are still inside the lectotype. The intestinal loops of the lectotype are of type I, with a transitional loop at 5 o’clock (Fig. 80F). The three types are green (hence the specific name chosen by Labbé) but that color is clearly due to preservation.

***Holotype*** (*Scaphis carbonaria*). New Caledonia • holotype, by monotypy, 40/26 mm; 1880; Réveillère leg.; MNHN-IM-2000-33708. Originally, no jar clearly labeled as the type material of *Scaphis carbonaria* was found at the MNHN. However, of the several old jars found at the MNHN with specimens collected from New Caledonia, only one matches perfectly the information provided in Labbé’s original description of *S. carbonaria*: an individual collected in 1880 by M. Réveillère (the French navy officer Paul Réveillère [1829–1905]) with an identification as *Peronia*. Other jars with specimens from New Caledonia were collected by Fisher in 1878 or by François in 1894. Therefore, it is extremely likely that the specimen collected by Réveillère in 1880 and identified as “*Peronia*” is the holotype, by monotypy, of *Scaphis carbonaria*. The size of the holotype (40/26 mm) matches the size provided by Labbé in the original description of *S. carbonaria* (36/25 mm). Its notum is not well preserved. Dorsal papillae are quite flattened (as pointed out by Labbé) and dorsal eyes cannot be seen, likely because their black color faded. However, dorsal gills are clearly present on the notum. Its intestinal loops are of type I (Fig. 80D) but its radula is missing. The posterior (female) reproductive parts are still present but poorly preserved. The copulatory parts are missing, except for the muscular sac of the accessory penial gland (approximately 10 mm long) and so the length of the spine of the accessory penial gland cannot be checked (it was not mentioned by Labbé in the original description).

***Lectotype* and *paralectotypes*** (*Scaphis gravieri*). Mayotte • lectotype, hereby designated, 27/18 mm; 1883; A Vimont leg.; MNHN-IM-2000-33695. Zanzibar • 4 paralectotypes, 30/28, 32/25, 27/23, and 14/10 mm; 1865; Grandidier leg.; MNHN-IM-2000-33693. The type material mentioned in the original description also includes two paralectotypes from Djibouti which could not be located with certainty at the MNHN (see below). Originally, no jar clearly labeled as the type material of *S. gravieri* was found at the MNHN, but most type material could be traced back.

The original description of *S. gravieri* is based on seven individuals: two individuals (10/7.5 and 8/6.5 mm) from Djibouti collected by Gravier in 1904; four individuals (32/29 and 30/25 mm) from Zanzibar collected by Grandidier (the French naturalist and explorer Alfred Gandidier [1836–1921]) in 1865; and one individual (28/19 mm) from Mayotte collected by Ach. Vimont in 1883.

One old jar was found at the MNHN with a specimen from Mayotte (MNHN-IM-2000-33695). The information on the label (specimen collected from Mayotte by Vimont in 1883) matches the information provided in Labbé’s original description of *S. gravieri*, and the specimen size also matches. Therefore, that specimen from Mayotte is here considered to form part of the type series of *S. gravieri* and designated as the lectotype (MNHN-IM-2000-33695). This lectotype was dissected by Labbé: the radula and the posterior (hermaphroditic) reproductive parts are still in place but the male parts are missing. The intestinal loops are of type I with a transitional loop at 6 o’clock (Fig. 85A).

Another old jar was found at the MNHN with specimens from Zanzibar (MNHN-IM-2000-33693). The information on the label (specimens collected from Zanzibar by Grandidier in 1865) matches the information provided in Labbé’s original description of *S. gravieri*, and the specimen size also matches (Labbé likely provided the size of the largest two specimens). Therefore, those four specimens from Zanzibar are considered to form part of the type series of *S. gravieri* and are now paralectotypes (MNHN-IM-2000-33693). Only one paralectotype (30/28 mm) from Zanzibar was dissected by Labbé: the radula and the posterior (female) reproductive parts are still in place but the male parts are missing. The intestinal loops are of type I.

The two paralectotypes from Djibouti could not be traced with certainty. There are two old jars of specimens collected by Gravier in 1904 at the MNHN. One jar is labeled with Obock as locality (not Djibouti, even though Obock is in Djibouti) and contains one *Peronia* specimen of which the size (80/60 mm) does not match Labbé’s original description of *S. gravieri*. Also, that specimen from Obock is more likely to be a paralectotype of *P. gaimardi* or a non-type specimen used by Labbé for the re-description of *Peronia tongana*. The three specimens (70/60, 70/65, and 65/65 mm) of the second jar collected by Gravier in 1904 are from Djibouti (MNHN-IM-2000-33696), which matches perfectly the original description of *S. gravieri* by Labbé. The problem is that the specimen sizes do not match because Labbé described two individuals of only 10/7.5 and 8/6.5 mm. It is likely that Labbé meant centimeters instead of millimeters (even though he wrote “mm”) because he described a muscular sac of 8 mm in the specimens from Djibouti, which is impossible in individuals that are only 8 and 10 mm long. One of three specimens, possibly dissected by Labbé, possibly is part of the type series of *S. gravieri*, but it remains questionable. In addition, a specific name was added in pencil on an old label with the number “69” but that name, which is impossible to read, seems to start with a J, and not a G. In summary, it remains unclear whether those three specimens from Djibouti can be regarded as part of the type series of *S. gravieri*; however, it ultimately does not matter because a lectotype is designated here.

***Syntypes*** (*Scaphis tonkinensis*). The type material of *Scaphis tonkinensis* (ten syntypes up to 20/18 mm, according to the original description) could not be located with certainty at the MNHN. Only one old jar was found at the MNHN (MNHN-IM-2000-33700) with specimens collected from Vietnam (as “Tonkin”), and the information on the label (material collected by M. Julien in 1874) matches the information provided in Labbé’s original description of *S. tonkinensis*. Therefore, it is possible that the jar mentioned here contains the type material of *S. tonkinensis*. Unfortunately, the jar only contains three pieces of unidentifiable and poorly preserved tissue (each piece measuring approximately 20/10 mm). Two pieces are likely not even part of an onchidiid slug, and it is unclear whether the third piece is part of an onchidiid dorsal notum or not. So, regardless of whether this material is regarded as part of the type material of *S. tonkinensis*, it is basically useless.

***Syntypes*** (*Scaphis lata*). The type material of *Scaphis lata* (four syntypes up to 28/28 mm, from Vietnam) could not be located at the MNHN. Only one old jar was found at the MNHN (MNHN-IM-2000-33700) with specimens collected from Vietnam (as “Tonkin”), but the information on the label (specimens collected by M. Julien, in 1874) does not match exactly the information provided in Labbé’s original description of *S. lata* (specimens collected by M. Julien in 1878), and, instead, matches the information provided in Labbé’s original description of *S. tonkinensis* (see above).

***Lectotype* and *paralectotypes*** (*Onchidium durum*). Red Sea • lectotype, hereby designated, 20/15 mm; 1893; Jousseaume leg.; MNHN-IM-2000-33698. • 25 paralectotypes, from 23/15 to 14/14 mm; same collection data as for the lectotype; MNHN-IM-2000-33698. Originally, no jar clearly labeled as the type material of *Onchidium durum* was found at the MNHN, but it could be traced back. The original description of *O. durum* is based on “approximately” 20 individuals (from 24/23 to 14/13 mm) from the Red Sea (“Mer Rouge”) collected by Jousseaume in 1893.

Several old jars were found at the MNHN with material collected from the Red Sea by Jousseaume. Most jars are labeled with 1892 as collecting date, one jar is labeled with 1893, and another with 1823. The jar with a collecting date of 1893 (MNHN-IM-2000-33678) contains the type series of *P. anomala* (see above). On the jar with the collecting date of 1823, there is another tiny label with the number “61” (for an unknown numbering system) on which *O. durum* is clearly written in pencil. It is one of the very few cases in which a species name is indicated for some MNHN material studied by Labbé and there is little doubt that the specimens are the type series of *O. durum*, especially because the number of individuals and their sizes perfectly match with Labbé’s original description. Clearly, 1823 is a mistake for 1893. Most importantly, contrary to what was described by Labbé, gills are present on the dorsal notum of those individuals. All specimens are poorly preserved. They likely dried at some point and their body is hard. Three specimens were opened by Labbé and are now largely destroyed with only the digestive system inside. A lectotype is designated here in order to clarify the application of *O. durum* (specimens in the type series could belong to more than one species). Its intestinal loops are not of type II (Labbé 1934a: 221): they clearly are of type I (Fig. 86C).

***Holotype* and *paratypes*** (*Peronia persiae*). Iran – **Persian Gulf** • holotype [not examined], by original designation, 35 mm; Lavan Island; 26°48.3498'N, 53°16.08'E; Feb 2016; ZSM Mol 20180017. • 2 paratypes [not examined], 22 and 37 mm; same collection data as for the holotype; ZSM Mol 20180018. • 1 paratype [not examined], 32 mm; Bandar Lengeh; 26°33.4833'N, 54°52.8333'E; Mar 2015; ZSM Mol 20180018.

The original description of *P. persiae* is based on a total of 14 individuals (from 13 to 37 mm): the four types (see above) and ten other specimens from the same two localities as the types. DNA sequences (COI and 16S) are provided for 11 of those 14 individuals, including all four type specimens. However, it is unclear which GenBank sequences correspond exactly to the holotype because this information is missing in GenBank as well as in Maniei et al. (2020a: table 2). It is assumed that the holotype, called “specimen LA7” in Maniei et al. (2020a: table 1), corresponds to the individual called “voucher LaFM7S” in GenBank. Ultimately, it does not matter at all because all mitochondrial sequences of *P. persiae* cluster together within the unit #4 of *P. verruculata*: only the COI (MK993404) and the 16S (MK993392) sequences of the “voucher LaFM7S” are included in our phylogenetic analyses to represent *P. persiae* (Fig. 2). Finally, note that the COI and 16S GenBank accession numbers are switched for *P. persiae* in Maniei et al. (2020a: table 2). Comments on the original description of *P. persiae* are provided in the species remarks (see below).

###### Additional material examined

**(unit #1).** Australia – **Queensland** • 1 specimen 35/25 mm [2682]; Mackay, Campwin Beach; 21°22.455'S, 149°18.753'E; 5 Jul 2013; TC Goulding and field party leg.; st 121, by boat ramp, mangrove margin with large rocks by creek, *Rhizophora* and soft mud; MTQ. • 1 specimen 40/25 mm [2620]; Bowen, Dingo Beach; 20°04.864'S, 148°29.576'E; 30 Jun 2013; TC Goulding and field party leg.; st 113, rocky shore nearby a small and dense *Rhizophora* mangrove patch; MTQ. • 1 specimen 25/15 mm [2622]; same collection data as for the preceding; MTQ. 1 specimen 22/18 mm [1538]; Magnetic Island, near Cockle Bay, off Townsville; 19°10.500'S, 146°49.552'E; 20 Sep 2005; I. Loch leg.; on top of dead coral on fringing reef and muddy sand flats with seagrasses; AM C.448363. • 1 specimen 25/20 mm [2571]; Cairns, Keewara Beach; 16°34.711'S, 145°30.751'E; 18 Jun 2013; TC Goulding and field party leg.; st 102, rocky platform; MTQ.

Indonesia – **Ambon** • 2 specimens 35/25 mm [2724] and 40/30 mm [2729]; Pulau Haruku; 03°36.31'S, 128°25.04'E; 11 Feb 2014; st 127; M Khalil and field party leg.; rocky *Sonneratia* mangrove with coral rubble; UMIZ 00162. • 1 specimen 45/30 mm [2856]; Wai; 03°34.65'S, 128°19.53'E; 15 Feb 2014; M Khalil and field party leg.; st 132, narrow band of old *Avicennia* trees on sandy mud, old logs on ground; UMIZ 00163. – **Bali** • 1 specimen 20/15 mm [3080]; Gilimanuk; 08°10.26'S, 114°26.61'E; 3 Apr 2014; M Khalil and field party leg.; st 155, large rocks near a patch of *Rhizophora*; UMIZ 00164. • 1 specimen 20/12 mm [3115]; Gilimanuk; 08°10.16'S, 114°26.65'E; 4 Apr 2014; M Khalil and field party leg.; st 156, sandy mudflat outside *Rhizophora* and *Avicennia* mangrove; UMIZ 00165. – **Halmahera** • 1 specimen 45/35 mm [5068]; Sofifi; 00°45.47'N, 127°35.90'E; 8 Mar 2015; st 205, *Sonneratia* mangrove; UMIZ 00166. • 3 specimens 40/25 mm [5120], 50/35 mm [5124], and 35/25 mm [5130]; Folly; 01°14.66'N, 128°10.61'E; 19 Mar 2015; M Khalil and field party leg.; st 217, rocky shore near a beach; UMIZ 00167. – **Lombok** • 1 specimen 40/25 mm [2987]; Don Don; 08°54.54'S, 116°21.50'E; 26 Mar 2014; M Khalil and field party leg.; st 149, old *Avicennia* forest with coral rubble; UMIZ 00168. – **Seram** • 3 specimens 50/40 mm [2868], 50/35 mm [2870], and 55/40 mm [3441]; 02°58.24'S, 128°07.07'E; 18 Feb 2014; M Khalil and field party leg.; st 135, mud next to a mangrove; UMIZ 00169. – **Sulawesi** • 1 specimen 45/25 mm [2127]; North Sulawesi, Wori; 01°36.06'N, 124°51.73'E; 9 Mar 2013; M Khalil and field party leg.; st 84, old *Sonneratia* and *Avicennia* mangrove; UMIZ 00170. • 2 specimens 25/20 mm [2150] and 60/45 mm [2162]; North Sulawesi, Bahoi; 01°43.36'N, 125°01.23'E; 10 Mar 2013; M Khalil and field party leg.; st 85, sand and small rocks outside a mangrove; UMIZ 00171. • 1 specimen 23/18 mm [731]; South East Sulawesi, Walowa, Pasarwajo Bay, Buton Island; 28 Oct 2005; MAE Malaquias leg.; upper tidal, on rock pools; NHMUK 20050628. – **Sumatra** • 1 specimen 40/30 mm [1747]; Lampung, Penegahan; 05°40.40'S, 105°33.76'E; 18 Oct 2012; M Khalil and field party leg.; st 78, coral rubble on beach exposed to estuary; UMIZ 00172. • 1 specimen 20/15 mm [1759]; Lampung, near Kalianda, Sungai Boluk; 05°40.793'S, 105°33.625'E; 23 Oct 2012; M Khalil and field party leg.; st 82, beach with a few rocks; UMIZ 00173. – **Timor** • 1 specimen 45/25 mm [5904]; Oesapa; 10°08.73'S, 123°38.10'E; 11 Jul 2016; M Khalil and field party leg.; st 250, sandy part of mangrove, with *Sonneratia* and *Avicennia* trees; UMIZ 00174. • 2 specimens 12/7 mm [5925] and 35/20 mm [5927]; Kelapa Lima; 10°08.715'S, 123°36.914'E; 13 Jul 2016; M Khalil and field party leg.; st 252, rocky area at sandy beach with algae; UMIZ 00175.

Japan • 2 specimens 40/30 mm [3752] and 32/25 mm [3751]; Honshu, Wakayama, Nishimuro, near the Seto Marine Biological Laboratory; 33°41.533'N, 135°20.265'E; 2014; T. Nakano leg.; NSMT-Mo 78988.

New Caledonia • 1 specimen 50/45 mm [6202]; Baie de Taaré; 22°15.286'S, 167°00.808'E; 19 Sep 2018; Our Planet Reviewed Koumac 2018 expedition leg.; st KM524, intertidal sandy coral rubble flat in front of mangroves; MNHN-IM-2019-1591. • 1 specimen 73/52 mm [6212]; Tontouta, South side of Page Island (Ubeakure); 22°03.443'S, 166°05.080'E; 25 Sep 2018; Our Planet Reviewed Koumac 2018 expedition leg.; st KM537, coastal rocky mangrove; MNHN-IM-2019-1592. • 1 specimen 43/30 mm [6214]; Pointe Sauveur, Presqu’île de Quano; 21°52.006'S, 165°49.195'E; 26 Sep 2018; Our Planet Reviewed Koumac 2018 expedition leg.; st KM538, muddy intertidal rocky flat in front of mangroves; MNHN-IM-2019-1593.

Palau • 1 specimen 35/30 mm [698]; Ngerchaol Island, East end, South shore, North of quarry on Malakal Island; 07°20.433'N, 134°27.150'E; 15 Feb 1995; K Auffenberg leg.; UF 253871.

Papua New Guinea – **Madang** • 1 specimen 35/30 mm [5467]; Rempi Area, south Dumduman Island; 05°00.2'S, 145°47.6'E; 9 Nov 2012; MNHN Expedition Papua Niugini leg.; st PM 12, limestone rocky intertidal; MNHN-IM-2013-12008. • 1 specimen 38/30 mm [5468]; same collection data as for the preceding; MNHN-IM-2013-12009. • 1 specimen 35/30 mm [5469]; same collection data as for the preceding; MNHN-IM-2013-12010. – **New Ireland** • 1 specimen 40/30 mm [6085]; Kavieng, west side of Nago Island; 02°36.3'S, 150°46'E; 6, 9, 10, 14 & 22 Jun 2014; MNHN Expedition Kavieng 2014 leg.; st KM 01; MNHN-IM-2013-50974. • 1 specimen 25/25 mm [6087]; Kavieng, Povalval, East coast of New Ireland; 02°41'S, 150°57'E; 11 & 13 Jun 2014; MNHN Expedition Kavieng 2014 leg.; st KM 05, mixed hard platform and seagrass bed at outlet of rivulet; MNHN-IM-2013-53523. • 1 specimen 30/30 mm [6088]; same collection data as for the preceding; MNHN-IM-2013-53525.

Philippines – **Bohol** • 2 specimens 40/25 mm [3379] and 35/30 mm [3380]; Maribojoc; 09°44.02'N, 123°47.45'E; 19 Jul 2014; B Dayrat and field party leg.; st 200, coral rubble with sand, at night; PNM 041274. • 2 specimens 30/20mm [3433] and 35/25 mm [3437]; Maribojoc; 09°44.28'N, 123°49.39'E; 20 Jul 2014; B Dayrat and field party leg.; st 202, coral rubble with sand and algae, near *Sonneratia*; PNM 041276. – **Cebu** • 1 specimen 22/15 mm [712]; Badian near Barila, across road from entrance to Children Spring and Kawasan Falls, behind huts; 27 Apr 2005; KNRL-012 leg.; fringing reef flat, 0–2 feet reef walk; UF 368518. – **Luzon** • 2 specimens 50/35 mm [3160] and 40/25 mm [3161]; Batangas, Lian; 13°59.76'N, 120°37.43'E; 5 Jul 2014; B Dayrat and field party leg.; st 181, sandy, open *Avicennia* forest, right by the shore; PNM 041277. – **Negros** • 1 specimen 15/8 mm [704]; San Jose, near Sibulan; 28 Apr 2005; KNRL-011 leg.; exposed rocky intertidal, under and between rocks; UF 368517.

Singapore • 1 specimen 20/15 mm [991]; Pasir Ris Park; 01°22.840'N, 103° 57.224'E; 1 Apr 2010; B Dayrat and SK Tan leg.; st 5, mangrove forest with rich litter, lobster mounds, dead logs, with sand area near the creek; ZRC.MOL.10497.

Vanuatu • 1 specimen 20/10 mm [5480]; Port Vila; ca. 2008; MNHN leg.; MNHN-IM-2013-62392. • 1 specimen 17/11 mm [5481]; same collection data as for the preceding; MNHN-IM-2013-62393.

Vietnam • 2 specimens 60/40 mm [5620] and 40/30 mm [5621]; Hòn Tre Island; 12°11.983'N, 109°18.093'E; 28 Jul 2015; TC Goulding and field party leg.; st 238, coral rubble near small *Rhizophora* sandy and muddy mangrove; ITBZC IM 00021. • 1 specimen 25/20 mm [5670]; Côn Đảo Islands; 08°38.803'N, 106°34.719'E; 22 Jul 2015; TC Goulding and field party leg.; st 235, edge of dense *Rhizophora* mangrove, near sand; ITBZC IM 00022. • 1 specimen 17/14 mm [5639]; Côn Đảo Islands; 08°38.780'N, 106°33.210'E; 23 Jul 2015; TC Goulding and field party leg.; st 236, mangrove patch with many big flat rocks outside; ITBZC IM 00023.

###### Additional material examined

**(unit #2).** India • 1 specimen 25/15 mm [1072]; South Andaman, Burman Nala; 11°33.226'N, 92°43.997'E; 8 Jan 2011; B Dayrat and field party leg.; st 53, rocky shore with a patch of *Rhizophora*, sand and coral rubble but no mud; BNHS 1072. • 1 specimen 10/8 mm [1077]; South Andaman, Corbyn’s Cove; 11°38.676'N, 92°45.005'E; 9 Jan 2011; B Dayrat and field party leg.; st 54, rocky shore only, no mangrove; BNHS 119. • 1 specimen 15/12 mm [1079]; same collection data as for the preceding; BNHS 120. • 1 specimen 30/20 mm [1080]; same collection data as for the preceding; BNHS 121. • 1 specimen 20/15 mm [1081]; same collection data as for the preceding; BNHS 122. • 1 specimen 20/10 mm [1084]; South Andaman, Wandoor; 11°37.140'N, 92°37.242'E; 9 Jan 2011; B Dayrat and field party leg.; st 55, sandy beach with coral rubble; BNHS 117.

Indonesia • 1 specimen 50/35 mm [1746]; Sumatra, Lampung, Penegahan; 05°40.40'S, 105°33.76'E; 18 Oct 2012; M Khalil and field party leg.; st 78, coral rubble on beach exposed to estuary; UMIZ 00178. • 2 specimens 25/20 mm [1741] and 30/22 mm [1742]; same collection data as for the preceding; UMIZ 00179. • 3 specimens 55/35 mm [1796], 50/30 mm [1797], and 45/30 mm [1795]; Sumatra, Lampung, near Kalianda, Sungai Boluk; 05°40.793'S, 105°33.625'E; 23 Oct 2012; M Khalil and field party leg.; st 82, beach with a few rocks; UMIZ 00180.

###### Additional material examined

**(unit #3).** Peninsular Malaysia • 1 specimen 35/25 mm [976]; Langkawi; 06°25.361'N, 99°47.269'E; 14 Jul 2011; B Dayrat and field party leg.; st 25, large boulders on sand beach; USMMC 00051. • 3 specimens 35/30 mm [974], 27/20 mm [975], and 30/20 mm [977]; same collection data as for the preceding; USMMC 00064. • 2 specimens 25/20 mm [2546] and 40/25 mm [2547]; Penang, Pasir Panjang, Pulau Betong; 05°17.967'N, 100°11.080'E; 2013; SH Tan leg.; boulders and rocks on a beach; USMMC 00065.

Singapore • 1 specimen 25/20 mm [990]; East Coast Park; 01°18.259'N, 103°55.644'E; 29 Mar 2010; B Dayrat leg.; st 3, rocks of artificial breakwaters; ZRC.MOL.10496. • 1 specimen 12/8 mm [989]; East Coast Park; 01°18.153'N, 103°55.289'E; 29 Mar 2010; B Dayrat leg.; st 2, rocky shore covered by oyster flats; ZRC.MOL.16070.

###### Additional material examined

**(unit #4).** India • 1 specimen 55/30 mm [1141]; Mumbai, Bandstand, Bandra; 19°02.863'N, 72°49.174'E; 18 Dec 2011; TC Goulding and field party leg.; st 44, solid rock area, some crevices, near wastewater discharge to ocean; BNHS 22. • 1 specimen 60/40 mm [1143]; same collection data as for the preceding; BNHS 24. • 1 specimen 60/40 mm [1144]; same collection data as for the preceding; BNHS 23.

Pakistan • 1 specimen 50/40 mm [6164]; Sindh Province, Balochistan coast, near Karachi city, Hab River Delta; 24°53.22'N, 66°42.30'E; Apr 2017; S Aslam leg.; on oyster beds; MNHN-IM-2019-1384. • 1 specimen 50/35 mm [6165]; same collection data as for the preceding; MNHN-IM-2019-1385. • 1 specimen 40/25 mm [6166]; same collection data as for the preceding; MNHN-IM-2019-1386.

###### Additional material examined

**(unit #5).** Madagascar • 5 specimens 30/25 mm [3140], 40/30 mm [3231], 15/12 mm [3597], 30/20 mm [3142], and 13/8 mm [3598]; Antisiranana (Diego Suarez), Baie Andovobazaha; 12°18.887'S, 49°19.735'E; 16 May 2014; TC Goulding and field party leg.; st 158, rocky platform near *Avicennia* and *Rhizophora* mangrove; MNHN-IM-2019-1610. • 5 specimens 50/35 mm [3143], 35/25 mm [3144], 30/20 mm [3146], 30/20 mm [3600], and 30/20 mm [3149]; Ampondrahazo; 12°25.297'S, 49°28.916'E; 20 May 2014; TC Goulding and field party leg.; st 162, sandy mangrove of *Bruguiera*, on sandy mud in between rocks; MNHN-IM-2019-1611.

Mozambique • 1 specimen 22/18 mm [5507]; Baie de Maputo, Inhaca, Ponta Punduine; 26°02.5'S, 32°53.5'E; 24 Nov 2011; MNHN Expedition Inhaca 2011 leg.; st MM2, tide pools with sand and dead coral rubble; MNHN-IM-2013-62395. • 1 specimen 25/20 mm [5510]; same collection data as for the preceding; MNHN-IM-2013-62398. • 1 specimen 17/13 mm [730]; Cabo Delgado Province, Ibo Island; ca. 12°22'S, 40.35'E; 2 Jul 2006; DG Reid leg.; on mud in seaward *Sonneratia* zone of mangroves; NHMUK 20080190. • 1 specimen 15/14 mm [733]; Cabo Delgado Province, Ilha Lipulula; 1 km off Mocimboa da Praia; 11°20.65'S, 40°22.95'E; 8 Jul 2006; DG Reid leg.; on beach rock outcrops, upper eulittoral, moderately sheltered shore; NHMUK 20060257.

###### Additional material examined

**(Red Sea).** Egypt • 4 specimens 35/25 mm [#1], 40/30 mm [#2], 35/30 mm [#3], and 35/25 mm [#4]; Gulf of Suez, Gimsah Bay, African coast; Mar 1913; Bannwarth leg.; ZMH 27472/4. • 4 specimens from 40/30 mm to 30/25 mm; Suez; Bannwarth leg.; ZMH 27474/4.

Red Sea • 4 specimens from 35/25 mm to 25/22 mm; no precise locality data; Savigny, from the collections of the museum in Marseille, France, leg.; NHMD 90791.

###### Additional material examined

**(historical museum collections).** Australia • 1 specimen 30/23 mm; Queensland, Cape York; 1867; Salmin leg.; SMNH 180712. • 1 specimen 30/18 mm; Queensland, Cape York; 1 Jan 1881; Mac Leay leg.; SMNH 180713. • 1 specimen 28/23 mm; Queensland, Palm Island; 1 Jan 1881; Mac Leay leg.; SMNH 180714.

China • 1 specimen 40/28 mm [dissected prior to present study]; Hong Kong; 13 Oct 1878; Salmin leg.; SMNH 180707.

India • 11 specimens 35/28 to 20/15 mm; Nicobar Islands, Sambelong, N. V. Bugt [Great Nicobar, Sambelong, north-west bay (possibly the Ganges Harbor)]; 1 Feb 1846; Reinhardt, Galathea Expedition leg.; NHMD 635300.

Indonesia – **Java** • 1 specimen 33/25 mm; Batavia [Jakarta]; 06°07'S, 106°48'E; 1890; A Groth leg.; SMNH 180720. • 1 specimen 25/25 mm; Edam Island [Pulau Demar Besar, Jakarta Bay]; 1891; C Aurivillius leg.; SMNH 180719. • 1 specimen 30/28 mm; Insel Mendanao, westlich von Billiton [Mendanau Island, west of Belitung Island, Java Sea]; 20 Sept 1899; C Aurivillius leg.; SMNH 180722. – **Tanimbar** • 1 specimen 30/26 mm; Jamdena Straits, 2 miles north of Tg Nuan; 07°24'S, 131°19'E; 23 Jun 1970; Mariel King Memorial Expedition Moluccas MV “Pele” 1970 leg.; WAM S26630.

Iran • 14 specimens 80/60 to 25/15 mm; Persian Gulf coast, Bandar Bushehr; 28 Feb 1937; G Thorson leg.; tidevandszonen klippekyst [intertidal rocky shore]; NHMD 635301.

Madagascar • 1 specimen 35/32 mm; Catsepe [Katsepy]; 15°46'S, 46°14'E; 12 May 1912; W Kaudern leg.; SMNH 180724.

Pakistan • 1 specimen 23/20 mm; Karachi; 1884; O Dickson leg.; SMNH 180721.

Singapore • 1 specimen 30/25 mm [dissected prior to present study]; Singapore; 15 Jan 1853; Eugenie Expedition 1851–1853 leg.; st. 1502; SMNH 180716.

Tanzania • 3 specimens 35/25 to 15/14 mm; Zanzibar, Mafia Island, South Juani Island; 29 Jun 1994; M Richmond (from N Yonow’s personal collection) leg.; on film-covered rock at cliff base on exposed cliff to open ocean; MNHN-IM-2014-7989. • 4 specimens 50/35 to 35/25 mm; Zanzibar, Kisakasaka; Jun 1995; M. Richmond (from N Yonow’s personal collection) leg.; on rock outcrops in mangrove channel, very sheltered, in daytime; MNHN-IM-2014-7990.

###### GenBank sequences.

One COI sequence was obtained from GenBank (MH002601) for an individual identified as *Peronia* sp. and collected from Singapore (Chang et al. 2018). This individual as well as others were referred to as a “Singapore clade” by Chang et al. (2018) and clearly belong to the mitochondrial unit #3 of *Peronia verruculata* (Fig. 2). Another COI sequence was obtained from GenBank (MH002570) for an individual identified as *Peronia* sp. and collected from Singapore (Chang et al. 2018). This individual as well as others referred to as “*Peronia* sp. 2” by Chang et al. (2018), following Dayrat et al. (2011), clearly belong to the mitochondrial unit #1 of *Peronia verruculata* (Fig. 2). A third COI sequence was obtained from GenBank (LC390389) for an individual identified as *Peronia* sp. and collected from Sakurajima, Kagoshima, Japan (Tagaki et al. 2019), which is south to the northernmost known locality near the Seto Marine Biological Laboratory (see material examined). This individual as well as others from “Group V” were referred to as “*Peronia* sp.” by Takagi et al. (2019) and clearly belong to the mitochondrial unit #1 of *Peronia verruculata* (Fig. 2). Four COI sequences were obtained from GenBank (JN543152, JN543153, JN543154, JN543165) for individuals from the coast of China, from Hainan (18°N) to Fujian (26°N) (Sun et al. 2014). These individuals were referred to as “*Peronia verruculata*” by Sun et al. (2014) and clearly belong to the mitochondrial unit #1 of *Peronia verruculata* (Fig. 2). Finally, the COI (MK993404) and 16S (MK993392) sequences of the “voucher LaFM7S” represent *P. persiae* (Fig. 2): all published mitochondrial sequences of *P. persiae* cluster together within the unit #4 of *P. verruculata* so only one individual is needed to represent *P. persiae*.

###### Distribution

**(Fig. 6).***Peronia verruculata* is the most widespread of all onchidiid species. Its most western records are known from the Red Sea and southern Mozambique (26°S). Its most eastern records are in Japan, Wakayama (33°N), Vanuatu, and Queensland (21°S). It is unclear how far south it is distributed in southeastern Australia, although we did not find it in Sydney, New South Wales (see remarks below as well as remarks on *P. sydneyensis*). Undoubtedly, the delineation and distribution of the mitochondrial units of *P. verruculata* will change as new DNA sequences are added, especially from the Arabian Sea, the Red Sea, southern India, as well as southeastern Australia (see species remarks). Note that the range of *P. verruculata* is continuous. Even though our molecular analyses do not include specimens of *P. verruculata* from places like southern India, the Persian Gulf, or the northwestern corner of the Indian Ocean (coasts of Somalia, Yemen, and Oman), *P. verruculata* must be present there (red areas in Fig. 6). As of today, units #1 and #2 are sympatric in southeastern Sumatra (we found them both together at our stations 78 and 82), and units #1 and #3 are sympatric in Singapore.

*Peronia verruculata* also is very abundant and has been very often recorded in the past. However, *Peronia* species are externally cryptic and can be easily misidentified and confused. Here the records that are positively confirmed are distinguished from the records that cannot be confirmed. Erroneous applications of the name *P. verruculata* (or some of its synonyms) are also listed. All the details can be found in the species remarks (see below).

The presence of *P. verruculata* is confirmed here at the following locations (as *O. verruculatum* or *P. verruculata*, unless specified): Australia, Queensland (Hoffmann 1928; present study), Torres Strait (type locality of *S. viridis*; new record); China (Sun et al. 2014; Liu et al. 2015; Xu et al. 2018), Hong Kong (Hoffmann 1928; Britton 1984; present study); India, Andaman Islands (new record), Gulf of Mannar (Farran 1905), Nicobar (Mörch 1872a, b, as *P. mauritiana*; Semper 1880; Bergh 1884a; present study), western coast (type locality of *P. gondwanae*; new record); Indonesia, Ambon (new record), Bali (new record), Halmahera (new record), Java (Hoffmann 1928; present study), Lombok (new record), Seram (new record), Sulawesi (type locality of *O. elberti*; Dayrat et al. 2011, as *Peronia* sp. 6; new record), Sumatra (new record), Tanimbar (new record), Timor (new record), West Papua (type locality of *O. ferrugineum* and *O. astridae*; new record); Iran, Persian Gulf (Maniei et al. 2020a, type locality of *P. persiae*; present study); Japan, Kagoshima (Tagaki et al. 2019, as *Peronia* sp.; new record), Wakayama (new record); Madagascar (Odhner 1919; present study); Malaysia, Peninsular Malaysia (new record); Mayotte (type locality of *S. gravieri*; new record); Mozambique (Dayrat et al. 2011, as *Peronia* sp. 4 and 5; new record); New Caledonia (type locality of *S. carbonaria*; new record); Pakistan (Hoffmann 1928; present study); Palau (new record); Papua New Guinea, Madang (new record), New Ireland (new record); Philippines (Labbé 1934a, as *P. branchifera*; new record), Bohol (new record), Cebu (Dayrat et al. 2011, as *Scaphis* sp.; new record), Luzon (type locality of *O. branchiferum*; new record), Negros (new record); Red Sea (type locality of *O. verruculatum*, *P. savignii*, *P. anomala*, and *O. durum*; paralectotypes of *P. gondwanae*; present study); Singapore (Hoffmann 1928; Chang et al. 2018; present study); Solomon Islands (type locality of *P. gaimardi*; new record); Tanzania, Zanzibar (paralectotypes of *S. gravieri*; new record); Vanuatu (new record); Vietnam (type locality of *S. lata* and *S. tonkinensis*; new record).

The following records from the literature are not confirmed here, because authors did not provide enough information supporting the identification (as *O. verruculatum* or *P. verruculata* unless specified): Australia (Hutchings and Recher 1982), New South Wales (Bretnall 1919; Dakin 1947; Smith and Kershaw 1979); Queensland (Semper 1880; Hedley 1909; Bretnall 1919; Allan and Bell 1947; Allan 1950); Djibouti (Labbé 1934a, paralectotypes of *S. gravieri*); eastern Africa (Semper 1880); India, Andaman Islands (Santhosh Kumar et al. 2016), Gulf of Kutch (Menon et al. 1961), Gulf of Mannar (Gopinadha Pillai and Appukuttan 1980), Nicobar (Plate 1893), northwestern coast (Mandal and Harkantra 2013; Solanki et al. 2017), Sri Lanka (Nevill 1870, 1878; Plate 1893), southeastern coast (Hoffmann 1928), Uran City, near Mumbai (Patil and Kulkarni 2013); Indonesia, Ambon (Semper 1880; Plate 1893; Martens 1897), Timor (Martens 1897); Japan (Nakaoka et al. 2006; Wardiatno et al. 2015), Tokara Islands (Baba 1958), Misaki (Baba 1958), Boso Peninsula (Katagiri and Katagiri 2007); Madagascar (Marcus and Marcus 1970); Mauritius (Labbé 1934a), New Caledonia (Fischer and Crosse 1878; Hoffmann 1928; Labbé 1934a); New Guinea (Labbé 1934a); Philippines (Semper 1880; Labbé 1934a, as *P. branchifera* and *P. verruculata*); Red Sea (Semper 1880; Hoffmann 1928; Labbé 1934a); Samoa (Schmeltz 1874); South Africa (Connolly 1939); Vanuatu (Solem 1959); Vietnam (Zvonareva and Kantor 2016, as *Peronia* sp.).

The following records are erroneous, i.e., the names that were used (as *O. verruculatum* or *P. verruculata* unless specified) refer to species that are not *P. verruculata*: Japan, Nagasaki (Keferstein 1865b) and Sagami Bay (Hoffmann 1928) are records of *P. setoensis*; Djibouti (paralectotype of *P. gaimardi*), Red Sea (paralectotype of *P. gondwanae*), South Africa (Hoffmann 1928), and western India (paralectotype of *P. gondwanae*; Awati & Karandikar, 1948) are records of *P. madagascariensis*; Australia, Northern Territory, Darwin (Hoffmann 1928) is a record of *P. willani*; India, Nicobar Islands (Mörch 1872a, b), Japan, Tokara Islands (Baba 1958), and Mauritius (possible paralectotype of *P. gondwanae*) are records of *P. peronii*; Hawaii (Hoffmann 1928; Labbé 1934a; Solem 1959) is a record of *P. platei*. Finally, Britton (1984) recorded from Hong Kong some slugs with intestinal loops of type II as *P. verruculata*; those slugs do not belong to *Peronia* (they likely were *Paromoionchis tumidus*, a species with intestinal loops of type II).

###### Etymology.

The etymology of specific names is treated alphabetically. *Peronia anomala* was named after the supposedly anomalous intestinal loops of type II, except Labbé made a mistake because the intestinal loops are of type I (Fig. 86B).

*Onchidium astridae* is named after Astrid of Sweden [1905–1935], spouse of Prince Leopold [1901–1983], King of the Belgians from 1934 to 1951; the type material of *O. astridae* was collected in 1929 during a scientific journey by Prince Leopold and his wife in the former Dutch East Indies (Indonesia).

*Onchidium branchiferum* was named after the dorsal gills on the dorsal notum.

*Scaphis carbonaria* was named after the (artificial) charcoal color (*carbonaria* in Latin) of the ventrum of the preserved holotype.

*Onchidium durum* was named after the hard (*durum* in Latin) notum of the preserved type specimens.

*Onchidium elberti* was named after Dr. J. Elbert, who collected the holotype in 1909.

*Onchidium ferrugineum* was named after the rusty (*ferrugineum* in Latin) color of the live individuals collected by Lesson which belong to two different species: the lectotype belongs to *Peronia verruculata* (unit #1) and the paralectotypes to *Wallaconchis ater*. The dorsal notum of some individuals of *W. ater* can be homogenously of rusty color (e.g., Goulding et al. 2018b: fig. 36F) but individuals of *P. verruculata* (unit #1) are not typically of rusty color, although their notum commonly displays red patches. Lesson’s (1833: pl. 19, figs 1, 2) illustrations of *Peronia ferruginea* in his *Illustrations de Zoologie* represent a *Peronia* slug with a dorsal notum that is homogenously of rusty color: it almost looks like an individual of *Wallaconchis ater* to which dorsal gills were artificially added.

*Peronia gaimardi* was named after Joseph Paul Gaimard [1793–1858], who collected (with Jean René Constant Quoy) the type material in Vanikoro in 1829 during a voyage of the *Astrolabe*.

*Paraperonia gondwanae* was named after its supposedly Gondwanan distribution (Red Sea, Mauritius, western India, and Torres Strait).

*Scaphis gravieri* was named after Charles Joseph Gravier [1865–1937], professor of zoology (worms and crustaceans) at the MNHN, who collected two paralectotypes from Djibouti.

*Scaphis lata* was named after the broad (*lata* in Latin) and circular shape of preserved type specimens.

*Peronia persiae* was named after the Persian Gulf.

*Peronia savignii* was named after Marie Jules César Lelorgne de Savigny [1777–1851], a French zoologist who participated in Napoleon’s expedition to Egypt and published a plate of illustrations for gastropods (including onchidiids) in the *Description de l’Egypte* (Savigny 1817: pl. 2).

*Scaphis tonkinensis* was named after its type locality in Tonkin, i.e., Vietnam.

*Onchidium verruculatum* was named after the dorsal notum covered with warts (*verruculatum* in Latin).

*Scaphis viridis* was named after the (artificial) green color of the preserved type specimens.

###### Habitat

**Figure 65.**
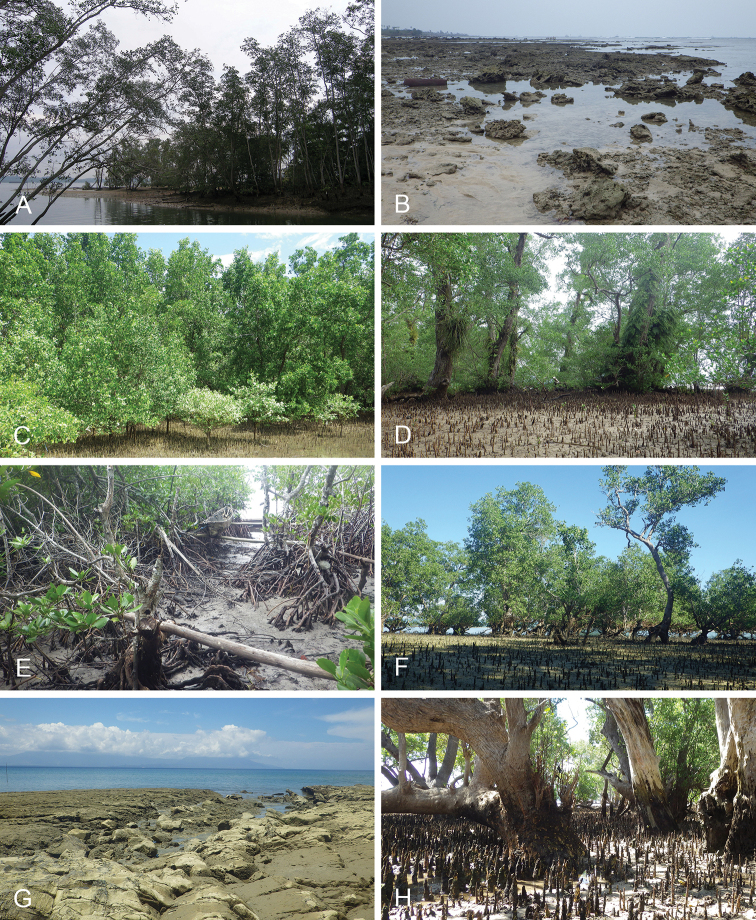
Habitats, *Peronia verruculata* (unit #1) **A** Singapore **B–H** Indonesia **A** Mangrove forest with rich litter, lobster mounds, dead logs, with sand area near the creek (st 5) **B** Sumatra, coral rubble on beach exposed to estuary (st 78) **C** Sulawesi, old *Sonneratia* and *Avicennia* mangrove (st 84) **D** Ambon, narrow band of old *Avicennia* trees on sandy mud, old logs on ground (st 132) **E** Seram, mud next to a mangrove (st 135) **F** Lombok, old *Avicennia* forest with coral rubble (st 149) **G** Halmahera, rocky shore near a beach (st 217) **H** Timor, sandy part of mangrove with *Sonneratia* and *Avicennia* trees (st 250).

**Figure 66.**
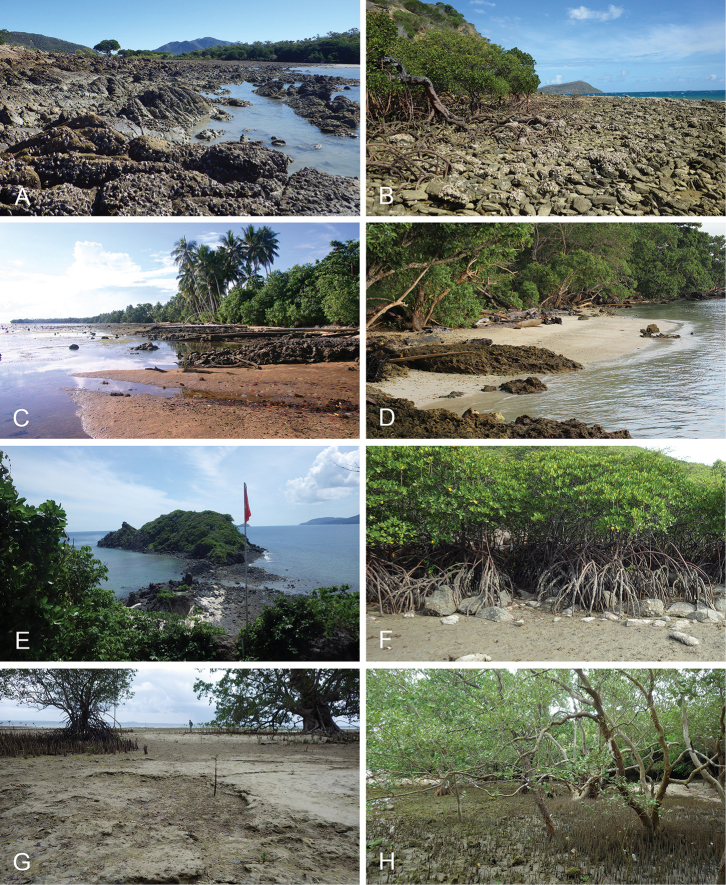
Habitats, *Peronia verruculata* (unit #1) **A** Australia, Queensland, rocky shore nearby a small and dense *Rhizophora* mangrove patch (st 113) **B** New Caledonia, coastal rocky mangrove (st KM 537) **C** Papua New Guinea, New Ireland, mixed hard platform and seagrass bed at outlet of rivulet (st KM 05) **D** Papua New Guinea, Madang, limestone rocky intertidal (st PM 12) **E** Vietnam, mangrove patch with many big flat rocks outside (st 236) **F** Vietnam, coral rubble near small *Rhizophora* sandy and muddy mangrove (st 238) **G** Philippines, Bohol, coral rubble with sand and algae, near *Sonneratia* (st 202) **H** sandy, open *Avicennia* forest, right by the shore (st 181).

**Figure 67.**
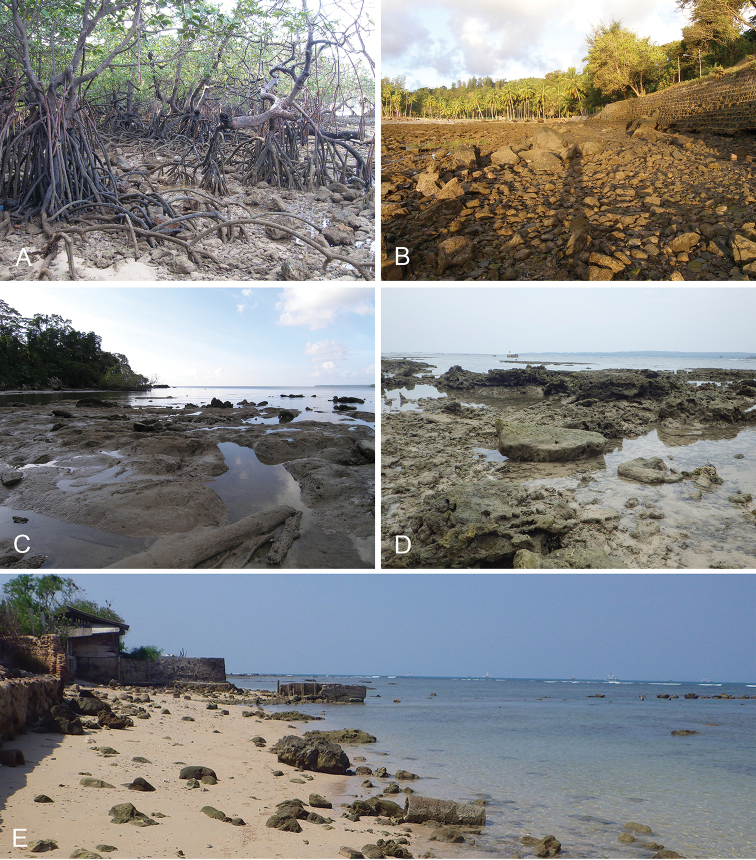
Habitats, *Peronia verruculata* (unit #2) **A** India, South Andaman, rocky shore with a patch of *Rhizophora*, sand and coral rubble but no mud (st 53) **B** India, South Andaman, rocky shore only, no mangrove (st 54) **C** India, South Andaman, sandy beach with coral rubble (st 53) **D** Indonesia, Sumatra, coral rubble on beach exposed to estuary (st 78) **E** Indonesia, Sumatra, beach with a few rocks (st 82).

**Figure 68.**
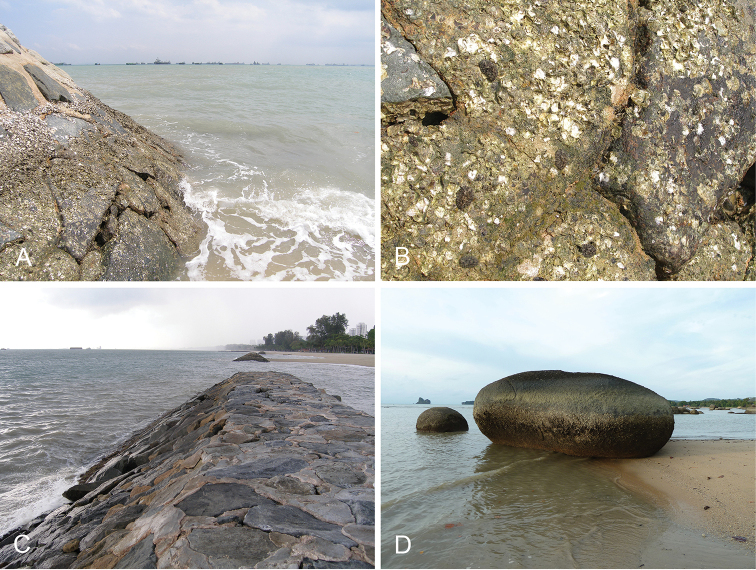
Habitats, *Peronia verruculata* (unit #3) **A** Singapore, rocky shore covered by oyster flats (st 2) **B** slugs on the rocks, same as A (st 2) **C** Singapore, rocks of artificial breakwaters (st 3) **D** Peninsular Malaysia, Langkawi, large boulders on sand beach (st 25).

**Figure 69.**
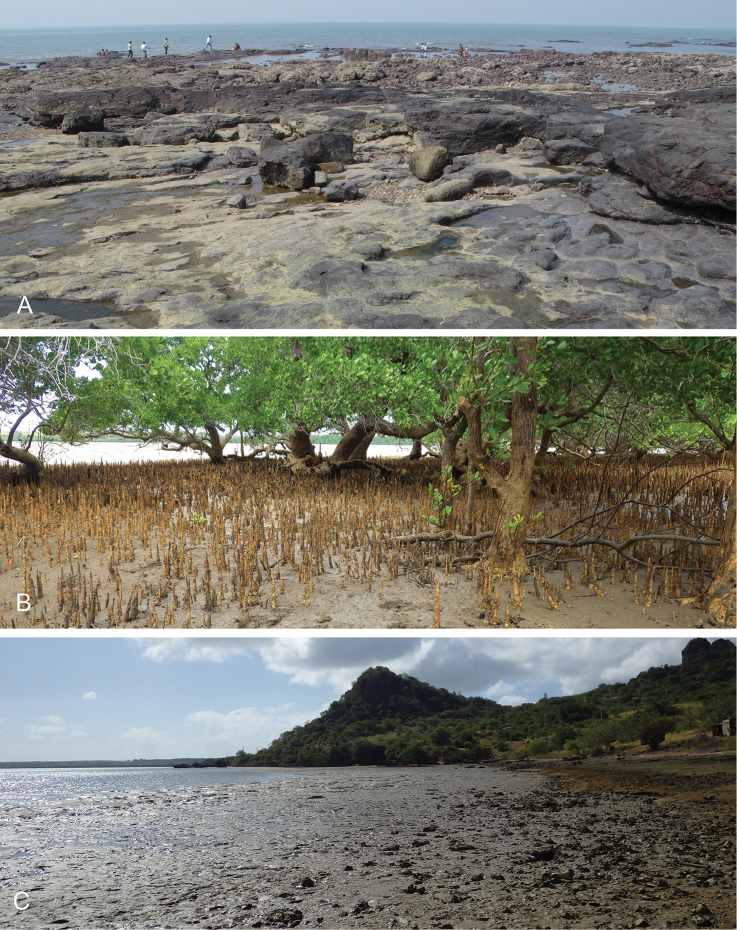
Habitats, *Peronia verruculata***A** unit #4 **B, C** unit #5 **A** India, Mumbai, solid rock area, some crevices (st 44) **B** Madagascar, sandy mangrove of *Bruguiera*, on sandy mud in between rocks (st 162) **C** Madagascar, rocky platform near *Avicennia* and *Rhizophora* mangrove (st 158).

(Figs 65–69). Unit #1 is found in a large variety of habitats. It is predominantly found on rocks in the rocky intertidal (including man-made structures). It can also be found on huge and isolated boulders on a sandy beach or in coral rubble mixed or not with sand. The rocks on which the unit #1 is found can be associated or not with sparse mangrove trees. It is also found on sandy mud inside or nearby mangroves. Exceptionally, it can be found on old logs inside muddy mangroves. Unit #2 is found on coral rubble and rocks on sandy beaches. Unit #3 is found on rocks on a beach and in the rocky intertidal. Unit #4 is found in the rocky intertidal. Unit #5 is found in the rocky intertidal as well as on mud, sandy or not. There was no habitat data on the labels of the material studied here for unit #6 but it is most likely found in the rocky intertidal, like the other units of *Peronia verruculata*.

*Peronia verruculata* is extremely common across its entire distribution. In localities where they overlap geographically, the different mitochondrial units are found more or less in equal abundance (units #1 and #3 in Singapore, and units #1 and #2 in southeastern Sumatra). *Peronia verruculata* is commonly found during the day, even though a few individuals were also collected at night.

###### Color and morphology of live animals

**Figure 70.**
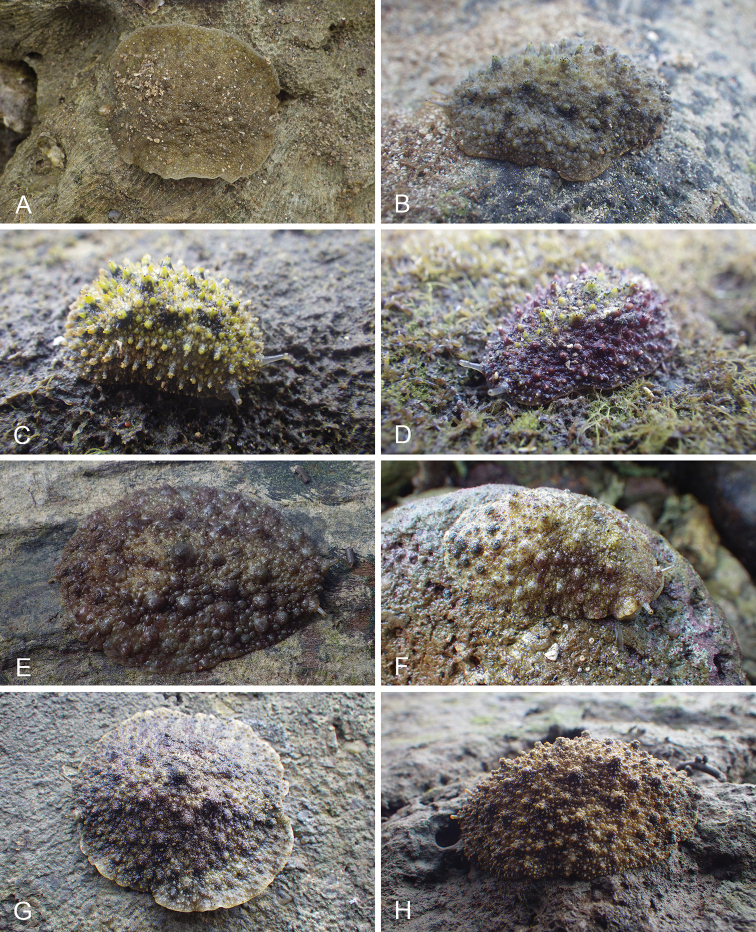
Live animals, dorsal view, *Peronia verruculata* (unit #1), Indonesia **A** 40 mm long [1747], Sumatra (UMIZ 00172) **B** 40 mm long [2987], Lombok (UMIZ 00168) **C** 20 mm long [3080], Bali (UMIZ 00164) **D** 20 mm long [3115], Bali (UMIZ 00165) **E** 45 mm long [2856], Ambon (UMIZ 00163) **F** 40 mm long [2729], Ambon (UMIZ 00162) **G** 50 mm long [2868], Seram (UMIZ 00169) **H** 55 mm long [3441], Seram (UMIZ 00169).

**Figure 71.**
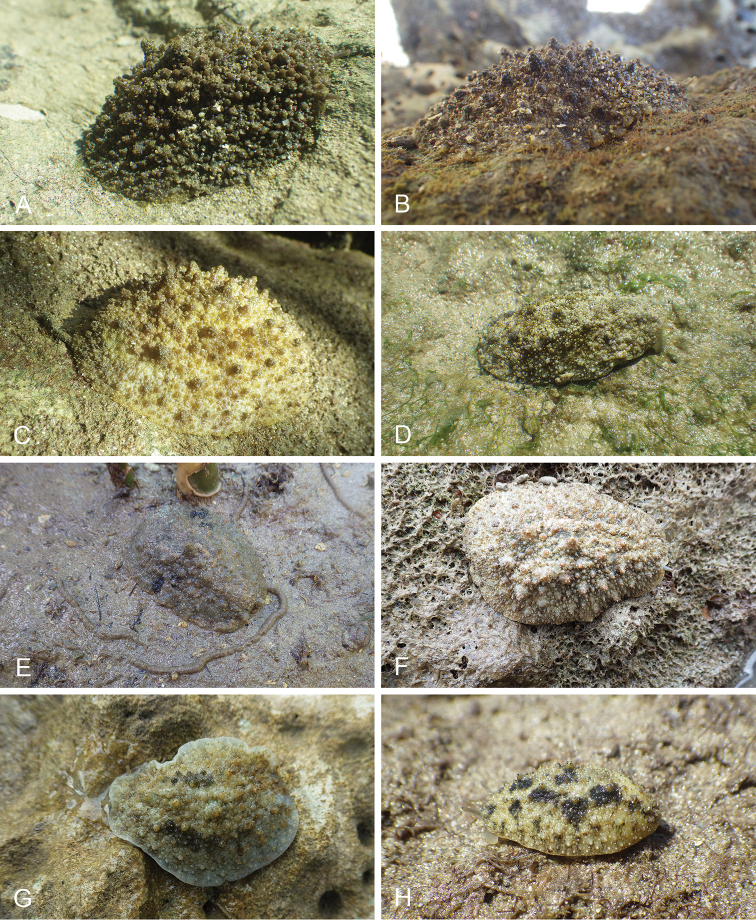
Live animals, dorsal view, *Peronia verruculata* (unit #1) **A–E** Indonesia **F–H** Vietnam **A** 35 mm long [5130], Halmahera (UMIZ 00167) **B** 40 mm long [5120], Halmahera (UMIZ 00167) **C** 50 mm long [5124], Halmahera (UMIZ 00167) **D** 35 mm long [5927], Timor (UMIZ 00175) **E** 45 mm long [5904], Timor (UMIZ 00174) **F** 60 mm long [5620] (ITBZC IM 00021) **G** 40 mm long [5621] (ITBZC IM 00021) **H** 17 mm long [5639] (ITBZC IM 00023).

**Figure 72.**
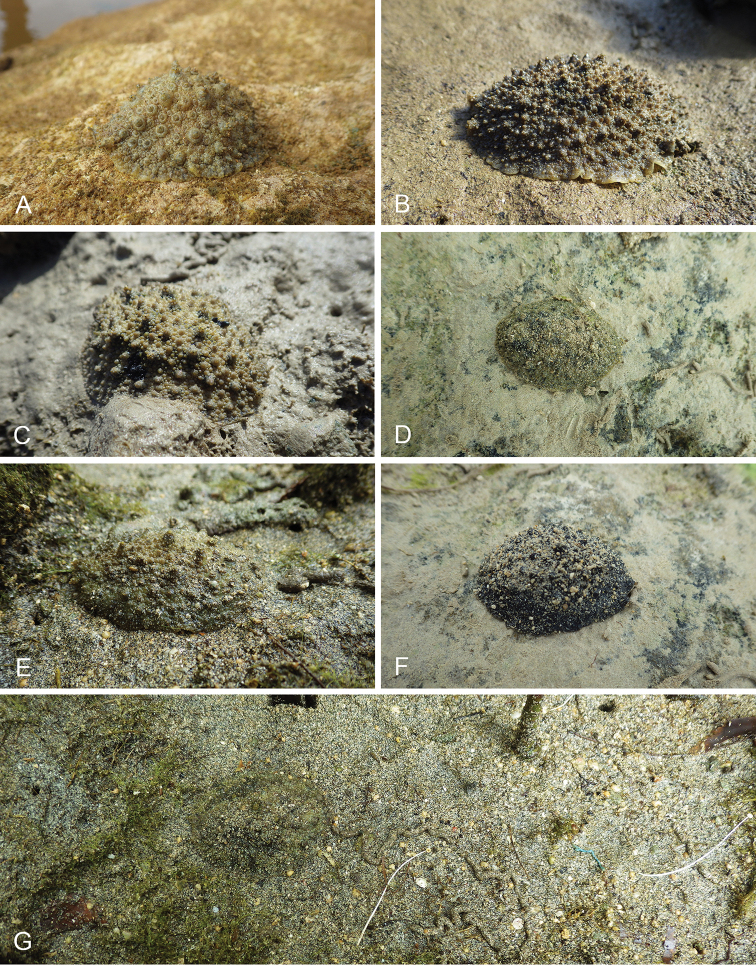
Live animals, dorsal view, *Peronia verruculata* (unit #1) **A–C** New Caledonia **D–G** Philippines **A** 50 mm long [6202] (MNHN-IM-2019-1591) **B** 73 mm long [6212] (MNHN-IM-2019-1592) **C** 43 mm long [6214] (MNHN-IM-2019-1593) **D** 35 mm long [3437], Bohol (PNM 041276) **E** 50 mm long [3160], Luzon (PNM 041277) **F** 30 mm long [3433], Bohol (PNM 041276) **G** 40 mm long [3161], Luzon (PNM 041277).

**Figure 73.**
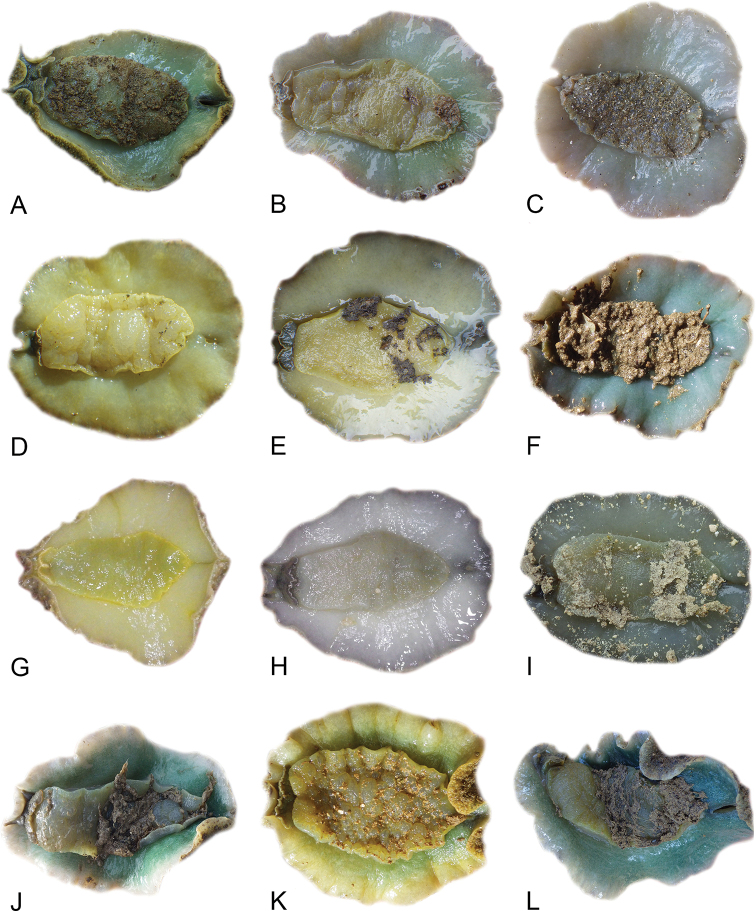
Live animals, ventral view, *Peronia verruculata* (unit #1) **A–F** Indonesia **G–I** Philippines, Bohol **J** Vietnam **K, L** New Caledonia **A** 45 mm long [2127], Sulawesi (UMIZ 00170) **B** 60 mm long [2162], Sulawesi (UMIZ 00171) **C** 50 mm long [5124], Halmahera (UMIZ 00167) **D** 40 mm long [5120], Halmahera (UMIZ 00167) **E** 50 mm long [2868], Seram (UMIZ 00169) **F** 45 mm long [5904], Timor (UMIZ 00174) **G** 40 mm long [3379] (PNM 041274) **H** 35 mm long [3380] (PNM 041274) **I** 30 mm long [3433] (PNM 041276) **J** 60 mm long [5620] (ITBZC IM 00021) **K** 50 mm long [6202] (MNHN-IM-2019-1591) **L** 73 mm long [6212] (MNHN-IM-2019-1592).

**Figure 74.**
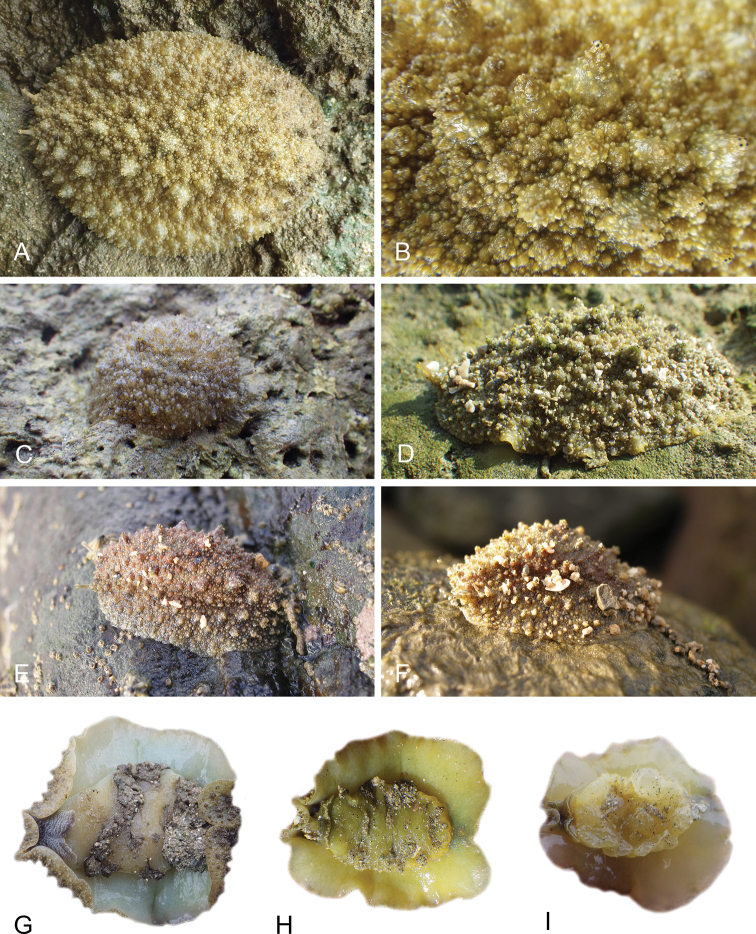
Live animals, *Peronia verruculata* (unit #2) **A** dorsal view, 50 mm long [1746], Indonesia, Sumatra (UMIZ 00178) **B** dorsal notum, detail, same as **A**; **C** dorsal view, 25 mm long [1741], Indonesia, Sumatra (UMIZ 00179) **D** dorsal view, 45 mm long [1795], Indonesia, Sumatra (UMIZ 00180) **E** dorsal view, 30 mm long [1080], India, South Andaman (BNHS 121) **F** dorsal view, 15 mm long [1079], India, South Andaman (BNHS 120) **G** Ventral view, same as **A**; **H** ventral view, same as **D**; **I** ventral view, same as **C**.

**Figure 75.**
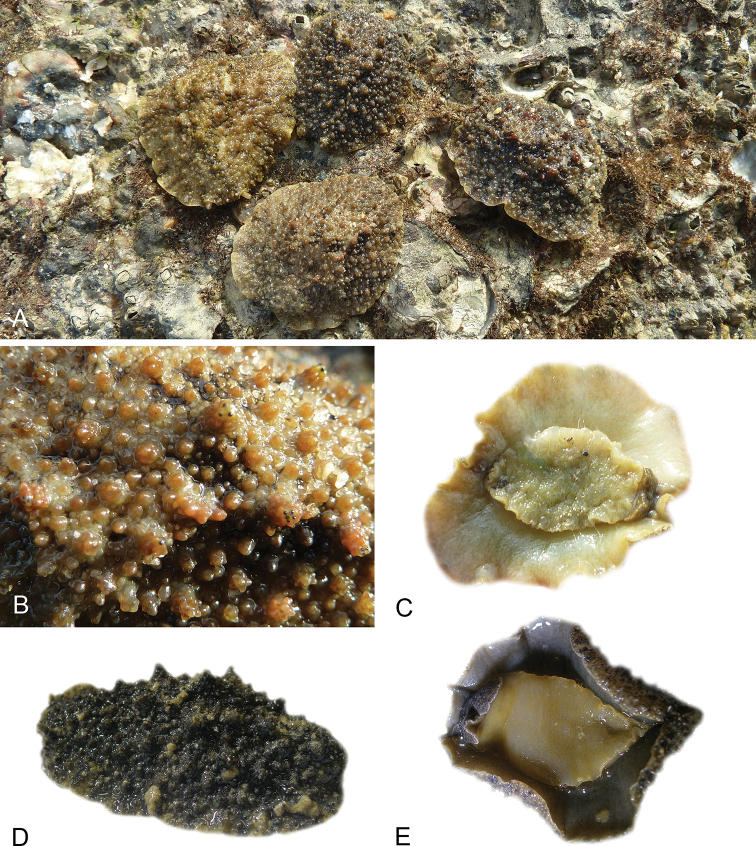
Live animals, *Peronia verruculata* (unit #3) **A** dorsal view, 35 mm long [974] at the bottom, 27 mm long [975] at the top, 35 mm long [976] on the left, 27 mm long [977] on the right, Peninsular Malaysia, Langkawi (USMMC 00051 & 00064) **B** dorsal notum, detail [974], Peninsular Malaysia, Langkawi (USMMC 00064) **C** ventral view, 35 mm long [976], Peninsular Malaysia, Langkawi (USMMC 00051) **D** dorsal view, 25 mm long [990], Singapore (ZRC.MOL.10496) **E** ventral view, same as **D**.

**Figure 76.**
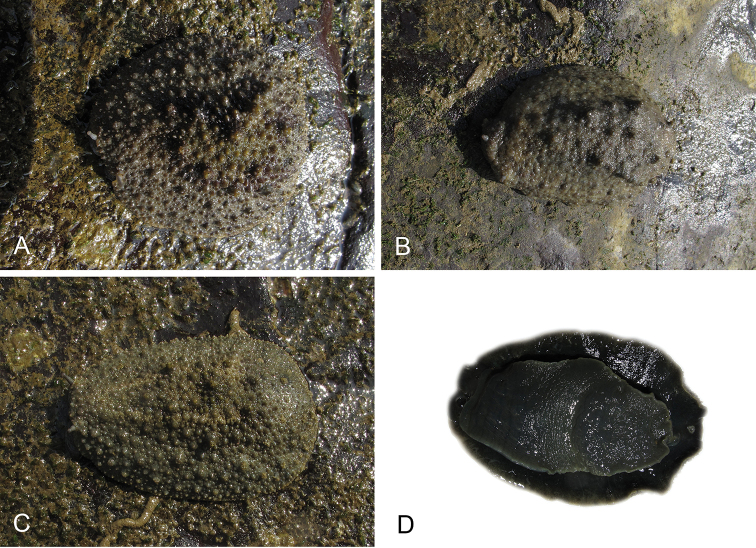
Live animals, *Peronia verruculata* (unit #4), India, Mumbai **A** dorsal view, 55 mm long [1141] (BNHS 22) **B** dorsal view, 60 mm long [1143] (BNHS 24) **C** dorsal view, 60 mm long [1144] (BNHS 23) **D** ventral view, same as **C**.

**Figure 77.**
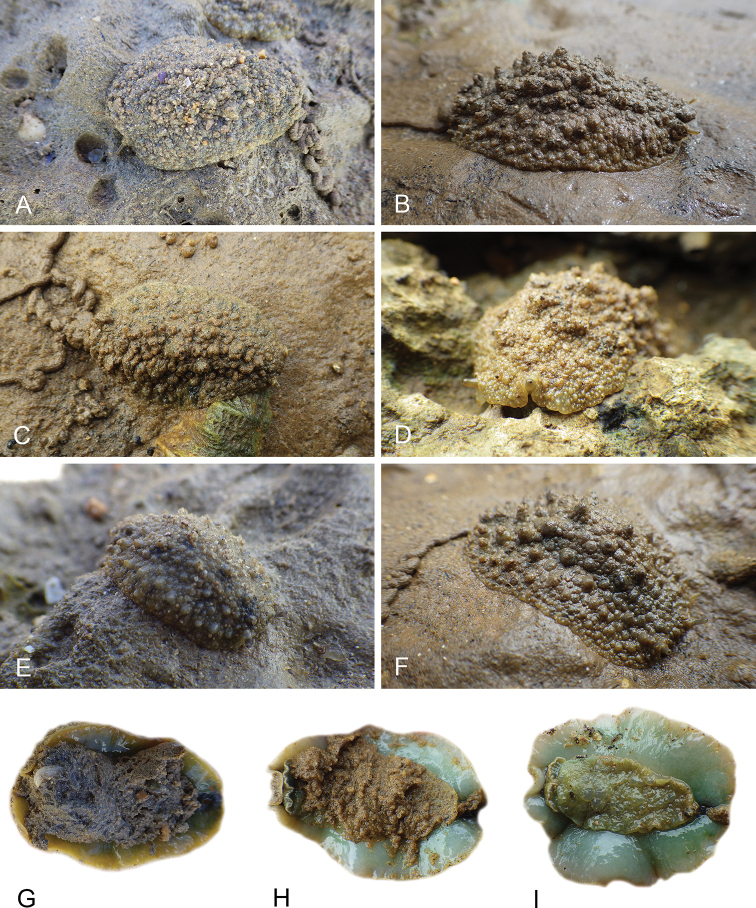
Live animals, *Peronia verruculata* (unit #5), Madagascar **A** dorsal view, 30 mm long [3142] (MNHN-IM-2019-1610) **B** dorsal view, 50 mm long [3143] (MNHN-IM-2019-1611) **C** dorsal view, 30 mm long [3146] (MNHN-IM-2019-1611) **D** dorsal view, 30 mm long [3149] (MNHN-IM-2019-1611) **E** dorsal view, 13 mm long [3598] (MNHN-IM-2019-1610) **F** dorsal view, 30 mm long [3600] (MNHN-IM-2019-1611) **G** ventral view, same as **A**; **H** ventral view, same as **C**; **I** ventral view, same as **D**.

(Figs 70–77). In unit #1, live animals are not covered with mud, but they can often bear tiny pieces of various materials, such as sand and broken shells (Figs 70–73). The background color of the dorsal notum is highly variable, most often brown (light to dark), or greenish, and occasionally even black. The background is mottled with darker areas, occasionally with red areas. In most animals, the color of the dorsal papillae varies as that of the background itself. In some animals, however, the tip of the dorsal papillae (with and without dorsal eyes) can be bright yellow. The color of the foot is the same as that of the hyponotum, which varies greatly from pure white to dark blue-green. In most animals, the ventral surface is yellowish-greenish or yellowish-bluish. The ventral color (foot and hyponotum) of an individual can change rapidly, especially when disturbed. The ocular tentacles are brown-grey (variable from light to dark), like the head. The ocular tentacles are short (just a few millimeters long). Preserved specimens no longer display the colors of live animals. Colors tend to fade rapidly with preservation.

The dorsal notum of live animals is covered by dozens of papillae of various sizes. Those papillae do not retract within the notum, whether animals are disturbed or not, and so the dorsal notum is never smooth. Larger papillae are not arranged in two longitudinal and lateral ridges (on either side of the median line), even though larger papillae are mostly concentrated in the central area of the dorsal notum. Some papillae bear from one to five black dorsal eyes at their tip (most papillae bear three eyes). The number of papillae with dorsal eyes is variable (from 10 to 22) and papillae in the central area of the dorsum tend to bear more eyes than those on the side. Occasionally, papillae can bear more than five eyes: a central, large papilla can bear up to eight eyes but, like other papillae, is not fully retractable within the notum. The exact number of papillae with eyes can be difficult to count because papillae are often dark, and because the eyes, which are located at the tip of the papillae, can be seen only if papillae are relaxed. Dorsal gills are present on the posterior third of the dorsal notum. Dorsal gills are most easily observed when animals are relaxed under water. When slugs are not under water, dorsal gills are retracted and hard to see. If animals were not relaxed before preservation, gills can be retracted and hard to see in preserved specimens (the best relaxation method is to immerse live specimens in a solution of magnesium chloride).

The color variation in unit #2 (Fig. 74) and unit #3 (Fig. 75) is similar to the color variation in unit #1, and specimens cannot be separated where units overlap geographically (in Singapore for units #1 and #2, and in southeastern Sumatra for units #1 and #3). The number of papillae with dorsal eyes observed in unit #2 (from 14 to 22) and in unit #3 (from 10 to 18) is within the range observed in unit #1. Slight differences may be due to a more limited sampling.

In unit #4, the color of the dorsal notum is brown, mottled with darker and lighter areas (Fig. 76). The ventral surface (foot and hyponotum) is brown-greyish. The number of papillae with dorsal eyes varies from 10 to 18.

In unit #5, the dorsal notum is brown, light to dark, mottled with darker areas (Fig. 77). The ventral surface (foot and hyponotum) is yellowish, greenish, or bluish, and can change rapidly in any given individual. The number of papillae with dorsal eyes varies from 10 to 20.

Pictures of live animals were not available for unit #6 (Red Sea). The dorsal color of preserved specimens is beige with faded darker areas. The ventrum is beige. The number of papillae with dorsal eyes varies from 10 to 18, but the black eye color possibly faded in some of them.

The largest specimens are 60 mm long in unit #1, 55 mm long in unit #2, 40 mm long in unit #3, 60 mm long in unit #4, 50 mm long in unit #5, and 40 mm long in unit #6. Exceptionally, one individual in New Caledonia was 73 mm long (unit #1).

###### External morphology

**Figure 78.**
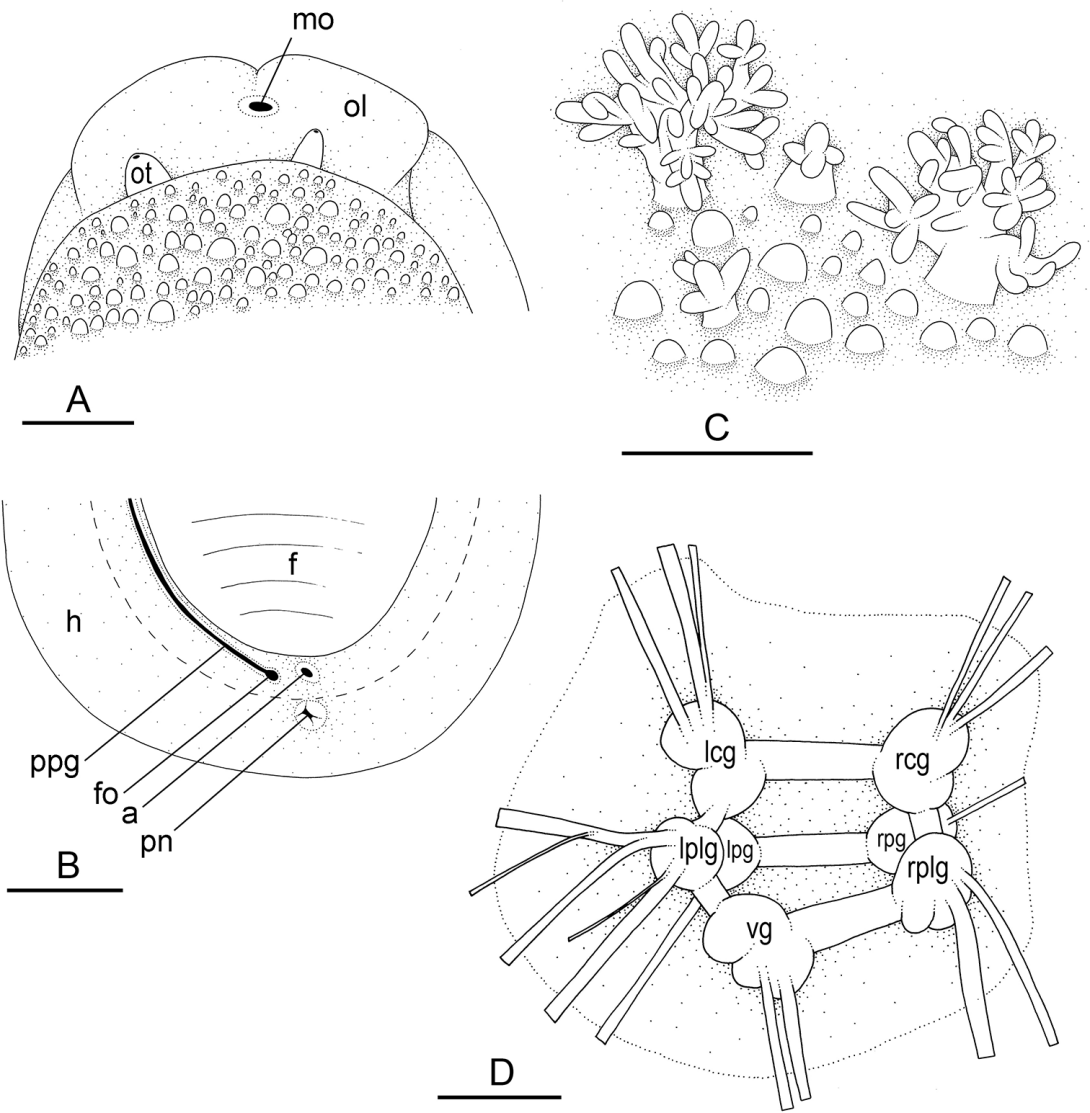
External morphology and nervous system, *Peronia verruculata* (unit #1), Indonesia, Sulawesi [2127] (UMIZ 00170) **A** anterior, ventral view **B** posterior, ventral view, the dotted line shows where the foot normally expands (it was partly cut to illustrate the anus, the peripodial groove, and the female pore) **C** dorsal gills and papillae **D** nervous system, dorsal view. Scale bars: 5 mm (**A, B**), 2 mm (**C**), 1 mm (**D**). Abbreviations: a anus, f foot, fo female opening, h hyponotum, lcg left cerebral ganglion, lpg left pedal glanglion, lplg left pleural ganglion, mo male opening, ol oral lobe, ot ocular tentacle, pn pneumostome, ppg peripodial groove, rcg right cerebral ganglion, rpg right pedal glanglion, rplg right pleural ganglion, vg visceral ganglion.

(Fig. 78A–C). The body is not flattened. The notum is oval. The hyponotum is horizontal in live animals. The orientation of the hyponotum as well as the shape of the dorsal notum of preserved animals greatly vary depending on preservation. The width of the hyponotum relative to the total width of the ventral surface (pedal sole and hyponotum) varies among individuals but is approximately one third. In the anterior region, the left and right ocular tentacles are superior to the mouth. Eyes are located at the tip of the two ocular tentacles. Inferior to the ocular tentacles, superior to the mouth, the head bears a pair of oral lobes. The latter are smooth, with no transversal protuberance. The male opening (of the copulatory complex) is below and to the left of the right ocular tentacle (i.e., between the two ocular tentacles, but closer to the right than to the left tentacle). The anus is posterior, median, close to the edge of the pedal sole. On the right side (to the left in ventral view), a peripodial groove is present at the junction between the foot and the hyponotum, running longitudinally all the way from the head to the posterior end. The female pore, which marks the posterior end of the peripodial groove, is located a few millimeters from the anus and the pneumostome, which does not vary much among individuals. The pneumostome is median. Its position on the hyponotum relative to the notum margin and the edge of the pedal sole varies among individuals but averages in the middle.

###### Visceral cavity and pallial complex.

The anterior pedal gland is small, more or less round, and flattened, lying on the floor of the visceral cavity below the buccal mass and below a thin layer of connective tissue (it can be hard to detect). The heart, enclosed in the pericardium, is on the right side of the visceral cavity, slightly posterior to the middle. An anterior vessel supports several anterior organs such as the buccal mass, the nervous system, and the copulatory complex. The kidney is nearly symmetrical, the right and left parts being equally developed. The kidney is intricately attached to the respiratory complex. The lung is posterior in two more or less symmetrical parts, left and right, which are joined in the middle.

###### Nervous system

(Fig. 78D). The circum-esophageal nerve ring is post-pharyngeal and pre-esophageal. The paired cerebral ganglia are separated by a short cerebral commissure of which the length varies among individuals. Paired pleural and pedal ganglia are also all distinct. The visceral commissure is short but distinctly present and the visceral ganglion tends to be slightly to the left. Cerebro-pleural and pleuro-pedal connectives are short and pleural and cerebral ganglia touch each other on either side. Nerves from the cerebral ganglia innervate the buccal area and the ocular tentacles and, on the right side, the penial complex. Nerves from the pedal ganglia innervate the foot. Nerves from the pleural ganglia innervate the lateral and dorsal regions of the mantle. Nerves from the visceral ganglia innervate the visceral organs. Ganglia are commonly surrounded by almost transparent connective tissue through which they can be observed.

###### Digestive system

**Figure 79.**
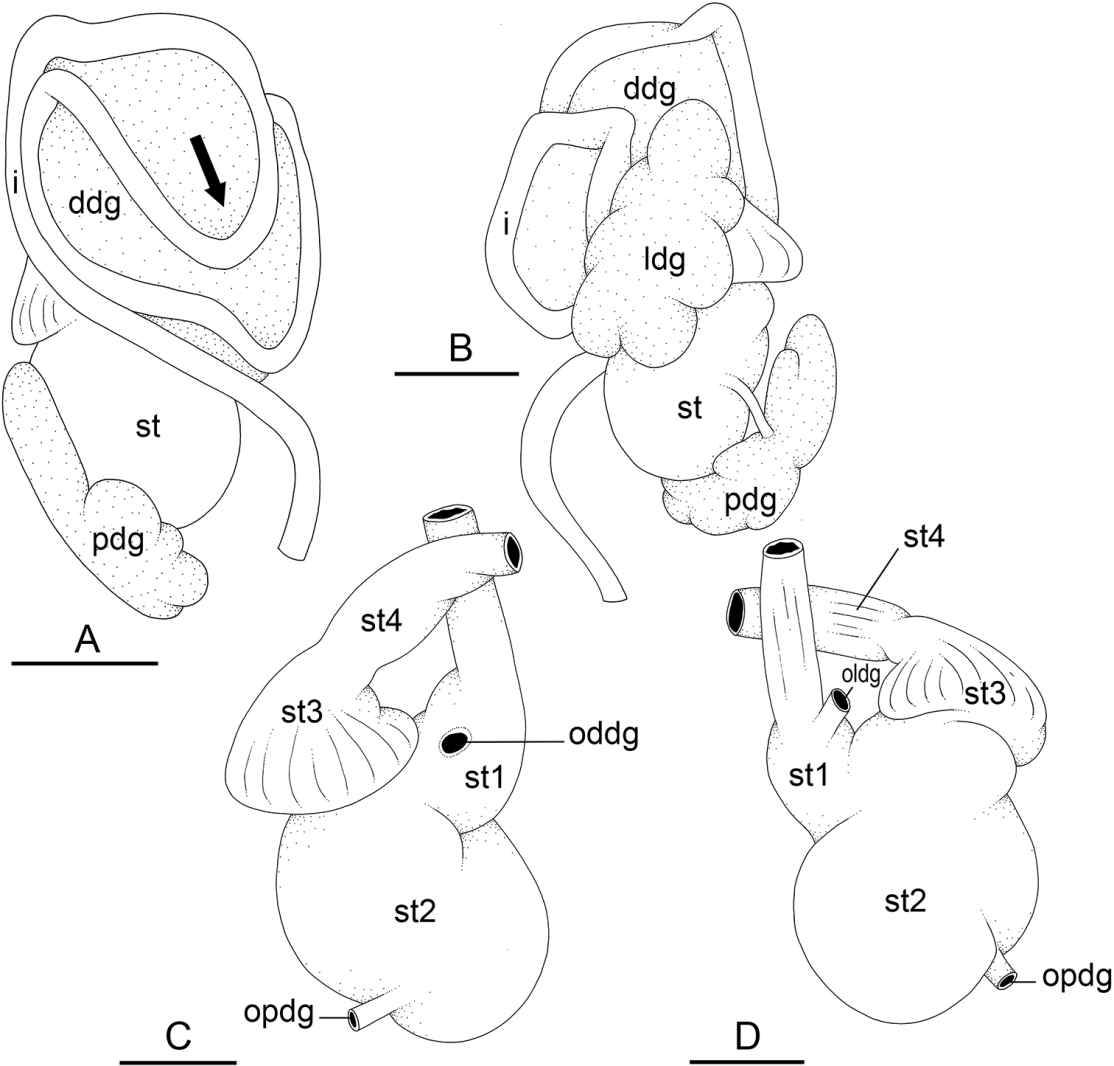
Digestive system, *Peronia verruculata* (unit #1), Indonesia, Sulawesi [2127] (UMIZ 00170) **A** dorsal view, the arrow indicates the orientation of the transitional loop **B** ventral view **C** stomach, dorsal view **D** stomach, ventral view. Scale bars: 5 mm (**A, B**), 4 mm (**C, D**). Abbreviations: ddg dorsal digestive gland, i intestine, ldg lateral digestive gland, oddg opening of the dorsal lobe of the digestive gland, oldg opening of the lateral lobe of the digestive gland, opdg opening of the posterior lobe of the digestive gland, pdg posterior digestive gland, st stomach, st1 first stomach chamber, st2 second stomach chamber, st3 third stomach chamber, st4 fourth stomach chamber.

**Figure 80.**
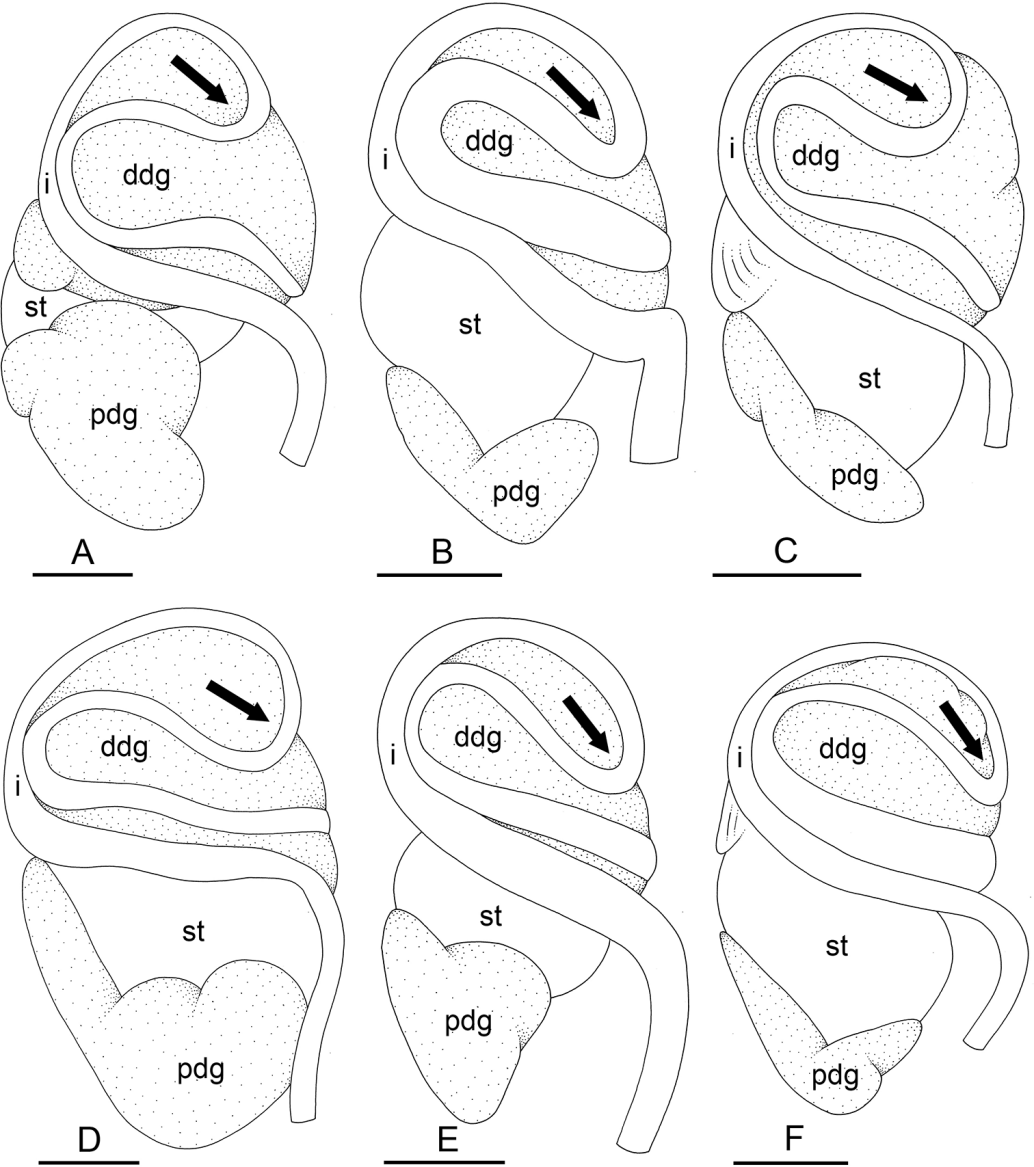
Digestive system, dorsal view, *Peronia verruculata* (unit #1), type specimens. The arrow indicates the orientation of the transitional loop **A** lectotype, *Onchidium ferrugineum* (MNHN-IM-2000-22951) **B** holotype, *Onchidium elberti* (SMF 45248) **C** holotype, *Scaphis astridae* (RBINS I.G.9223/MT.3822) **D** holotype, *Scaphis carbonaria* (MNHN-IM-2000-33708) **E** lectotype, *Peronia gaimardi* (MNHN-IM-2000-33705) **F** lectotype, *Scaphis viridis* (MNHN-IM-2000-22964). Scale bars: 4 mm (**A–C**), 5 mm (**D–F**). Abbreviations: ddg dorsal digestive gland, i intestine, pdg posterior digestive gland, st stomach.

**Figure 81.**
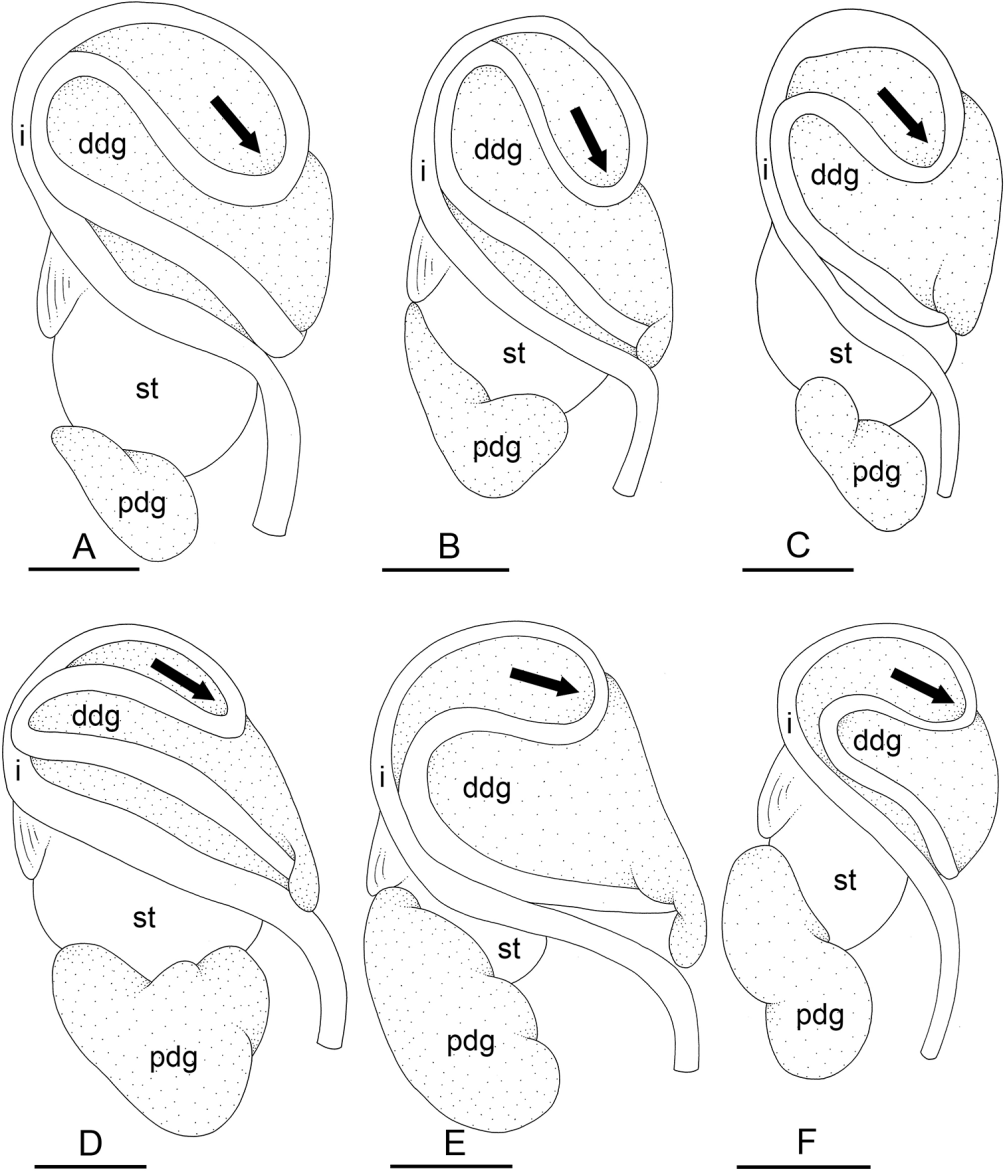
Digestive system, dorsal view, *Peronia verruculata* (unit #1). The arrow indicates the orientation of the transitional loop **A** Palau [698] (UF 253871) **B** Singapore [991] (ZRC.MOL.10497) **C** Australia, Queensland [2622] (MTQ) **D** Philippines, Bohol [3380] (PNM 041274) **E** Philippines, Bohol [3433] (PNM 041276) **F** Japan, Honshu [3751] (NSMT-Mo 78988). Scale bars: 5 mm (**A, D–F**), 3 mm (**B, C**). Abbreviations: ddg dorsal digestive gland, i intestine, pdg posterior digestive gland, st stomach.

**Figure 82.**
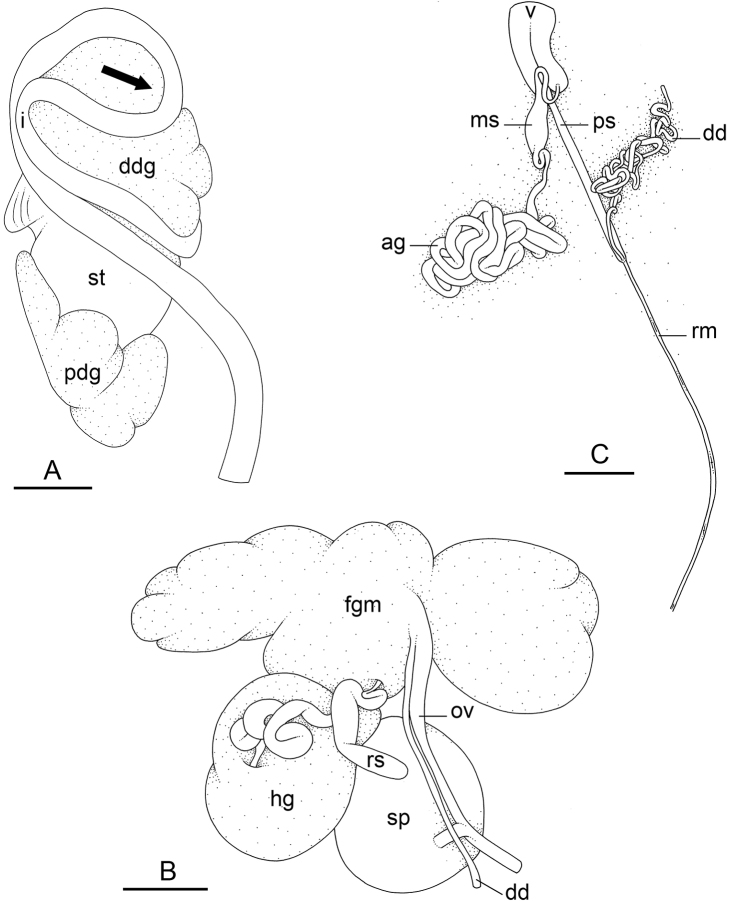
*Peronia verruculata* (unit #2), Indonesia, Sumatra, [1746] (UMIZ 00178) **A** digestive system, dorsal view, the arrow indicates the orientation of the transitional loop **B** posterior, hermaphroditic (female) reproductive system **C** anterior, male, copulatory apparatus. Scale bars: 5 mm (**A, C**), 4 mm (**B**). Abbreviations: ag accessory penial gland, dd deferent duct, ddg dorsal digestive gland, fgm female gland mass, hg hermaphroditic gland, i intestine, ms muscular sac, ov oviduct, pdg posterior digestive gland, ps penial sheath, rm retractor muscle, rs receptaculum seminis, sp spermatheca, st stomach, v vestibule.

**Figure 83.**
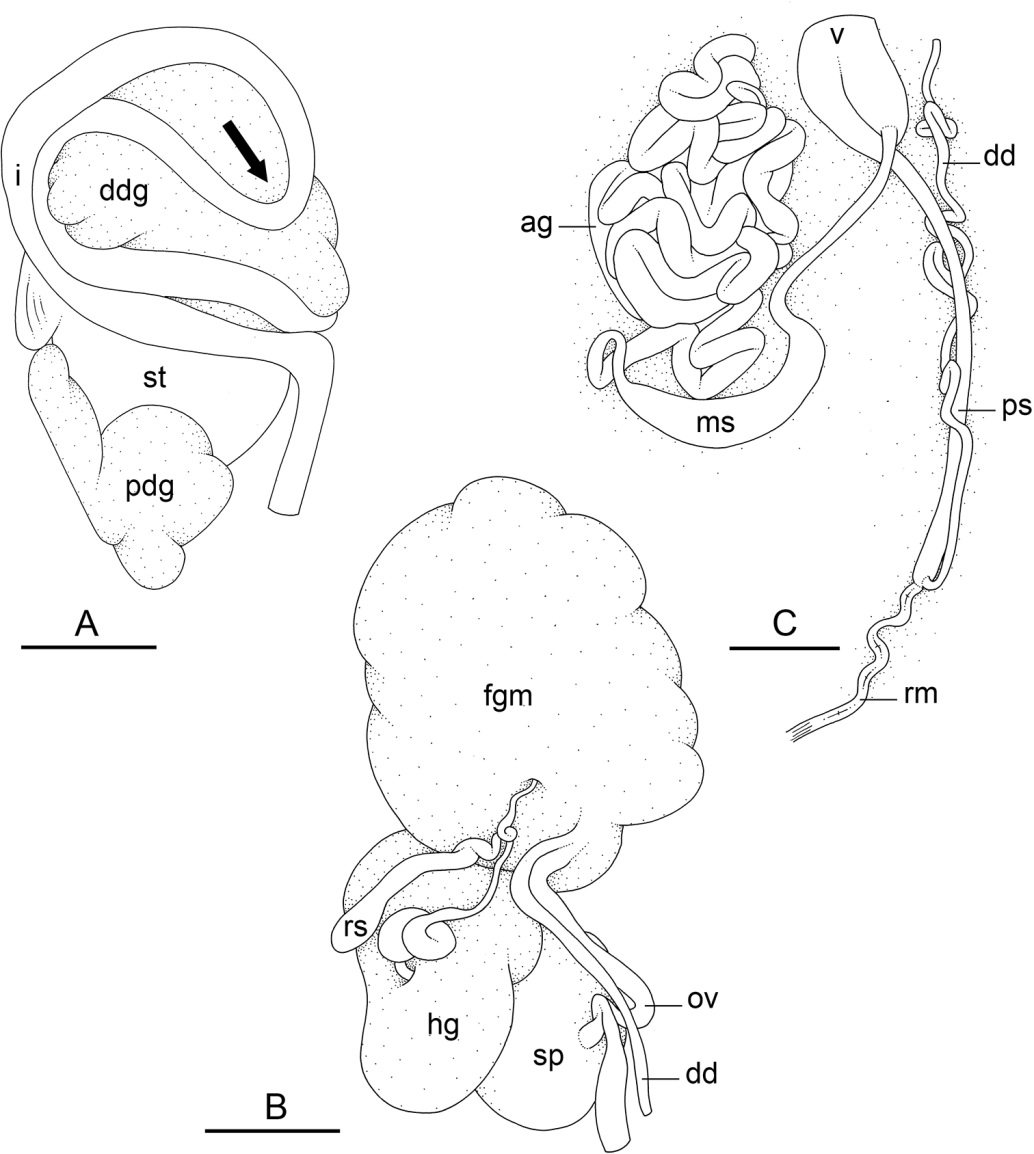
*Peronia verruculata* (unit #3), Peninsular Malaysia, Langkawi, [976] (USMMC 00051) **A** digestive system, dorsal view, the arrow indicates the orientation of the transitional loop **B** posterior, hermaphroditic (female) reproductive system **C** anterior, male, copulatory apparatus. Scale bars: 5 mm (**A**), 3 mm (**B**), 4 mm (**C**). Abbreviations: ag accessory penial gland, dd deferent duct, ddg dorsal digestive gland, fgm female gland mass, hg hermaphroditic gland, i intestine, ms muscular sac, ov oviduct, pdg posterior digestive gland, ps penial sheath, rm retractor muscle, rs receptaculum seminis, sp spermatheca, st stomach, v vestibule.

**Figure 84.**
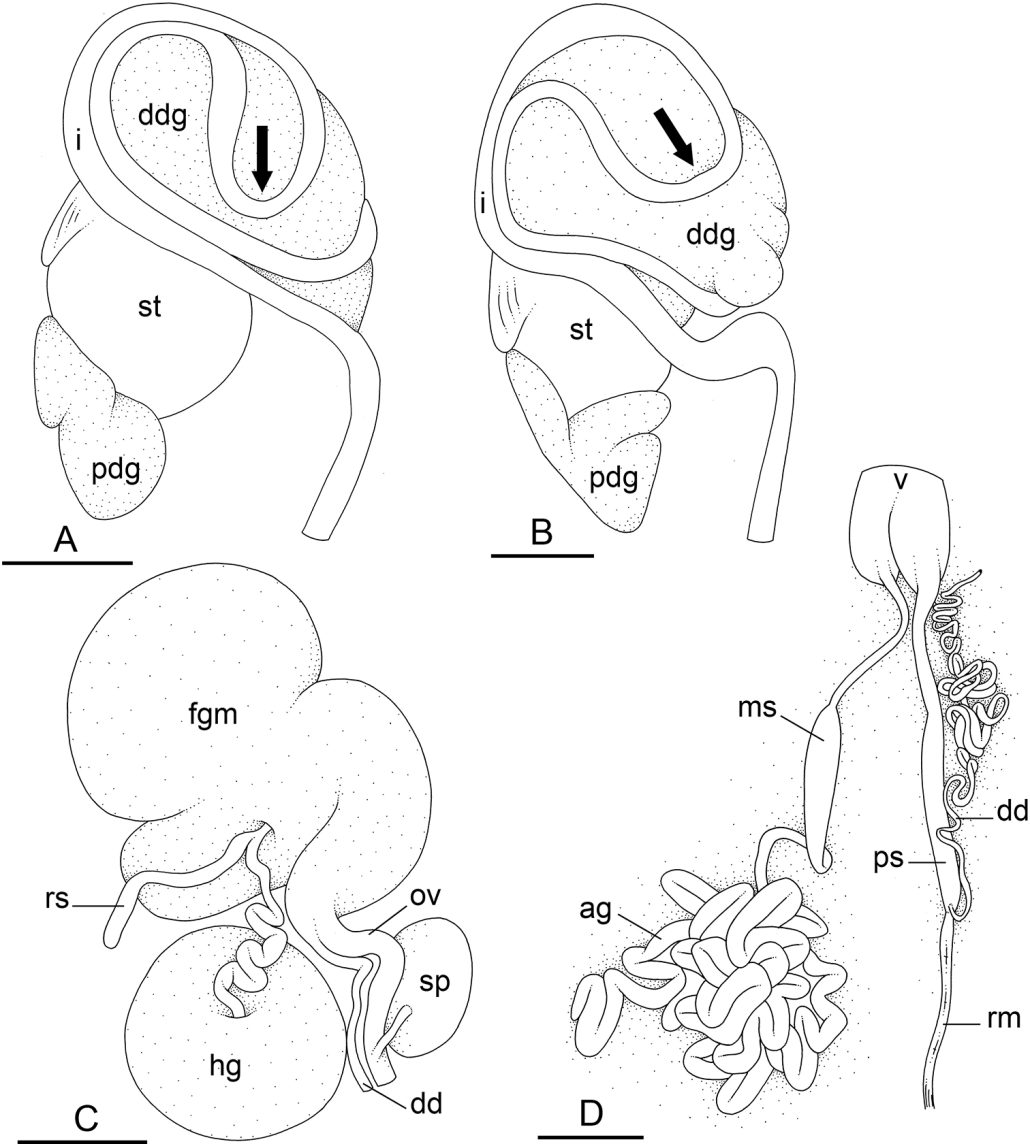
*Peronia verruculata* (unit #4) **A** lectotype, *Paraperonia gondwanae*, India, Mumbai (MNHN-IM-2000-33681) **B–D** Pakistan, [6164] (MNHN-IM-2019-**1384**) **A** digestive system, dorsal view, the arrow indicates the orientation of the transitional loop **B** digestive system, dorsal view **C** posterior, hermaphroditic (female) reproductive system **D** anterior, male, copulatory apparatus. Scale bars: 4 mm (**A, C**), 5 mm (**B, D**). Abbreviations: ag accessory penial gland, dd deferent duct, ddg dorsal digestive gland, fgm female gland mass, hg hermaphroditic gland, i intestine, ms muscular sac, ov oviduct, pdg posterior digestive gland, ps penial sheath, rm retractor muscle, rs receptaculum seminis, sp spermatheca, st stomach, v vestibule.

**Figure 85.**
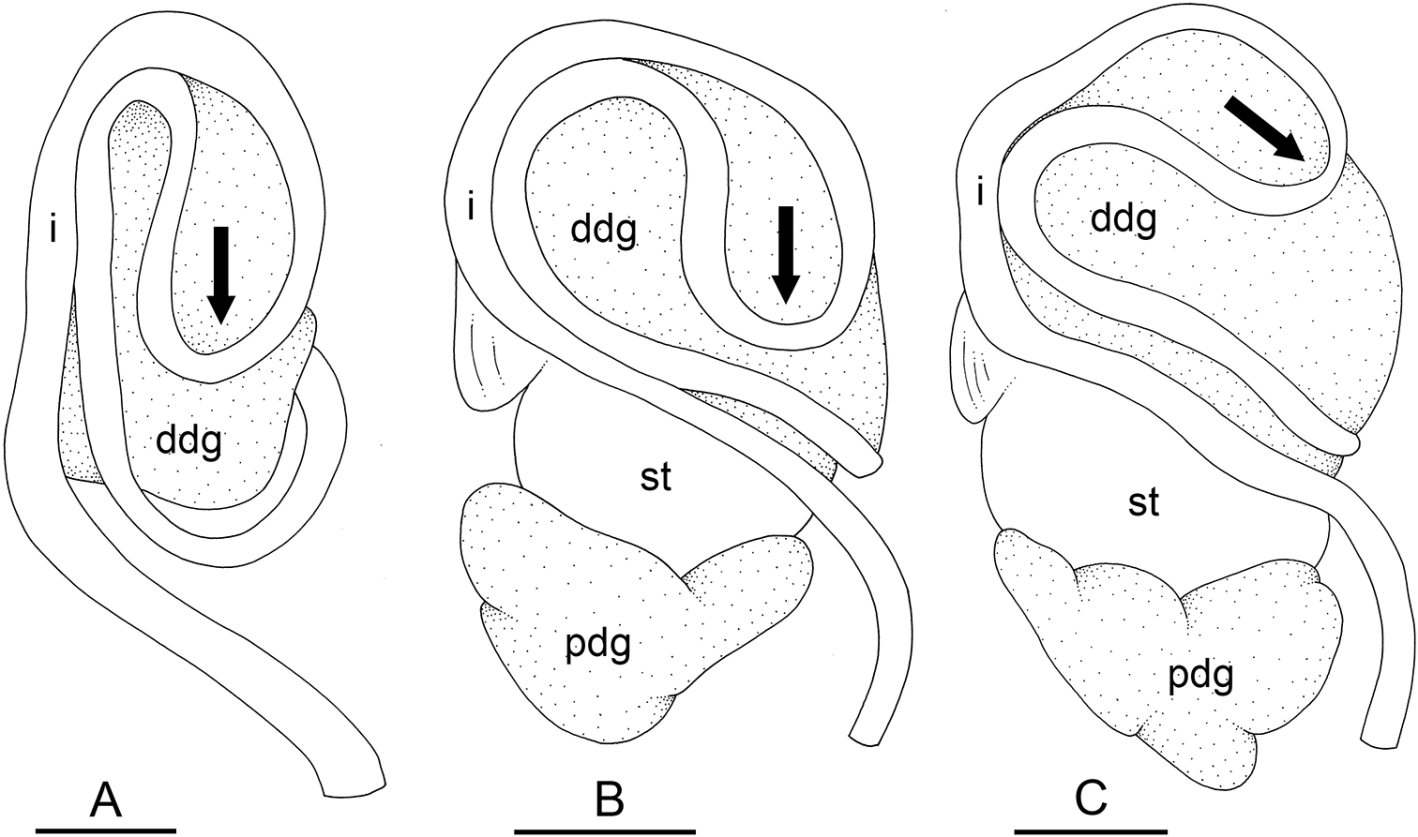
Digestive system, dorsal view, *Peronia verruculata* (unit #5). The arrow indicates the orientation of the transitional loop **A** lectotype, *Scaphis gravieri*, Mayotte (MNHN-IM-2000-33695) **B** Madagascar [3144] (MNHN-IM-2019-1611) **C** Madagascar [3231] (MNHN-IM-2019-1610). Scale bars: 2 mm (**A**), 4 mm (**B**), 5 mm (**C**). Abbreviations: ddg dorsal digestive gland, i intestine, pdg posterior digestive gland, st stomach.

**Figure 86.**
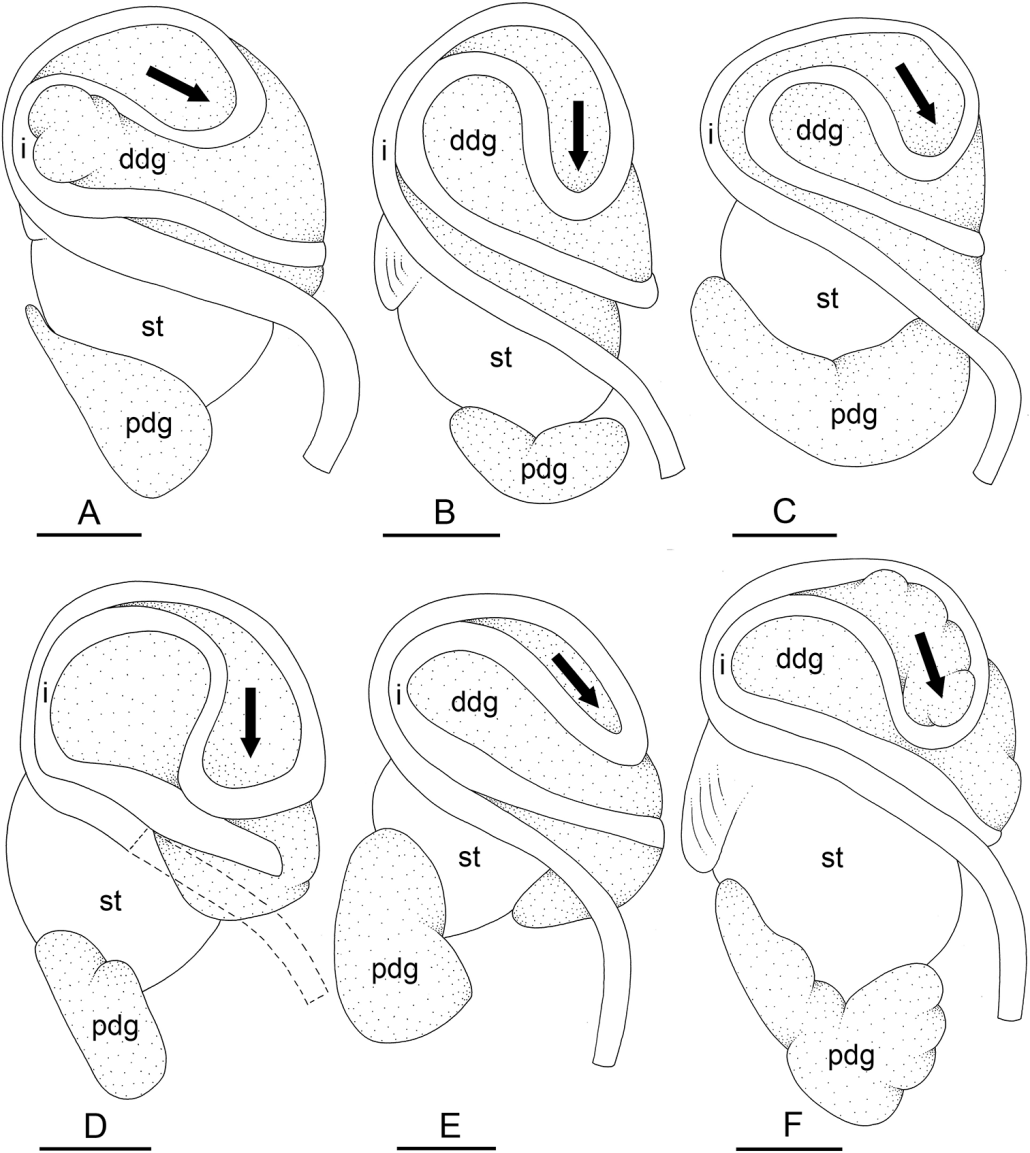
Digestive system, dorsal view, *Peronia verruculata* (Red Sea). The arrow indicates the orientation of the transitional loop **A** lectotype, *Onchidium verruculatum*, Red Sea (MNHN-IM-2000-22941) **B** lectotype, *Peronia anomala*, Red Sea (MNHN-IM-2000-33678) **C** lectotype, *Onchidium durum*, Red Sea (MNHN-IM-2000-33698) **D** paralectotype, *Paraperonia gondwanae*, Red Sea (MNHN-IM-2000-33684) **E** paralectotype, *Paraperonia gondwanae*, locality unknown (MNHN-IM-2000-33688) **F** Red Sea, spm #1 (ZMH 27472/4). Scale bars: 5 mm (**A, D**), 4 mm (**B, E**), 3 mm (**C, F**). Abbreviations: ddg dorsal digestive gland, i intestine, pdg posterior digestive gland, st stomach.

**Figure 87.**
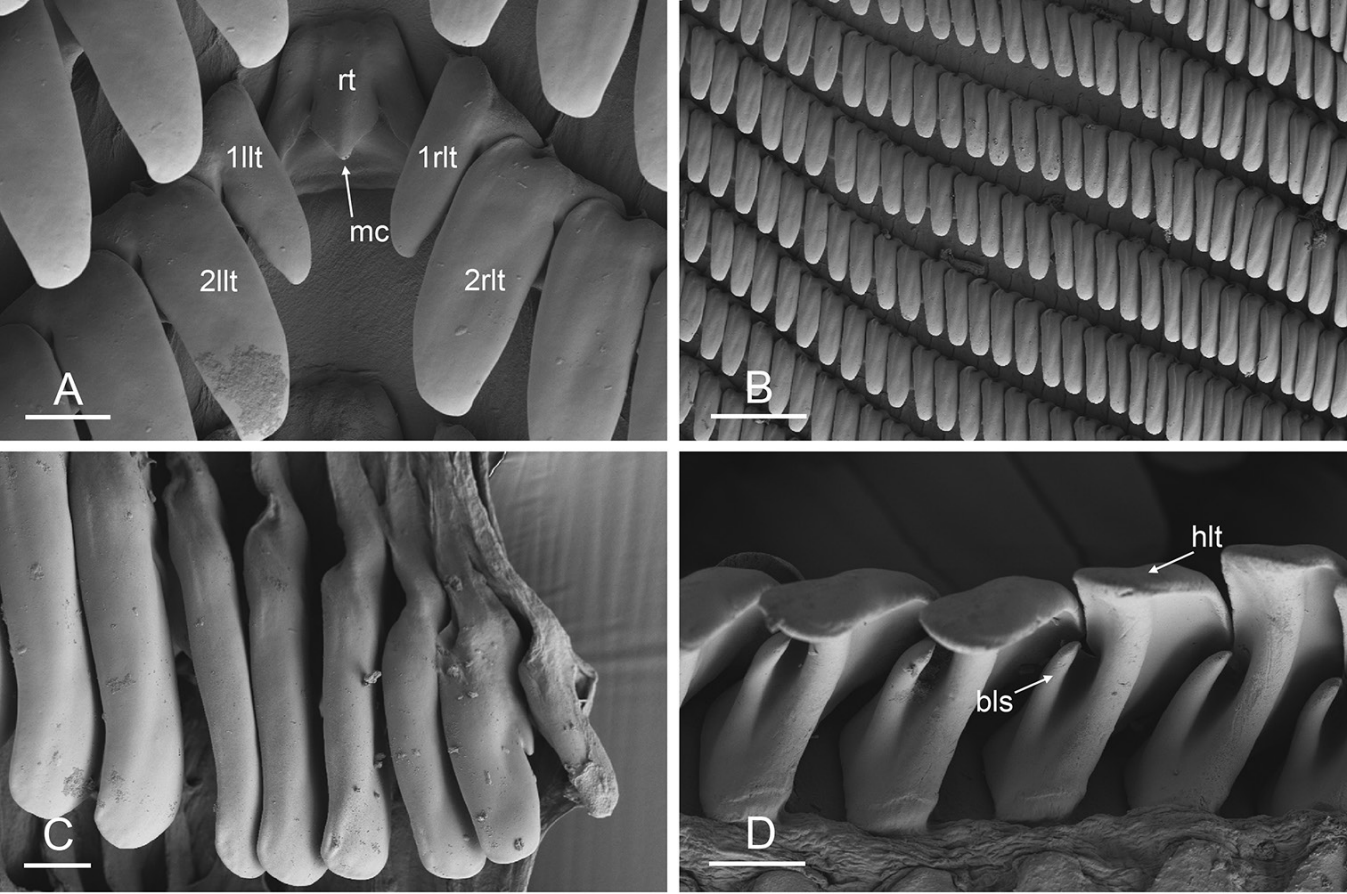
Radula, Peronia verruculata (unit #1), Vanuatu [5481] (MNHN-IM-2013-62393) **A** rachidian and innermost lateral teeth **B** lateral teeth **C** outermost lateral teeth **D** lateral teeth, frontal view. Scale bars: 20 μm (**A**), 100 μm (**B**), 10 μm (**C, D**). Abbreviations: 1llt first left lateral tooth, 1rlt first right lateral tooth, 2llt second left lateral tooth, 2rlt second right lateral tooth, bls basal lateral spine, hlt hook of a lateral tooth, mc median cusp, rt rachidian tooth.

**Figure 88.**
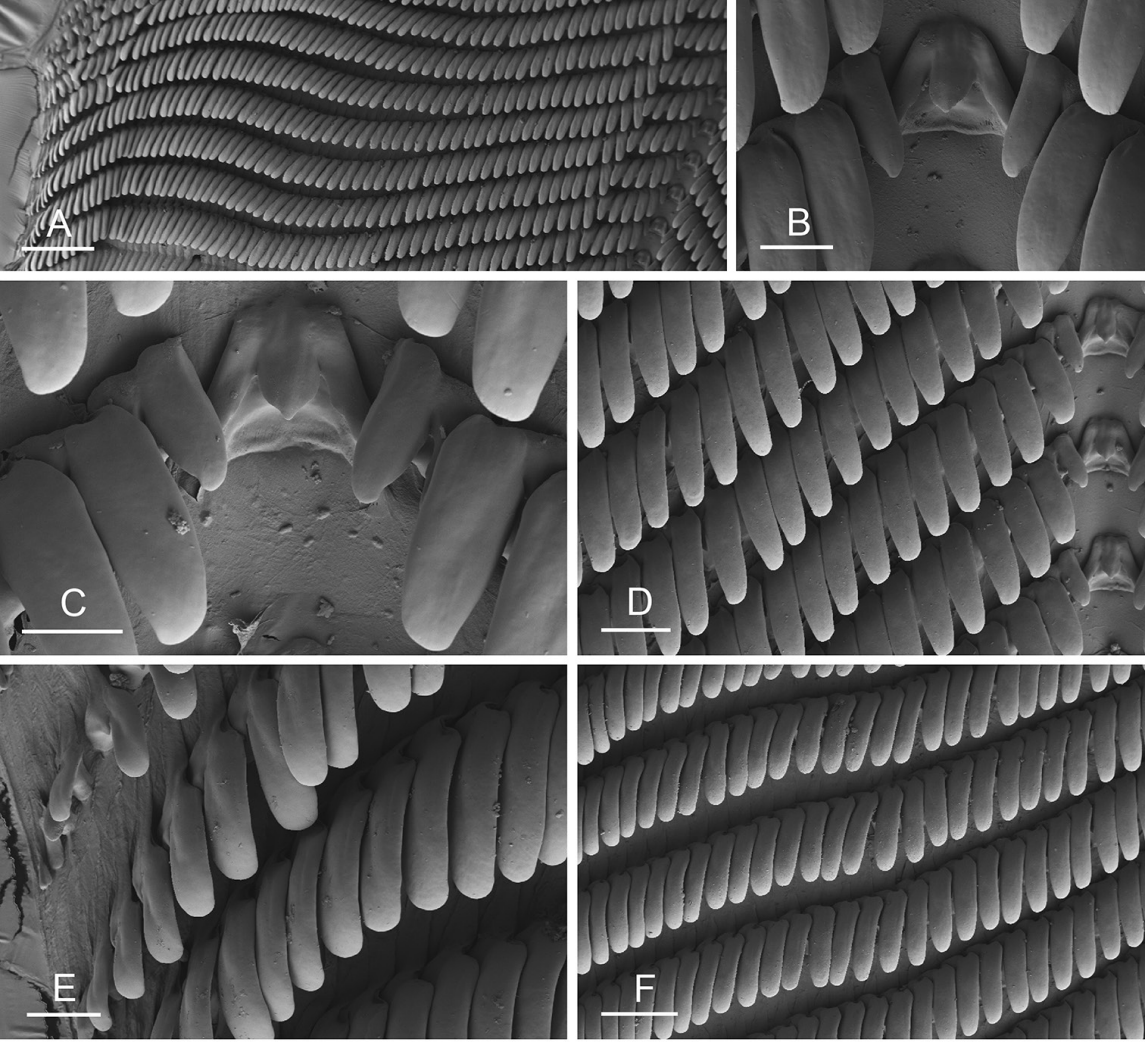
Radula, *Peronia verruculata* (unit #1), Indonesia **A, B** Seram [2870] (UMIZ 00169) **C–F** Lombok [2987] (UMIZ 00168) **A** left half rows of teeth **B** rachidian and innermost lateral teeth **C** rachidian and innermost lateral teeth **D** rachidian and lateral teeth **E** outermost lateral teeth **F** lateral teeth. Scale bars: 200 μm (**A**), 30 μm (**B, C**), 60 μm (**D**), 40 μm (**E**), 100 μm (**F**).

**Figure 89.**
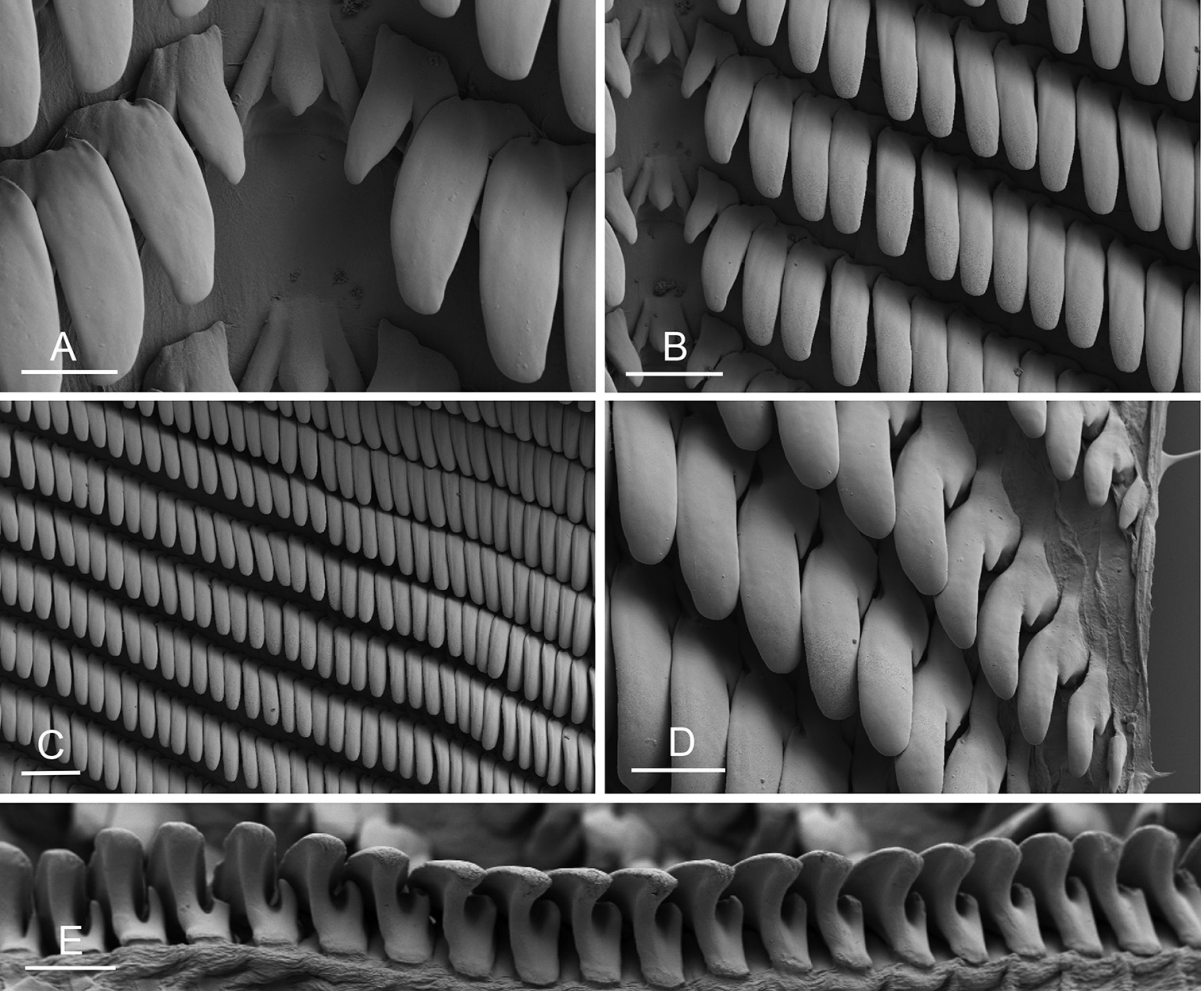
Radula, *Peronia verruculata* (unit #2), Indonesia, Sumatra **A–D** [1795] (UMIZ 00180) **E** [1746] (UMIZ 00178) **A** rachidian and innermost lateral teeth **B** rachidian and innermost lateral teeth **C** lateral teeth **D** outermost lateral teeth **E** lateral teeth, frontal view. Scale bars: 40 μm (**A, D**), 80 μm (**B**), 100 μm (**C**), 60 μm (**E**).

**Figure 90.**
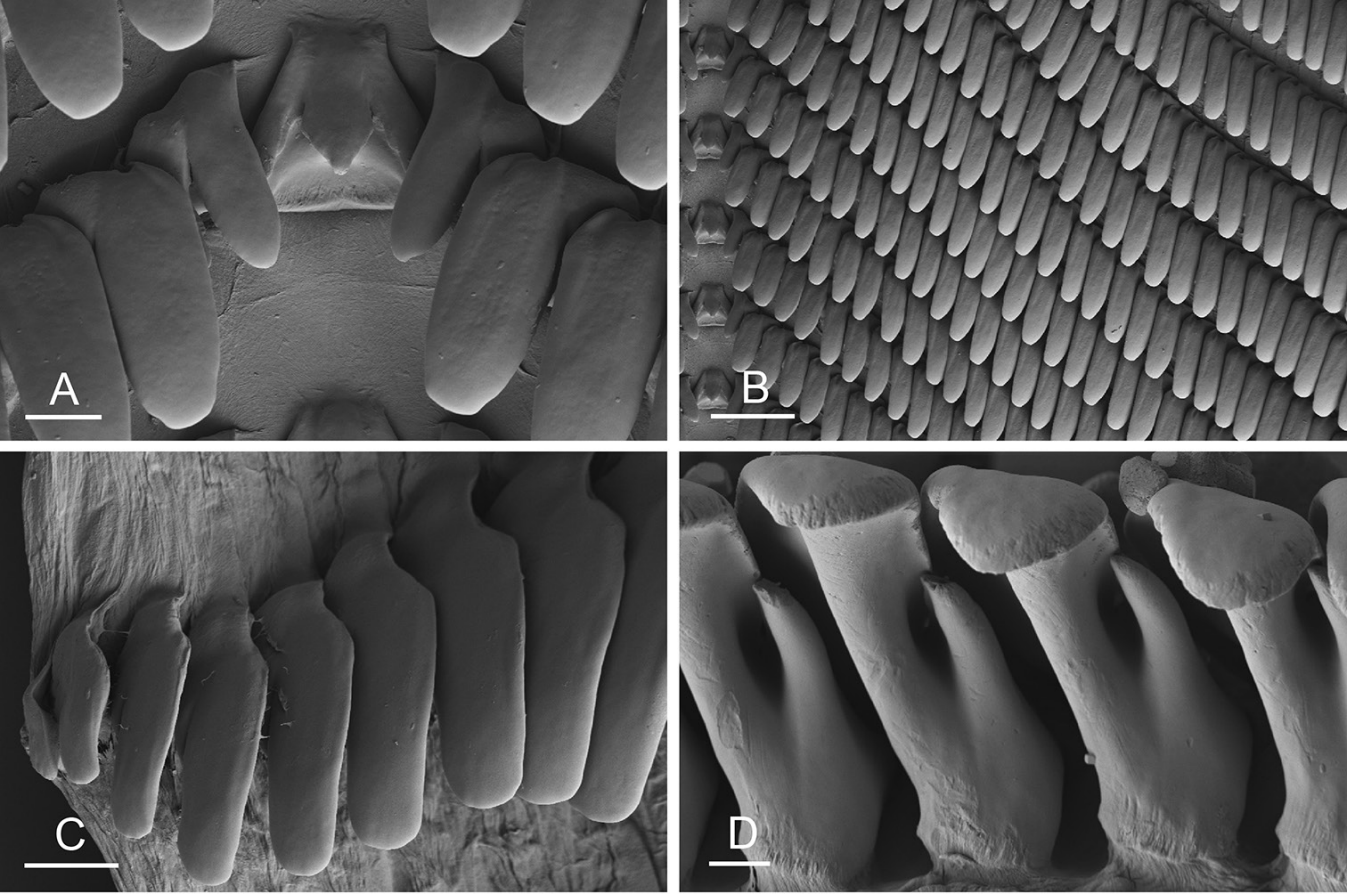
Radula, *Peronia verruculata* (unit #3), Peninsular Malaysia **A–C** [976] (USMMC 00051) **D** [975] (USMMC 00064) **A** rachidian and innermost lateral teeth **B** rachidian and lateral teeth **C** outermost lateral teeth **D** lateral teeth, frontal view. Scale bars: 20 μm (**A, C**), 100 μm (**B**), 10 μm (**D**).

**Figure 91.**
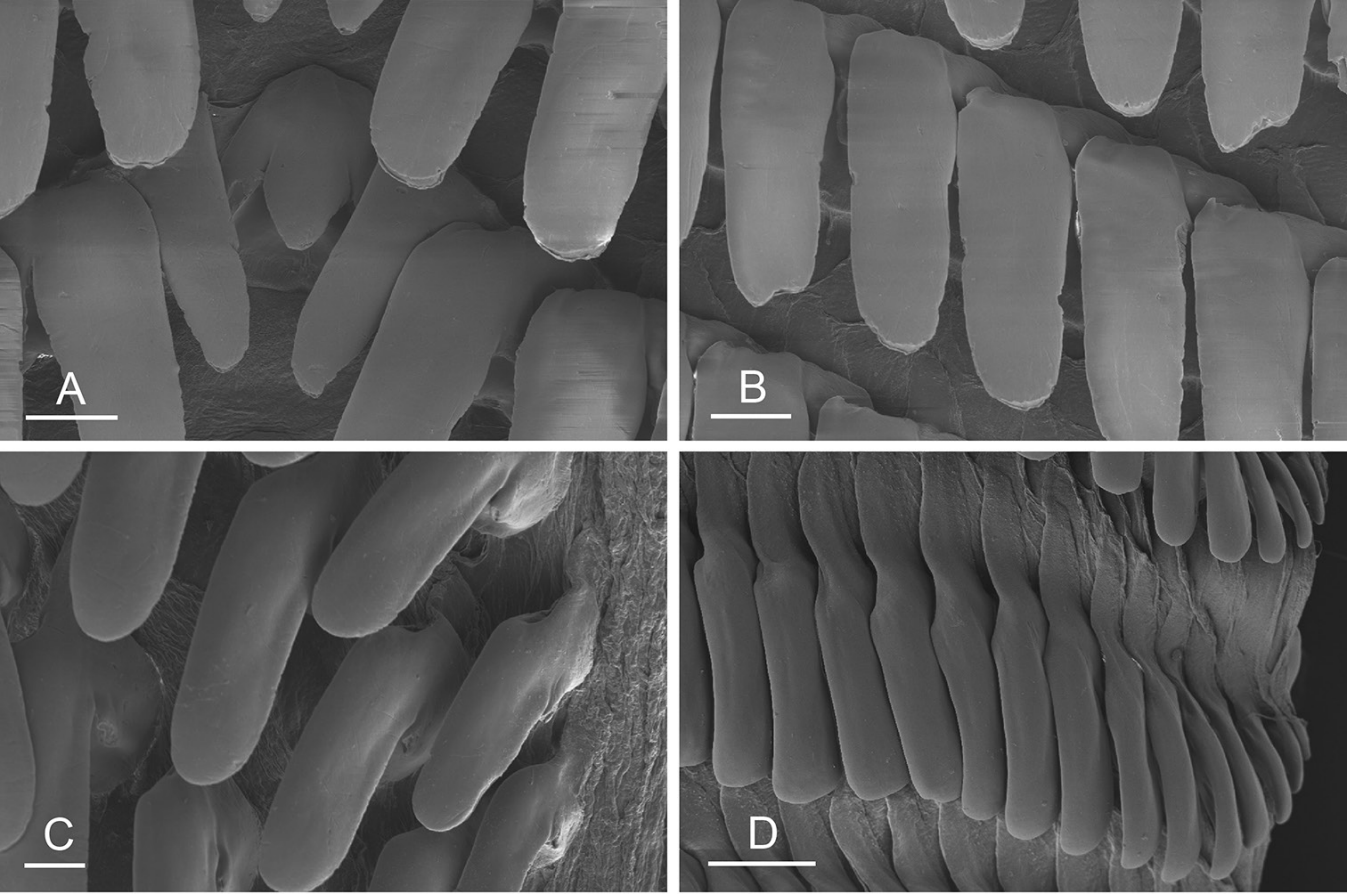
Radula, *Peronia verruculata* (unit #4), Pakistan **A–C** [6164] (MNHN-IM-2019-1384) **D** [6165] (MNHN-IM-2019-1385) **A** rachidian and innermost lateral teeth **B** lateral teeth **C** outermost lateral teeth **D** outermost lateral teeth. Scale bars: 20 μm (**A, B**), 10 μm (**C**), 40 μm (**D**).

**Figure 92.**
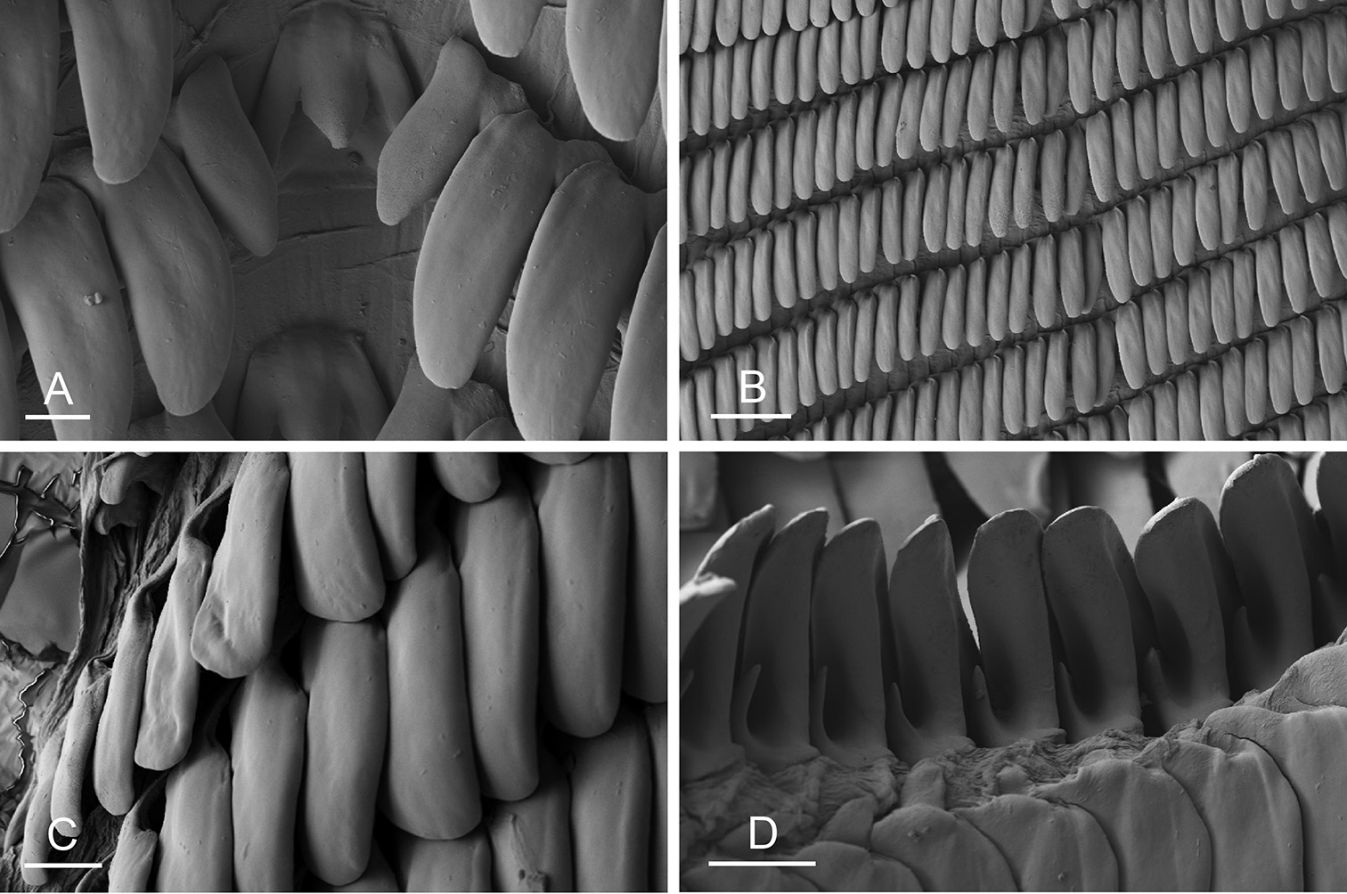
Radula, *Peronia verruculata* (unit #5), Madagascar **A–C** [3231] (MNHN-IM-2019-1610) **D** Mozambique [5510] (MNHN-IM-2013-62398) **A** rachidian and innermost lateral teeth **B** lateral teeth **C** outermost lateral teeth **D** lateral teeth, frontal view. Scale bars: 20 μm (**A, C**), 100 μm (**B**), 30 μm (**D**).

**Figure 93.**
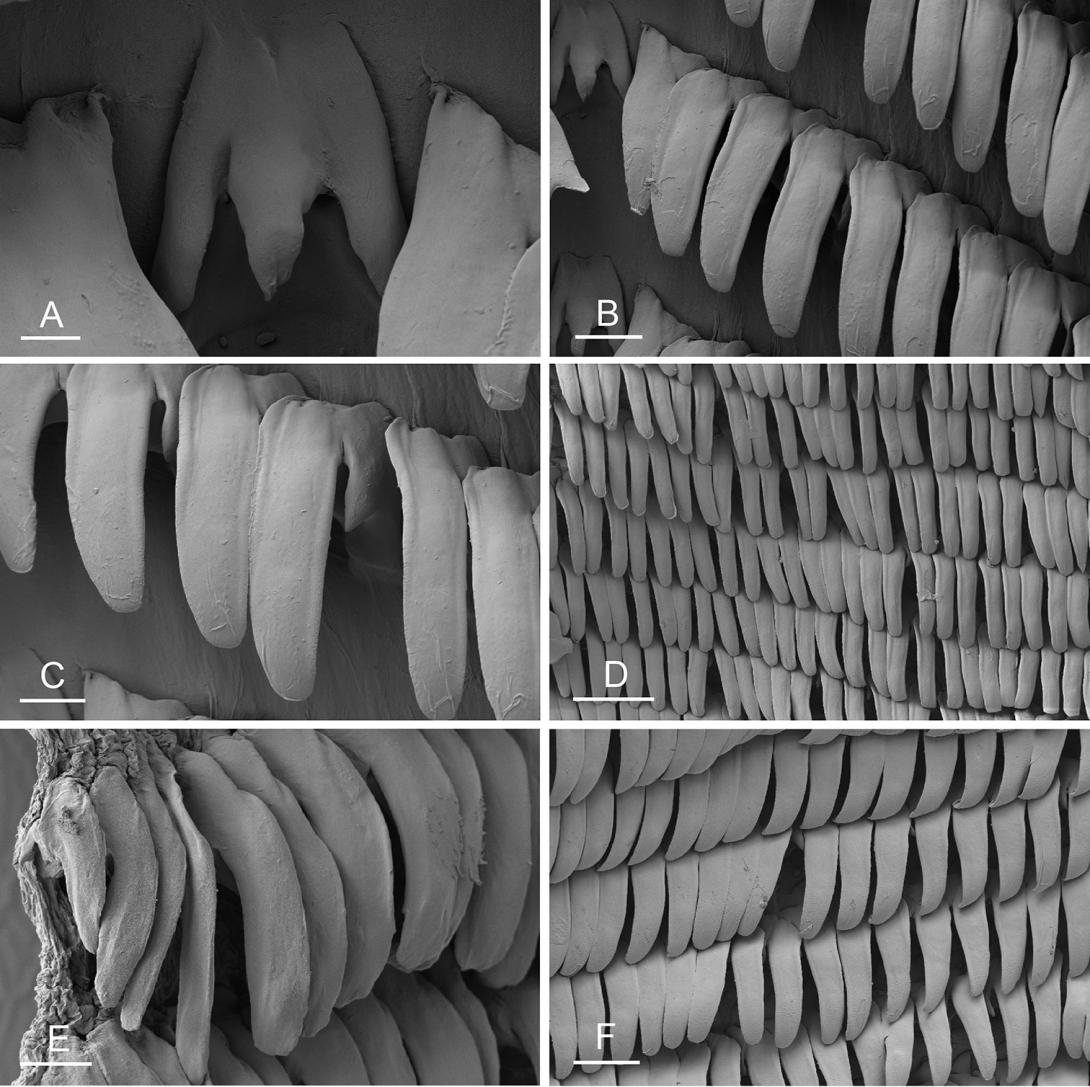
Radula, *Peronia verruculata*, Red Sea, spm #3 (ZMH 27472/4) **A** rachidian tooth **B** rachidian and innermost lateral teeth **C** lateral teeth **D** lateral teeth **E** outermost lateral teeth **F** lateral teeth. Scale bars: 10 μm (**A**), 30 μm (**B**), 20 μm (**C, E**), 100 μm (**D**), 60 μm (**F**).

(Figs 79–81, 82A, 83A, 84A, B, 85–93). There are no jaws. The left and right salivary glands, heavily branched, join the buccal mass dorsally, on either side of the esophagus. The esophagus is narrow and straight, with thin internal folds. The esophagus enters the stomach anteriorly (Fig. 79). Only a portion of the posterior aspect of the stomach can be seen in dorsal view because it is partly covered by the lobes of the digestive gland. The dorsal lobe is mainly on the right. The left, lateral lobe is mainly ventral. The posterior lobe covers the posterior aspect of the stomach. The stomach is a U-shaped sac divided into four chambers (Fig. 79). The first chamber, which receives the esophagus, is delimited by thin tissue, and receives the ducts of the dorsal and lateral lobes of the digestive gland. It is internally smooth (with no ridges). The second, posterior chamber, delimited by thick muscular tissue (which takes most of the space inside), receives the duct of the posterior lobe of the digestive gland. The third, funnel-shaped chamber is delimited by thin tissue with high leaflet-like ridges internally. The fourth chamber is continuous and externally similar to the third, but it bears only low, thin ridges internally.

The intestine is long and narrow. Intestinal loops were checked in every specimen listed in the material examined: the intestinal loops are of type I with a transitional loop oriented between 3 and 6 o’clock (Figs 79–81, 82A, 83A, 84A, B, 85, 86). There is no rectal gland.

The radula is in between two large postero-lateral muscular masses (Figs 87–93). Each radular row contains a rachidian tooth and two half rows of lateral teeth of similar size and shape. Examples of radular formulae are presented in Table 5. The rachidian teeth are unicuspid (Fig. 87A): the median cusp is always present; there are no conspicuous cusps on the lateral sides of the base of the rachidian tooth. The median cusp of the rachidian teeth is approximately 40 μm long. The lateral aspect of the base of the rachidian teeth is straight. The half rows of lateral teeth form an angle of 45° with the rachidian axis. Except for the few innermost and few outermost lateral teeth, the size and shape of the lateral teeth do not vary along the half row, nor do they vary among half rows. The lateral teeth are unicuspid with a flattened and curved hook (approximately from 80 to 120 μm long) with a rounded tip, but there is also a pointed spine on the outer lateral expansion of the base, or basal lateral spine (Fig. 87D). In most cases, the basal lateral spine cannot be observed because it is hidden below the hook of the next, outer lateral tooth. It can only be observed when the teeth are not too close (such as in the innermost and outermost regions) or when teeth are placed in an unusual position. The inner and outer lateral aspects of the hook of the lateral teeth are straight (i.e., not wavy and not with any protuberance).

###### Reproductive system

(Figs 82B, C, 83B, C, 84C, D, 94–109). Sexual maturity is correlated with animal length. Mature individuals have large female organs (with a large female gland mass) and fully developed male copulatory parts. The smallest, immature individuals may have inconspicuous (or no) female organs and rudimentary anterior male parts.

**Figure 94.**
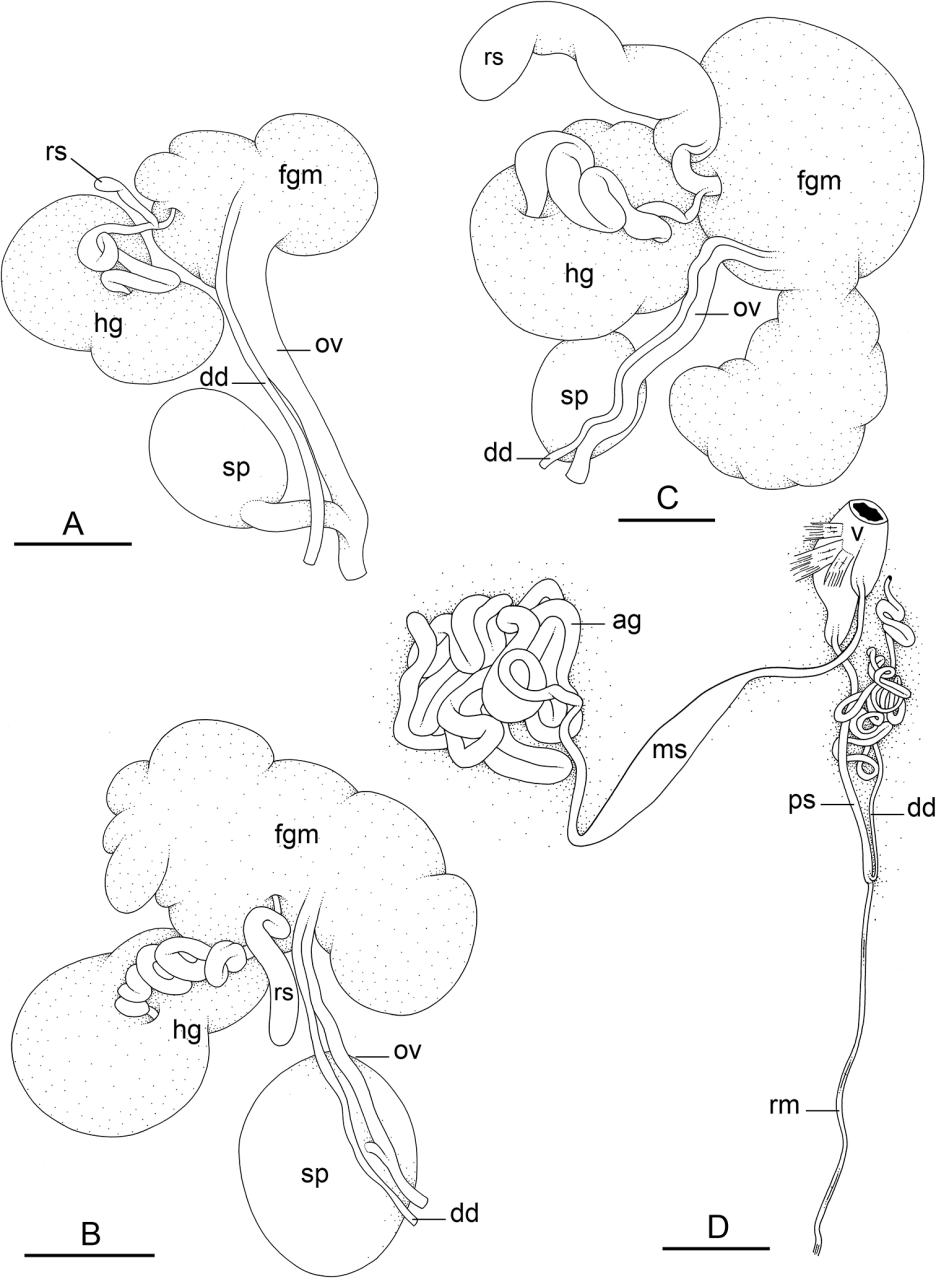
Reproductive system, *Peronia verruculata* (unit #1) **A** immature, posterior, hermaphroditic (female) reproductive system, Australia, Queensland [2622] (MTQ) **B** posterior, hermaphroditic (female) reproductive system, Vietnam [5621] (ITBZC IM 00021) **C** posterior, hermaphroditic (female) reproductive system, Indonesia, Sulawesi [2127] (UMIZ 00170) **D** anterior, male, copulatory apparatus, same as C. Scale bars: 1 mm (**A**), 4 mm (**B**), 3 mm (**C**), 5 mm (**D**). Abbreviations: ag accessory penial gland, dd deferent duct, ddg dorsal digestive gland, fgm female gland mass, hg hermaphroditic gland, i intestine, ms muscular sac, ov oviduct, pdg posterior digestive gland, ps penial sheath, rm retractor muscle, rs receptaculum seminis, sp spermatheca, st stomach, v vestibule.

**Figure 95.**
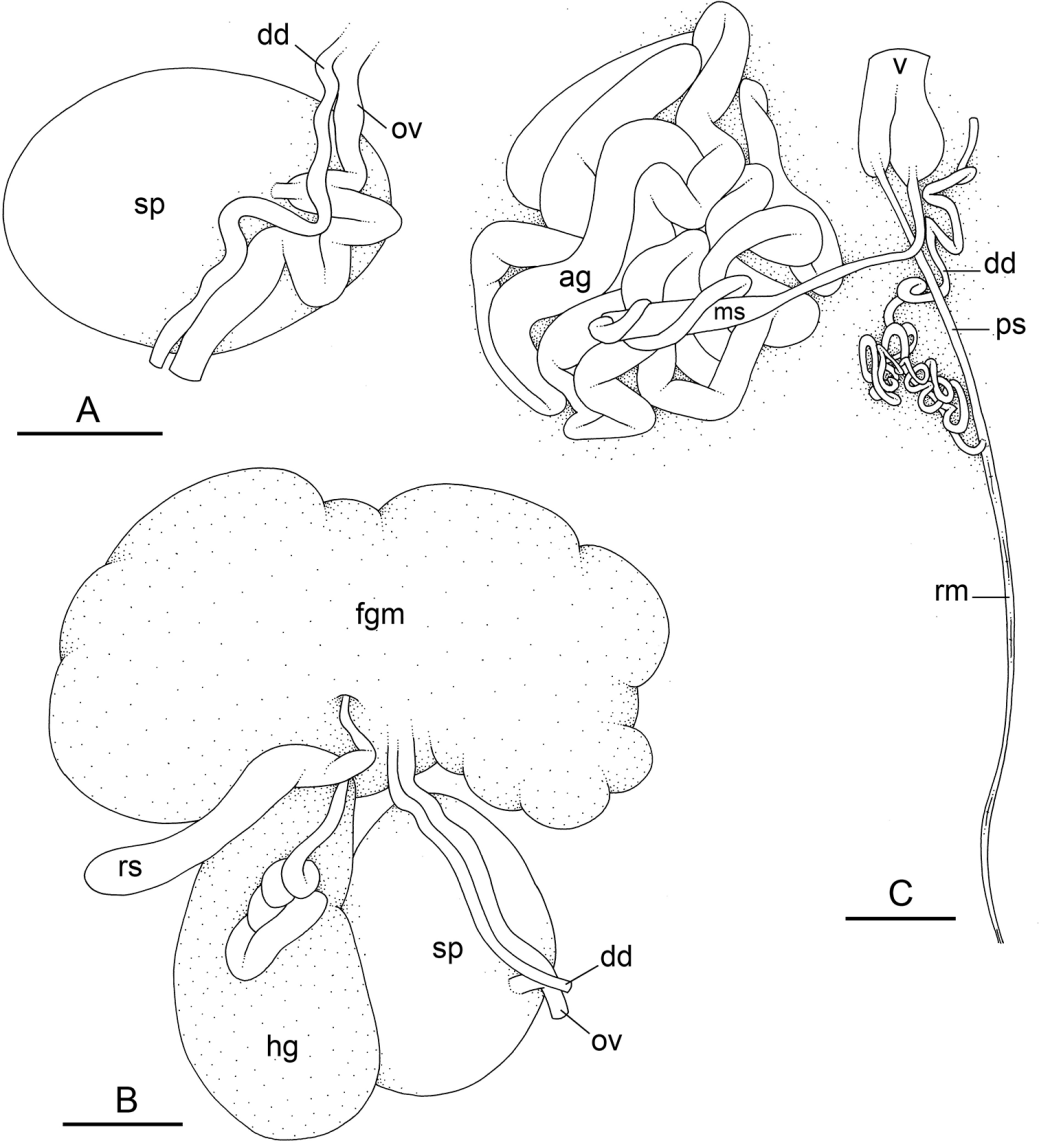
Reproductive system, *Peronia verruculata* (unit #5), Madagascar **A** posterior, hermaphroditic (female) reproductive system, only the spermatheca, the deferent duct, and the oviduct, [3143] (MNHN-IM-2019-1611) **B** posterior, hermaphroditic (female) reproductive system, [3231] (MNHN-IM-2019-1610) **C** anterior, male, copulatory apparatus, same as B. Scale bars: 5 mm (**A**), 3 mm (**B**), 4 mm (**C**). Abbreviations: ag accessory penial gland, dd deferent duct, fgm female gland mass, hg hermaphroditic gland, ms muscular sac, ov oviduct, ps penial sheath, rm retractor muscle, rs receptaculum seminis, sp spermatheca, v vestibule.

**Figure 96.**
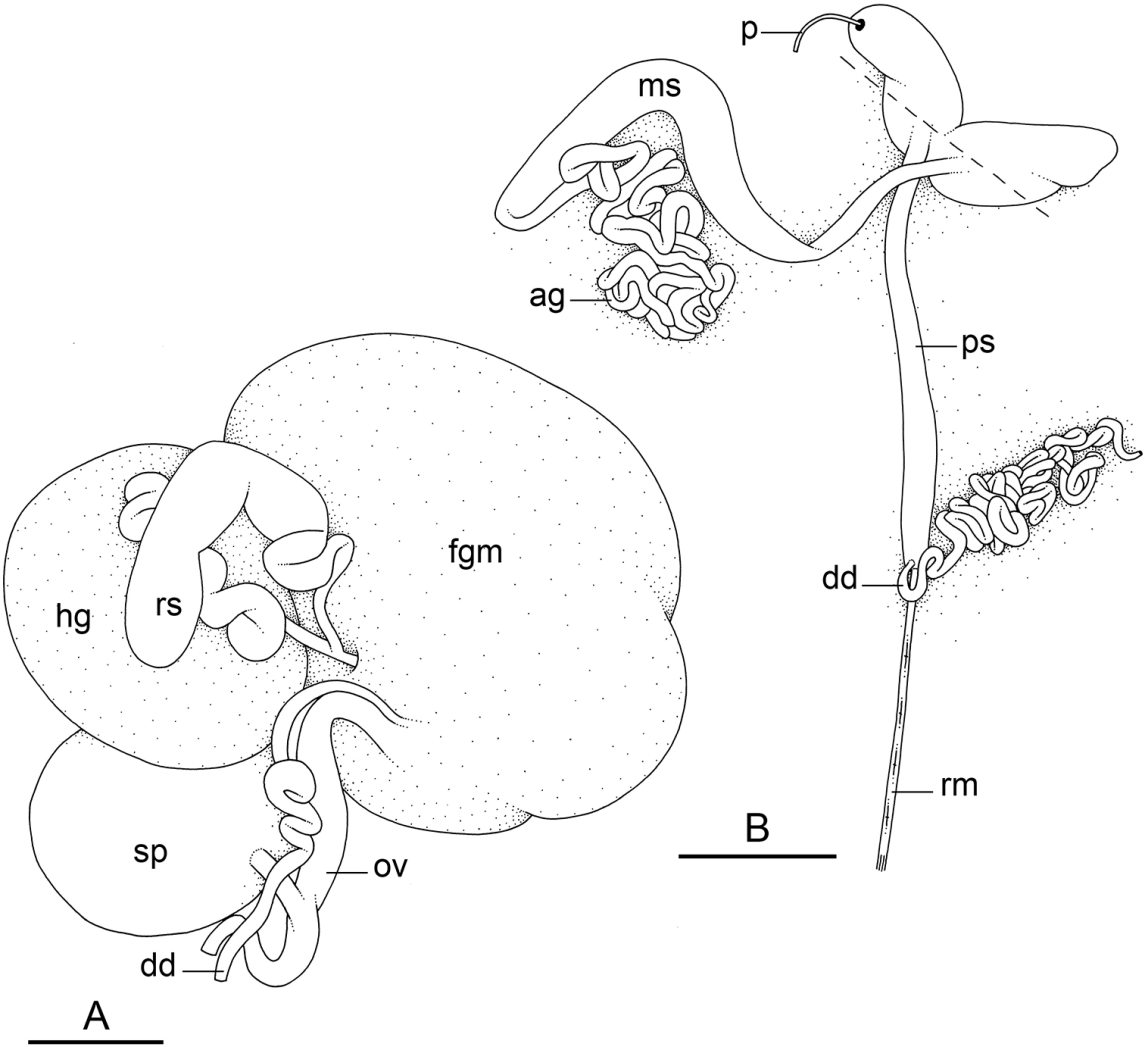
Reproductive system, *Peronia verruculata*, Red Sea, spm #1 (ZMH 27472/4) **A** posterior, hermaphroditic (female) reproductive system **B** anterior, male, copulatory apparatus. Scale bars: 2 mm (**A**), 4 mm (**B**). Abbreviations: Abbreviations: ag accessory penial gland, dd deferent duct, fgm female gland mass, hg hermaphroditic gland, ms muscular sac, ov oviduct, p penis, ps penial sheath, rm retractor muscle, rs receptaculum seminis, sp spermatheca.

The female organs are located (with some male parts) at the posterior end of the visceral cavity (Figs 82B, 83B, 84C, 94A–C, 95A, B, 96A). The hermaphroditic gland is a single mass, joining the spermoviduct through the hermaphroditic duct (which conveys the eggs and the autosperm). There is a narrow, elongated receptaculum seminalis (caecum) along the hermaphroditic duct. The female gland mass contains various glands (mucus and albumen) which can hardly be separated by dissection and of which the exact connections remain uncertain. The hermaphroditic duct becomes the spermoviduct (which conveys eggs, exosperm, and autosperm). Proximally, the spermoviduct is not divided (at least externally) and is embedded within the female gland mass. Distally, the spermoviduct branches into the straight deferent duct (which conveys the autosperm up to the anterior region, running through the body wall) and the oviduct. The free oviduct conveys the eggs up to the female opening and the exosperm from the female opening up to the fertilization chamber. The large, spherical-ovate spermatheca connects to the oviduct through a short duct. The oviduct is narrow and straight. There is no vaginal gland.

The male anterior organs consist of the penial complex (penis, penial sheath, vestibule, deferent duct, retractor muscle) and the accessory penial gland (Figs 82C, 83C, 84D, 94D, 95C, 96B, 97–109). The penial complex and the accessory penial gland share the same vestibule and the same anterior male opening.

**Figure 97.**
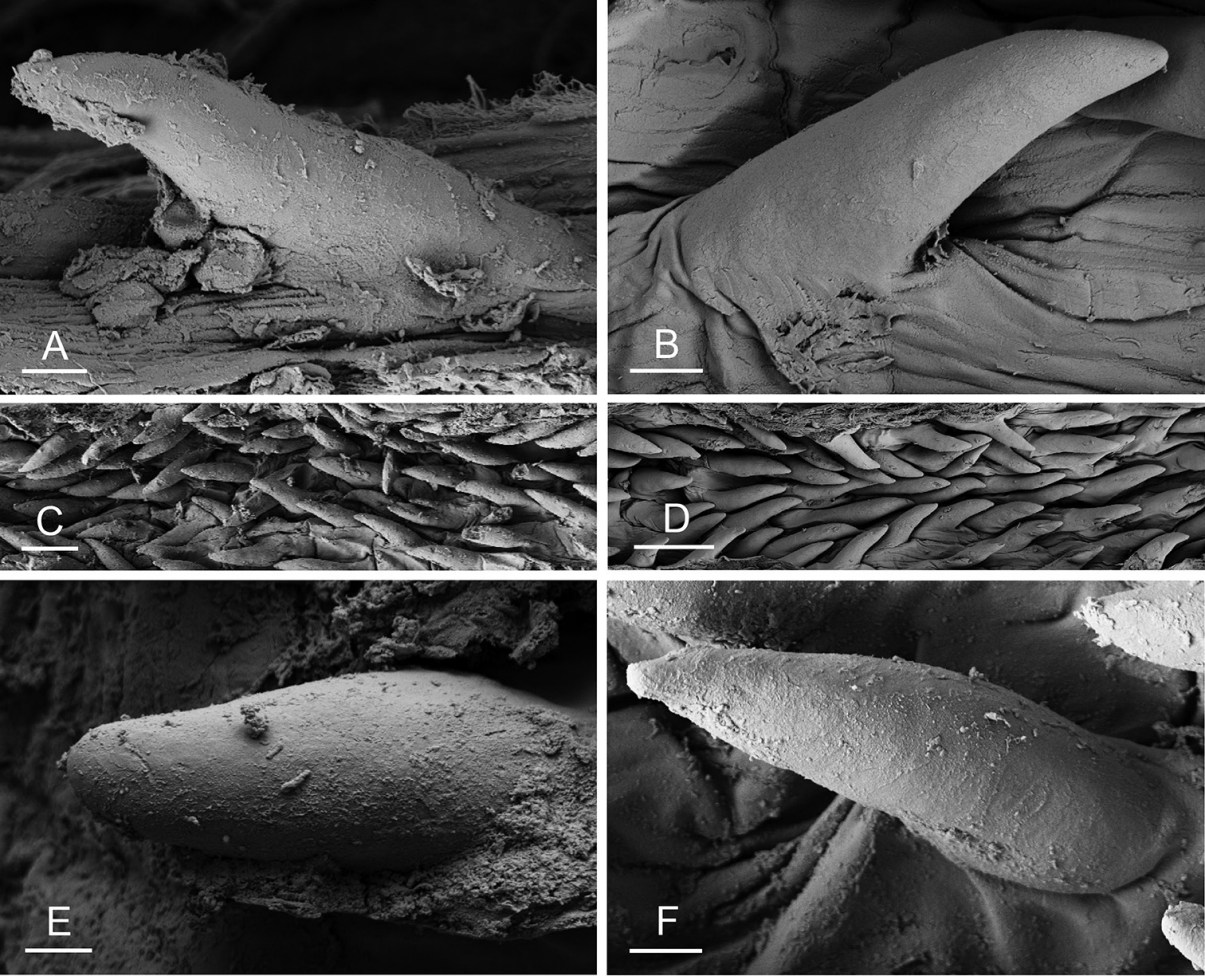
Penial hooks, *Peronia verruculata* (unit #1) **A** Australia, Queensland [2622] (MTQ) **B** Indonesia, Halmahera [5068] (UMIZ 00166) **C** Papua New Guinea, Madang [5469] (MNHN-IM-2013-12010) **D** same as **B**; **E** Indonesia, Seram [2870] (UMIZ 00169) **F** same as **C**. Scale bars: 4 μm (**A, B**), 20 μm (**C**), 30 μm (**D**), 3 μm (**E, F**).

**Figure 98.**
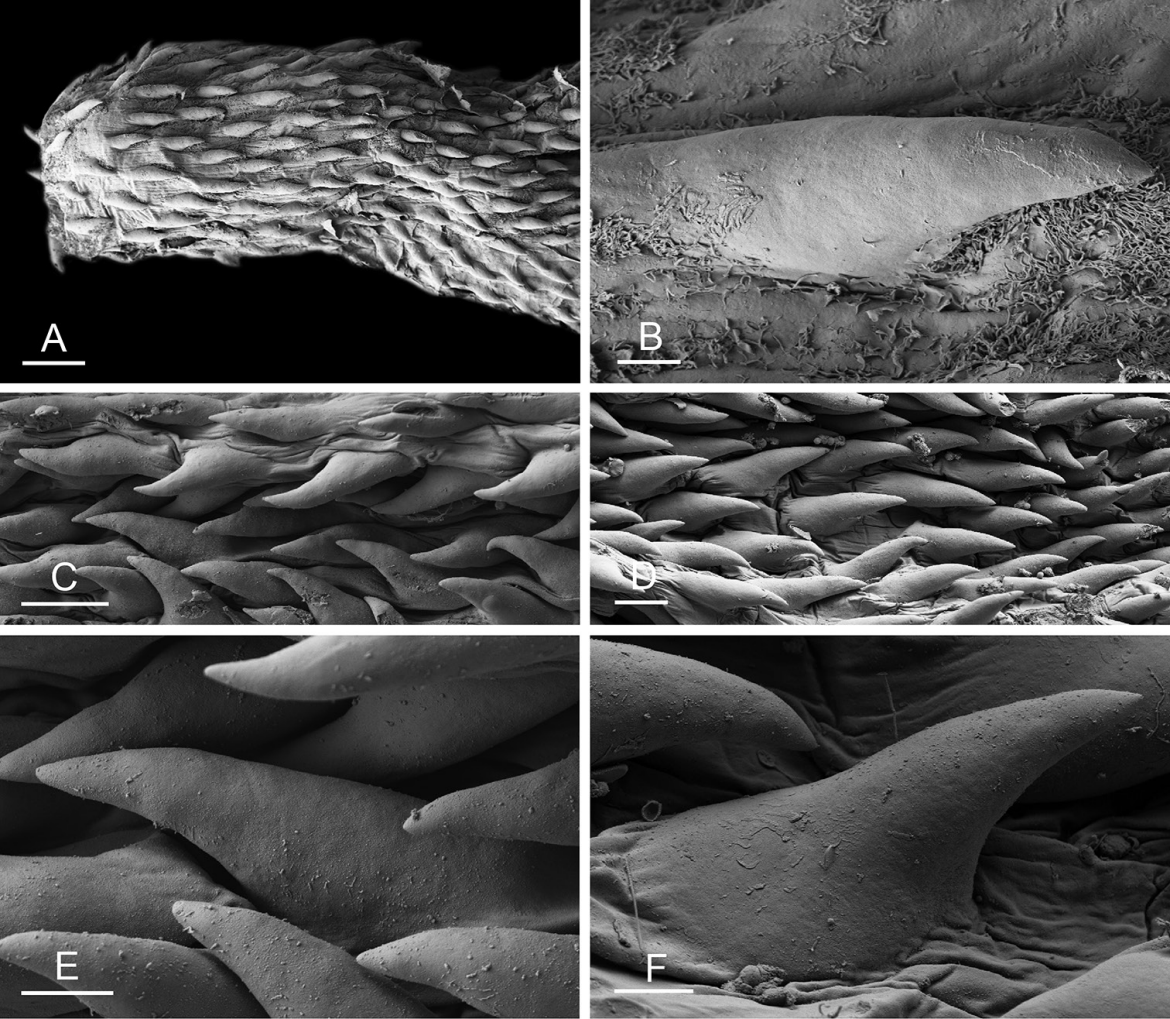
Penis and penial hooks, *Peronia verruculata* (unit #2), Indonesia, Sumatra **A, B** [1746] (UMIZ 00178) **C, E** [1795] (UMIZ 00180) **D, F** [1797] (UMIZ 00180). Scale bars: 60 μm (**A**), 6 μm (**B, E, F)**, 20 μm (**C, D**).

**Figure 99.**
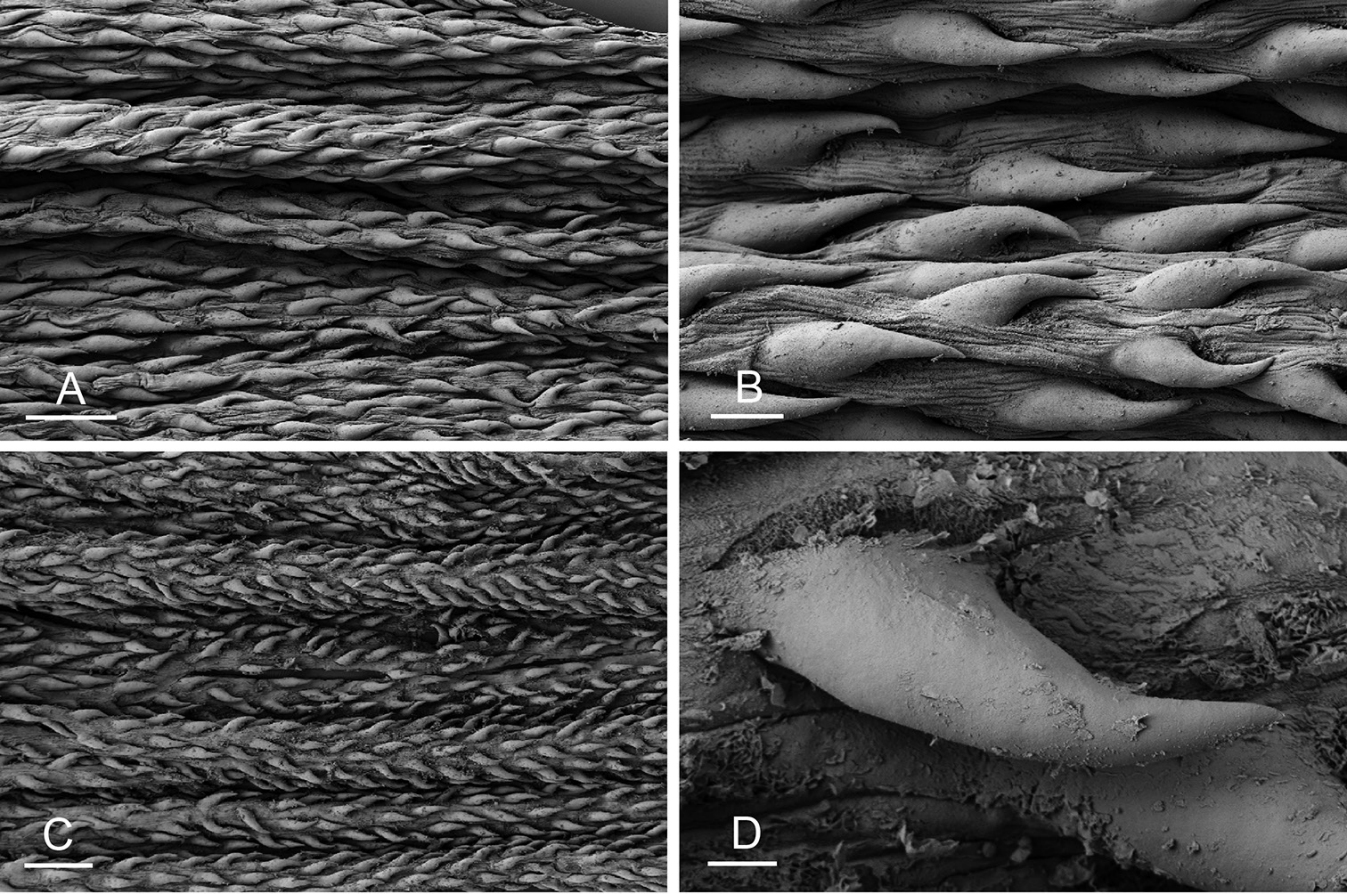
Penial hooks, *Peronia verruculata* (unit #3), Peninsular Malaysia **A, B** [2547] (USMMC 00065) **C, D** [975] (USMMC 00064). Scale bars: 40 μm (**A, C**), 20 μm (**B**), 6 μm (**D**).

**Figure 100.**
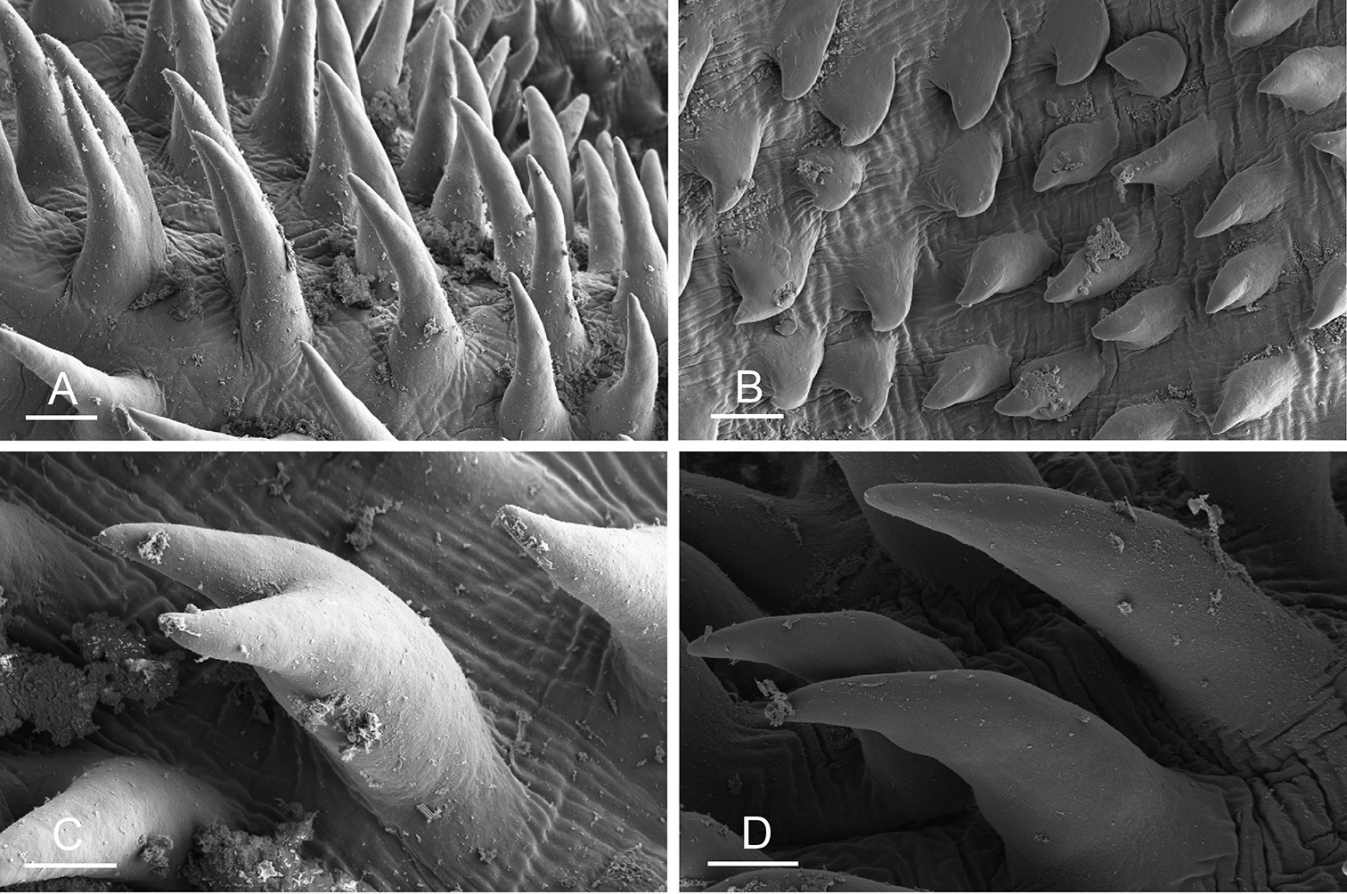
Penial hooks, *Peronia verruculata* (unit #4), Pakistan **A–C** [6164] (MNHN-IM-2019-1384) **D** [6165] (MNHN-IM-2019-**1385**). Scale bars: 20 μm (**A**), 10 μm (**B–D**).

**Figure 101.**
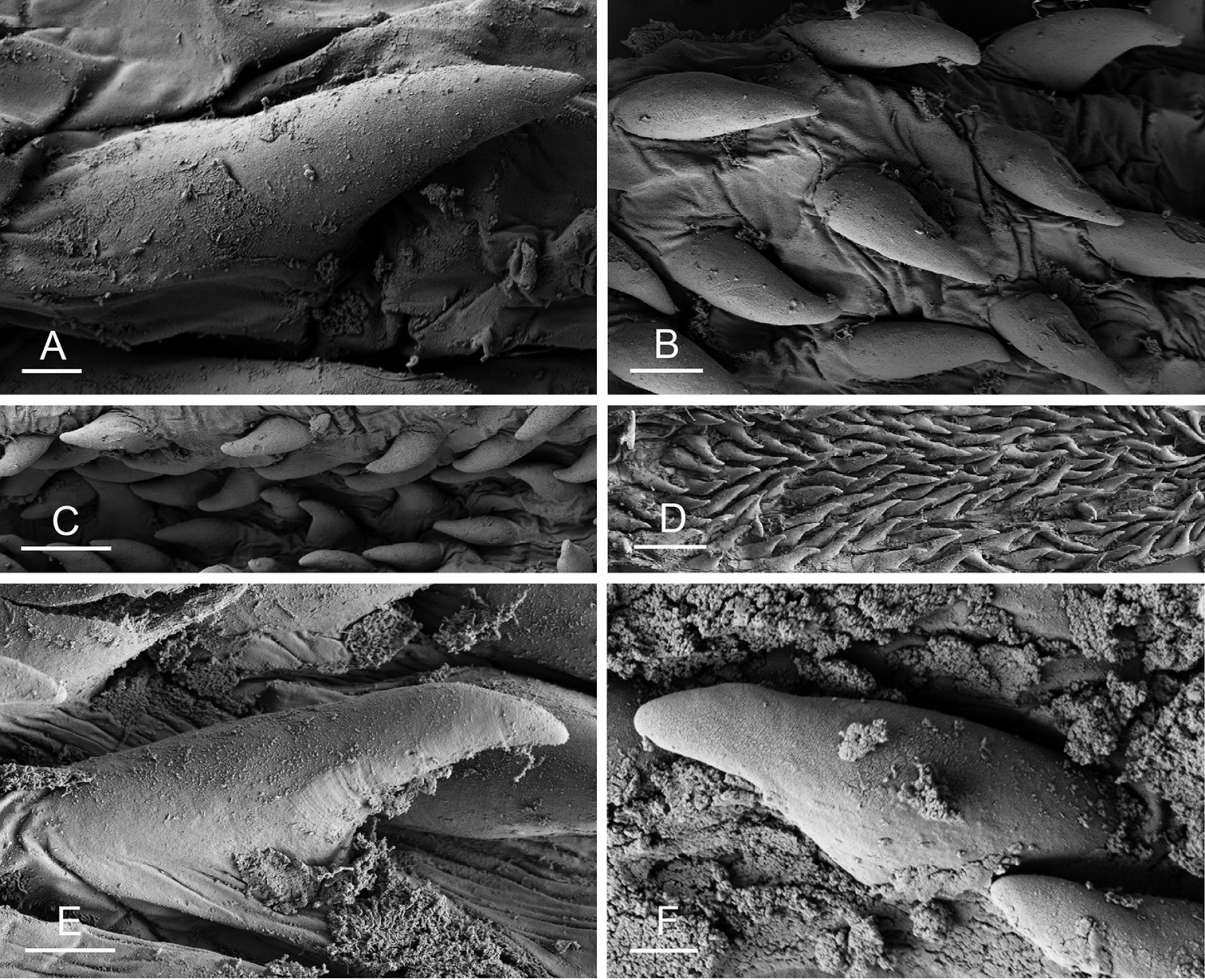
Penial hooks, *Peronia verruculata* (unit #5) **A–C** Madagascar [3144] (MNHN-IM-2019-1611) **D, E** Madagascar [3231] (MNHN-IM-2019-1610) **F** Mozambique [5510] (MNHN-IM-2013-62398). Scale bars: 6 μm (**A**), 10 μm (**B, E**), 20 μm (**C**), 100 μm (**D**), 4 μm (**F**).

**Figure 102.**
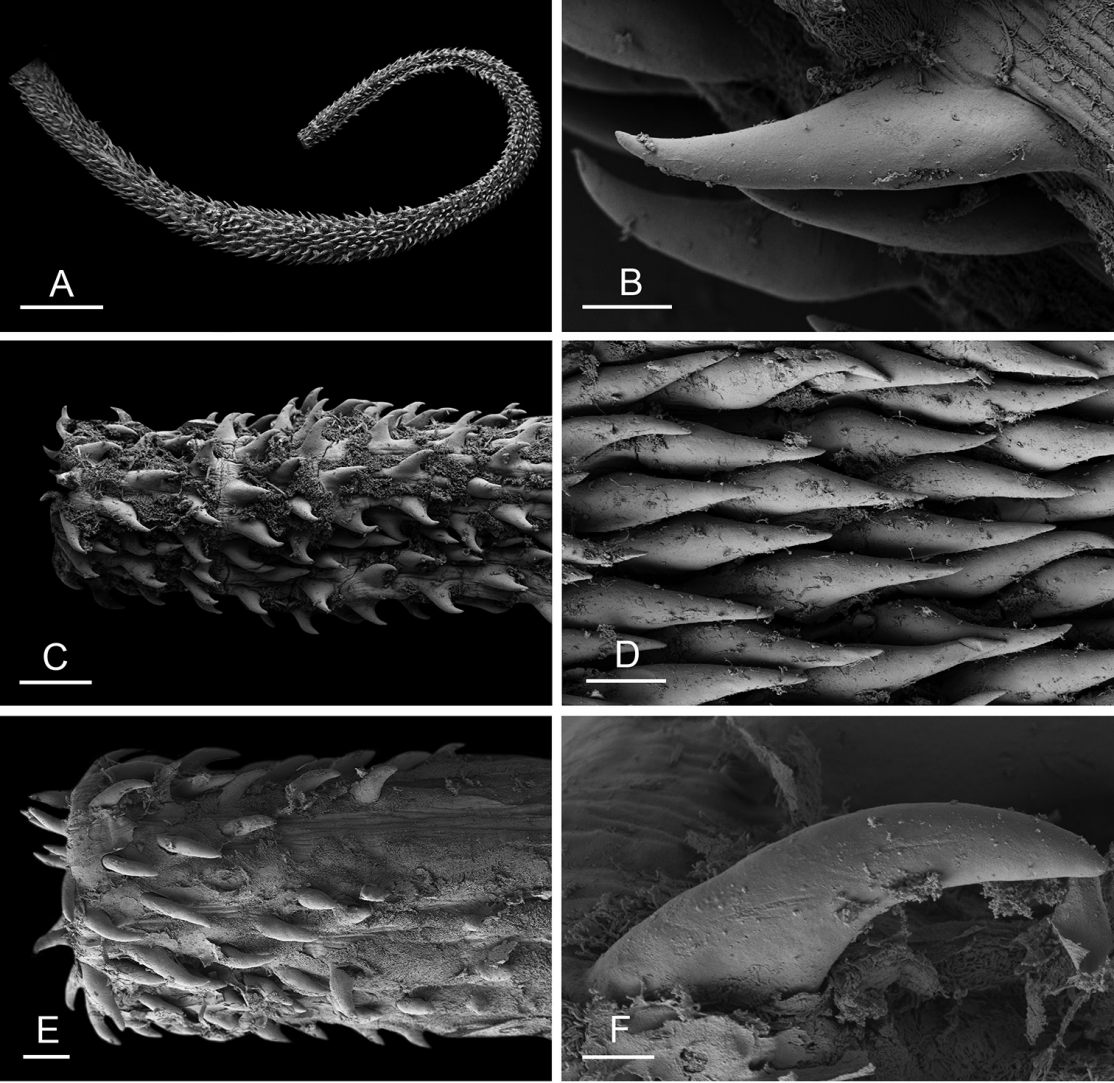
Penis and penial hooks, *Peronia verruculata*, Red Sea **A–C** spm #1 (ZMH 27472/4) **D** spm #2 (ZMH 27472/4) **E, F** spm #4 (ZMH 27472/4). Scale bars: 300 μm (**A**), 10 μm (**B**), 30 μm (**C**), 20 μm (**D, E**), 4 μm (**F**).

The penial sheath is narrow and elongated. The penial sheath protects the penis for its entire length. The beginning of the retractor muscle marks the separation between the penial sheath (and the penis inside) and the deferent duct, which is highly coiled. The retractor muscle, which can be shorter or longer than the penial sheath, inserts at the posterior end of the visceral cavity. Inside the penial sheath, the penis is a narrow, elongated, soft, hollow tube. Its distal end bears conical hooks which are less than 50 μm long in units #1 and #2, less than 55 μm long in units #5 and #6, and less than 60 μm in units #3 and #4 (Figs 97–102). When the penis is retracted inside the penial sheath, the hooks are densely packed inside the tube-like penis; during copulation, the penis is evaginated like a glove and the hooks are outside, not as densely packed. In some individuals of unit #4, a few penial hooks are exceptionally double, or two-pronged (Fig. 100C).

The accessory penial gland is a long, tube-like flagellum with a proximal dead end. The length of the flagellum of the penial gland varies among individuals but it is always highly coiled. Near its distal end (just before the hollow spine), the flagellum is enlarged into a thick muscular sac, which is less than 15 mm long in units #1 and #6 and less than 10 mm long in the other units. Distally, the flagellum ends in a hard, hollow spine protected by a sheath which opens into the vestibule.

**Figure 103.**
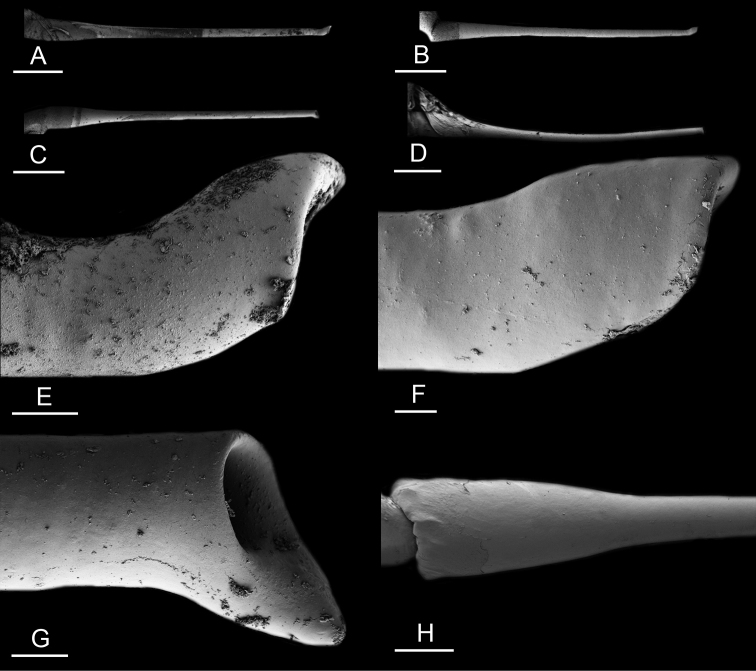
Accessory penial gland spine, *Peronia verruculata* (unit #1) **A–G** Indonesia **H** Singapore **A** Halmahera [5068] (UMIZ 00166) **B** Ambon [2729] (UMIZ 00162) **C** Lombok [2987] (UMIZ 00168) **D** Sulawesi [2127] (UMIZ 00170) **E** same as **A**; **F** same as **B**; **G** same as **C**; **H** [991] (ZRC.MOL.10497). Scale bars: 300 μm (**A–D**), 20 μm (**E**), 10 μm (**F**), 15 μm (**G**), 150 μm (**H**).

**Figure 104.**
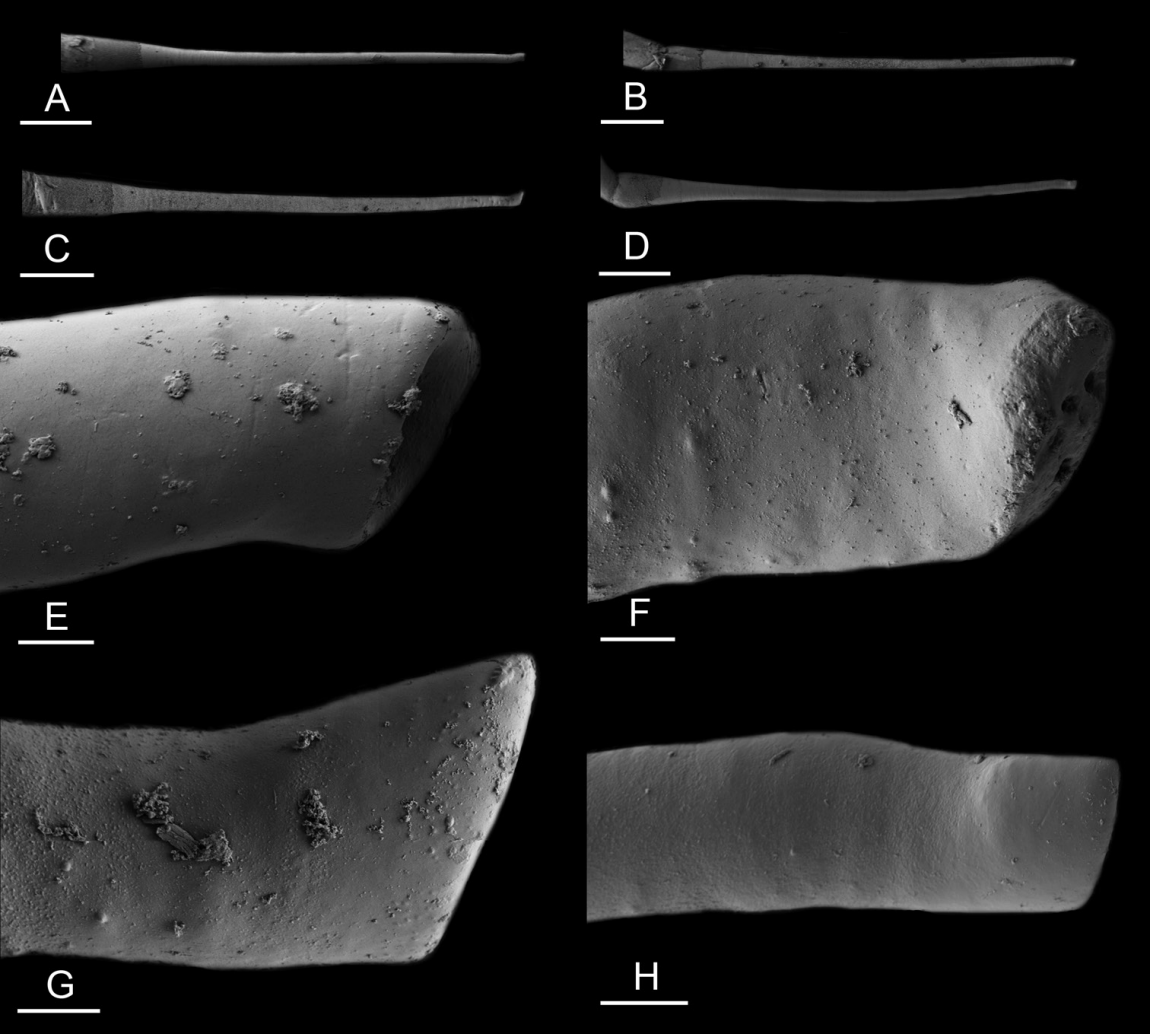
Accessory penial gland spine, *Peronia verruculata* (unit #1) **A** Vietnam [5621] (ITBZC IM 00021) **B** Papua New Guinea [5469] (MNHN-IM-2013-12010) **C** Vanuatu [5481] (MNHN-IM-2013-62393) **D** New Caledonia [6214] (MNHN-IM-2019-1593) **E** same as **A**; **F** same as **B**; **G** same as **C**; **H** same as **D**. Scale bars: 300 μm (**A, B, D**), 200 μm (**C**), 10 μm (**E–G**), 20 μm (**H**).

**Figure 105.**
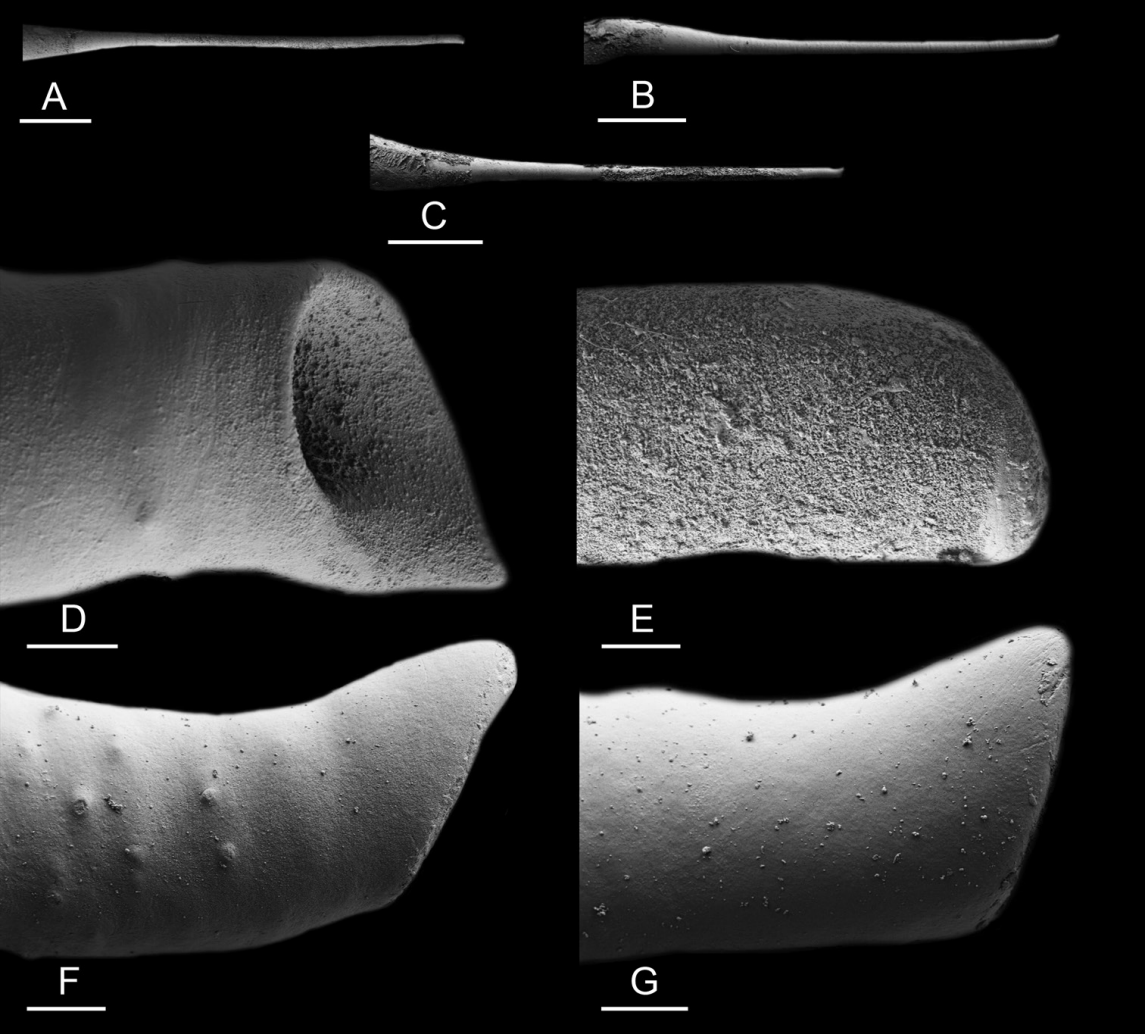
Accessory penial gland spine, *Peronia verruculata* (unit #2), Indonesia, Sumatra **A, E** [1797] (UMIZ 00180) **B, F** [1795] (UMIZ 00180) **C, G** [1796] (UMIZ 00180) **D** [1746] (UMIZ 00178). Scale bars: 300 μm (**A–C**), 10 μm (**D–G**).

**Figure 106.**
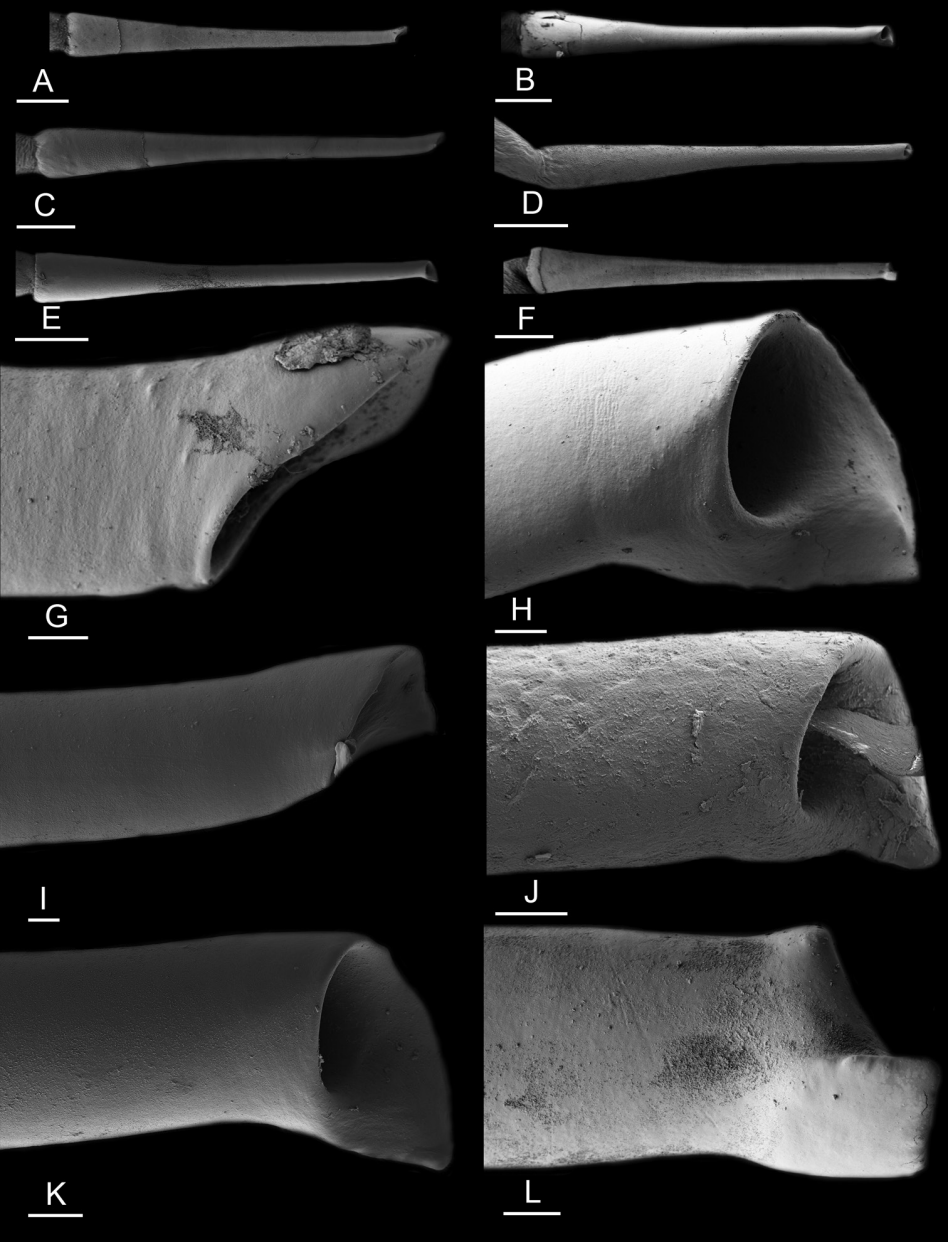
Accessory penial gland spine, *Peronia verruculata* (unit #3) **A–C, E–I, K, L** Peninsular Malaysia **D, J** Singapore **A** [975] (USMMC 00064) **B** [976] (USMMC 00051) **C** [977] (USMMC 00064) **D** [989] (ZRC.MOL.16070) **E** [2546] (USMMC 00065) **F** [2547] (USMMC 00065) **G** same as **A**; **H** same as **B**; **I** same as **C**; **J** same as **D**; **K** same as **E**; **L** same as **F**. Scale bars: 300 μm (**A–F**), 20 μm (**G–L**).

**Figure 107.**
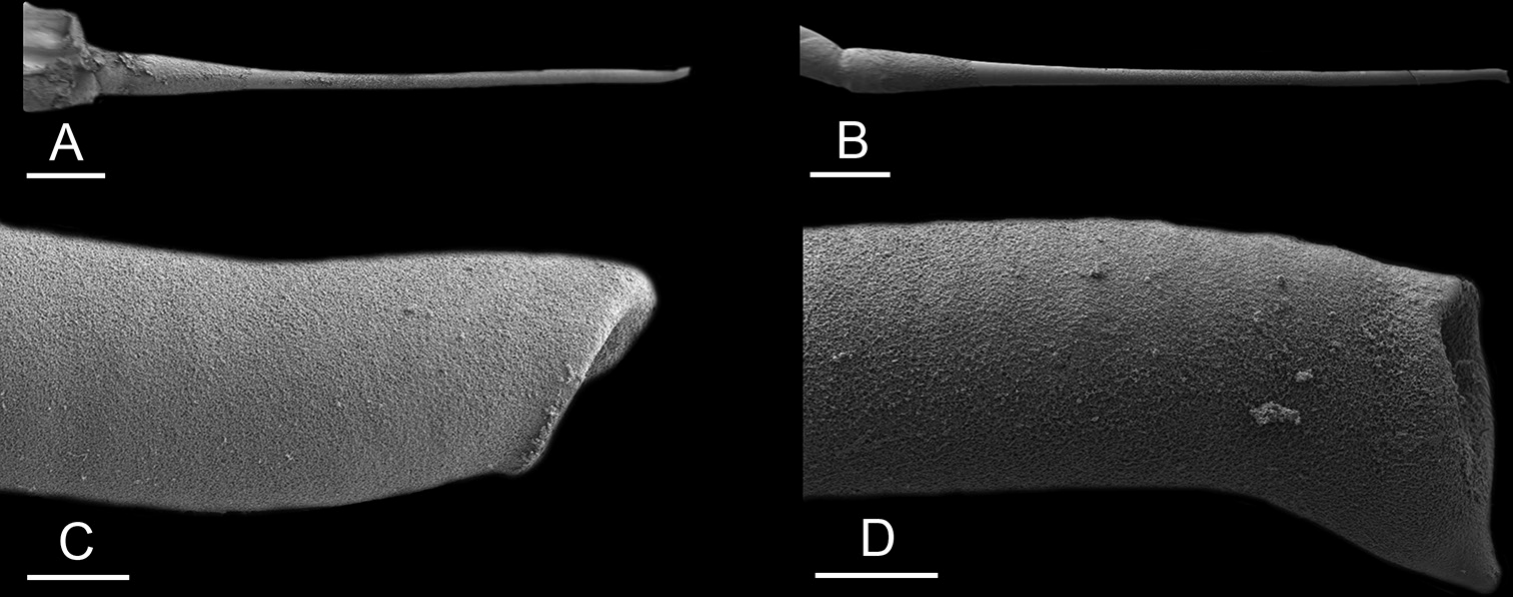
Accessory penial gland spine, *Peronia verruculata* (unit #4), Pakistan **A, C** [6164] (MNHN-IM-2019-**1384**) **B, D** [6166] (MNHN-IM-2019-**1386**). Scale bars: 300 μm (**A, B**), 20 μm (**C, D**).

**Figure 108.**
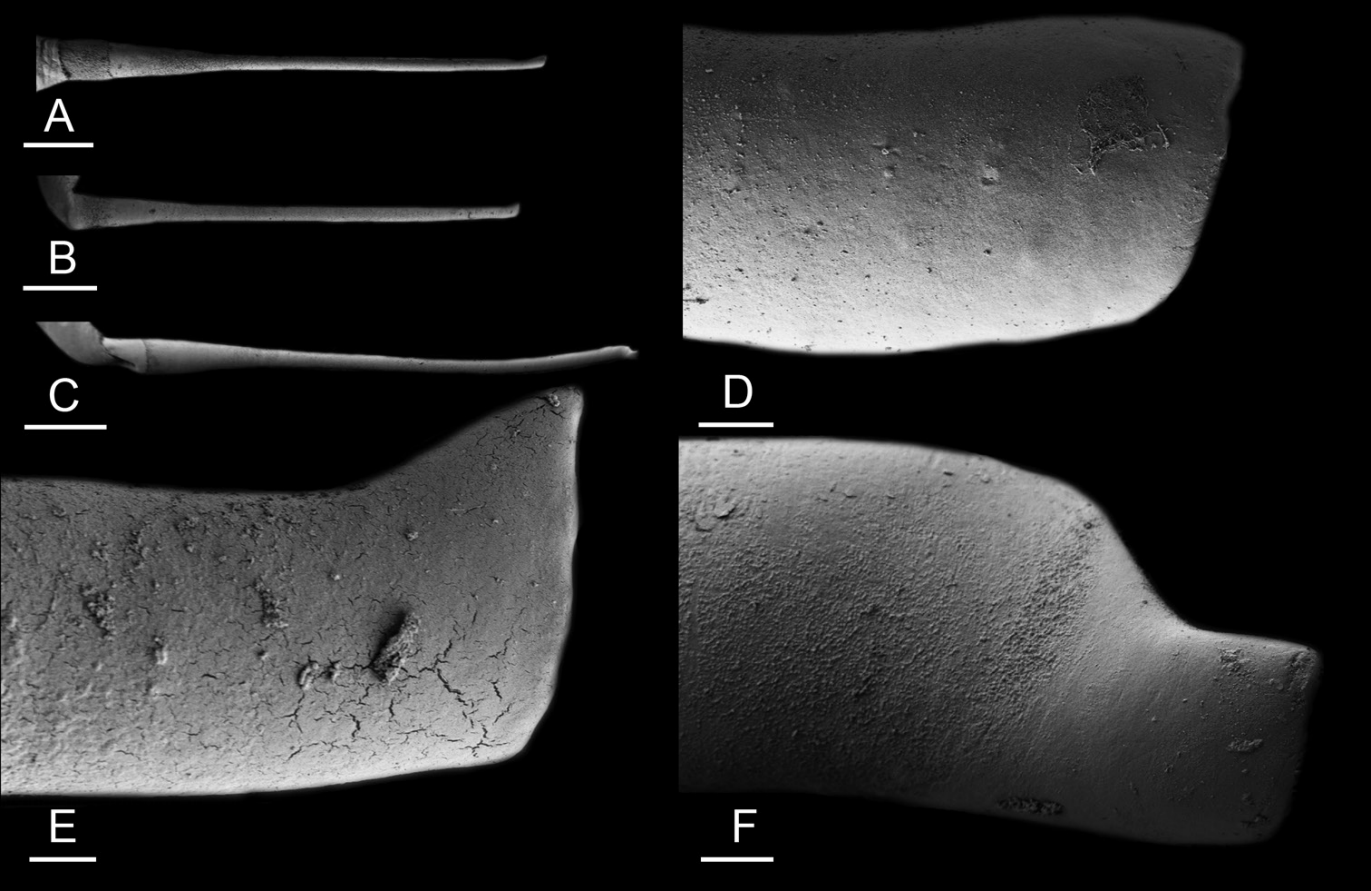
Accessory penial gland spine, *Peronia verruculata* (unit #5) **A** Madagascar [3231] (MNHN-IM-2019-1610) **B** Madagascar [3144] (MNHN-IM-2019-1611) **C** Mozambique [5510] (MNHN-IM-2013-62398) **D** same as **A**; **E** same as **B**; **F** same as **C**. Scale bars: 300 μm (**A–C**), 10 μm (**D–F**).

**Figure 109.**
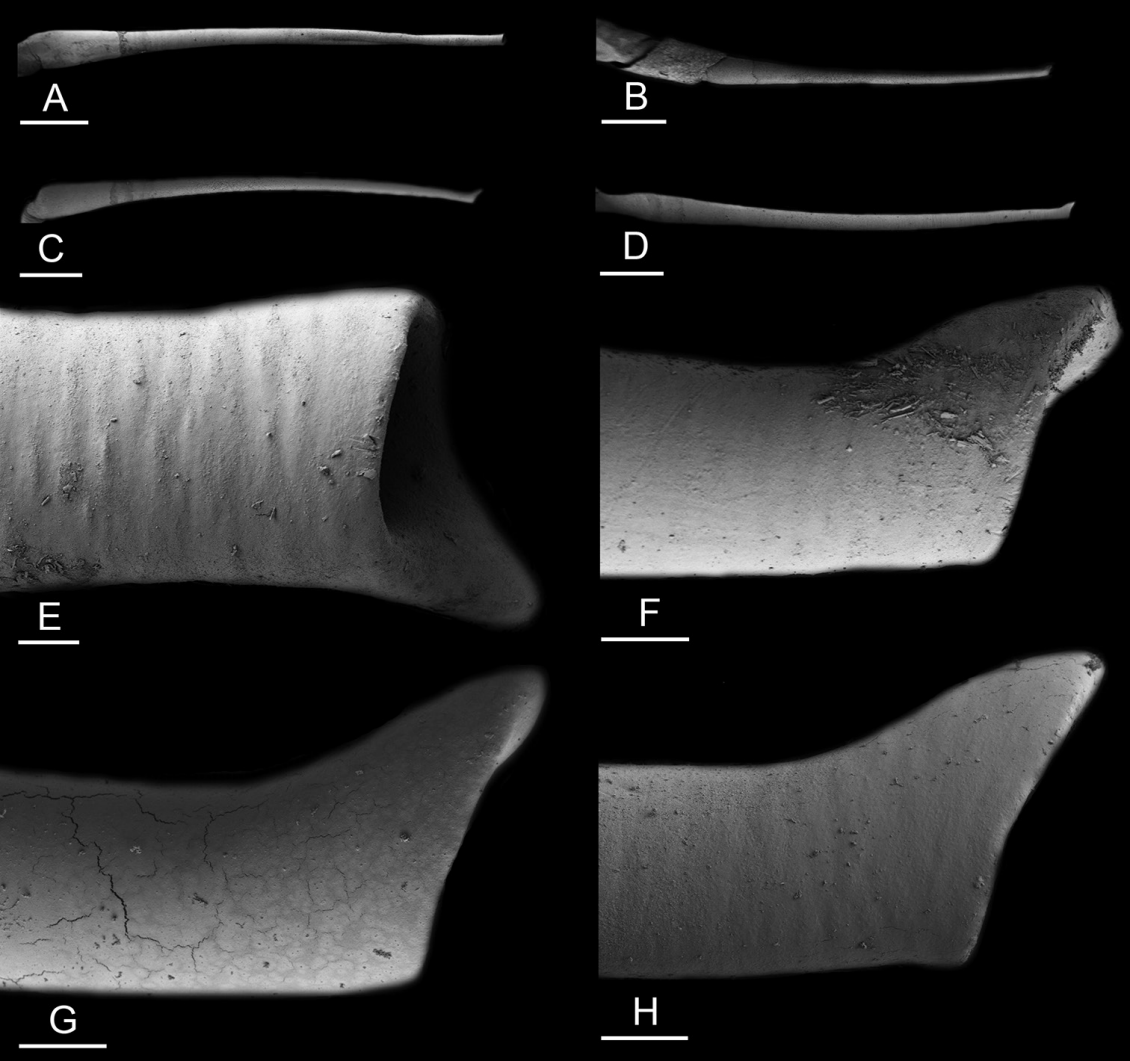
Accessory penial gland spine, *Peronia verruculata*, Red Sea **A, E** spm #1 (ZMH 27472/4) **B, F** spm #2 (ZMH 27472/4) **C, G** spm #3 (ZMH 27472/4) **D, H** spm #4 (ZMH 27472/4). Scale bars: 300 μm (**A, C, D**), 400 μm (**B**), 10 μm (**E**), 20 μm (**F–H**).

The hollow spine is narrow, elongated, conical at its base, and straight or slightly curved (Figs 103–109). Its shape varies between individuals, including at its tip which may or may not be pointed. Its length (Table 4) ranges from 1.4 mm ([5481] MNHN-IM-2013-62393) to 2 mm ([5068] UMIZ 00166, [5469] MNHN-IM-2013-12010) in unit #1, from 1.4 mm ([1796] UMIZ 00180) to 1.7 mm ([1797] UMIZ 00180) in unit #2, from 1.8 mm ([990] ZRC.MOL.10496) to 2.2 mm ([976] USMMC 00051) in unit #3, from 2.2 mm ([6165] MNHN-IM-2019-1385) to 2.8 mm ([6164] MNHN-IM-2019-1384) in unit #4, from 1.8 mm ([3144] MNHN-IM-2019-1611) to 2 mm ([3231] MNHN-IM-2019-1610) in unit #5, from 2 mm (ZMH 27472, spm #4) to 2.4 mm (ZMH 27472, spm #2).

Its diameter at the base (Table 4) ranges from 100 μm ([5481] MNHN-IM-2013-62393) to 200 μm ([5621] ITBZC IM 00021) and even, exceptionally, 270 μm ([991] ZRC.MOL.10497) in unit #1, from 140 μm [1796] UMIZ 00180) to 160 μm [1797] UMIZ 00180) in unit #2, from 200 μm ([989] ZRC.MOL.16070) to 270 μm ([977] USMMC 00064) in unit #3, around 200 μm ([6164] MNHN-IM-2019-1384, [6165] MNHN-IM-2019-1385, and [6166] MNHN-IM-2019-1386) in unit #4, from 150 μm ([3144] MNHN-IM-2019-1611) to 180 μm ([3231] MNHN-IM-2019-1610) in unit #5, and from 140 μm (ZMH 27472, spm #4) to 200 μm (ZMH 27472, spms #2 and #3) in unit #6.

Its diameter at the tip (Table 4) ranges from 35 μm ([5481] MNHN-IM-2013-62393) to 50 μm (e.g., [5068] UMIZ 00166) in unit #1, from 30 μm [1795] UMIZ 00180) to 35 μm [1796] UMIZ 00180) in unit #2, and from 40 μm [989] ZRC.MOL.16070) to 80 μm [977] USMMC 00064) in unit #3, around 50 μm ([6164] MNHN-IM-2019-1384, [6165] MNHN-IM-2019-1385, and [6166] MNHN-IM-2019-1386) in unit #4, from 45 μm ([3231] MNHN-IM-2019-1610) to 50 μm ([3144] MNHN-IM-2019-1611) in unit #5, and from 55 μm (ZMH 27472, spm #1) to 60 μm (ZMH 27472, spms #2 to #4) in unit #6.

###### Diagnostic features

(Table 4). Externally, *Peronia verruculata* cannot be distinguished from the other *Peronia* species. The animal length, which is helpful to identify *P. peronii*, does not help identify *P. verruculata*. In our material, most live animals are between 30 and 40 mm long, but some animals are exceptionally longer: e.g., 60 mm for [2162] (UMIZ 00171) in Sulawesi, 60 mm for [5620] (ITBZC IM 00021) in Vietnam, and 73 mm for [6212] (MNHN-IM-2019-1592) in New Caledonia. Internally, all units of *P. verruculata* are cryptic with each other (Table 4). The ranges of sizes for the accessory penial gland (length, diameter at base, diameter at tip) overlap when all units are considered, but ranges may differ when only a pair of units is considered (e.g., the accessory penial gland spine is shorter than 160 μm in unit #2 and longer than 200 μm in unit #3).

The units #1 and #3 are sympatric in Singapore but they cannot be always separated anatomically. Based on the length of its spine (270 μm), the Singapore individual [991] would be assigned to the mitochondrial unit #3 because the spine is longer than 200 μm in unit #3 while it usually is less than 200 μm in unit #1, but it belongs to the mitochondrial unit #1 (Fig. 2). The diameter of the tip of the spine only partly overlaps between unit #1 (from 35 to 50 μm) and unit #3 (from 40 to 80 μm), but that trait is hardly practical when it comes to identification (it requires SEM). The units #1 and #2 are sympatric in Sumatra (we found them both together at the stations 78 and 82) but they cannot be separated because they are completely cryptic anatomically (Table 4). All that is not to say that there are no anatomical differences between units of *P. verruculata*. On average, the diameter of the spine of the accessory penial gland tends to be larger both at the base and at the tip in unit #3. However, because ranges of variation overlap, anatomical traits cannot be used to reliably assign individuals to any particular unit.

*Peronia verruculata* is close anatomically to *P. sydneyensis* and *P. willani*. They all share intestinal loops of type I with a transitional loop oriented between 3 and 6 o’clock. There are, however, important differences. The muscular sac of the accessory penial gland is significantly longer in *P. willani* (up to 25 mm) than in *P. verruculata* (up to 15 mm); the spine of the accessory penial gland is significantly shorter in *P. sydneyensis* (less than 1 mm) than in *P. verruculata* (at least 1.3 mm); strong, hemispherical protuberances cover the spine in all individuals of *P. sydneyensis* and are absent in all other species. *Peronia sydneyensis* and *P. verruculata* cannot be confused even where they are sympatric (Queensland and New Caledonia) and *Peronia verruculata* and *P. willani* are not sympatric based on current data.

###### Remarks.

***Species delineation.*** Our decision of recognizing a single species with high population structure and several mitochondrial units is explained in the results (see species delineation). Fresh material from the Red Sea, Somalia, Yemen, Oman, and the Persian Gulf is needed to determine the relationships between the populations of *P. verruculata* from the Red Sea and the remainder of the species. Similarly, fresh material is needed from southwestern and southeastern India, including Sri Lanka, to determine the relationships between the western (Indian Ocean) and eastern (South-East Asia and West Pacific) populations. Most likely, additional populations will show that mitochondrial units are even more mixed than what is already shown here, and new units may be found. Nuclear markers will remain indispensable as the current data show that populations that seem divergent using mitochondrial markers are not reproductively isolated. It is not excluded that populations from the Red Sea belong to two distinct species (both with intestinal loops of type I): *P. verruculata* and another species endemic to the Red Sea. The *Peronia* diversity in the Red Sea would thus be similar to what is found in Japan, which is also at the periphery of the distribution of *Peronia* (Fig. 6).

***Synonymy.*** The application of all the species names regarded as junior synonyms of *P. verruculata* is addressed here, following a chronological order starting with *P. verruculata* (Tables 1, 6).

Based on our data, there are two *Peronia* species in the Red Sea, one characterized by intestinal loops of type I (with a transitional loop oriented between 3 and 6 o’clock) and the other characterized by intestinal loops of type V (see remarks on *P. madagascariensis*). Because the intestinal loops of the lectotype of *O. verruculatum* are of type I (Fig. 86A), *P. verruculata* applies to the species described here with intestinal loops of type I.

The original description of *Onchidium ferrugineum* was published four times in different venues by Lesson, twice in 1831 (first in the *Bulletin des sciences naturelles* and then in the zoology section of the *Coquille* voyage), once in February 1832 (in the *Mémorial encyclopédique*), and once again in 1833 (in his *Illustrations de Zoologie*). According to Cretella (2010), the date of publication for the description of *O. ferrugineum* in the *Coquille* voyage is November 15, 1831. Therefore, the oldest and original description of *O. ferrugineum* is the one published in the *Bulletin des sciences naturelles* in April 1831. Both descriptions from 1831 did not include any illustration. An illustration of an animal ventral view was published by Lesson (1832: 36–37, fig. 32) in the *Mémorial encyclopédique*. Two beautiful, colored pictures were published in Lesson’s (1833: pl. 19) *Illustrations de Zoologie*.

The type locality (of the lectotype) of *Onchidium ferrugineum* is Manokwari, West Papua, Indonesia, where at least three *Peronia* species are known to be present (Fig. 6). Based on the length of the lectotype (35 mm) and its intestinal loops of type I with a transitional loop at 4 o’clock (Fig. 80A), *Onchidium ferrugineum* applies to the species described here (*P. verruculata*) and not to *P. griffithsi* or *P. peronii* (Table 4). Unfortunately, this identification cannot be confirmed by the muscular sac or the spine of the accessory penial gland, which are missing in the lectotype. Labbé (1934a: 213–216) claims that there is no accessory penial gland in *Onchidium ferrugineum* and thus does not comment on the spine and the muscular sac of the accessory penial gland of the lectotype. It is unclear whether Labbé dissected the lectotype or if he found it already dissected by Lesson (who commented on the penis of the paralectotypes and thus might have dissected the lectotype as well). Eleven dorsal papillae with eyes were counted on the lectotype, but it is possible that others faded with time.

Lesson (1833: pl. 19) transferred *Onchidium ferrugineum* to *Peronia*. In the written description, Lesson (1833: unnumbered page) considered *Peronia ferruginea* the type of a genus which he decided to call *Peronia*, following Blainville, but the type species of *Peronia* is *O. peronii*, by monotypy, and the author of *Peronia* is Fleming (1822a, b). Oken (1834b: 269–270) reported *Peronia ferruginea* from Lesson’s (1833: pl. 19) *Illustrations de Zoologie*. Van der Hoeven (1850: 786; 1856: 817) suggested, based on Lesson’s (1833: pl. 19) own illustration, that *Peronia ferruginea* may be a nudibranch instead of an onchidiid, but there is no question that *Peronia ferruginea* applies to an onchidiid species. Gray (1850: 117), Adams and Adams (1855: 235), and Tapparone Canefri (1883: 214) classified *O. ferrugineum* in *Peronia* but other authors preferred the original combination with the generic name *Onchidium*.

Semper (1882: 268) kept *O. ferrugineum* in *Onchidium* and regarded it as a questionable name because he (erroneously) thought that its original locality was unknown. Plate (1893) did not comment on it. Bretnall (1919: 326–327) thought that *O. ferrugineum* referred to a species insufficiently known and merely repeated Lesson’s original description. Bretnall (1919: 326) also suggested that *O. ferrugineum* seemed “closely related to that of M. de Blainville,” i.e., *Peronia mauritiana*, a synonym of *P. peronii*. Solely based on information from the original description, Hoffmann (1928: 71, 74) regarded *O. ferrugineum* as a junior synonym of *O. verruculatum* and disagreed with Bretnall that it could refer to *O. peronii*. However, the application of *O. ferrugineum* cannot be deduced from the original description, especially because it is based on *Peronia* and *Wallaconchis* specimens (see above the remarks on the type material). Finally, Labbé (1934a: 213–216), who re-examined the type specimens of *O. ferrugineum*, created the generic name *Lessonia* (later replaced by *Lessonina*) for *O. ferrugineum*, for a genus characterized by a unique combination of traits (large and coiled penis, dorsal gills, etc.), without realizing that the types of *O. ferrugineum* were part of two species from two different genera.

*Peronia savignii* is an objective junior synonym of *Onchidium verruculatum* because they share the same lectotype (see above, the remarks on the type material of *Peronia savignii*). Hoffmann (1928: 69, 72), Labbé (1934a: 193), and Dayrat (2009: 16) all regarded *Onchidium savignyi* Semper, 1800 as an emendation of *Peronia savignii* Récluz, 1869, and *Onchidium savignyi* as a junior synonym of *O. verruculatum*. However, *Onchidium savignyi* Semper, 1880 is not an emendation of *Peronia savignii* Récluz, 1869. Semper (1880: 260–261, pl. 19, fig. 6, pl. 20, fig. 1, pl. 22, figs 5–9) created *Onchidium savignyi* as a new name for two individuals from Bohol, Philippines. Semper (1880: 260) merely suggested (with a question mark) that his *Onchidium savignyi* might refer to the same species as “O. Peronii Savigny, Description de l’Egypte,” but did not even mention the existence of *Peronia savignii*. One syntype of *Onchidium savignyi* is well preserved and still undissected (ZMB/Moll 39018). Its notum bears gills, which agrees with Semper’s original description and means that *Onchidium savignyi* refers to a *Peronia* species. Therefore, *Peronia savignyi* (Semper, 1880) is a secondary junior homonym of *Peronia savignii* Récluz, 1869 because ICZN Article 58 applies (*savignyi* and *savignii* are deemed to be identical spellings). As junior secondary homonym, *Peronia savignyi* is an available but subjectively invalid name (ICZN 1999: Article 57.3) (Tables 1, 6).

Some authors (Hoffmann 1928: 72–74; Labbé 1934a: 193; Connolly 1939: 454) regarded *Onchidium savignyi* as a junior synonym of *O. verruculatum*, while others regarded *Onchidium savignyi* as valid (e.g., Connolly 1912: 225; Collinge 1910: 172). Also, *Onchidium savignyi* Semper has naturally caused some confusion with respect to whether it refers to the same species as *Peronia savignii* Récluz (e.g., Fischer and Crosse 1878: 697; Smith 1903: 401). Strangely enough, the situation is simple: *Peronia savignii* Récluz is objectively invalid (as junior objective synonym of *O. verruculatum*) and *Peronia savignyi* (Semper) is subjectively invalid (as junior secondary homonym *O. savignii*). Semper’s description of the two specimens of *O. savignyi* from Bohol seems to suggest that they belong to *P. verruculata* (e.g., retractor muscle attaching to the end of the body cavity, accessory penial gland spine 2.5 mm long), but the long muscular sac (22 mm long) matches better the anatomy of *P. peronii* (Table 4).

The two syntypes of *Onchidium branchiferum* are from Manila, Luzon, Philippines. Anatomical traits described by Plate (insertion of the retractor muscle of the penis at the end of the visceral cavity, spine of the accessory penial gland 1 mm long) indicate that *O. branchiferum* applies to *P. verruculata*, even though they cannot be confirmed on the syntypes in which all internal organs are either missing or destroyed (Table 4). Plate did not draw the intestinal loops but he describes them as being type I (the orientation of the transitional loop is unknown). The number of radular teeth per half row (88) also matches what is known in *P. verruculata* (Table 5). According to Plate (1893: 184), *O. branchiferum* is easily recognizable because its branchial plumes are only present on the posterior end of the dorsum (posterior sixth). However, this trait is not distinct from other species and varies depending on preservation (gills are often retracted in preserved specimens and can only be observed if specimens were carefully relaxed before preservation). Plate (1893) did not provide any other feature supporting *O. branchiferum* as a distinct species, and he did not compare it with any other existing species. *Onchidium branchiferum* is regarded here as a new junior synonym of *P. verruculata* (Tables 1, 6).

Hoffmann (1928: 75) listed *Onchidium branchiferum* as a valid name (solely based on information from the original description) but considered it to refer to a “local form” of *O. verruculatum*. Labbé (1934a: 194) transferred *Onchidium branchiferum* to *Peronia* and regarded *P. branchifera* as a valid name “out of deference to the eminent zoologist Ludwig Plate” even though he agreed with Hoffmann that *P. branchifera* most likely was just a local form of *P. verruculata*. Labbé’s re-description of *P. branchifera* was based on a specimen (30/23 mm) collected by Ach. Cuming in 1844 from an unknown locality in the Philippines. There are two jars preserved at the MNHN with *Peronia* specimens collected by Ach. Cuming in 1844. Labbé (1934a: 192–194) also re-described a specimen collected from the Philippines by Ach. Cuming in 1844 as *P. verruculata*. It is not possible to determine which jar corresponds to what species in Labbé’s (1934a) monograph because Labbé did not indicate species identifications for any of the MNHN specimens he examined. Labbé’s description of a “short penial gland” indicate that he most likely examined *P. verruculata* (unit #1). Finally, Marcus and Marcus (1970: 213) wrote that *P. branchifera* was close to *P. verruculata* but with no explanation.

*Onchidium elberti* was described by Simroth (1920) from Muna Island, southeastern Sulawesi, Indonesia, where only *Peronia verruculata* is known to be present (Fig. 6). Internal features of the holotype (24 mm long) are fully compatible with the anatomy of *P. verruculata*: intestinal loops are of type I with a transitional loop oriented at 5 o’clock (Fig. 80B) and the muscular sac of the accessory penial gland is 8 mm long (Table 4). Eleven papillae with dorsal eyes were counted (which fits within the range of the species) but some may have faded with time. As a result, *Onchidium elberti* is regarded here as a junior synonym of *Peronia verruculata* (Tables 1, 6). Hoffmann (1928: 71, 75) thought *Onchidium elberti* was a junior synonym of *O. verruculatum*, based on information from Simroth’s original description.

*Onchidium astridae*, the type species of Labbé’s genus *Scaphis*, was originally described by Labbé (1934b) within the genus *Onchidium*. Only one specimen is known, the holotype (20/18 mm) by monotypy, from Sorong, West Papua, Indonesia. There is no doubt that *Onchidium astridae* applies to a *Peronia* species because the dorsum of the holotype bears gills. All copulatory parts are missing and Labbé did not describe the length of the muscular sac or the length of the spine of the accessory penial gland. Labbé (1934a: 213, fig. 46) described two muscular sacs instead of just one, but that could not be confirmed here. At least three *Peronia* species are present in West Papua (Fig. 6). However, given the size of the holotype (20 mm long) and, importantly, its intestinal loops of type I with a transitional loop at 4 o’clock (Fig. 80C), *Onchidium astridae* is regarded as a junior synonym of *P. verruculata* (Tables 1, 4, 6). Note that the number of papillae with dorsal eyes could not be counted on the preserved holotype. According to Labbé (1934b), *Onchidium astridae* is close to *Onchidium vaigiense* and *O. steenstrupi*, but both names refer to *Marmaronchis vaigiensis*, a species which belongs to a distinct genus (Dayrat et al. 2018).

The original description of *Peronia gaimardi* was based on two specimens from Vanikoro, Solomon Islands, which were found at the MNHN, and one specimen from Djibouti, which could not be located. The type locality is Vanikoro, locality of the lectotype designated in the present study. Our molecular data demonstrate that *Peronia verruculata* (unit #1) is present in Vanikoro, but *P. peronii* and *P. platei* could also be found there (Fig. 6). Given the intestinal loops of type I (with a transitional loop at 5 o’clock) observed in the lectotype (Fig. 80F), *P. gaimardi* is regarded as a synonym of *P. verruculata* (Tables 1, 4, 6). The male parts of the lectotype are missing and Labbé’s description of the copulatory apparatus is confusing because it is based indiscriminately on individuals from both Vanikoro and Djibouti. His measurement of the spine of the accessory gland (8 mm long) is most likely a mistake. In the present study, the longest spine (5 mm long) was found in the lectotype of *P. fidjiensis* (a synonym of *P. peronii*) from Fiji. Also, the lectotype of *P. gaimardi* only is 44 mm long, which would make it a very small individual of *P. peronii*. Given the large size (80 mm long, according to Labbé) of the paralectotype from Djibouti, it most likely belongs to *P. madagascariensis*, a species present there, and for which large specimens are known (Table 4). It would imply that Labbé confused its intestinal loops of type V for a type I, which is a mistake he often made. Marcus and Marcus (1970: 214) wrote that *P. gaimardi* might be a junior synonym of *P. verruculata* based on information from the original description.

*Peronia anomala*, originally described from the Red Sea, is regarded as a junior synonym of *P. verruculata* because, contrary to what Labbé indicated in the original description, *Peronia anomala* is characterized by intestinal loops of type I (Fig. 86B). It is assumed in this work that there is only one species of *Peronia* slugs with intestinal loops of type I in the Red Sea, although fresh material from the Red Sea may show that there is more than one species. Marcus and Marcus (1960: 881) suggested that *P. anomala* could be a synonym of *P. verruculata* and that intestinal loops of both types I and II are found in *P. verruculata*, but intestinal loops are only of type I in *P. verruculata* and there are no intestinal loops of type II in *Peronia*. Maniei et al. (2020a: table S1) took Labbé’s description for granted and considered that *P. anomala* was characterized by intestinal loops of type II.

The type specimens used by Labbé for the original description of *Paraperonia gondwanae* belong to several species, because our data show that slugs with intestinal loops of types I and V necessarily belong to distinct species. The application of the name *Paraperonia gondwanae* is determined by the lectotype from Bombay (MNHN-IM-2000-33681) with intestinal loops of type I (Fig. 84A). *Paraperonia gondwanae* applies to *P. verruculata*, and, more specifically, to the populations of the mitochondrial unit #4 from western India and Pakistan (Fig. 6, Tables 1, 6). The paralectotypes from the Red Sea with intestinal loops of type I also belong to *P. verruculata*: one “*e*” paralectotype from the Red Sea (MNHN-IM-2000-33688), and two “*d*” paralectotypes from Suez (MNHN-IM-2000-33684). The paralectotypes with intestinal loops of type V belong to *P. madagascariensis*: one of the “*a*” paralectotypes from Bombay (MNHN-IM-2000-33682) and one of the “*c*” paralectotypes from Suez (MNHN-IM-2000-33683). The large specimen with intestinal loops of type I from Mauritius (MNHN-IM-2000-33686), which may or may not be part of the type material of *P. gondwanae*, likely belongs to *P. peronii*.

*Scaphis viridis* was described by Labbé based on three syntypes (four according to the original description) from Thursday Island, in the Torres Strait, Australia. The presence of *P. verruculata* in the Torres Strait is not demonstrated positively with fresh material. However, *P. verruculata* is the only species we found in northeastern Queensland (up to Cairns, 16°S). None of the *Peronia* slugs we collected north of Bowen (20°S) were individuals of *P. sydneyensis* which is thought to be only distributed from southern Queensland down to New South Wales (Sydney) and eastwards to New Caledonia. More importantly, both the original description (Labbé 1934a: 207–208, figs 31–34) and the traits examined in the lectotype here confirm that *S. viridis* applies to *P. verruculata* (Table 4): intestinal loops of type I with a transitional loop at 5 o’clock (Fig. 80F; Labbé 1934a: fig. 32), muscular sac of the accessory penial gland 14 mm long (Labbé) and 15 mm long (lectotype), spine of the accessory penial gland 1 mm long (Labbé) and 1.7 mm long (lectotype), retractor muscle attaching at the posterior end of the visceral cavity. Because those traits are only compatible with the anatomy of *P. verruculata*, *S. viridis* is regarded here as a junior synonym of *P. verruculata* and which applies to the unit #1 (Tables 1, 6). Finally, a total of 13 dorsal papillae with eyes was observed in the lectotype; more may have faded with time. Labbé only compared *S. viridis* with *Peronia acinosa*, a *nomen dubium* which may or may not refer to an onchidiid species (see general discussion).

There are three *Peronia* species in New Caledonia, the type locality of *Scaphis carbonaria* (Fig. 6). DNA sequences of individuals from New Caledonia belong to two species in our molecular data set (*P. verruculata* and *P. sydneyensis*). Although our molecular data do not include any specimen of *P. peronii* from New Caledonia, it is present there based on the rest of its distribution (it is found all the way to Fiji and Tonga; Fig. 6) and on an old specimen from a historical museum collection (ANSP 203028). Two characters in Labbé’s original description are problematic. The penis, described as “wide and short, without hooks” (Labbé 1934a: 209, our translation), is absolutely incompatible with *Peronia*, in which the penis is thin, elongated, and always with hooks in the distal region. The absence of dorsal eyes on the notum is also quite perplexing. The notum of the holotype is in poor condition and its dorsal eyes cannot be seen, likely because their black color faded. However, dorsal gills are clearly present on the notum and there is no doubt that *S. carbonaria* applies to a *Peronia* species. Based on the length of the muscular sac of the penial accessory gland (10 mm), *S. carbonaria* is not a junior synonym of *P. peronii*. However, its muscular sac and its intestinal loops of type I with a transitional loop oriented at 4 o’clock are compatible with both *P. verruculata* and *P. sydneyensis* (Table 4). The length of the spine helps distinguish both species but Labbé did not mention it and it is missing in the holotype. Therefore, strictly speaking, *S. carbonaria* should be regarded as a *nomen dubium*. However, because there are many older names available for the unit #1 of *P. verruculata* (Table 6), *S. carbonaria* can be regarded as another junior synonym of *P. verruculata*. It would make no sense to apply it to *P. sydneyensis* because several important organs (the penis, the spine of the penial accessory gland, the radula) are missing in the holotype and because Labbé’s original description is problematic and incomplete.

**Table 6.** Available species names that apply to the five mitochondrial units of *Peronia verruculata* as well as populations in the Red Sea (not represented by DNA sequences in the present study).

Unit	Current distribution	Names available	Type locality
#**1**	Singapore to eastern Australia, New Caledonia & Japan	*Onchidium ferrugineum* Lesson, 1831a	West Papua, Indonesia
		*Onchidium branchiferum* Plate, 1893	Luzon, Philippines
		*Onchidium elberti* Simroth, 1920	Sulawesi, Indonesia
		*Onchidium astridae* Labbé, 1934b	West Papua, Indonesia
		*Peronia gaimardi* Labbé, 1934a	Vanikoro, Solomon Islands
		*Scaphis viridis* Labbé, 1934a	Torres Strait, Australia
		*Scaphis carbonaria* Labbé, 1934a	New Caledonia
		*Scaphis tonkinensis* Labbé, 1934a	Vietnam
		*Scaphis lata* Labbé, 1934a	Vietnam
#**2**	Sumatra & Andaman Islands	–	–
#**3**	Peninsular Malaysia & Singapore	–	–
#**4**	Persian Gulf, Pakistan & western India	*Paraperonia gondwanae* Labbé, 1934a	Mumbai, western India
		*Peronia persiae* Maniei et al., 2020a	Persian Gulf, Iran
#**5**	Mozambique & Madagascar	*Scaphis gravieri* Labbé, 1934a	Mayotte
	Red Sea	*Onchidium verruculatum* Cuvier, 1830	Red Sea
		*Peronia savignii* Récluz, 1869	Red Sea
		*Peronia anomala* Labbé, 1934a	Red Sea
		*Onchidium durum* Labbé, 1934a	Red Sea

*Scaphis gravieri* was described originally based on types from Mayotte, Zanzibar, and Djibouti. The application of *Scaphis gravieri* is now based on the lectotype from Mayotte (MNHN-IM-2000-33695), with intestinal loops of type I (Fig. 85A). Our data do not include fresh material from Mayotte, but Mayotte is located between Madagascar and Mozambique where *P. verruculata* (unit #5) is present. Therefore, *S. gravieri* is regarded as a junior synonym of *P. verruculata* (Tables 1, 4, 6). Note that *P. madagascariensis*, a distinct species with intestinal loops of type V, also is expected to be present in Mayotte, even though it has not been recorded there so far (Fig. 6). The presence of *P. verruculata* in Zanzibar (locality of some paralectotypes of *S. gravieri*) is possible but needs to be confirmed with fresh material. Additional, non-type specimens from Zanzibar were examined (MNHN-IM-2014-7989, MNHN-IM-2014-7990): their intestinal loops are of type I with a transitional loop at 5 o’clock. Therefore, those specimens cannot belong to *P. madagascariensis* (intestinal loops of type V) or *P. peronii* (intestinal loops of type I with a transitional loop oriented between 12 and 3 o’clock), and thus likely belong to *P. verruculata*. *Peronia verruculata* is expected to be present in Djibouti (locality of some paralectotypes of *S. gravieri*), but that still needs to be demonstrated with fresh material from the northwestern Indian Ocean (Somalia, Yemen, Oman) as well as from the Red Sea and the Persian Gulf.

Pieces of possibly up to three syntypes of *Scaphis tonkinensis* were located at the MNHN (MNHN-IM-2000-33700) but they are useless, poorly preserved, unidentifiable pieces of tissues. Determining the status of *S. tonkinensis* thus relies entirely on Labbé’s original description. Given that *P. verruculata* (unit #1) is the only species known in Vietnam, and that several characters provided by Labbé (1934a: 213) match its anatomy (muscular sac 12 mm long, intestinal loops of type I), *S. tonkinensis* is regarded as a junior synonym of *P. verruculata* (Tables 1, 4, 6).

No type material could be located for *Scaphis lata*. Determining the status of *S. lata* thus relies entirely on Labbé’s original description. Labbé’s original description to determine its status. Given that *P. verruculata* (unit #1) is the only species known in Vietnam, and that several characters provided by Labbé (1934a: 213) match its anatomy (muscular sac 8 mm long, intestinal loops of type I), *S. lata* is regarded as a junior synonym of *P. verruculata*. Labbé mentioned the presence of dorsal gills and so at least some of the syntypes of *S. lata* were *Peronia* slugs. The fact that he also described intestinal loops of type II (which is absent in *Peronia*) means that he either made a mistake (all loops were of type I) or that some syntypes were not *Peronia* slugs.

*Onchidium durum*, originally described from the Red Sea, is regarded as a junior synonym of *Peronia verruculata* because, contrary to what was indicated in the original description, *Onchidium durum* is characterized by dorsal gills and intestinal loops of type I. It is presumed here that there is only one species of *Peronia* with intestinal loops of type I in the Red Sea. Labbé frequently confused types of intestinal loops; there are no well-documented cases of *Peronia* slugs with intestinal loops of type II.

*Peronia persiae*, originally described from the Persian Gulf, is regarded as a new junior subjective synonym of *P. verruculata* because its mitochondrial DNA sequences, represented by the GenBank “voucher LaFM7S” in our analyses, all cluster together within the unit #4 of *P. verruculata* (Fig. 2). An older name, *P. gondwanae* (Labbé, 1934a), already refers to the unit #4 of *P. verruculata* (Tables 1, 6). So, even in the hypothetical event that unit #4 would later need to be named as a distinct taxon (of subspecific or specific rank), *P. persiae* would still remain invalid because *P. gondwanae* would always take priority over it.

The description of *P. persiae* by Maniei et al. (2020a) is an example of the common but regrettable practice that consists in creating new species names without a comprehensive revision, which almost inevitably leads to increasing the number of unnecessary synonyms (Dayrat 2005). Here are a few of the major methodological issues in the study by Maniei et al. (2020a). First, Maniei et al. (2020a) ignored the existence of many available *Peronia* species names, which is especially problematic in the case of names with type localities near the Persian Gulf (Table 1), such as *Onchidium durum* and *Paraperonia jousseaumei* with a type locality in the Red Sea, and *Scaphis gravieri* with a type locality in Mayotte. Second, Maniei et al. (2020a) decided to create a new name before the nomenclatural status of the other *Peronia* names was addressed. For instance, Maniei et al. (2020a: table S1) compared *P. persiae* with *P. branchifera*, *P. ferruginea*, *P. gaimardi*, and *P. lata* as if they were all valid names, but these names all refer to the unit #1 of *P. verruculata* (Tables 1, 6). Third, Maniei et al. (2020a) only examined specimens of *P. persiae* from the Persian Gulf, which means that, for comparison, they relied exclusively on the literature which, as the present work shows, is plagued with taxonomic and anatomical errors. For instance, Maniei et al. (2020a: table S1) assumed that the intestinal loops of *P. verruculata* were of types I and II, but it is positively demonstrated here that the intestinal loops of *P. verruculata* are all of type I and that there are no loops of type II in *Peronia*. Fourth, apart from *P. persiae*, only *P. verruculata* and *P. peronii* are represented in the phylogenetic trees by Maniei et al. (2020a: figs 11, 12), exclusively based on sequences obtained from GenBank (many of which were misidentified). Most specimens in their phylogenetic trees are not even identified at the species level. Using DNA sequences to create a new species name while most species are not being included in phylogenetic analyses is highly problematic.

Maniei et al. (2020b) used the same mitochondrial COI sequences as in Maniei et al. (2020a) to compare metabolites between the *Peronia* slugs they called *P. persiae* and one *Peronia* individual from Bangka Island, near Sumatra, Indonesia. That specimen from Bangka Island, identified as *Peronia* sp. 7 by Maniei et al. (2020a) and as *P. verruculata* by Maniei et al. (2020b), belongs to the unit #1 of *P. verruculata*: its COI (MK993397) and 16S (MK993396) sequences cluster within unit #1. Note that the GenBank accession numbers for COI and 16S are switched in Maniei et al.’s (2020a) Table 2.

Maniei et al. (2020b) summarized their rationale for creating the name *P. persiae* as follows: “The ABGD test revealed that specimens of *P. persiae* form a separate clade (clade 2). Thus, the specimens from two localities of the Persian Gulf (Iran), i.e. Bandar Lengeh and Lavan Island, were considered as a distinct new species.” Mitochondrial loci alone are not sufficient evidence to delineate species: molecular delimitation analyses can over-split species based on population structure, particularly when these are based on a single locus (Sukumaran and Knowles 2017). More importantly, very high intra-specific mitochondrial divergence has been repeatedly documented in several onchidiid genera (e.g. Goulding et al. 2018c; Dayrat et al. 2019a).

Maniei et al. (2020b) argue that research on metabolites requires sound taxonomic knowledge. That certainly is a commendable goal: indeed, any comparative work in any biological field should be based on correct taxonomy. Unfortunately, *P. persiae* is a junior synonym of both *P. gondwanae* and *P. verruculata* (Tables 1, 6). So, the metabolites compared between “*P. persiae*” and “*P. verruculata*” merely are intra-specific differences (within *P. verruculata*) due to the long geographic distance (between the Persian Gulf and Bangka Island) as well as, most likely, different diets: in fact, Maniei et al. (2020b) acknowledged in their introduction that numerous biotic and abiotic factors influence the chemical composition. To conclude anything about specific differences in metabolites among *Peronia* based on specimens from only two regions, one of which being represented by a single individual is, to say the least, premature. In order to demonstrate that distinct metabolites are found in distinct species, one needs to study actually distinct species, i.e., species that were reliably identified, and one also needs specimens of the same species from different habitats and from different locations. It is our hope that the present, comprehensive, taxonomic revision will help physiologists, biochemists, ecologists, etc., to identify *Peronia* slugs correctly.

Some comments are also needed regarding the original anatomical description of *P. persiae* by Maniei et al. (2020a). According to Maniei et al. (2020a: 510, fig. 6, table S1), the intestinal loops of *P. persiae* are of type II, but they are without doubt of type I: the transitional loop is oriented at ~ 5 o’clock, as in intestinal loops of type I (Fig. 1). The radular formulae provided by Maniei et al. (2020a: 509) fit well with what was observed here for the unit #4 of *P. verruculata* (Table 5), acknowledging individual variation: from 49 × 47.1.47 (in a live specimen 22 mm long) up to 71 × 87.1.87 (in a live specimen 65 mm long). The length of the spine of the accessory penial gland (“around 1.3 mm”) reported by Maniei et al. (2020a: 513) is shorter than what was observed here (from 2.2 to 2.8 mm) but this trait is known to vary between individuals (Table 4). Maniei et al. (2020a: table S1) compared the shape of the tip of the spine of the accessory penial gland between species, but that trait varies greatly intra-specifically and is useless to distinguish species. Finally, Maniei et al. (2020a: 513, fig. 8B) reported some “fork-shaped” penial hooks, which were also observed here in the unit #4 of *P. verruculata* (Fig. 100C).

***Additional material (historical museum collections).*** A specimen from Tanimbar, Indonesia (WAM S26630) is identified as *P. verruculata* because of its accessory gland spine (1.5 mm long), its intestinal loops of type I (with a transitional loop at 3 o’clock), and its muscular sac (10 mm). Seven specimens from Zanzibar (MNHN-IM-2014-7989 and MNHN-IM-2014-7990) are also identified as *P. verruculata* because their internal anatomy is only compatible with that species (Table 4). Finally, specimens from the Persian Gulf (NHMD 635301) with intestinal loops of type I (with a transitional loop at 6 o’clock) demonstrate that there is more than one *Peronia* species in the Persian Gulf (Fig. 6). Indeed, based on our DNA sequences, *P. madagascariensis* (with intestinal loops of type V) is present in the Persian Gulf, and individuals with intestinal loops of type I must belong to a different species. Given that *P. verruculata* is known from Pakistan and western India (unit #4), eastern Africa (unit #5), and the Red Sea, it most likely lives in the Persian Gulf too. The fresh material recently described as *P. persiae* by Maniei et al. (2020a) confirms with molecular data the presence of the unit #4 of *P. verruculata* in the Persian Gulf (Fig. 2). In addition, several historical specimens preserved at various institutions were examined for the present study. They are discussed below in the secondary literature section because they were studied by previous authors.

***Secondary literature.*** JE Gray (1850: 117) and Adams and Adams (1855: 235) did not mention *Onchidium verruculatum* in their list of *Peronia* species names. That might seem surprising because they transferred to *Peronia* all slugs with “radiating processes” (Gray 1850: 117) or “arbusculiform tufts” (Adams and Adams 1855: 234) on the dorsal notum. And, clearly, *Onchidium verruculatum* refers to a species of slugs with such appendages. However, there is an explanation. *Onchidium verruculatum* was created by Cuvier (1830: 281) in reference to Savigny’s (1817: pl. 2, figs 3.1–3.8) illustrations (of slugs from the Red Sea) for which the figure captions by Audouin (1826: 18–20) used Cuvier’s (1804) older name *Onchidium peronii*, originally described from Mauritius. JE Gray (1850: 117) mentioned Savigny’s *Onchidium peronii* in his list of *Peronia* species names and ME Gray reproduced some of Savigny’s drawings: ME Gray’s (1850: pl. 183, figs 4, 4a, 5) illustrations are exact copies of Savigny’s (1817: pl. II, figs 3.1–3.3) illustrations. Note that Savigny (1817: pl. II, figs 3.1–3.3) illustrated two individuals which may or may not belong to the same species (see above, remarks on the type material of *O. verruculatum*). Also, note that one of Savigny’s (1817: pl. 2, fig. 3.5) drawings beautifully illustrates dorsal gills, which JE Gray was certainly aware of. So, long story short, JE Gray (1850) knew the existence of Savigny’s (1817: pl. 2, figs 3.1–3.8) illustrations of slugs with dorsal gills from the Red Sea but decided to refer to them using Cuvier’s (1804) older name *Onchidium peronii* (from Mauritius) and ignore Cuvier’s (1830) newer name *O. verruculatum* (specifically created for those slugs from the Red Sea). Similarly, Adams and Adams (1855: 235) mentioned *P. peronii* with a reference to Savigny’s (1817) plate, not to Cuvier’s (1804) original description of *P. peronii*, which means that, exactly like JE Gray (1850), Adams and Adams (1855: 235) decided to ignore Cuvier’s newer name *Onchidium verruculatum* created for the slugs on Savigny’s plate.

Keferstein (1865b) described as *Peronia verruculata* three slugs from Nagasaki, Japan. Keferstein’s (1865b: pl. VI, fig. 16) drawing of the internal anatomy unmistakably illustrates intestinal loops of type V, which means that he examined *P. setoensis* instead of *P. verruculata* (see remarks on *P. setoensis*). It is unclear whether Keferstein’s (1865a: pl. CII, figs 20*, 20**, pl. CV, figs 1, 2) drawings illustrate the same Nagasaki individual as the one with intestinal loops of type V (Keferstein 1865b: pl. VI, fig. 16). It cannot be excluded that Keferstein examined several of the species found in Japan (Fig. 6). Keferstein (1865a: pl. CIV, figs 9–12) also reproduced four of Savigny’s (1817: pl. 2, figs 3.2, 3.3, 3.5, 3.7) original drawings used as a reference by Cuvier for the name *Onchidium verruculatum*. And note, again, that the two individuals illustrated by Savigny may or may not both belong to *O. verruculatum*: one specimen belongs to *P. verruculata* but the other may belong to *P. madagascariensis* (see above, remarks on the type material of *O. verruculatum*). At any rate, to our knowledge, it is in Keferstein’s (1865a, b) work that *Onchidium verruculatum* was first transferred to *Peronia*.

Both H. Nevill (1870: 304–305) and G. Nevill (1878: 1) mentioned the presence of *Onchidium verruculatum* in Ceylon. It most likely refers to *P. verruculata*, although there possibly is more than one mitochondrial unit in southern India.

Mörch (1872a: 28; 1872b: 325) mentioned *Peronia verruculata* (as spelling mistake *vermiculata* in 1872b) from Pulo Milu [Pulo Milo, Little Nicobar] and Nancouri [Nancowry, Nicobar Islands], where he says it is common. Those specimens, re-examined for the present study (NHMD 613753), are a record of *P. peronii* (see remarks on *P. peronii*).

Specimens collected during the Galathea Expedition from a bay in Sambelong, Great Nicobar, were examined and the largest individual (35/28 mm) was dissected (NHMD 635300). Those specimens are important historically because they were mentioned by several authors (see below). Given their size (35/28 to 20/15 mm), their digestive system (type I with a transitional loop oriented at 6 o’clock, in the largest individual), and the size of their accessory gland spine (1 mm in the largest individual), those specimens belong to *P. verruculata*, but could potentially belong to more than one mitochondrial unit (Table 4). Mörch (1872a: 28; 1872b: 325) first mentioned them as *Peronia mauritiana*. Semper (1880: 255) identified them as *O. verruculatum*. Bergh (1884a) described one of them in detail (see below). Hoffmann (1928: 44, 73) also listed them in his material examined for *O. verruculatum*.

Schmeltz (1874: 96) listed *Peronia verruculata* from Samoa in a catalog of the Museum Godeffroy. This possibly is a record of *P. peronii*, although *P. platei* could also live there (Fig. 6).

Ihering (1877: 230–237, pl. IV, fig. 3) described the nervous system of *Peronia verruculata* but did not provide any information on the specimens he examined. It is impossible to determine what *Peronia* species he actually studied.

Fischer and Crosse (1878: 689–690, pl. XXXI, figs 13–15) briefly described the radula of specimens they identified as Onchidium (Peronia) verruculatum from New Caledonia. There are three *Peronia* species in New Caledonia, and it is not possible to determine what species they examined.

Semper (1880: 255–257, pl. 22, figs 3, 4; 1882: pl. 21, fig. 1) re-described *O. verruculatum* based on specimens from a variety of localities (Red Sea, East Coast of Africa, Nicobar, Ambon, eastern Australia, Philippines). His written description mostly focuses on traits that are not informative for species identification (e.g., number of dorsal papillae, number of dorsal eyes, radular teeth). Some of Semper’s records of *P. verruculata* most likely are correct, given the geographic origin of the material (Fig. 6): Ambon, Philippines, and Cape York (Queensland, Australia). Some other material could be a mix of more than one species: *P. madagascariensis* and *P. verruculata* in the Red Sea and eastern Africa; *P. verruculata* and *P. sydneyensis* in MacKay, Queensland. Semper’s material from Brisbane (27°S) most likely was part of *P. sydneyensis* (Fig. 6). Finally, Semper’s specimen from Nicobar was part of some material collected during the Galathea Expedition and first reported by Mörch (1872a: 28; 1872b: 325) as *Peronia mauritiana* (NHMD 635300). Those specimens, re-examined for the present study, belong to *P. verruculata* (see above).

Bergh (1884a: 148–151, pl. VII, figs 7–12, pl. VIII, fig. 14) described in detail the anatomy of an individual of *O. verruculatum* from Nicobar. The animal size (33/23 mm) and the size of the accessory penial gland spine (1.76 mm) match well the anatomy of *P. verruculata* (unit #1). This specimen was part of a group of specimens collected during the Galathea Expedition in Sambelong, Great Nicobar, which were examined for the present study (NHMD 635300). Their size (35/28 to 20/15 mm), their digestive system (type I with a transitional loop oriented at 6 o’clock, in the largest individual), and the length of their accessory gland spine (1 mm in the largest individual) are also compatible with *P. verruculata*. However, those specimens could potentially belong to more than one mitochondrial unit (Fig. 6).

Plate’s (1893: 168–170, pl. 7, figs 11, 12, 15, pl. 8, figs 26, 33, pl. 9, figs 36–40, pl. 10, figs 50a, 55, pl. 11, fig. 56, pl. 12, figs 83, 88, 90, 98) re-description of *Onchidium verruculatum* was based on specimens from Ambon, Ceylon, and Nicobar. Given the size (at most 50 mm long), it seems likely that Plate examined *P. verruculata*. However, without precise measurements of the spine of the accessory penial gland, it is not possible to ascertain that Plate examined *P. verruculata* (e.g., *P. peronii* is also present in Nicobar). According to Plate, the only anatomical difference between *O. verruculatum* and *O. savignyi* Semper, 1880, is that dorsal gills are “much longer and tubular” in *O. savignyi*, which is a weak character, to say the least. At any rate, *Peronia savignyi* (Semper, 1880) is deemed to be identical to *Peronia savignii* Récluz, 1869 (ICZN 1999: Article 58) and, as junior secondary homonym, is subjectively invalid (ICZN 1999: Article 57.3) (see remarks on synonymies above). Plate (1893) did not compare *O. verruculatum* to any other species.

Von Martens (1897: 126) mentioned *Onchidium verruculatum* from both Ambon and Timor with no description. Our molecular data indicate that *Peronia verruculata* does live in Ambon and Timor. However, *Peronia peronii* also lives in Timor and likely lives in Ambon too.

Farran (1905: 358–359, pl. VI, figs 13–22) described a *Peronia* slug he identified as *Onchidium verruculatum* from the Gulf of Mannar based on one preserved specimen. Given the specimen size (31/34 mm) and the length of the spine of the penial accessory gland (2.8 mm), it is likely a record of *P. verruculata*, but it is unclear whether it is the unit #2 (known from the Andaman Islands) or unit #4 (known from Mumbai, western India). It could also be a record of a small, immature individual of *P. peronii* (which has not been recorded from southern India but could possibly be found there). Our present study does not include any specimen from Sri Lanka or the Gulf of Mannar.

*Onchidium verruculatum* is one of the eight onchidiid species mentioned by Hedley (1909: 369) from Queensland, Australia, without any reference to any material. It is impossible to know what species Hedley refers to. Our data show that there are two *Peronia* species in Queensland which overlap geographically (Fig. 6).

The references listed by Bretnall (1919: 310) for *Onchidium verruculatum* are all commented on above already. Let us say a few words about the specimens he examined himself. Bretnall’s (1919: 310) records of *O. verruculatum* from Broken Bay, New South Wales (33°30'S) are likely records of *Peronia sydneyensis*, the only *Peronia* species known in New South Wales (Fig. 6). Bretnall’s (1919: 310) records of *O. verruculatum* from Port Curtis, Queensland (ca. 23°30'S) could be records of *P. sydneyensis* but they could also include *P. verruculata* because the known southernmost locality of the mitochondrial unit #1 of *P. verruculata* is at ca. 21S (see remarks on *P. sydneyensis*).

The record of *Onchidium verruculatum* from Katsepy (Catsèpe), northwestern Madagascar, by Odhner (1919: 23) is within the geographical range of both *P. verruculata* (unit #5) and *P. madagascariensis* (Fig. 6). The voucher specimen, re-examined here (SMNH 180724), clearly belongs to *P. verruculata* because of its intestinal loops of type I (with a transitional loop at 6 o’clock).

Hoffmann (1928: 72) listed many references for *O. verruculatum*, all of which (but one) are commented upon elsewhere already: comments on the references for *Onchidium peronii*, *O. punctatum*, and *Peronia mauritiana* can be found in our remarks on *P. peronii*; comments on the references for *Onchidium ferrugineum* and *O. elberti* can be found above, in our remarks on synonymies; *Peronia alderi* is regarded as a *nomen dubium* and is commented on in the general discussion. Mörch’s (1872a: 28; 1872b: 326) record of Peronia (Onchidiella) marmorata from Nicobar Islands, which Hoffmann (1928: 72) included in his list of correct references for *O. verruculatum*, is commented on here: it is not possible to know to what species Mörch refers; Godwin-Austen (1895: 443) listed Mörch’s record as Onchidium (Onchidiella) marmorata in a faunistic inventory of Nicobar and Andaman, without clarifying to what species that name was referring. At any rate, Lesson’s (1831b) *Onchidium marmoratum* belongs to *Marmaronchis* (Dayrat et al. 2018).

More importantly, Hoffmann (1928: 44) examined specimens from the collections in Stockholm and Copenhagen which he identified as *O. verruculatum*. Most of those specimens could be re-examined for the present study and are commented on here. Several specimens are confirmed here to belong to *P. verruculata* based on diagnostic anatomical traits (Table 4): the material from Karachi (SMNH 180721) belongs to the unit #4 of *P. verruculata*; the material from Hong Kong (SMNH 180707) and Queensland (SMNH 180712, 180713, 180714) belongs to the widespread unit #1; the material from Singapore (SMNH 180716) and the Java Sea (SMNH 180719, 180720, 180722) could belong to any of the three units (#1, #2, #3) present in the region. However, several specimens listed by Hoffmann (1928: 44, 72) clearly do not belong to *P. verruculata* (see remarks on each corresponding species): the specimen from Port Natal, South Africa (SMNH 180711) belongs to *P. madagascariensis*; the specimen from Sagami Bay, Japan (SMNH 180725) belongs to *P. setoensis*; and the specimen from Port Darwin, Northern Australia (SMNH 180715) belongs to *P. willani*.

The Red Sea specimens from the Copenhagen collections listed as “Savigny leg., Mus. Marsil” belong to *P. verruculata* because of their intestinal loops of type I (NHMD 90791). The label in the jar says that they were obtained by the Copenhagen Museum in 1860 (journal entry) from Savigny and the museum of Marseille (erroneously spelled “Marsielle”). Given that the type material of *O. verruculatum* was originally illustrated by Savigny (1817), it is worth making it clear here that those specimens are not the type material of *O. verruculatum* (Hoffmann did not say they were). The type material of *O. verruculatum* is in Paris (MNHN-IM-2000-22941).

The other specimens mentioned by Hoffmann (1928: 44, 73) could not be re-examined for the present study: the specimens from the Red Sea could potentially belong to *P. verruculata* or *P. madagascariensis*; the specimens from Tharangambadi (Tranquebar), southeastern India, most likely belong to *P. verruculata*; the specimens from New Caledonia could potentially belong to any of the three species present there; the specimens from Hawaii clearly belong to *P. platei*.

All the references mentioned by Labbé (1934a: 192–193) for *Peronia verruculata* are already commented on above. Labbé (1934a: 193) blindly accepted the distribution provided by Hoffmann (1928: 44, 73), which was not accurate because, for instance, *P. verruculata* is not present in Hawaii (see above). Labbé (1934a: 193) mentioned intestinal loops of type II in one individual from the Red Sea, even though he did not list any material examined from the Red Sea. At any rate, those intestinal loops were most likely of type I as aforementioned Labbé often made that kind of mistake. For instance, Labbé (1934a: 196) described as *P. anomala* a species with supposedly anomalous intestinal loops of type II, but the type material, re-examined here, clearly is characterized by loops of type I (Fig. 86B). The specimens examined by Labbé from the Philippines likely belong to *P. verruculata*, but the individuals from New Caledonia or New Guinea could belong to several *Peronia* species. Finally, so far, only *P. peronii* and *P. griffithsi* are positively known from Mauritius and his record of *P. verruculata* there (as Ile de France) must not be taken for granted.

The record of Onchidium (Peronia) verruculatum from Natal, South Africa (Connolly 1939: 454) likely is a record of *P. madagascariensis*, the only *Peronia* species so far known from South Africa. However, *P. verruculata* (unit #5) could also be found in northeastern South Africa because its southernmost known locality is in Maputo (ca. 26°S), very close to South Africa.

Allan and Bell (1947: 152) and Allan (1950: 368) reported onchidiid slugs living in dead coral which they identified as *Onchidium verruculatum* from Moreton Bay, Brisbane, Queensland, Australia. Given its latitude (ca. 27°S), Brisbane is clearly in the range of *P. sydneyensis* and possibly of *P. verruculata* (unit #1) as well. Indeed, it is still unclear how far south *P. verruculata* is distributed in southeastern Australia, although we did not find it in Sydney, ca. 33S (see remarks on *P. sydneyensis*).

For the record of *O. verruculatum* from New South Wales by Dakin (1947: 144), see remarks on *P. sydneyensis*.

Awati and Karandikar (1948) published a detailed anatomical study of a species they identified as *Onchidium verruculatum* based on material from the western coast of India. They mention four localities: Vengurla (ca. 15°50'S), Malvan (ca. 16°06'S), Mumbai (ca. 19°S), and Kathiawar (ca. 21°S). The illustration of the intestinal loops provided by Awati and Karandikar (1948: fig. 6) leaves no doubt about the fact that they examined individuals of *P. madagascariensis*, a species with intestinal loops of type V distributed from South Africa all the way to (at least) Mumbai. Whether a type V was observed by the authors in all the specimens, including those from the southernmost localities (Vengurla and Malvan), is unclear. The presence of intestinal loops of type V in all the specimens examined by Awati and Karandikar (1948) would mean that *P. madagascariensis* is found much farther south than Mumbai. If the authors did not notice that some intestinal loops were of type I, then they described two species under the name *Onchidium verruculatum*: *P. madagascariensis* and *P. verruculata* (Fig. 6).

Baba (1958: 144) reported that some individuals of *Onchidium verruculatum* from Tokara Islands (ca. 30°N), just south of Kyushu, were very large (up to 120 mm long), suggesting that they were *P. peronii* instead (see remarks on *P. peronii*). The smaller specimens, however, could be *P. verruculata* (unit #1) and possibly *P. setoensis* (see remarks on *P. setoensis*). The two species which Baba (1958: 21) seems to distinguish (as *Onchidium* and *Onchidium verruculatum*) from Misaki (ca. 34°N), near Osaka, could be *P. verruculata* (unit #1) and *P. setoensis*, which, based on our DNA sequences, are sympatric near the Seto Marine Laboratory, which is close to Osaka (Fig. 6).

Solem (1959: 39) recorded *O. verruculatum* from Vanuatu (Esperitu Santo) which hosts at least two species: *P. verruculata* (unit #1) and *P. peronii* (Fig. 6). The references that he mentioned (Bretnall 1919; Hoffmann 1928; Awati and Karandikar 1948) are already commented on above. His proposed distribution (“Esperitu Santo, Africa to Japan, New Guinea, Australia and New Caledonia. Also common in Hawaii, but not known from Polynesia”) is inaccurate (Fig. 6). For instance, the only *Peronia* species in Hawaii is *P. platei* (for comments on Solem’s comparison between *O. peronii* and *O. verruculatum*, see remarks on *P. peronii*).

Menon et al. (1961: 493, pl. 10, fig. 84) mentioned *Onchidium verruculatum* in the Gulf of Kutch, northwestern India, which is within the distribution range of both *P. madagascariensis* and *P. verruculata* (Fig. 6). Our data suggest that *P. verruculata* (mitochondrial unit #4) is found from Pakistan (north of the Gulf of Kutch) to Mumbai (south of the Gulf of Khambhat). Also, a paralectotype of *P. gondwanae* from Mumbai (MNHN-IM-2000-33682) with intestinal loops of type V suggests that *P. madagascariensis* lives south of the Gulf of Kutch and Gulf of Khambhat.

Marcus and Marcus (1970: 213) recorded *P. verruculata* from Madagascar based on one specimen (45/20 mm) for which they reported intestinal loops of type II. Most likely, they confused types of intestinal loops (there are no documented intestinal loops of type II in *Peronia* slugs). The specimen they examined likely belongs to *P. verruculata* (unit #5), characterized by intestinal loops of type I, but it could also belong to *P. madagascariensis*, which is characterized by intestinal loops of type V. Both species were recorded in Madagascar and both are characterized by a retractor muscle inserting at the end of the visceral cavity, a trait reported by Marcus and Marcus (1970: 213).

For the record of *O. verruculatum* from the central coast of New South Wales by Smith and Kershaw (1979: 92), see remarks on *P. sydneyensis*.

Gopinadha Pillai and Appukuttan (1980: 34) listed *Onchidium verruculatum* in the Gulf of Mannar, with no description or material. It likely refers to *P. verruculata*.

Hutchings and Recher (1982: 119) listed *Onchidium verruculatum* from Northern Territory, Queensland, and New South Wales, Australia. Based on our data, *O. verruculatum* (unit #1) is only present in Queensland, but it cannot be excluded that it also is present in northern New South Wales (see remarks on *P. sydneyensis*). Northern Territory and New South Wales host other species than *P. verruculata* (Fig. 6).

Britton (1984: 183–184, fig. 2) described *Peronia verruculata* from Hong Kong. Some of the specimens he examined clearly are *Peronia* slugs because he mentioned dorsal gills, and they most likely belong to *Peronia verruculata* (unit #1), the only species known from the coast of China (Fig. 6). However, his specimen with intestinal loops of type II (NHMUK 1982284) was misidentified because there are no intestinal loops of type II in *Peronia*.

Katagiri and Katagiri (2007) distinguished two species (both as *Onchidium verruculatum*) in the waters of the Boso Peninsula (ca. 35N, near Sagami Bay, Honshu) based on external appearance and development. Most likely, those two species correspond to *P. verruculata* (unit #1) and *P. setoensis*, which are the only two *Peronia* species found north of 30N (Fig. 6; see remarks on *P. setoensis*).

The name *Peronia verruculata* mentioned in ecological studies in Japan (Nakaoka et al. 2006; Wardiatno et al. 2015) can potentially refer to any of the four *Peronia* species found in Japan (Fig. 6). The name *P. verruculata* mentioned from Mumbai and the Gulf of Khambhat, northwestern India (Mandal and Harkantra 2013; Solanki et al. 2017), could refer to both *P. verruculata* (unit #4) and *P. madagascariensis* (Fig. 6). The name *P. verruculata* mentioned from the coast of mainland China (Sun et al. 2014, 2016; Liu et al. 2015; Xu et al. 2018) refers to the mitochondrial unit #1 of *P. verruculata* (Fig. 6). The name *P. verruculata* mentioned from the Andaman Islands (Santhosh Kumar et al. 2016) could refer to more than one mitochondrial unit of *P. verruculata* (Fig. 6). The name *Peronia* sp. mentioned from Vietnam (Zvonareva and Kantor 2016: 432, fig. 9D) most likely refers to the mitochondrial unit #1 of *P. verruculata* (Fig. 6).

Chang et al. (2018) reported two *Peronia* least-inclusive units in Singapore based on COI sequences, which correspond to our mitochondrial units #1 (their “*Peronia* sp. 2 clade”) and #3 (their “Singapore clade”): two of their COI sequences were included in our mitochondrial analyses (Table 2 and Fig. 2). Chang et al. (2018) also reported some anatomical differences between those two units, mostly because they only examined individuals from Singapore. The variation in the number of dorsal eyes per dorsal papilla is not different between both units: for instance, up to eight dorsal eyes per papilla are present in our individual [2987] from Lombok. The diameter measurements of the conical base of the spine of the accessory penial gland overlap: they reported 113–181 μm in unit #1 and 187–267 μm in unit #3, but ranges of 100–270 μm in unit #1 and 200–270 μm in unit #3 were observed here (Table 4). Finally, the ranges in diameter at the tip of the spine also overlap between both units: 35–50 μm in unit #1 and 40–80 μm in unit #3 (Table 4).

Two specimens from Mozambique ([730] NHMUK 20080190, and [733] NHMUK 20060257), tentatively identified as *Peronia* sp. 4 and sp. 5 respectively by Dayrat et al. (2011: 428), belong to the unit #5 of *P. verruculata* (Fig. 2). The specimens [731] (NHMUK 20050628) from Sulawesi, Indonesia, and [712] (UF 368518) from Cebu, Philippines, referred to as *Peronia* sp. 6 and *Scaphis* sp. respectively by Dayrat et al. (2011), belong to the unit #1 of *P. verruculata* (Fig. 2).

A few COI sequences available in GenBank are not included in our analyses because they do not add any information regarding the species distribution. Ran et al. (2020) published four COI*Peronia* sequences from Hainan, China (MN389204 to MN389207), which all cluster within *P. verruculata* unit #1. Two unpublished sequences (MN690327 and MN690328) from Singapore (uploaded in November 2019 by Ip and colleagues) cluster within *P. verruculata* units #1 and #3. A COI sequence (MK993397) from Bangka, Indonesia, identified as *Peronia* sp. 7 by Maniei et al. (2020a) and as *P. verruculata* by Maniei et al. (2020b), cluster within *P. verruculata* unit #1. Note that the GenBank accession numbers for COI and 16S of that Bangka Island individual are switched in Maniei et al.’s (2020a) Table 2. Finally, a sequence (EF489391) from Queensland, Australia, identified as *Onchidium verrucosum* (a spelling mistake for *O. verruculatum*) by Klussmann-Kolb et al. (2008), cluster within *P. verruculata* unit #1.

### Identification key

**Table:** 

1	Intestinal loops of type V	**2**
–	Intestinal loops of type I	**5**
2	Spine of the accessory penial gland more than 2 mm long	***P. madagascariensis* (western Indian Ocean)**
–	Spine of the accessory penial gland less than 1 mm long	**3**
3	Spine of the accessory penial gland less than 0.7 mm long	***P. griffithsi* (PNG to Mauritius)**
–	Spine of the accessory penial gland more than 0.7 mm long	**4**
4	Distributed from PNG to Hawaii and French Polynesia	***P. platei***
–	Endemic to Japan (Honshu, Wakayama)	***P. setoensis***
5	Transitional loop oriented between 12 and 3 o’clock	**6**
–	Transitional loop oriented between 3 and 6 o’clock	**7**
6	Spine of the accessory penial gland more than 3 mm long	***P. peronii* (Indo-West Pacific)**
–	Spine of the accessory penial gland less than 2.3 mm long	***P. okinawensis* (endemic to Okinawa, Japan)**
7	Spine of the accessory penial gland with strong hemispherical protuberances on its surface	***P. sydneyensis* (New South Wales, Queensland, New Caledonia)**
–	Spine of the accessory penial gland without strong hemispherical protuberances on its surface	8
8	Penial hooks less than 40 μm long	***P. willani* (endemic to Northern Territory, Australia)**
–	Penial hooks more than 40 μm long	***P. verruculata* (Indo-West Pacific)**

## Discussion

### Specific names not to combine with *Peronia*

Eight *Onchidella* species names were originally created or subsequently transferred to *Peronia* (Table 1). When *Onchidium celticum* was still a *nomen nudum* (Cuvier 1816: 411), Blainville (1826: 523) had mentioned that it should be classified in *Peronia* because it clearly referred to marine onchidiid slugs. *Onchidium celticum* was transferred to *Peronia* by Adams & Adams (1855: 235) and to *Onchidella* by Fischer & Crosse (1878: 687). All authors subsequently agreed with Fisher and Crosse, although Semper (1882: 283–284) still used the combination *Onchidium celticum*. Three names by Quoy and Gaimard (1832) were transferred to *Peronia* by Oken (1834a) but subsequently to *Onchidella* (e.g., Gray 1850): *Onchidium incisum*, *O. nigricans*, and *O. patelloide*. *Peronia irrorata* was first transferred to *Onchidella* by Adams & Adams (1855: 234). *Peronia indolens*, *Peronia marginata*, and *Peronia parthenopeia* Delle Chiaje, 1841 were transferred to *Onchidella* by Fischer & Crosse (1878: 696). Note that the name *Peronia parthenopeia* appears only in the text (Delle Chiaje 1841: 13); the combination *Onchidium parthenopeium* is used on the plate (Delle Chiaje 1841: pl. 46, figs 6–9).

Blainville (1826: 523) attributed by mistake the authorship for *Peronia laevis* to “Quoy et Gaimard, Atlas de zoologie du voyage de l’Uranie,” but the specific name *laevis* does not appear in Quoy and Gaimard’s (1825) work. Thus, the author of *Peronia laevis* is Blainville (ICZN 1999: Article 50.1.1). Blainville’s *Peronia laevis* corresponds to Quoy and Gaimard’s (1825) *Onchidium vaigiense* (Dayrat 2009: 12). *Peronia laevis* is thus a junior objective synonym of *O. vaigiense*, of which it shares the same name-bearing type, and is objectively invalid (Table 1). Note that *O. vaigiense* is regarded as a valid name in the genus *Marmaronchis* (Dayrat et al. 2018). In addition to the “Peronie lisse” or *Peronia laevis*, Blainville (1825: 465) also described an “Onchidie lisse” or *Onchidium laeve*, which clearly applies to a veronicellid, as illustrated by Blainville (1827, pl. 41, fig. 7) himself in the *Atlas* of his *Manuel*. Dayrat (2009: 12) erroneously attributed Blainville’s (1827: pl. 41, fig. 7) figure to *Peronia laevis*.

*Onchidium ater* Lesson, 1831a was transferred recently to the genus *Wallaconchis* by Goulding et al. (2018: 63) and *Wallaconchis ater* is a valid species name. Bretnall (1919: 327) and Hoffmann (1928: 68, 83–84) classified it in the genus *Onchidium* and Tapparone Canefri (1883: 212) transferred it to *Onchidella*, exclusively based on information from the original description. Labbé (1934a: 206) transferred *O. ater* to his genus *Scaphis* because he saw “feathery gills” on the dorsum of the two syntypes (MNHN-IM-2000-22950). However, there clearly are no gills on the dorsal notum of the types of *Onchidium ater* (Goulding et al. 2018: 67). Note that the oldest original description of *O. ater* was published by Lesson (1831a: 128) in April 1831 in the *Bulletin des sciences naturelles*, i.e., before the description in the zoology section of the *Coquille* voyage published on 15 November 1831 according to Cretella (2010).

### Names of doubtful application

Fifteen names of doubtful application are discussed here, following a chronological order (Table 1). Blainville (1816: 97) described the new species *Onchidium oniscoides* based on specimens that he saw “en Angleterre,” i.e., in a collection now part of the NHMUK in London (Dayrat 2009: 15). The two syntypes (12/12 and 12/10 mm) of *Onchidium oniscoides* labeled as “*Onchidium oniscoides*, Mus. Sloane” (NHMUK 20190559) clearly are onchidiid slugs but are not *Peronia* slugs. They could possibly belong to *Platevindex luteus*. Regardless, the type locality is unknown because Blainville’s expression “On ignore sa patrie” means that he did not know from where those slugs were. As a result, *Onchidium oniscoides* is regarded as a *nomen dubium*. *Onchidium oniscoides* was transferred to *Peronia* by Blainville (1826: 523) and largely overlooked. Hoffmann (1928) does not mention it in his checklist of onchidiid species names. Labbé (1934a: 243) mentions the name “*Oncidiella onisciforme* de Blainville, 1825” with *Peronia oniscoides* de Blainville as synonym (*onisciforme* being most likely a spelling mistake of *oniscoides*).

According to Quoy and Gaimard (1825: 428, our translation), *Onchidium planatum* is “related to *Onchidium peronii*, with which it differs by its smaller size, its color [dirty greenish], and the shape and arrangement of the dorsal warts.” Also, the “extremely small eyes placed at the superior part of the tentacles” likely refer to the eyes at the tip of the ocular tentacles. The most striking trait of *Peronia peronii*, its dorsal gills, is not mentioned in the original description of *O. planatum*, and it is clearly indicated that the dorsal “warts” of *O. planatum* differ from those of *P. peronii*. So, based on the original description, one could say that *O. planatum* may or may not refer to an onchidiid species. Given that Quoy and Gaimard (1825: 429–430, pl. 66, fig. 9) were able to describe and illustrate as *Onchidium secatum* a slug that obviously is not an onchidiid, the name *Onchidium planatum* is regarded as a *nomen dubium* (which may or may not refer to an onchidiid). There is, at the MNHN, a specimen which is part of the type series of *O. planatum* (MNHN-IM-2000-33706). That specimen is accompanied by three labels: the oldest label says “Onchidium planum, Q. G. Freyc. p. 428., de Guam, MM Quoy et Gaimard, Expn Freycinet.” The name “Peronia” was subsequently added on that oldest label in pencil. Because the oldest label clearly refers to *Onchidium planatum* described from Guam by Quoy and Gaimard (1825: 428), a recent label indicates that the specimen is a syntype of *O. planatum*, from Guam. The third label says “OncidiumPeronii, Guam, Quoy et Gaimard, A. Labbé, dét. 1933,” suggesting that Labbé re-identified that specimen at some point as *Peronia peronii* even though he listed it as part of the material he examined for his re-description of *Onchidium planatum*. The specimen is now completely destroyed and poorly preserved: there are only two pieces of notum of which the length of 55 mm matches the original description; the oral area is totally destroyed; all internal organs are missing; no dorsal gills can be seen, but possibly because the notum is so poorly preserved; and it is unclear if a peripodial groove is present or not. Labbé (1934a: 225), who did not seem to realize that he was looking at one of the syntypes of *O. planatum*, mentioned several internal characters suggesting that *O. planatum* refers to an onchidiid species (intestinal loops of type I, accessory penial gland present with a muscular sac) although whether Labbé did see those structures or not remains an open question (Labbé often described and even drew structures that he could not have seen). The lack of dorsal eyes and gills could be due to the poor preservation, in which case a good guess would be that the (destroyed) syntype of *O. planatum* (MNHN-IM-2000-33706) belongs to a *Peronia* species. However, the fact that no dorsal gills and no dorsal eyes can be seen at all and that it is extremely unclear whether there is a peripodial groove or not seems to suggest that *O. planatum* may not even refer to an onchidiid species. It is very possible that the type series included more than one species.

Semper (1882: 289) listed *O. planatum* as a problematic species name. Hoffmann (1928: 69, 84–85) thought that it was a valid *Onchidium* species name with *Onchidella tabularis* Tapparone Canefri, 1883 and Onchidium (Oncis) applanatum Simroth, 1920 as synonyms. *Onchidella tabularis* is a *nomen dubium* although it is clear that it does not refer to an *Onchidella* species (Dayrat et al. 2016: 37). *Onchidium applanatum* is a valid *Platevindex* species name. *Platevindex applanatum* was described as Onchidium (Oncis) applanatum by Simroth, and *Oncis* was replaced by *Platevindex*. Nothing indicates that *O. planatum* refers to *Platevindex* slugs. Labbé (1934a: 225–226, figs 62–64) adopted Hoffmann’s nomenclature and synonymies. Bretnall (1919: 311) listed Quoy and Gaimard (1825: 428) as a reference for *Onchidium peronii*, but Quoy and Gaimard (1825: 428) merely mentioned the name *O. peronii* in a comparison with *O. planatum*. Regardless, *Onchidium planatum* is regarded as a *nomen dubium* which cannot be applied to any taxon and which may not even refer to onchidiid slugs.

Blainville (1826: 523) attributed by mistake the authorship for *Peronia semituberculata* to “Quoy et Gaimard, Atlas de zoologie du voyage de l’Uranie,” but the specific name *semituberculata* does not appear in Quoy and Gaimard’s (1825) work. Thus, the author of *Peronia semituberculata* is Blainville (ICZN 1999: Article 50.1.1). Blainville’s *Peronia semituberculata* corresponds to Quoy and Gaimard’s (1825) *Onchidium planatum* (Dayrat 2009: 17). *Peronia semituberculata* is thus a junior objective synonym of *O. planatum*, with which it shares the same name-bearing type, and is objectively invalid (Table 1). Note that *O. planatum* is regarded here as a *nomen dubium* which may refer to an onchidiid or not (see above).

Lesson (1831b: 299–300, pl. 14, fig. 2) described *Onchidium granulosum* in the zoology section of the *Coquille* voyage. The publication date (1826) for *Onchidium granulosum* in Dayrat (2009) is erroneous. Based on the collation of the voyage of the *Coquille* by Cretella (2010), the date of the original publication for both the text (pp. 299–300) and the plate (pl. 14, fig. 2) with the name *Onchidium granulosum* is 15 November 1831. *Onchidium granulosum* was recorded by Lesson from “Nouvelle-Irlande,” i.e., New Ireland, Papua New Guinea. The type material could not be located. Gray (1850: 117), Adams and Adams (1858: 234), and Tapparone Canefri (1883: 212) transferred *Onchidium granulosum* to *Onchidella* for no obvious reason. Hoffmann (1928: 86) thought that it was a valid *Platevindex* species name (as *Oncis granulosa*) despite the fact that Lesson’s figure hardly illustrates a *Platevindex* slug. Hoffmann (1928: 86) also thought that *Oncis lata* Plate, 1893 was a junior synonym of *Oncis granulosa* but *Platevindex latus* (Plate, 1893) is a valid species name (a monograph of the genus *Platevindex* is in preparation). Labbé (1934a: 234) adopted Hoffmann’s decision. Semper (1882: 290) regarded it as a problematic name and Bretnall (1919: 327) regarded it as valid but considered that its application remains difficult without access to the type material. *Onchidium granulosum* is a *nomen dubium* which refers to an onchidiid species, possibly a *Wallaconchis* or a *Peronia* species (Table 1).

In 2018, just based on the brief and incomplete original description, *Onchidium cinereum* Quoy & Gaimard, 1833 was regarded as a *nomen dubium* within the onchidiids (Goulding et al. 2018b: 96), which meant that it could potentially refer to *Peronia* slugs. Two syntypes (9/6 and 7/4 mm) of *Onchidium cinereum* were located recently in the MNHN collections (MNHN-IM-2000-33703): based on these syntypes, it is very likely that *Onchidium cinereum* refers to *Wallaconchis* slugs. *Onchidium cinereum* was described by Quoy and Gaimard (1833: 661, pl. 15, fig. 29) based on specimens from the island of “Tonga-Tabou” (Tonga). Note that the correct date of publication is 1833, not 1832, based on the collation by Sherborn and Woodward (1901b). There is only one jar at the MNHN with the following information (the label is not the original label): “*Oncidiella*, Tongatabou, Mrs. Quoy et Gaimard, 1829.” The sizes provided by Quoy and Gaimard (13 to 15 mm long), likely for live animals, approximately match the preserved specimens considering preservation. The large syntype was dissected prior to the present study and was found empty with no internal organs. The small syntype, still intact, was opened for the present study. It is an immature individual with no reproductive parts. However, its intestinal loop of type I, the lack of rectal gland, and the lack of dorsal gills all indicate that it belongs to a *Wallaconchis* species. However, because the penial apparatus could not be checked, *Onchidium cinereum* is regarded as a *nomen dubium*, even though there is only one *Wallaconchis* species known so far in southwestern Pacific Ocean. Past authors transferred *O. cinereum* to *Peronia* (Oken 1834a: 287) or *Onchidella* (e.g., Gray 1850: 117; Adams and Adams 1855: 234), or just kept the original combination (Semper 1882: 286–287; Plate 1893: 142; Bretnall 1919: 319; Hoffmann 1928: 68, 81).

*Peronia alderi* JE Gray, 1850 was created by JE Gray (1850: 117) for a slug illustrated by his wife ME Gray (1850: pl. 226, fig. 3) and which Alder had apparently identified as *P. punctata* in a manuscript: the only information associated with that illustration says “P. Alderi. P. punctata, Alder, MSS, t. 226. f. 3.” That slug clearly belongs to *Peronia*, based on the presence of dorsal gills. Alder and Hancock (1855: 34) briefly mentioned “*Onchidium punctatum* (*Peronia Alderi*, Gray)” in the context of a comparison between the dorsal gills in onchidiids and those in nudibranchs. However, given that no type locality is indicated and that no type specimen could be located (of which the label could have potentially indicated the type locality), *Peronia alderi* must be regarded as a *nomen dubium* (Table 1). Semper (1882: 268) transferred *P. alderi* to *Onchidium* but could not make any decision regarding its status because of insufficient data. Even though he does not seem to have examined any material, and for unclear reasons, Hoffmann (1928: 68, 72) mentioned New Guinea and the Torres Strait as records for *Peronia alderi* which he regarded as a synonym of *Onchidium verruculatum*.

*Peronia acinosa* was described by Gould (1852: 291–292; 1856: pl. 21, fig. 384a), based on an unspecified number of type specimens from Fiji Islands. *Peronia acinosa* may or may not refer to an onchidiid species, mostly because its long ocular tentacles lack eyes at their tip (some onchidiids illustrated by Gould distinctly have ocular tentacles with eyes at their tip), its color would be very unusual for an onchidiid (deep beryl-green dorsum and slatey violet foot), and it is “everywhere closely covered with large rounded papillae” (which are not characteristic of onchidiids). Also, the type material could not be located. Johnson (1964: 36) could not find it either. Therefore, *Peronia acinosa* is regarded here as a *nomen dubium* (Table 1). Adams and Adams (1855: 234) transferred *P. acinosa* to *Onchidella*. Bretnall (1919: 326) thought that *P. acinosa* was a valid name, although he admitted that data were insufficient. Hoffmann (1928: 68, 102) rejected that *P. acinosa* could be an *Onchidella* and questioned that it could be a *Peronia*, and proposed (with a question mark) that it could be a synonym of *Onchidina australis*. Given that, for instance, Gould described large rounded papillae, *P. acinosa* clearly does not refer to *Onchidina* slugs.

The type material of *Peronia corpulenta* Gould, 1852, described from Direction Island [Namena Island], Fiji, could not be located. Johnson (1964: 60) could not find it either. The type material seems to consist of a holotype, by monotypy, because only one animal size (63/25 mm) is provided in the original description. Gould’s (1852: 293) brief written description of the external appearance does not mention dorsal gills or dorsal eyes. Gould’s (1856: pl. 22, fig. 385a) illustration of the dorsal notum is colorful and quite pretty, but it also lacks dorsal gills and eyes. That being said, gills can only be seen when individuals are relaxed in water, and dorsal eyes often are difficult to see when dorsal papillae are retracted. Its animal length (63 mm) is compatible with both *P. peronii* and *P. verruculata*. That *Peronia corpulenta* refers to a *Peronia* species is possible but not certain; it is not even sure that it refers to an onchidiid species and thus is regarded as a *nomen dubium* (Table 1). Based on Gould’s information, past authors transferred *P. corpulenta* to *Onchidella* (Adams and Adams 1855: 234), regarded it as a possible synonym of *Onchidium peronii* (Plate 1893: 172; Bretnall 1919: 311; Labbé 1934a: 190), as a synonym of *Onchidium peronii* (Hoffmann 1928: 71), or as a questionable valid species name (Bretnall 1919: 326).

Stimpson (1855: 380) described *Onchis fruticosa* from Kikaisima, i.e., Kikaijima (ca. 28°30'N), between Kyushu and Okinawa, Japan. The brief description, restricted to the external morphology, clearly indicates that this name refers to a *Peronia* species, because some dorsal papillae bear one to three “oculiform black dots at their summits” and papillae on the posterior half of the body are “styliform branches” The length is given as 25 mm. *Peronia verruculata* is definitely present there and, possibly, *P. okinawensis* too. It cannot be excluded that *P. setoensis* could be found there as well; therefore, *O. fruticosa* is regarded as a *nomen dubium* (Table 1). To our knowledge, all past authors have overlooked Stimpson’s (1855) binomial.

Tapparone Canefri (1874: 101–102, pl. II, fig. 1) described *Onchidella griseofusca* based on specimens from Singapore. Note that the original spelling “*griseo-fusca*” needs to be corrected by removing the hyphen (ICZN 1999: Article 32.5.2.3). The type material could not be located. Tapparone Canefri’s (1874: pl. II, fig. 1) original illustration is useless, and *Onchidella griseofusca* could not be identified and compared to any of the onchidiid species we collected in Singapore. Martens (1897: 128) briefly mentioned the existence of *Onchidella griseofusca* without any new material. Hoffmann (1928: 85–86) arbitrarily transferred *Onchidella griseofusca* to *Onchidium* but could not determine whether it was an *Onchidium* or a *Platevindex* (as *Oncis*) species. *Onchidella griseofusca* clearly is a *nomen dubium* (Dayrat et al. 2016: 37, 2017: 1893). It could possibly refer to a *Peronia* species or *Marmaronchis vaigiensis*, but it does not refer to an *Onchidella* species (Table 1).

Semper (1880: 257–258; 1882: pl. XXI, figs 2–4) described *Onchidium nebulosum* from Aibukit, Palaos (Palau) based on a holotype (40/30 mm), by monotypy (ZMB/Moll 39040). *Onchidium nebulosum* clearly applies to a *Peronia* species because the notum of the holotype bears dorsal gills. The holotype was fully dissected by Semper. Its dorsum bears 16 papillae with eyes. The radula is missing. Male parts are missing, except for pieces of the flagellum of the penial accessory gland and possibly of the deferent duct which remain in a small vial. The female (hermaphroditic) posterior parts are still in place inside. The digestive system was destroyed and the type of the intestinal loops could not be determined. Semper did not indicate the length of the spine of the accessory penial gland nor the length of the muscular sac, which is missing. Without any of those critical characters, it is impossible to determine the application of *Onchidium nebulosum*, especially given that both *P. verruculata* and *P. peronii* are known to be present in Palau and that both *P. okinawensis* and *P. platei* could potentially be there as well. As a result, *O. nebulosum* is regarded as a *nomen dubium* even though it is clear that it applies to a *Peronia* species (Table 1).

Plate (1893: 171–172) described a “medium-sized” specimen which he identified as *Onchidium nebulosum*, from Pohnpei, Micronesia, which is 2600 km east of Palau. Plate’s description is problematic for at least two reasons. First, the presence of dorsal gills is not mentioned, which means that it is not certain that Plate did examine a *Peronia* individual. Second, Plate indicated a series of traits and measurements (intestinal loops of type I, muscular sac of the accessory penial gland 11 mm long, spine of the accessory penial gland 2.5 mm long, retractor muscle of the penis inserting near the heart) but those cannot be compared to the original description of *Onchidium nebulosum* because Semper did not mention them. Thus, there is no reason to admit that Plate did examine what Semper had originally described as *Onchidium nebulosum*. If Plate examined a specimen with dorsal gills, then it possibly was an individual of *P. okinawensis*, from Okinawa, which is 3800 km west of Pohnpei. Some of the characters seem to match (Table 4). It is unlikely, however, that Plate examined a specimen of *P. platei*, because most characters do not match (Table 4). Finally, it is not excluded that Plate examined a *Paromoionchis* or a *Laspionchis* individual instead (with no dorsal gills). Bretnall (1919: 310–311), Hoffmann (1928: 71), and Labbé (1934a: 224) assumed that Plate’s identification was correct and accepted Palau and Pohnpei as two records of *O. nebulosum*, but did not add any new material.

*Onchidium multiradiatum* Semper, 1882 refers to an onchidiid species which belongs to *Peronia* or not, but is regarded as a *nomen dubium* because the type locality is unknown (Table 1). Semper (1882: 269) mentioned two individuals in the original description. One syntype, 30/22 mm, was located (ZMB/Moll 39026): the male and female parts are missing and the region of the male opening is partly destroyed; the radula is still present but the type of the intestinal loops cannot be determined; dorsal gills are not obvious but are present. Plate (1893: 141) merely mentioned *Onchidium multiradiatum* as an available species name. Both Hoffmann (1928: 79–80) and Labbé (1934a: 225) listed *Onchidium multiradiatum* as a valid species name, exclusively based on Semper’s information. Hoffmann also briefly compared it to *Onchidium griseum*, which seems to refer to a species of *Paromoionchis* but also is a *nomen dubium* because its type locality is unknown (Dayrat et al. 2019: 70).

*Quoya indica* Labbé, 1934a, the type species, by monotypy, of the genus *Quoya* Labbé, 1934a, was originally described based on three specimens for which, according to Labbé, there was no information other than the locality, “Mer des Indes,” i.e., Indian Ocean. Because the type locality is too vague, *Quoya indica* is regarded as a *nomen dubium*. Three specimens (16/8, 10/8, and 7/5 mm) were found (MNHN-IM-2000-33679), which seem to match the material used by Labbé to describe *Quoya indica*. The only information on the labels tells us that they are from the “Mer des Indes.” No collector or collecting date are indicated. As often with Labbé, no species identification is indicated either. Those three specimens possibly are the syntypes of *Quoya indica*. They dried and are very hard and poorly preserved. However, dorsal gills are present on the largest specimen and possibly on the smallest specimen too. Labbé (1934a: 216, fig. 51) described a double male opening (with the openings of the penis and of the accessory penial gland being separated). This could not be confirmed and is by no means a trait of generic value. Indeed, the opening of the penis and the opening of the accessory penial gland occasionally appear to be separated due to preservation (when the vestibule is everted). Internal characters could not be checked. In particular, the presence of intestinal loops of type V (not illustrated by Labbé) and of an accessory penial gland (Labbé 1934a: fig. 53) could not be confirmed. There is at the MNHN another jar (numbered “31” on an old label) with a single specimen from the “Mer des Indes.” However, that specimen is identified as “*Oncidium*,” suggesting that it is not part of the type series of *Quoya indica*. Instead, it possibly is the non-type specimen that Labbé (1934a: 204) identified as *Scaphis punctata*. It is confirmed here that *Quoya indica* refers to a *Peronia* species (dorsal gills are present on the largest possible syntype), and that, therefore, *Quoya* is a junior synonym of *Peronia*. However, because the type locality is too vague and because no internal characters could be confirmed, *Peronia indica* is regarded as a *nomen dubium* (Table 1).

The species-group name *hombroni*, created before 1961 as a variety name, is now of subspecific rank (ICZN 1999: Article 45.6.4). Labbé (1934a: 202, fig. 23) described *Paraperonia gondwanae hombroni* based on one specimen from the Torres Strait, Australia. Originally, no jar clearly labeled as the type material of *P. gondwanae hombroni* was found at the MNHN. However, only one old jar was found at the MNHN with material collected from the Torres Strait by M. Hombron aboard the *Astrolabe*, as in Labbé’s original description of *P. gondwanae hombroni* (MNHN-IM-2000-33694). Therefore, it most likely contains the holotype, by monotypy, of *P. gondwanae hombroni*. Unfortunately, there is little doubt that whatever is in that jar is not an onchidiid (it seems to be an empty notum of a nudibranch). Three explanations are possible. First, this material (MNHN-IM-2000-33694) is not the holotype of *P. gondwanae hombroni*, even though all the collecting information matches. Second, it was originally the holotype of *P. gondwanae hombroni*, but that holotype was switched by mistake with something completely different. Third, the material inside the jar is the material examined by Labbé, which means that he would have completely made up the description. Given the description of dorsal gills and of an accessory penial gland by Labbé (1934a: 202), it is likely that *P. gondwanae hombroni* applies to a *Peronia* species. However, the original description is problematic because Labbé (1934a: 202) writes that the intestinal loops are of type V and sometimes of type I, which is just impossible given that he examined only one individual. Because the type of the intestinal loops is uncertain, *P. gondwanae hombroni* cannot be applied reliably to any *Peronia* species and is regarded as a *nomen dubium* (Table 1).

As discussed in detail in our revision of *Paromoionchis*, *Onchidium straelenii* Labbé, 1934b is a *nomen dubium* (Dayrat et al. 2019: 70–72). The examination of the two syntypes used by Labbé (RBINS I.G.9223/MT.3823) revealed that Labbé’s original description is erroneous regarding several important characters. In particular, Labbé (1934a: 213) subsequently transferred *Onchidium straelenii* to his genus *Scaphis* based on dorsal gills being supposedly numerous and highly ramified. However, there are no gills at all on the dorsal notum so it is clear that *Onchidium straelenii* cannot be classified in *Peronia*. *Onchidium straelenii* was arbitrarily placed in the genus *Onchidium* but it clearly should not be classified in *Onchidium* because several traits, such as the lack of a rectal gland, are incompatible with *Onchidium* (Dayrat et al. 2016). The generic placement of *Onchidium straelenii* remains unclear, hence its status as a *nomen dubium* (Table 1).

### Species delineation

*Peronia* species cannot be distinguished externally, except for the longest individuals of *P. peronii* (more than 100 mm). However, they all differ internally, apart from *P. platei* and *P. setoensis* which cannot be distinguished (Table 4). This situation is similar to what has been observed in several other onchidiid genera: in *Wallaconchis*, *Laspionchis*, *Paromoionchis*, and *Peronina*, species cannot be distinguished externally but they all differ with respect to their copulatory apparatus (Dayrat et al. 2019a, b; Goulding et al. 2018b, c). The special difficulty in *Peronia* is that species differ in minute details. In other genera, species differences tend to be obvious. For instance, an accessory penial gland is present in *Peronina tenera* and absent in *P. zulfigari* (Goulding et al. 2018c). In comparison, *Peronia* species may only differ with respect to the length of the spine of the accessory penial gland (Table 4). This has made it very difficult for past authors to interpret anatomical differences. *Peronia* species diversity has been interpreted in two opposite directions, both of which were unfortunately erroneous. At one end of the spectrum, Labbé (1934a) considered that every single difference justified the creation of a new taxon name. As a result, while Hoffmann (1928) accepted only six species of slugs with dorsal gills, all being still classified in *Onchidium* along with 34 species without dorsal gills, Labbé (1934a: 187) thought that there were five genera and 21 species of slugs with dorsal gills. However, the present monographic revision shows that only one of all Labbé’s new names is valid (Table 1): *Peronia madagascariensis* (Labbé, 1934a). At the other end of the spectrum, more recent authors accepted only two species, *P. peronii* and *P. verruculata*, which they could not even really distinguish (e.g., Solem 1959: 38–39; Marcus and Marcus 1970: 213–214; Britton 1984: 183).

*Peronia* is a taxon for which the use of DNA sequences as an independent test for species delineation has been indispensable. Without DNA sequences, it would have been impossible to determine which anatomical traits differ or not among species, which is perfectly illustrated by the species diversity of *Peronia* in Japan. Past authors have somehow sensed that there were more than one species in Japan but could not tell them apart (e.g., Baba 1958; Katagiri and Katagiri 2007; Ueshima 2007). Our data show that there are four species in Japan, two being endemic (Fig. 6). After species are delineated using DNA sequences, their anatomical differences become clear: for instance, *P. setoensis* is the only species in Japan with intestinal loops of type V (Table 4).

The present study also demonstrates that even though mitochondrial COI sequences are necessary, they must not be used blindly. Indeed, if one were to take into account only mitochondrial DNA sequences (Figs 2, 5), one might think that there are up to 16 *Peronia* species: *P. verruculata* could be split into five distinct species, and *P. peronii*, *P. platei*, and *P. griffithsi* could be split into two species each. However, our two other data sets (nuclear DNA sequences and comparative anatomy) strongly suggest that those merely are cases of species with high genetic structure: all individuals of *P. platei*, for instance, are completely indistinguishable anatomically, and nuclear ITS2 sequences do not support the existence of two distinct taxa in *P. platei*. The long geographic distances between sampling sites (e.g., Hawaii and Papua New Guinea for *P. platei*) may partly explain the high intra-specific genetic structure. In cases where mitochondrial units are sympatric (*P. verruculata* units #1 and #2 overlap in southeastern Sumatra, and *P. verruculata* units #1 and #3 overlap in Singapore), genetic distances could be explained by the fact that those mitochondrial lineages were isolated for some time before coming into contact again. The importance of investigating nuclear DNA sequences as well as comparative anatomy has been demonstrated in other onchidiid genera, especially in *Paromoionchis*, *Wallaconchis*, and *Peronina* (Dayrat et al. 2019a; Goulding et al. 2018b, c).

Species are not externally cryptic in all onchidiid genera: the six *Melayonchis* species and the four *Onchidium* species can all be distinguished in the field on external traits (Dayrat et al. 2016, 2017, 2019c, d). When species can be distinguished externally, they are also unequivocally supported by both mitochondrial and nuclear sequences, i.e., DNA sequences do not support any cryptic diversity within those *Melayonchis* and *Onchidium* species. True cryptic diversity remains exceptional in onchidiids: *Marmaronchis vaigiensis* (Quoy & Gaimard, 1825) and *M. marmoratus* (Lesson, 1831) cannot be distinguished externally or internally (Dayrat et al. 2018).

### Types of intestinal loops

It is not an exaggeration to say that identifying the types of intestinal loops, originally defined by Plate (1893) and Labbé (1934a), has remained challenging for authors. A thorough re-examination of the specimens examined by Plate and Labbé in the context of the complete revision of the Onchidiidae has shown that even they were confused about intestinal types. For instance, Dayrat et al. (2019c: fig. 2) demonstrated that Plate’s original definition of the type III was based on an erroneous number of dorsal loops in a specimen of *Onchidium stuxbergi* (Westerlund, 1883). Labbé (1934a) also repeatedly made mistakes (see all the species remarks above), the most notorious being his original description of *P. anomala*: the specific name *anomala* was created to emphasize that the intestinal loops of that species were anomalous (i.e., of type II instead of type I as in most *Peronia*), but the intestinal loops of the type material of *P. anomala* are of type I (Figs 1, 86B). In that context, it is thus not too surprising that Maniei et al. (2020a) described the intestinal loops of *P. persiae* as of type II although they clearly are of type I, with a transitional loop at 5 o’clock (see above remarks on *P. verruculata*).

Hopefully, the method that Dayrat et al. (2019b: fig. 1; 2019c: fig. 2; 2019d: fig. 13) recently introduced to identify types of intestinal loops will help put an end to that confusion. This method is based on the coloration of different sections of the intestinal loops and, most importantly, takes individual variation into account (Fig. 1). It is very important to note that this method does not redefine the types of intestinal loops, it merely clarifies them, and the difference between the types I and II originally defined by Plate and Labbé is maintained. According to Plate and Labbé, type I is characterized by a transitional loop oriented to the right in dorsal view, at 3 o’clock (Dayrat et al. 2019b: Fig. 1A), and type II is characterized by a transitional loop oriented to the left in dorsal view, at 9 o’clock (Dayrat et al. 2019b: fig. 1C). The reality is that the orientation of the transitional loop varies between individuals, but a left or right orientation of the transitional loop remains true (Fig. 1): in type I, the transitional loop is oriented between 12 and 6 o’clock (always to the right in dorsal view, as stipulated by Plate and Labbé); in type II, the transitional loop is oriented between 6 and 12 o’clock (always to the left in dorsal view, as stipulated by Plate and Labbé).

Only the types I and V are found in *Peronia* (Table 4, Fig. 1): the transitional loop of type I is always oriented to the right (from 12 to 3 o’clock, or from 3 to 6 o’clock); there is no transitional loop in type V. To this day, there is no positive record (proven with an illustration) of intestinal loops of type II in *Peronia*. In the future, a few individuals may be shown to exceptionally possess a transitional loop oriented at 7 o’clock (which strictly speaking would correspond to a type II), but this has never been observed among the hundreds of *Peronia* specimens dissected for the present study.

### Geographic distribution

The genus *Peronia* includes the two most widespread onchidiid species, *P. verruculata* and *P. peronii*, as well as species that are endemic to comparatively small areas, at least according to the current data (Fig. 6): *P. setoensis* and *P. okinawensis* are endemic to Japan, and *P. willani* is endemic to Northern Territory. One reason may be the development mode. In Japan, on the eastern coast of Honshu, near Sagami Bay (ca. 35°N), Katagiri and Katagiri (2007) documented two *Peronia* species, one characterized by a planktotrophic development (called “Isowamochi”) and the other characterized by a direct development (called “Minneawamochi”). Most likely, those species correspond to *P. verruculata* (unit #1) and *P. setoensis*, which are the only two *Peronia* species found north of 30N in Japan (Fig. 6). Another reason may be that all species cannot compete ecologically with *P. verruculata*, one of the most abundant onchidiid species in the Indo-Malayan region (*P. tumidus* is also extremely abundant but it lives in mangroves, not in the rocky intertidal, although both *P. verruculata* and *P. tumidus* often are found together on muddy sand). A third reason may be related to diversification history. The fact that several species (*P. okinawensis*, *P. platei*, *P. setoensis*, *P. sydneyensis*, *P. willani*) are characterized by narrow distribution ranges at the periphery of broadly-distributed species (*P. griffithsi*, *P. peronii*, *P. verruculata*) raises the question of whether peripatric speciation events may have occurred. Phylogenetic relationships of sister species suggest that *P. okinawensis* could have emerged peripatrically from *P. peronii*. As for the other species, it remains uncertain because relationships among clades E, F, and G are still unclear (Figs 2–4). Finally, given that they are sister species, it is most likely that *P. willani* and *P. sydneyensis* are the result of a recent allopatric speciation (the Torres Strait serving as a biogeographic barrier).

In the future, it will be necessary to investigate the phylogenetic relationships of populations of *P. verruculata* from the regions from where no fresh material could be obtained, especially the northeastern Indian Ocean (the coasts of Somalia, Yemen, and Oman), the Persian Gulf, the Red Sea, and southern India. It will also be necessary to include fresh material from new localities for *P. peronii* (its distribution provided here is based on many specimens identified only based on anatomy). Dozens of new specimens of *P. peronii* may reveal some higher genetic structure within *P. peronii*, as observed in *P. verruculata*, given that both species are widely distributed. At the moment, the low level of genetic structure within *P. peronii* (compared to *P. verruculata*) may simply be due to the fact that our mitochondrial analyses include thirteen specimens of *P. peronii* while they include 102 specimens of *P. verruculata*. Populations of *Peronia* slugs also need to be investigated in southeastern Australia (southern Queensland and northern New South Wales) to determine more precisely the geographic range of *P. verruculata*. Also, it is possible that species that are endemic based on current data (*P. okinawensis*, *P. setoensis*, *P. willani*) will be found elsewhere and will thus be characterized by a wider range. Finally, it is not excluded that additional new species will be found.

## Supplementary Material

XML Treatment for Peronia

XML Treatment for Peronia peronii

XML Treatment for Peronia okinawensis

XML Treatment for Peronia madagascariensis

XML Treatment for Peronia platei

XML Treatment for Peronia setoensis

XML Treatment for Peronia griffithsi

XML Treatment for Peronia sydneyensis

XML Treatment for Peronia willani

XML Treatment for Peronia verruculata
